# Combined Experimental and Computational Study of Ruthenium *N*-Hydroxyphthalimidoyl Carbenes in Alkene Cyclopropanation Reactions

**DOI:** 10.1021/acscatal.1c02540

**Published:** 2021-08-18

**Authors:** Ferran Planas, Matteo Costantini, Marc Montesinos-Magraner, Fahmi Himo, Abraham Mendoza

**Affiliations:** Department of Organic Chemistry, Arrhenius Laboratory, Stockholm University, SE-106 91 Stockholm, Sweden

**Keywords:** transition-metal catalysis, asymmetric catalysis, cyclopropanes, kinetics, DFT calculations, redox-active carbenes

## Abstract

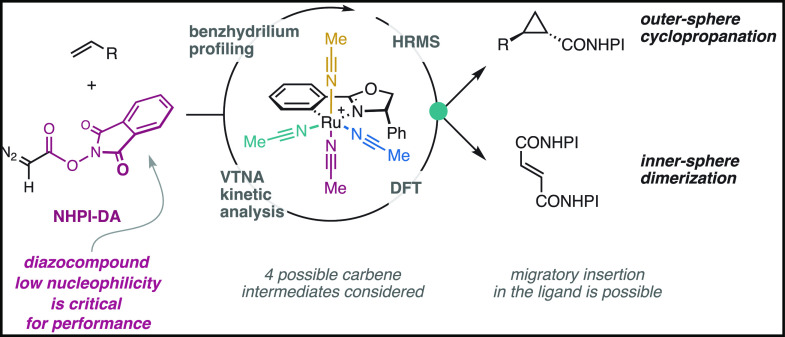

A combined experimental–computational approach has been used to study the cyclopropanation reaction of *N*-hydroxyphthalimide diazoacetate (NHPI-DA) with various olefins, catalyzed by a ruthenium-phenyloxazoline (Ru-Pheox) complex. Kinetic studies show that the better selectivity of the employed redox-active NHPI diazoacetate is a result of a much slower dimerization reaction compared to aliphatic diazoacetates. Density functional theory calculations reveal that several reactions can take place with similar energy barriers, namely, dimerization of the NHPI diazoacetate, cyclopropanation (inner-sphere and outer-sphere), and a previously unrecognized migratory insertion of the carbene into the phenyloxazoline ligand. The calculations show that the migratory insertion reaction yields an unconsidered ruthenium complex that is catalytically competent for both the dimerization and cyclopropanation, and its relevance is assessed experimentally. The stereoselectivity of the reaction is argued to stem from an intricate balance between the various mechanistic scenarios.

## I Introduction

**Scheme 1:**
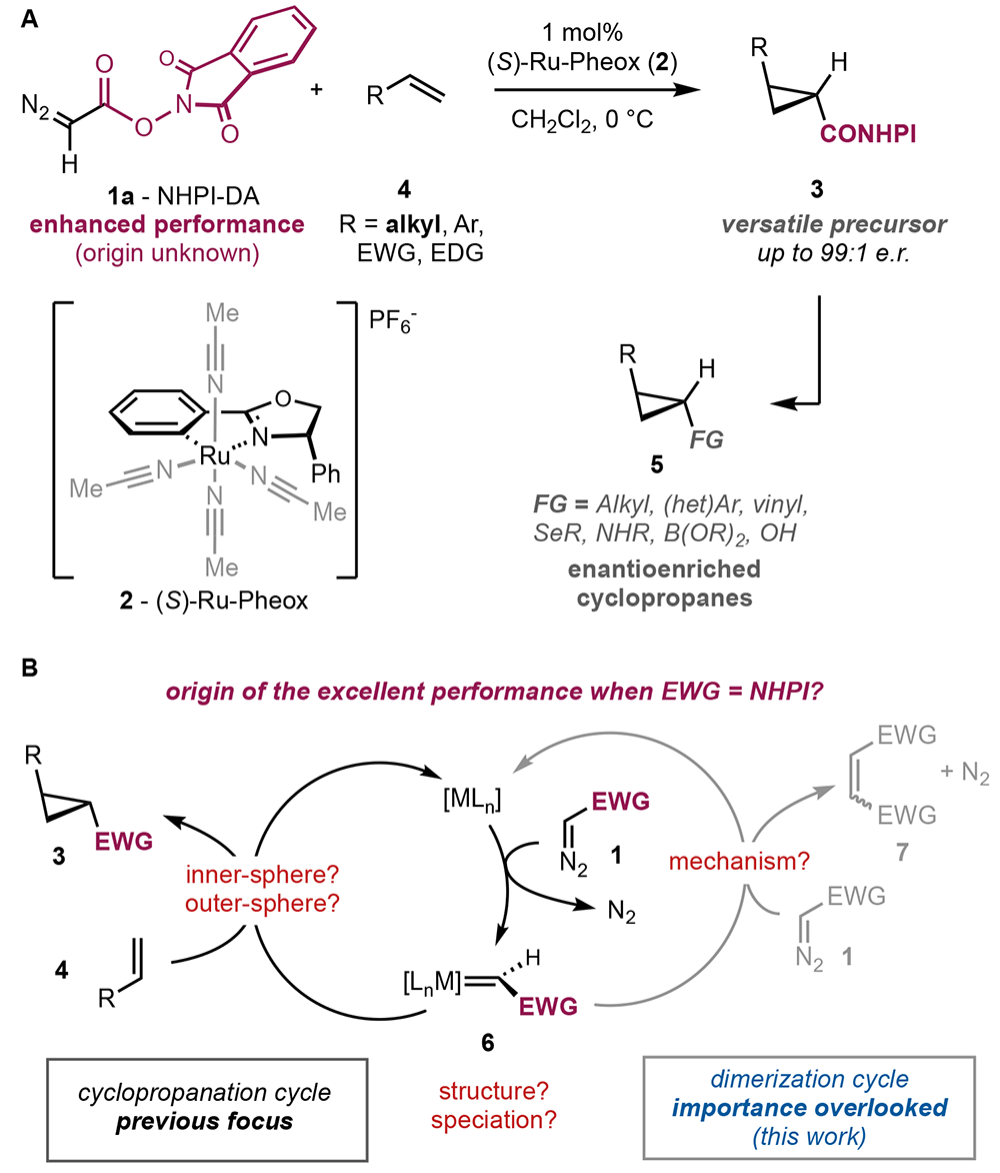
(A) Cyclopropanation of Unactivated Olefins Using NHPI-DA and Ru-Pheox by Mendoza and Co-workers;^5^ (B) Simplified Catalytic Cycles for the Cyclopropanation and Dimerization (DM) Reactions

Cyclopropanes have found wide use across organic chemistry, spanning from medicinal chemistry to more fundamental synthetic applications.^1^ One of the most versatile tools available for the synthesis of these compounds is the metal-catalyzed carbene-transfer reaction to abundant olefin feedstocks.^2^ Diverse methods have been developed over the years aiming at improving its generality, efficiency, and stereoselectivity.^3^ In this context, the cyclopropanation of aliphatic olefins has remained challenging in terms of efficiency and particularly enantioselectivity.^2i,2k,2n,2p,2q^ Recently, Mendoza and co-workers disclosed the use of a new *N*-hydroxyphthalimide diazoacetate (NHPI-DA, **1a**) reagent, which displayed unique reactivity in combination with Iwasa’s ruthenium-phenyloxazoline catalyst (Ru-Pheox, **2**).^4,5^ This system enabled the synthesis of highly enantioenriched cyclopropanes **3** from olefin starting materials **4**, including challenging aliphatic alkenes (Scheme 1A).^5^ The performance in the cyclopropanation reaction bestowed by the redox-active ester group in NHPI-DA (**1a**) surpassed that of any comparable diazo compound reagent in terms of olefin scope and enantioselectivity.^5^ Moreover, late-stage diversification of the resulting redox-active ester products **3** enabled the unified enantioselective synthesis of cyclopropylamines, cyclopropanols, alkyl-, (hetero)aryl-, vinyl-, boryl-, or selenyl-cyclopropanes (**5**) alike.^6^

Generally, cyclopropanation reactions are proposed to occur through the generation of a metal carbene intermediate **6** from the diazocompound **1** and the catalyst (Scheme 1B). This carbene **6** reacts with the olefin **4** to yield the cyclopropane product **3** and regenerate the metal catalyst. Gathering more detailed information on unstabilized carbenes **6** is particularly problematic due to their fast reactivity that makes high-quality reaction monitoring difficult (*e.g.*, NMR spectroscopy). Thus, it is common that mechanistic information is extrapolated from the final composition of the reaction mixture.^7^ As far as we are aware, a few kinetic studies in two specific catalytic systems have been performed,^7,8^ revealing that cyclopropanation can occur through both inner-sphere (IS) and outer-sphere (OS) mechanisms. Computational investigations have provided more detailed information on these pathways^9,10^ and some exceptional alternatives.^11^ In the inner-sphere mechanism, a metallacyclobutane intermediate^12^ is formed upon a [2 + 2] cycloaddition between the carbene and the olefin ligands.^9^ The outer-sphere mechanism, on the other hand, involves intermolecular reaction with the olefin either in a single step or *via* radical intermediates.^10^ More recent computational work has focused on catalysts that promote the outer-sphere mechanism, employing bulky ligands or dimeric metal species.^11,13^ Mechanistic studies on simpler catalysts of the Ru-Pheox type are, however, still rather scarce.^14^ While previously studied copper^7^ and rhodium^8^ catalysts have only one or two coordination sites available, the situation is greatly complicated in the case of Ru-Pheox (**2**) due to its octahedral *C*_1_-symmetric environment with four inequivalent positions that could lead to different intermediates (see Scheme 1A).

The dimerization of diazocompounds (**1**) is a major side reaction that limits the efficiency of metal-catalyzed cyclopropanation reactions (Scheme 1B). Limited information exists on this process, but related cross-dimerization reactions with copper^7b^ or rhodium^8b^ catalysts occur through outer-sphere addition of the diazocompound **1** on the metal carbene **6** to produce the olefin byproduct **7**. To overcome this obstacle, research has focused on developing metal complexes that are more selective for the cyclopropanation process.^7,15−17^ Often, the intrinsic selectivity is low, and experimentally, it has been found that large excess of either the olefin substrate (**4**) or syringe-pump slow addition of the diazocompound (**1**) is beneficial. However, inefficient use of one of the coupling partners is often unavoidable.^2a−2c,2e−2q,7a,15−17^ This competition is experimentally more severe when using less reactive olefins such as aliphatic and acrylate derivatives. Compared to conventional diazoacetate reagents, the redox-active ester group in NHPI-DA (**1a**) bestowed remarkable cyclopropanation/dimerization selectivity even on these very challenging substrates, but its origin is yet unknown.^5^

In principle, we reasoned that the improved performance could stem from either (1) an unusually high affinity of the redox-active metal carbene intermediate for olefin substrates (fast cyclopropanation), (2) a particularly low nucleophilicity of the diazocompound (slow dimerization), or (3) a combination of both. To the best of our knowledge, lowering diazocompound nucleophilicity has not been targeted specifically to limit the dimerization side reaction, and efforts have focused instead on catalyst design to accelerate the desired cyclopropanation process.^7,15−17^ The ester substituents in diazocompounds have been modified to enhance diastereo- and/or enantioselectivity by adjusting their size and/or enabling chelate intermediates with the catalyst.^4,18^ The unique performance of NHPI-DA (**1a**) in combination with Ru-Pheox (**2**) observed by Mendoza and co-workers,^5^ and the limited experimental data on the mechanism of cyclopropanation and dimerization reactions (particularly on ruthenium metallacyclic catalysts), prompted us to conduct a joint experimental–computational study to discern the key mechanistic features that enable the unusual and interesting applications that have been recently discovered.^5,19^

## II Results

In the following, we will first present the kinetic profiling of the dimerization reaction followed by the computational description of its reaction mechanism. Next, we will discuss the experimental and computational investigations of the cyclopropanation reaction of two olefins of different reactivities, an aromatic and an aliphatic one. Finally, we will present the computational discussion of the possible factors governing the selectivity of the cyclopropanation reaction.

### II.I Kinetics of the Dimerization Reaction and Nucleophilicity Benchmarking

The origin of the unexpected performance in the NHPI-DA (**1a**)/Ru-Pheox (**2**) system has been studied experimentally through high-resolution mass spectroscopy (HRMS), visible spectroscopy, and dinitrogen evolution measurements. The latter involved a continuous monitoring of the pressure inside a sealed reactor with a digital wireless sensor (see the Supporting Information (SI) for details).^20^ This allows us to measure the conversion of NHPI-DA over time with sufficient data density and accuracy to reliably undertake detailed kinetic analyses. Initially, we set out to explore the behavior of the dimerization process. Reactions run with or without previous saturation of the solvent with N_2_ exhibited identical data under standard conditions, thus validating the technique to evaluate the progress of the experiments (see the SI). Finally, all reactions were analyzed by ^1^H NMR at the end of the measurement to determine the conversion reached.

**Figure 1:**
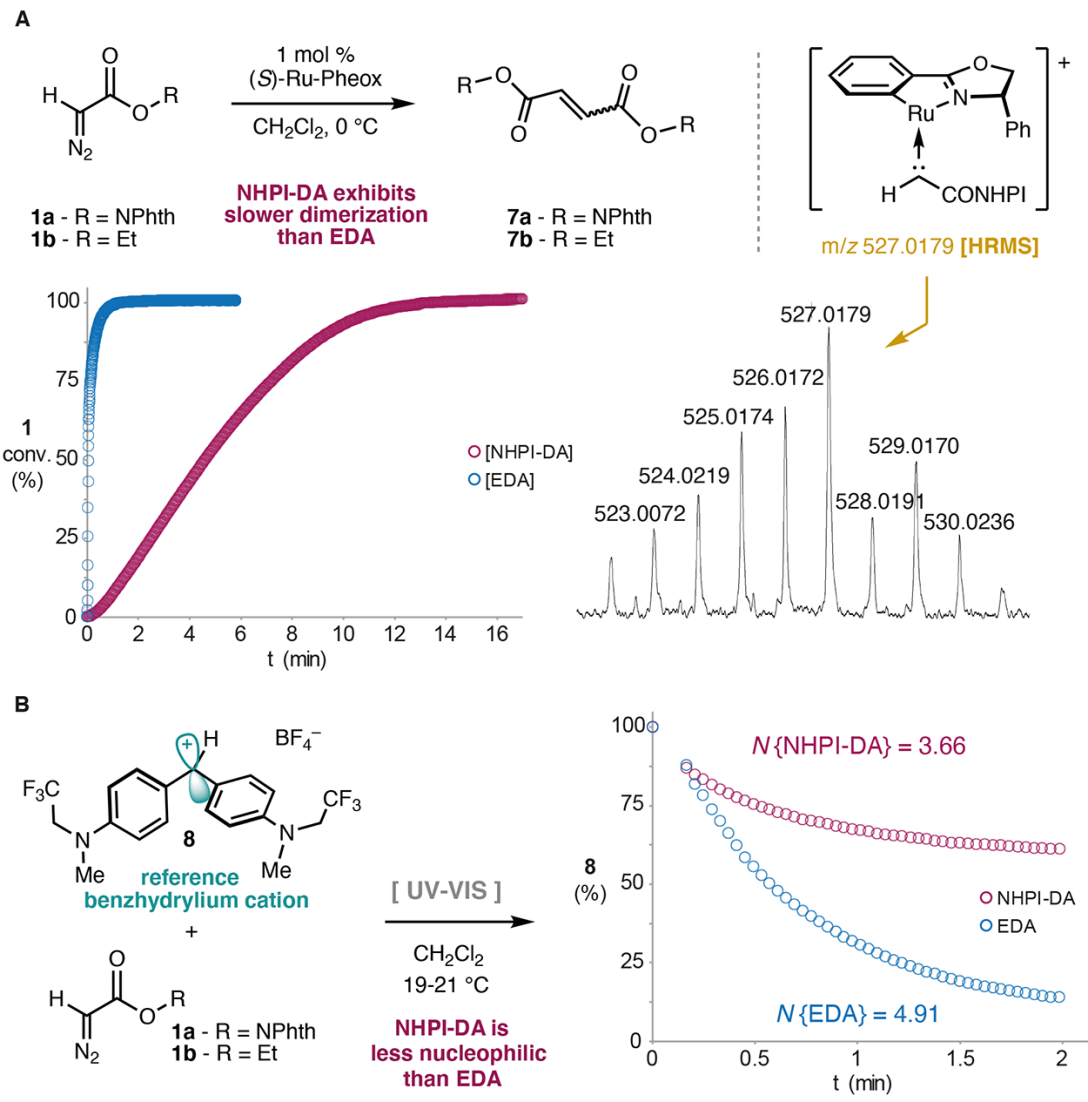
(A) N_2_ evolution profile of the dimerization of NHPI-DA and EDA in the presence of Ru-Pheox in CH_2_Cl_2_ at 0 °C. Initial concentrations: [EDA]_0_ = [NHPI-DA]_0_ = 0.1 M, [**2**]_0_ = 0.001 M. Experimental high-resolution mass spectrum at *m*/*z* = 527 consistent with the molecular formula C_25_H_17_N_2_O_5_Ru. (B) Nucleophilicity benchmarking of NHPI-DA against EDA using a benzhydrylium cation reference electrophile.

Figure 1A (left) compares the kinetic profiles of the dimerization of the benchmark ethyl diazoacetate (EDA; **1b**) and NHPI-DA (**1a**) in the presence of catalytic amounts of Ru-Pheox (**2**).^21^ While the conventional diazoester EDA (**1b**) is fully consumed in less than 2 min, the dimerization of the redox-active diazocompound **1a** is unusually slow, requiring approximately 15 min to complete. The initial curvature of the kinetic trace may indicate a short induction period, but could also originate from the stabilization of the pressure detector. Interestingly, upon injection of the reaction mixture in an electrospray ionization (ESI)-HRMS instrument right after mixing of NHPI-DA (**1a**) and catalyst **2** in dichloromethane, a clear peak at *m*/*z* = 527 was detected (Figure 1A, right), whose isotopic pattern is consistent with the ruthenium redox-active carbene **6a**, despite its expected fleeting nature at room temperature. This species could not be observed after the reaction was over, and our efforts to isolate and characterize this species failed.

The slow dimerization of NHPI-DA could in principle be ascribed to a lower nucleophilicity of the diazocompound or to a slower reaction of its derived ruthenium-carbene intermediate. To distinguish these two possibilities, the nucleophilicity parameter of NHPI-DA (*N*{**1a**}) was determined using the benzhydryl cation reference electrophile method popularized by Mayr (Figure 1B).^22^ This method has the advantage of evaluating the intrinsic nucleophilicity of the diazocompound in the absence of any catalyst. Among other possibilities, the fluorinated cation **8** displayed the right reactivity and a convenient spectroscopic detection in the visible range (λ_max_ = 569 nm). After triplicate determination, NHPI-DA (**1a**) was found to be substantially less nucleophilic (*N*{**1a**} = 3.66) than the benchmark diazocompound EDA (*N*{**1b**} = 4.91),^22^ probably due to the electron-deficient *N*-hydroxyphthalimide ester. The lower nucleophilicity of NHPI-DA can explain its surprising cyclopropanation performance with challenging aliphatic and electrophilic olefin substrates^5^ through a slower dimerization side reaction.

### II.II Calculations of the Dimerization Reaction

**Figure 2:**
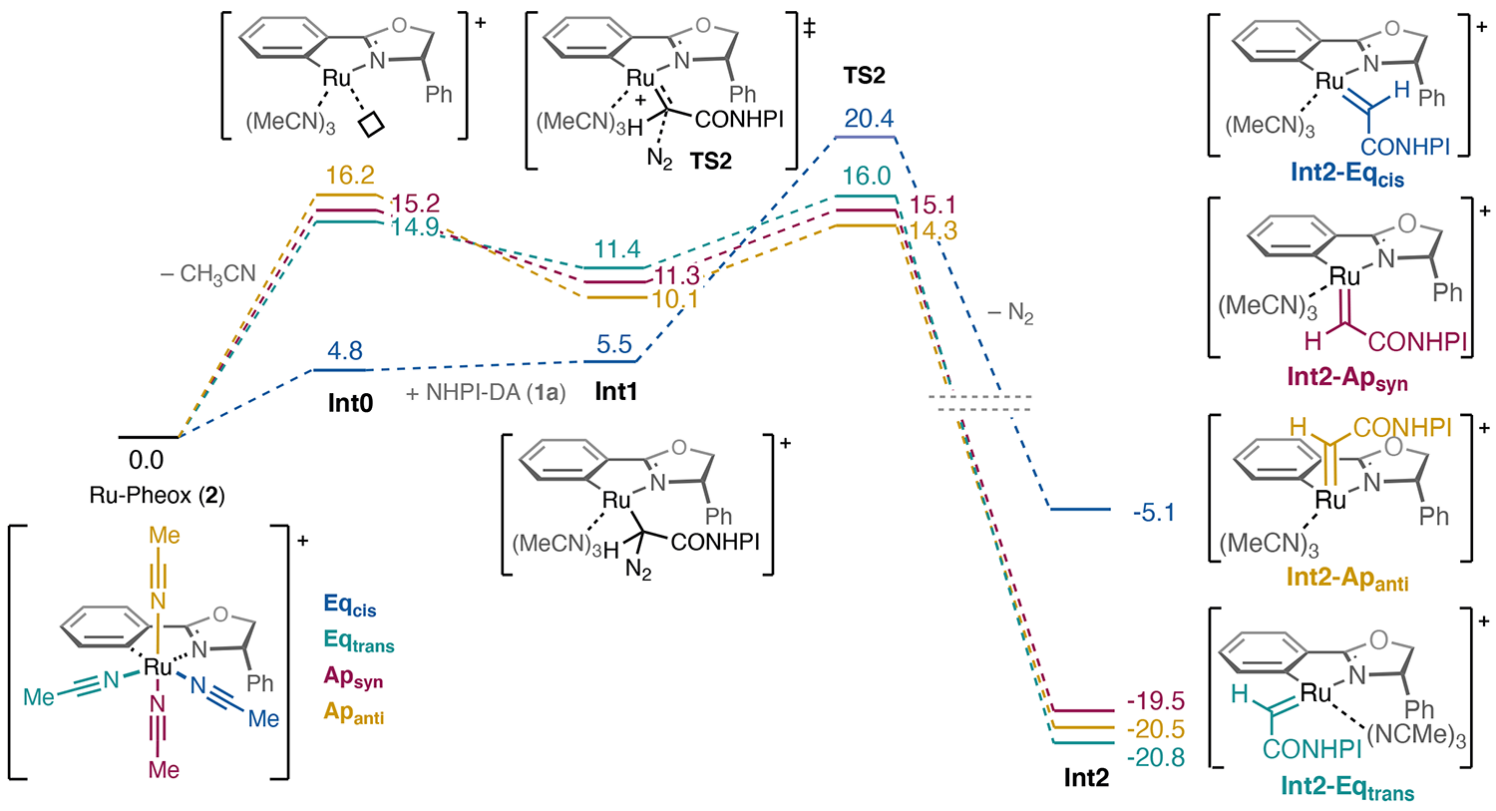
Calculated energies (kcal/mol) for the carbene formation step at the various positions of the Ru-Pheox (**2**) catalyst.

The computational investigation of the dimerization reaction started with the determination of the most stable species that can be formed from the precatalyst Ru-Pheox tetrakis(acetonitrile) complex and the NHPI-DA reagent in the dichloromethane solvent (see the SI). The calculations show that the most stable starting structure is the Ru-Pheox complex with four acetonitriles coordinated in an octahedral manner, consistently with the published X-ray diffraction (XRD) structure.^4^ We have confirmed by ^1^H NMR that four acetonitrile ligands remain coordinated in CD_2_Cl_2_ solution (see the SI), even in the presence of styrene (**4a**). It is important to note here that due to the lack of symmetry of the catalyst, the complex has four different positions where different species can bind (see Figure 2). Namely, there are two equatorial positions *trans* and *cis* to the oxazoline (labeled **Eq**_**trans**_ and **Eq**_**cis**_, respectively), and two apical coordination sites *anti* and *syn* to the phenyl substituent in the stereogenic center of the equatorial Pheox ligand (labeled **Ap**_**anti**_ and **Ap**_**syn**_, respectively).

The dimerization reaction starts with the binding of the NHPI-DA reagent to the catalyst, replacing one of the acetonitrile ligands. Experimentally, we have observed that the exchange of the four acetonitriles in the initial Ru-Pheox complex is certainly slower than the ^1^H NMR timescale, as evidenced by their sharp differentiated signals in solution (see the SI). In a dissociative mechanism, this transformation would proceed *via* the penta-coordinated complexes **Int0** that retained an octahedral coordination environment around the metal. Depending on the vacant position, formation of these complexes from the initial catalyst is endergonic by 5–16 kcal/mol (Figure 2). The subsequent binding of the NHPI-DA substrate results in complexes **Int1** calculated to be slightly lower in energy than **Int0**, but still considerably higher in energy than the initial complex. Next, the carbene intermediate is formed by dissociation of the dinitrogen, with calculated barriers of 14.3, 15.1, 16.0, and 20.4 kcal/mol for the **Ap**_**anti**_, **Ap**_**syn**_, **Eq**_**trans**_, and **Eq**_**cis**_ positions, respectively. The resulting carbene intermediates **Int2** are calculated to be more stable than the initial complex, by 5–21 kcal/mol (Figure 2). The different stabilities of the four intermediates **Int0** in the dissociative ligand exchange step (**2** → **Int1**) are in line with a previous computational work on this system, in which the dissociation of acetonitrile at **Eq**_**cis**_ was found to be more favorable than at the other three positions.^14c^ This result was considered an indication that carbene intermediate would preferably form at the **Eq**_**cis**_ position. However, it becomes apparent from our calculations that this position is the least likely to form the carbene, as seen from the higher barrier in the nitrogen evolution step (**TS2-Eq**_**cis**_) compared to the other three possibilities. This is probably a result of the strong *trans*-influence of the metallacyclic aryl in the Pheox ligand in the barrier and the stability of the resulting complex. It is also important to remark that the energy of **Int0** is similar to that of **TS2** at **Ap**_**anti**_, **Ap**_**syn**_, or **Eq**_**trans**_, and therefore a scenario in which the formation of the penta-coordinated complex partly determines the carbene(s) to be formed is also possible. Importantly, once the **Ap**_**anti**_, **Ap**_**syn**_, and **Eq**_**trans**_ carbenes are formed, they cannot interconvert. Attempts were made to find a feasible energy path for interconversion, but without success (see the SI).

**Figure 3:**
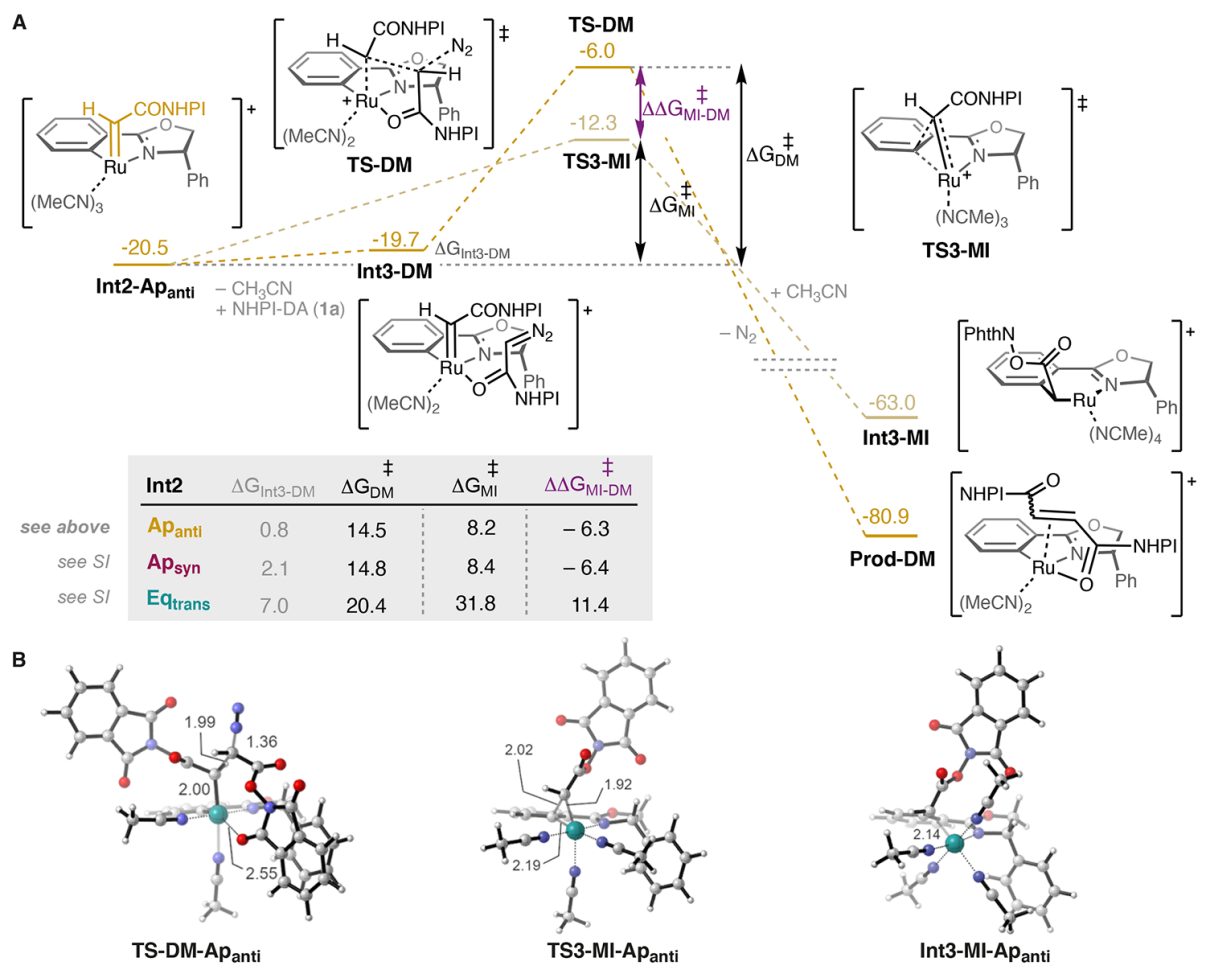
(A) Calculated free energy profile (kcal/mol) for the dimerization and migratory insertion (MI) reactions starting from the **Int2-Ap**_**anti**_ carbene. (B) Optimized structures of relevant TSs and intermediates. The inserted table shows the energies for reactions at the other positions, each relative to its own carbene intermediate **Int2**.

We have next considered the dimerization reactions starting from carbenes **Int2-Ap**_**anti**_, **Int2-Ap**_**syn**_, and **Int2-Eq**_**trans**_. For clarity, we will discuss the energies for the reaction at the **Int2-Ap**_**anti**_ position, while the relevant energies for the other positions are presented in the inserted table (Figure 3). The inner-sphere dimerization mechanism starts with a ligand exchange between acetonitrile and a second NHPI-DA molecule, which in the case of **Int2-Ap**_**anti**_ will preferably occupy the **Eq**_**cis**_ position. The resulting intermediate **Int3-Ap**_**anti**_**-DM** is calculated to be +0.8 kcal/mol relative to the carbene. The dimerization step then takes place *via* transition state **TS-Ap**_**anti**_**-DM**, with an associated barrier of 14.5 kcal/mol relative to the carbene, leading to the formation of the final product. In contrast to other systems,^7b,8b^ the outer-sphere dimerization mechanism in carbenes **Int2** derived from Ru-Pheox (**2**) led to much higher transition-state barriers (see the SI).

In addition to the dimerization reaction, we also found that the migratory insertion of the carbene into the Pheox ligand is also a viable option (see Figure 3). The migratory insertion of carbenes into aryl-metallacycles is well documented in other reactions,^23^ but has previously not been invoked in the catalysis of this complex. The calculated barrier (**TS1-Ap**_**anti**_**-MI**) is indeed very low, only 8.2 kcal/mol relative to the carbene, and it results in the formation of a new very stable ruthenium species **Int3-Ap**_**anti**_**-MI** that is 40.7 kcal/mol lower than the carbene. The barrier for migratory insertion (MI) starting from **Int2-Ap**_**anti**_ is thus 6.3 kcal/mol lower than the barrier for dimerization (DM). We have calculated these energy differences for all of the operative carbene complexes (ΔΔ*G*_MI-DM_^‡^, see Figure 3). A similar energy difference is found starting from **Int2-Ap**_**syn**_ (ΔΔ*G*_MI-DM_^‡^ = −6.4 kcal/mol), while starting from **Int2-Eq**_**trans**_, the situation is the opposite, *i.e.*, the barrier for the dimerization is lower than that for migratory insertion, by 12.2 kcal/mol (see Figure 3).

The results show thus that the migratory insertion is more favorable than the dimerization at the two apical positions (**Ap**_**syn**_, **Ap**_**anti**_), which would indicate that the dimerization will not take place, at least not starting at these positions. This result seems to contrast with the experimental observations presented above (Figure 1). However, it is possible that product of the insertion reaction, *i.e.*, **Int3-MI**, can itself function as a catalyst for the dimerization reaction. Indeed, we have considered this possibility, and the calculations show that it follows the same steps as the Ru-Pheox complex (**2**), and the calculated barriers are also very similar (see the SI). Furthermore, we also explored the possibility of the migratory insertion product acting as a catalyst for the cyclopropanation reaction, and we found that the reaction was associated with feasible energy barriers (see the SI). It is important to note that the migratory insertion results in the formation of a new stereogenic center at the ligand, and thus two different diastereomers of the insertion product can form. However, the migratory insertions at the two apical positions result in the formation of the same product **Int3-MI** (see the SI).

Whether or not the migratory insertion is a favored reaction in the presence of the olefin can have important implications on the mechanism and the selectivity of the reaction. The formation of a hypothetically dimerization-competent **Int3-MI** would be consistent with the possible induction period observed in the kinetic profiling of the dimerization reaction and the intermediate detected by HRMS (Figure 1). Given that the induction period could also be explained by de-coordination of an acetonitrile molecule prior to carbene formation (see the SI), and the intermediate on HRMS could correspond to **Int2** (see above), further studies were required.

### II.III Experimental Determination of the Active Catalyst in the Cyclopropanation Reaction

**Scheme 2:**
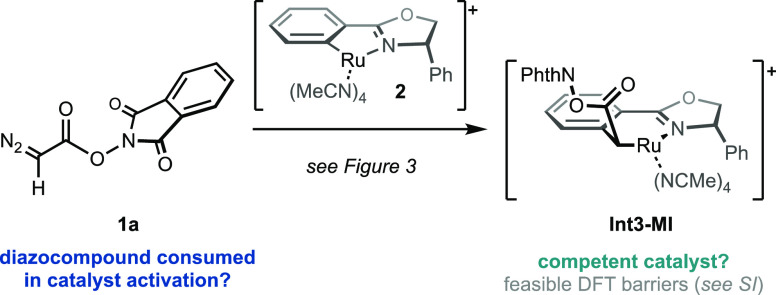
Possible Activation of the Catalyst Ru-Pheox *via* the Migratory Insertion Complex **Int3-MI**

We set out to assess the feasibility that cyclopropanation reactions are mediated by **Int3-MI** species, but all attempts to isolate the organometallic species generated during the reaction were unsuccessful. We recognized that the ruthenium migratory insertion complex **Int3-MI** incorporates the structure of the carbene into the ligand. This is reflected in the overall stoichiometry of the reaction on the diazocompound **1** and would influence the selectivity of the resulting modified catalyst **Int3-MI** (Scheme 2).

On the stoichiometry side, diazocompound **1** would be initially consumed without producing the cyclopropane product **3**, if migratory insertion is required to initiate the catalysis. Therefore, in this scenario, stoichiometric or superstoichiometric amounts of Ru-Pheox (**2**) would inhibit the formation of cyclopropane **3**. Different relative concentrations of NHPI-DA (**1a**) and Ru-Pheox (**2**) were explored to observe this effect (Table 1). Increasing the relative concentration of Ru-Pheox (**2**) from the standard 1 mol % (entry 1) up to 5 equiv. has no effect on the yield or enantioselectivity of the cyclopropane product **3a** (entries 2–4). These experiments demonstrate that migratory insertion complex **Int3-MI** is unlikely to be required for the cyclopropanation of the olefin. Importantly, the association of more than one diazocompound **1a** with the catalyst **2** prior to reaction with the olefin **4** can also be ruled out.

**Table 1:**
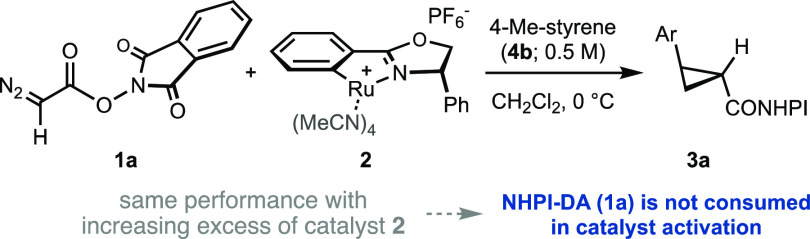
Study of Stoichiometry on the Cyclopropanation of *p*-Methylstyrene Using NHPI-DA (**1a**) and Ru-Pheox (**2**)^d^

entry	[**1a**]_0_ (M)	[**2**]_0_ (M)	[**2**]_0_/[**1a**]_0_	**3a** (%)^a^	e.r.^b^
1^c^	0.1	0.001	0.01	97	96:4
2	0.2	0.1	0.5	95	93:7
3	0.1	0.1	1	96	94:6
4	0.05	0.1	2	97	96:4
5	0.02	0.1	5	95	96:4

a Yields were measured by ^1^H NMR using 1,1,2,2-tetrachloroethane as an internal standard.

b Enantiomeric ratios (e.r.) were determined by high-performance liquid chromatography (HPLC).

c Standard conditions of the synthetic method.^5^

d The diazo reagent was added in one portion to a solution of olefin and Ru-Pheox. The product was obtained as a single diastereomer.

**Scheme 3:**
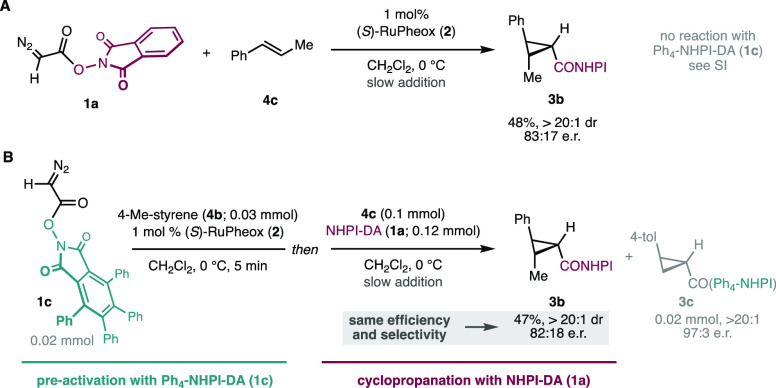
(A) Cyclopropanation of β-Methylstyrene Employing NHPI-DA under Standard Conditions; (B) Cyclopropanation Experiment Using Preactivation with Ph_4_-NHPI-DA Aimed to Observe the Effect of Potential **Int3-MI** Catalysts Yields were measured by ^1^H NMR using 1,1,2,2-tetrachloroethane as an internal standard.

However, it could still be possible that only a small amount of the migratory insertion complex **Int3-MI** would be exceedingly more active in the cyclopropanation reaction than the Ru-Pheox carbenes **Int2**. To exclude this possibility, we synthesized a new diazocompound reagent Ph_4_-NHPI-DA (**1c**) bearing a much larger redox-active ester substituent, to compare its efficiency and selectivity in cyclopropanation. We reasoned that upon migratory insertion, the much bulkier group in the corresponding intermediate (tetraphenyl analog of **Int3-MI**) would have a noticeable effect, particularly on enantioselectivity. We chose β-methylstyrene (**4c**) as a substrate due to its modest performance in the cyclopropanation reaction with NHPI-DA (**1a**) (Scheme 3A) and the lack of reactivity toward its tetraphenyl analogue Ph_4_-NHPI-DA (**1c**; see the SI). These features enable the detection of small changes in efficiency, diastereoselectivity, and enantioselectivity. Under standard conditions, NHPI-DA (**1a**) produces cyclopropane **3b** as a single diastereomer, in 48% yield and e.r. = 83:17 (Scheme 3A). As shown in Scheme 3B, the precatalyst Ru-Pheox (**2**) was activated with the bulkier Ph_4_-NHPI-DA (**1c**) and a good substrate like *p*-methylstyrene (**4b**). After complete consumption of the diazo reagent **1c** to yield cyclopropane **3c** quantitatively, β-methylstyrene (**4c**) was added to the mixture, followed by NHPI-DA (**1a**). Analysis of the cyclopropanation product **3b** showed that the same yield and the same enantiomeric ratio was obtained as in standard conditions (without initial pretreatment with Ph_4_-NHPI-DA; Scheme 3A). These results may alternatively be explained by a negligible effect of the Ph_4_-NHPI moiety in the migratory insertion complexes **Int3-MI**; nevertheless, the identical enantioselectivity observed is unlikely to be accidental. Even if these experiments are not completely conclusive, we have been unable to observe any sign that cyclopropanation catalysis occurs *via* migratory insertion complexes **Int3-MI**. Despite the fact that their formation seems viable in our DFT calculations presented above, and the current experiments cannot rule this possibility out completely, they deem it rather improbable using aromatic olefins.

### II.IV Kinetics of the Cyclopropanation Reaction

**Figure 4:**
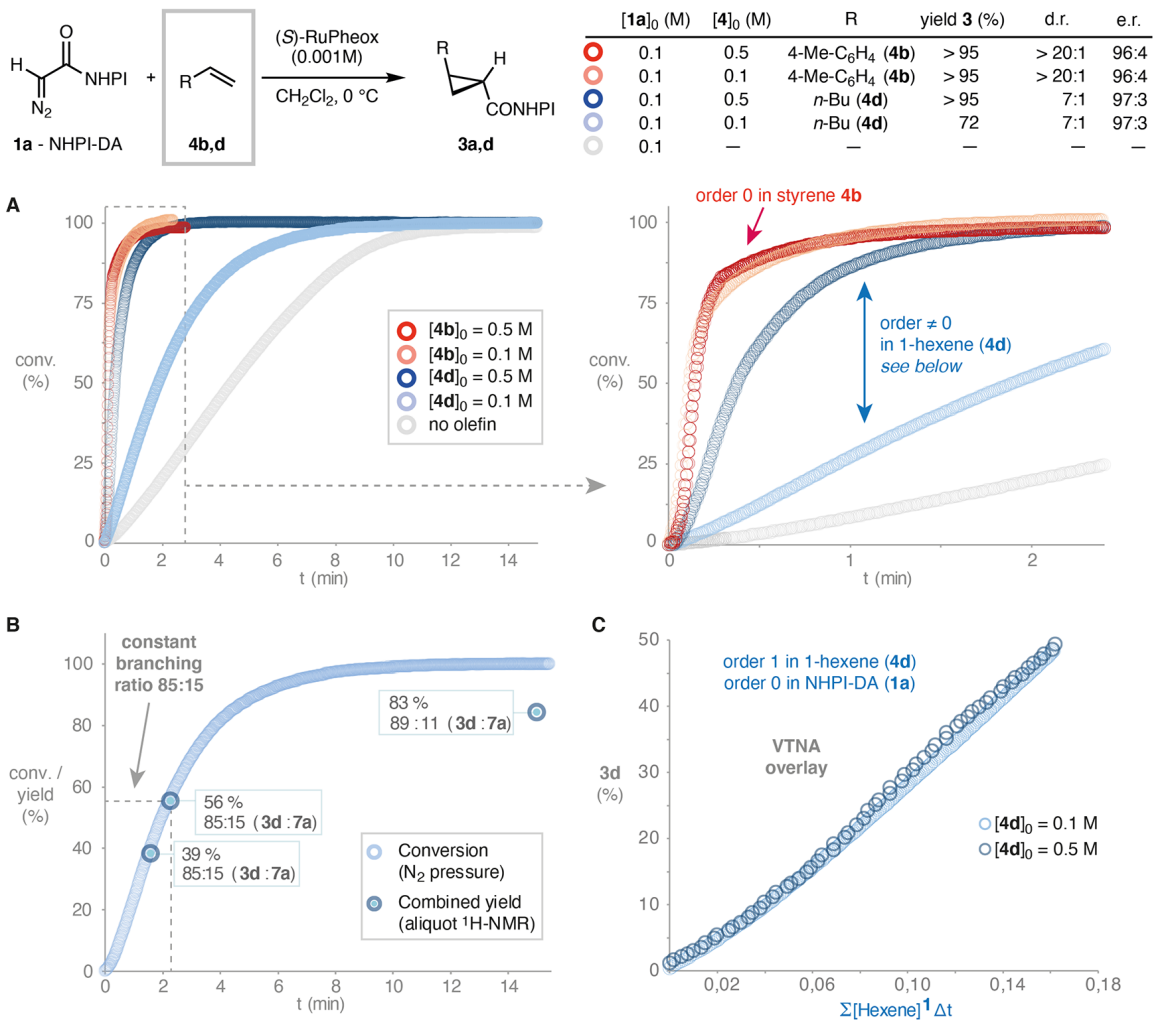
(A) N_2_ evolution profile (expansion on the right) of the cyclopropanation of 1-hexene and *p*-methylstyrene using NHPI-DA catalyzed by 1 mol % Ru-Pheox, in CH_2_Cl_2_ at 0 °C. Initial concentrations: [NHPI-DA]_0_ = 0.1 M, [**2**]_0_ = 0.001 M (top left). (B) Profile of the cyclopropanation of 1-hexene (**4d**) displaying a constant ratio (85:15) of cyclopropane and dimer products up to 56% conversion. (C) Variable time normalization analysis (VTNA) up to 56% conversion demonstrates first-order kinetics in 1-hexene (**4d**) and zero-order kinetics in NHPI-DA (linear range). The N_2_ released in the cyclopropanation was calculated using the constant selectivity factor in this regime (85:15; see (B)).

Next, the kinetics of cyclopropanation reactions using NHPI-DA (**1a**) catalyzed by Ru-Pheox (**2**) were investigated. Experimentally, two representative olefins with different electronic properties were chosen to study the system: 4-methylstyrene (**4b**) and 1-hexene (**4d**). It was found that in both cases, the cyclopropanation reactions were substantially faster than the corresponding dimerization process (Figure 4A). A brief activation period is observed, and is more evident when employing the less reactive substrate 1-hexene (**4d**). It is also evident that the rate of the reaction increases with higher concentrations of 1-hexene (**4d**), but it is unaffected when increasing the initial concentration of styrene **4b**.

The slower reaction with 1-hexene (**4d**) allowed it to be aliquoted and analyzed by ^1^H NMR at different conversions to determine cyclopropane and dimer concentrations. The match between the conversion determined by nitrogen evolution and the sum of the cyclopropane and dimer concentrations detected by ^1^H NMR demonstrated that at least up to 56% conversion, there are no other pathways consuming NHPI-DA (**1a**) to an appreciable extent. Moreover, it was noticed that the ratio between cyclopropane and dimer products (85:15) is completely stable in this period. This allowed us to estimate the nitrogen evolved in the cyclopropanation reaction from the total nitrogen pressure raw data (Figure 4B). Interestingly, variable time normalization analysis (VTNA)^24^ of the cyclopropanation kinetic profile revealed that the reaction is of first order in 1-hexene at least up to 56% conversion (Figure 4C), in line with the notion of the alkene substrate being involved in the rate-determining step (RDS) or any equilibria before the RDS. Moreover, the linearization observed in the VTNA plot after the initial activation period indicates that the reaction is of zero order with respect to the diazo compound in that conversion regime.^13f^ These reaction orders are consistent with a fast reaction of the diazocompound and the catalyst to form the putative carbene intermediate **Int2** followed by rate-determining cyclopropanation (either inner-sphere or outer-sphere). At high conversion, overlay is lost due to either catalyst deactivation or the alteration of the cyclopropanation/dimerization ratio toward the end of the reaction (see the SI). The apparent zero order observed in styrene **4b** is consistent with an intrinsically much faster cyclopropanation that is no longer rate-limiting.^25^ This explains why the cyclopropanation of styrene substrates with NHPI-DA did not require syringe-pump slow addition or excess, unlike with conventional diazocompounds.^4^ The concentration threshold for the olefin to operate under first-order (1-hexene; **4d**) and zero-order kinetics (styrene **4b**) correlates with the higher nucleophilicity of the latter (*N*{1-hexene} = −2.77; *N*{styrene} = +0.78).^26^ Thus, the previously empirical relationship between alkene nucleophilicity and cyclopropane yield is demonstrated to stem from a kinetic branching ratio of competing cyclopropanation and dimerization processes from a common intermediate (likely the metal carbene **Int2**).

In the case of the rate-limiting cyclopropanation of 1-hexene (**4d**), these results indicated that accumulation of the carbene intermediate **Int2** would occur, thus enabling an opportunity for its detection. *In situ* HRMS analysis of the reaction mixture resulted in the detection of the 527 *m*/*z* signal (see Figure 1), which faded away when the reaction was complete. In the cyclopropanation of *p*-methylstyrene, this intermediate was not detected, probably because **Int2** is not accumulated in the faster cyclopropanation cycle. The fact that this intermediate is only detected during rate-limiting cyclopropanation (1-hexene) or dimerization catalysis seems most consistent with it being the active species **Int2**. The possibility that this signal originates from the transient generation of **Int3-MI** cannot be completely ruled out. No olefin-bound complexes were detected by HRMS in any case, in agreement with the weak olefin binding predicted by the calculations (see Section II.V) and the corresponding NMR measurements on olefin–catalyst mixtures (see Section II.II).

### II.V Calculations of the Cyclopropanation Reaction

Next, we calculated the different possible mechanisms for the cyclopropanation reaction using propene (**4e**) and styrene (**4a**) as representative cases of aliphatic and aromatic olefins, respectively. Earlier work has established that propene (**4e**) and 1-hexene (**4d**) display similar performance and selectivity in this cyclopropanation,^5^ and the use of propene simplifies the computational treatment of the aliphatic chain of the olefin.

**Figure 5:**
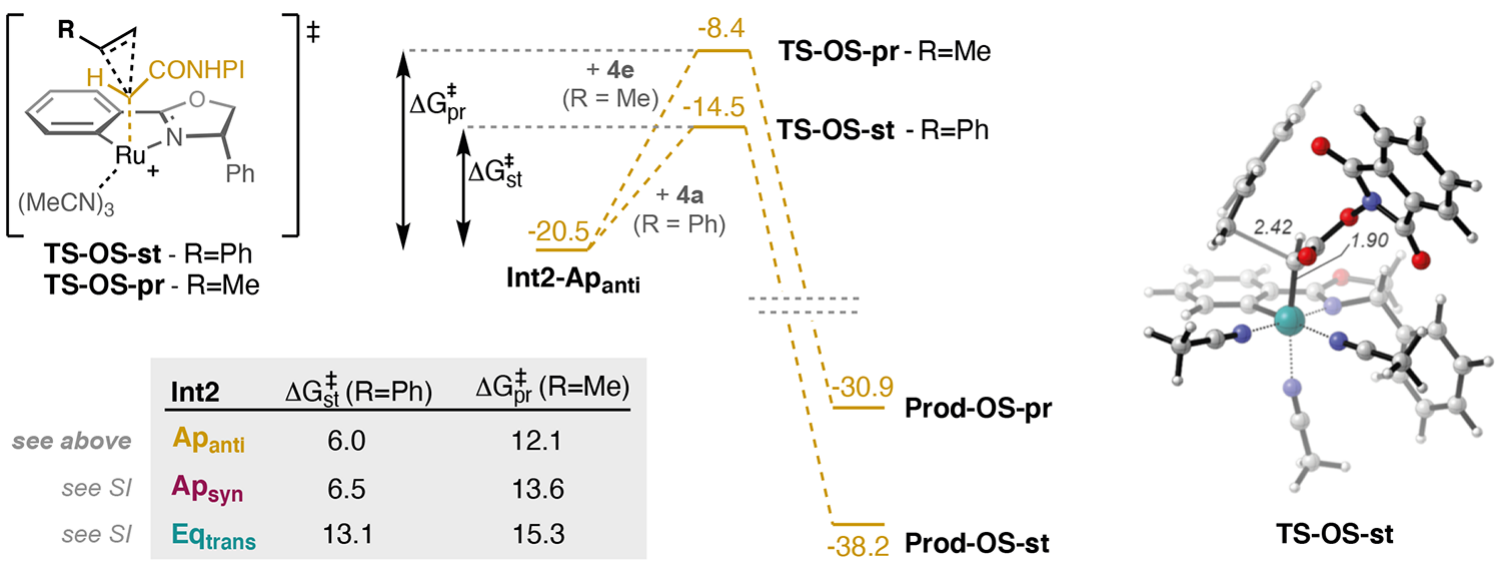
Calculated free energy profiles for the outer-sphere (OS) mechanism starting from the **Ap**_**anti**_ position using styrene and propene as substrates. The inserted table shows the calculated barriers relative to the viable **Int2** carbenes.

**Figure 6:**
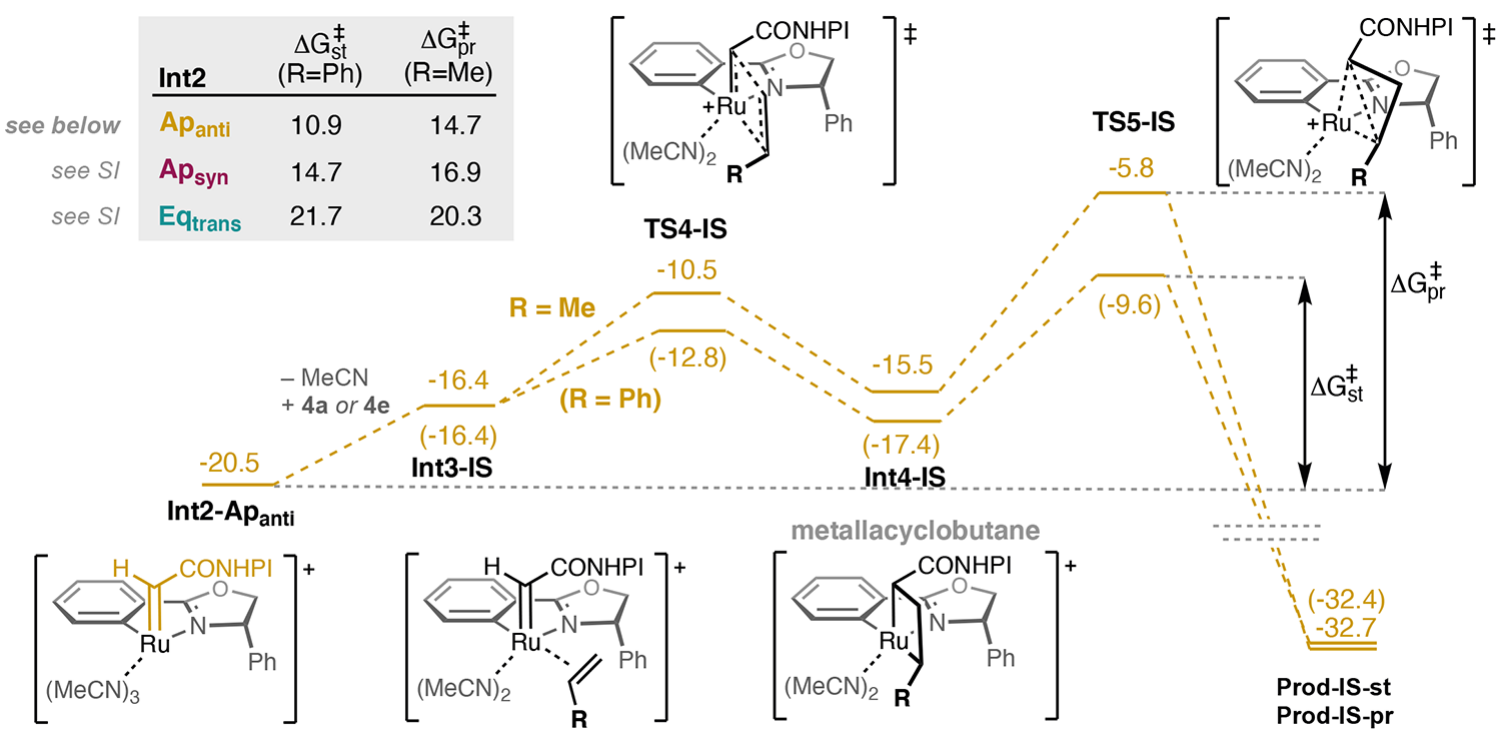
Calculated free energy profile for the inner-sphere (IS) mechanism at the **Ap**_**anti**_ position using styrene and propene as substrates. The inserted table shows the calculated barriers for the reductive elimination step relative to **Int2** at the different positions of the catalyst.

First, starting from the Ru-Pheox complex, we calculated the energies of a number of complexes in which one or several acetonitrile ligands were exchanged for a propene molecule. These complexes were in all cases calculated to be higher in energy than the starting Ru-Pheox complex **2** (see the SI). The energy barriers for the carbene formation with the olefin bound to the metal were also calculated to be higher in energy than the ones presented above for the reaction starting from Ru-Pheox (**2**, Figure 2). The study of the cyclopropanation reaction can therefore start from carbene intermediate **Int2**. Both the inner-sphere (IS) and outer-sphere (OS) cyclopropanation mechanisms were considered, as both would be consistent with the olefin reaction orders detected experimentally (see Section II.IV). Again, Figures 5 and 6 display the energy profiles of the reaction starting from **Int2-Ap**_**anti**_ position, and the energies starting from the other **Int2** isomers are summarized.

The outer-sphere mechanism entails a direct insertion of the carbene into the olefin molecule in the second shell. Figure 5 shows the calculated free energy profile for this mechanism starting from the **Ap**_**anti**_ position, which is associated with a barrier (**TS-OS**) of 12.1 kcal/mol for propene (**4e**) and 6.0 kcal/mol for styrene (**4a**). In the case of the outer-sphere mechanism, the styrene has a lower barrier than the propene for all carbenes **Int2**, as shown in Figure 5. This trend is consistent with the higher nucleophilicity of the aromatic olefin (see Section II.IV).

The alternative inner-sphere mechanism consists of a ligand exchange between the olefin and an acetonitrile at the **Eq**_**cis**_ position, followed by a formal [2 + 2] cycloaddition step (**TS4-IS**) to form the metallacyclobutane intermediate **Int4-IS**, and a subsequent reductive elimination through **TS5-IS** to form the final product. Importantly, the barrier for the reductive elimination step is higher than the cycloaddition step regardless of the olefin and the carbene isomer considered, and as seen from the calculated free energy profiles in Figure 6, the inner-sphere mechanism is always associated with higher barriers than the outer-sphere mechanism. We also considered the possibility that the **Int4-IS** intermediate undergoes a retro-[2 + 2] cycloaddition step to form a new carbene derived from styrene, but the associated barrier was considerably higher than the subsequent **TS5-IS** (see the SI). Finally, we also evaluated if coordination of a ligand other than acetonitrile could result in lower barriers for the cyclopropanation reaction. To this end, we compared the energies of the ligand exchange between acetonitrile and the other ligands (dichloromethane, propene, and diazoacetate **1a**) in the case of the initial Ru-Pheox (**2**) and **Int2-Ap**_**anti**_ carbene. We considered the **Eq**_**cis**_ position because it is the one that presents the more favorable ligand exchange energies among the four available positions in the initial complex **2**. The obtained energies are in all cases endothermic (see the SI). Therefore, the possibility that a ligand exchange would result in more stable TSs is unlikely.

As a final note, the formation of carbenes **Int2** at the **Ap**_**anti**_, **Ap**_**syn**_, and **Eq**_**trans**_ positions is calculated to be rate-determining in the case of styrene (**4a**). This result is in good agreement with the experimental evidences presented in Section II.IV, which shows that the rate for the cyclopropanation of the aromatic olefin **4b** does not depend on its concentration (zero-order). For propene (**4e**), the barriers for the carbene formation and the subsequent cyclopropanation are calculated to be close in energy, indicating that either of the two steps could be the RDS. This is also in agreement with the experiments, showing that the reaction rate for the cyclopropanation of 1-hexene (**4d**) is dependent on its concentration.

### II.VI Analysis of the Selectivity

The calculations so far indicate that several reactions/pathways are possible in this system. Carbene formation reactions at the **Ap**_**anti**_, **Ap**_**syn**_, and **Eq**_**trans**_ positions are feasible and have similar barriers (within 2 kcal/mol), while it is unfavored at **Eq**_**cis**_, and this can be disregarded in the discussion. Each of these carbenes **Int2** could in principle react with olefins **4***via* inner-sphere or outer-sphere mechanisms to yield cyclopropanes **3**. Alternatively, the carbenes **Int2** can react with a second NHPI-DA (**1a**) molecule to form the dimer side product or undergo a migratory insertion reaction to form a new ruthenium complex **Int3-MI** that can also catalyze both the cyclopropanation and dimerization reactions. It is instructive here to compare the energies of the various scenarios and analyze their consequences in terms of reaction outcome and how well it fits with the experimental observations. In Table 2, the barriers for each of the four different reactions that can take place at the carbene intermediate are compared.

**Table 2:** Comparison of the Calculated Barriers (kcal/mol) of the Various Reactions That Can Take Place at the Carbene Intermediate **Int2**^a^

		cyclopropanation		dimerization	migratory insertion
olefin	**Int2**	**TS-OS**	**TS5-IS**	**TS-DM**	**TS-MI**
styrene (**4a**)	**Ap**_**anti**_	**0.0**	+4.9	+8.5	+2.2
	**Ap**_**syn**_	**0.0**	+8.2	+8.3	+1.9
	**Eq**_**trans**_	**0.0**	+8.6	+7.3	+18.7
propene (**4e**)	**Ap**_**anti**_	+3.9	+4.1	+6.3	**0.0**
	**Ap**_**syn**_	+5.2	+8.2	+6.4	**0.0**
	**Eq**_**trans**_	**0.0**	+5.0	+5.1	+16.5

a For each carbene, the lowest barrier among the four scenarios is set to zero (shown in bold).

For the reaction of the styrene (**4a**) substrate, the outer-sphere cyclopropanation mechanism is calculated to have the lowest barrier among the four possible reaction scenarios for all three carbene positions. This is consistent with the experimental outcome for this substrate, showing that no dimerization product is formed and that almost full conversion of the diazoacetate into the cyclopropane product is achieved. In the case of propene (**4e**), the outer-sphere cyclopropanation is preferred over the dimerization by a lower margin than with styrene, in accordance with the experiments on 1-hexene (**4d**). However, the migratory insertion is calculated to be the most favored pathway for the apical carbenes in this case.

**Figure 7:**
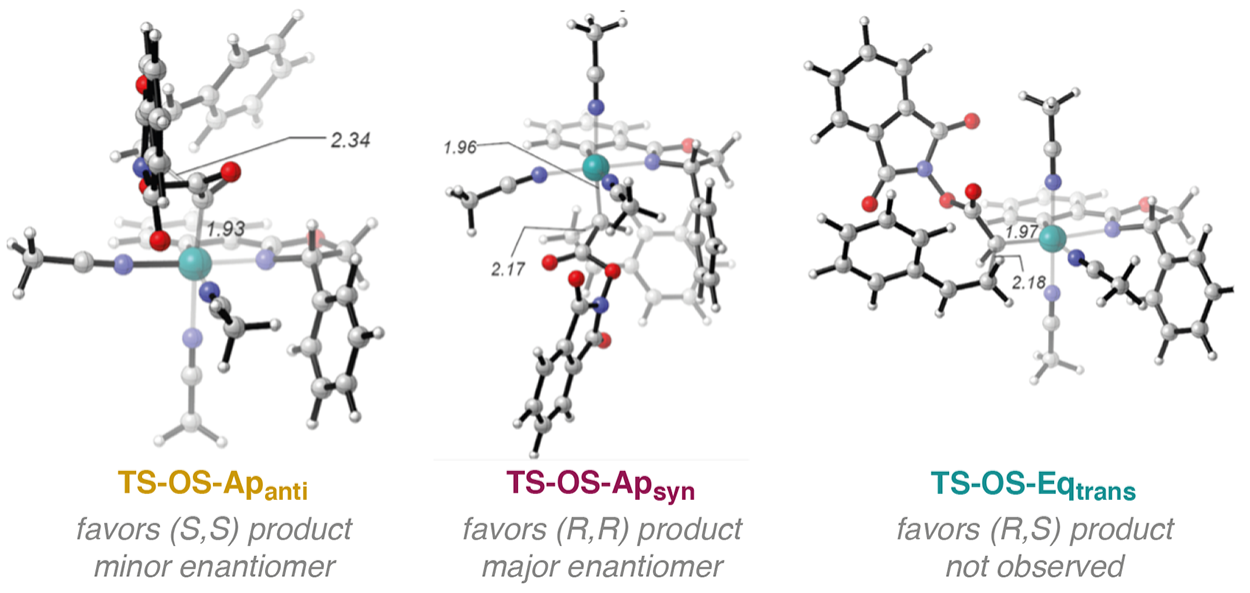
Optimized geometries of the lowest-energy outer-sphere (OS) transition states leading to the various diastereomers of the cyclopropane product starting from the **Ap**_**anti**_, **Ap**_**syn**_, and **Eq**_**trans**_ carbene intermediates.

Since all three carbene positions (**Ap**_**anti**_, **Ap**_**syn**_, and **Eq**_**trans**_) yield the cyclopropane product and the three intermediates have different environments around the carbene, one way to single out which one of them is the dominant one could be to compare their stereochemical outcomes to the experiments. The cyclopropanation reaction has been experimentally shown to favor the formation of the *trans* products with (*R*,*R*) absolute configuration when using (*S*)-Ru-Pheox.^5^ We optimized all possible transition states leading to the different diastereomers in the case of styrene for the three carbene intermediates **Int2**, considering both the outer-sphere (**TS-OS**) and inner-sphere (**TS5-IS**) mechanisms. The energies are listed in Table 3, and the geometries of the most stable TSs starting from each of the three positions are displayed in Figure 7 (geometries of the other TSs are given in the SI). For all of the three carbene positions, the inner-sphere transition states are much higher than the outer-sphere (by 5–8 kcal/mol), and can therefore be left out in the following discussion of the enantio- and diastereoselectivity.

**Table 3:** Energies (kcal/mol) of the Possible Transition States in the Cyclopropanation of Styrene Leading to the Different Stereoisomers of the Product for the Three Carbene Intermediates **Int2**, Considering Both the Outer-Sphere (**TS-OS**) and Inner-Sphere (**TS5-IS**) Mechanisms^a^

	outer-sphere				inner-sphere			
**Int2**	*R*,*R*	*S*,*S*	*R*,*S*	*S*,*R*	*R*,*R*	*S*,*S*	*R*,*S*	*S*,*R*
**Ap**_**anti**_	+3.9	**0.0**	+5.9	+5.1	+6.0	+9.3	+11.9	+4.9
**Ap**_**syn**_	**0.0**	+1.8	+3.0	+4.2	+11.5	+10.7	+8.2	–
**Eq**_**trans**_	+1.1	+0.8	+5.1	**0.0**	+8.6	+8.9	+11.1	+11.5

a For each carbene, the lowest barrier among the eight possible scenarios is set to zero (shown in bold).

Starting from **Int2-Ap**_**anti**_, which has the lowest barrier of carbene formation (Figure 2), the calculations predict that the (*S*,*S*)-product to be formed. The barriers leading to the other products are *ca*. 4–6 kcal/mol higher in energy. This is in contrast to the experimental outcome, showing (*R*,*R*) as the dominant product. On the other hand, starting from the other apical carbene **Int2-Ap**_**syn**_, whose formation is within 1 kcal/mol of **Int2-Ap**_**anti**_ (Figure 2), the (*R*,*R*)-product is correctly reproduced, and the barriers leading to the other products are *ca*. 2–4 kcal/mol higher. Finally, starting from the **Eq**_**trans**_ carbene, the calculations predict that the (*S*,*R*) product to be dominant, with the barriers for (*S*,*S*) and (*R*,*R*) being only *ca*. 1 kcal/mol higher in energy (Table 3).

The stereochemistry calculations indicate thus that it is possibly the carbene **Int2-Ap**_**syn**_ that is operational in the reaction, since it is the only one reproducing the correct stereochemistry of the product in the case of the styrene substrate. Nevertheless, on the reaction of the propene substrate, the migratory insertion of the **Ap**_**syn**_ carbene is favored by 5.2 kcal/mol (Table 2) over the outer-sphere cyclopropanation pathway. This indicates that, in this case, the migratory insertion product **Int3-MI** could be involved in the catalysis or deactivation of the system (see the SI). Although we have not found any experimental evidence of this pathway being operational in the cyclopropanation aromatic olefins (see Section II.III), our experiments do not rule out this possibility for aliphatic olefins or other diazocompounds, and this mechanism should be considered further in related carbene-transfer reactions with metallacyclic catalysts.

The alternative scenario is that the operational carbene is **Int2-Eq**_**trans**_. In this carbene, the cyclopropanation reaction is associated with the lowest barriers for both the aromatic and aliphatic olefins (Table 2). However, the favored cyclopropane product according to the calculations does not qualitatively reproduce the stereochemical outcome of the reaction as the nondetected diastereoisomer is predicted (Table 3).

**Figure 8:**
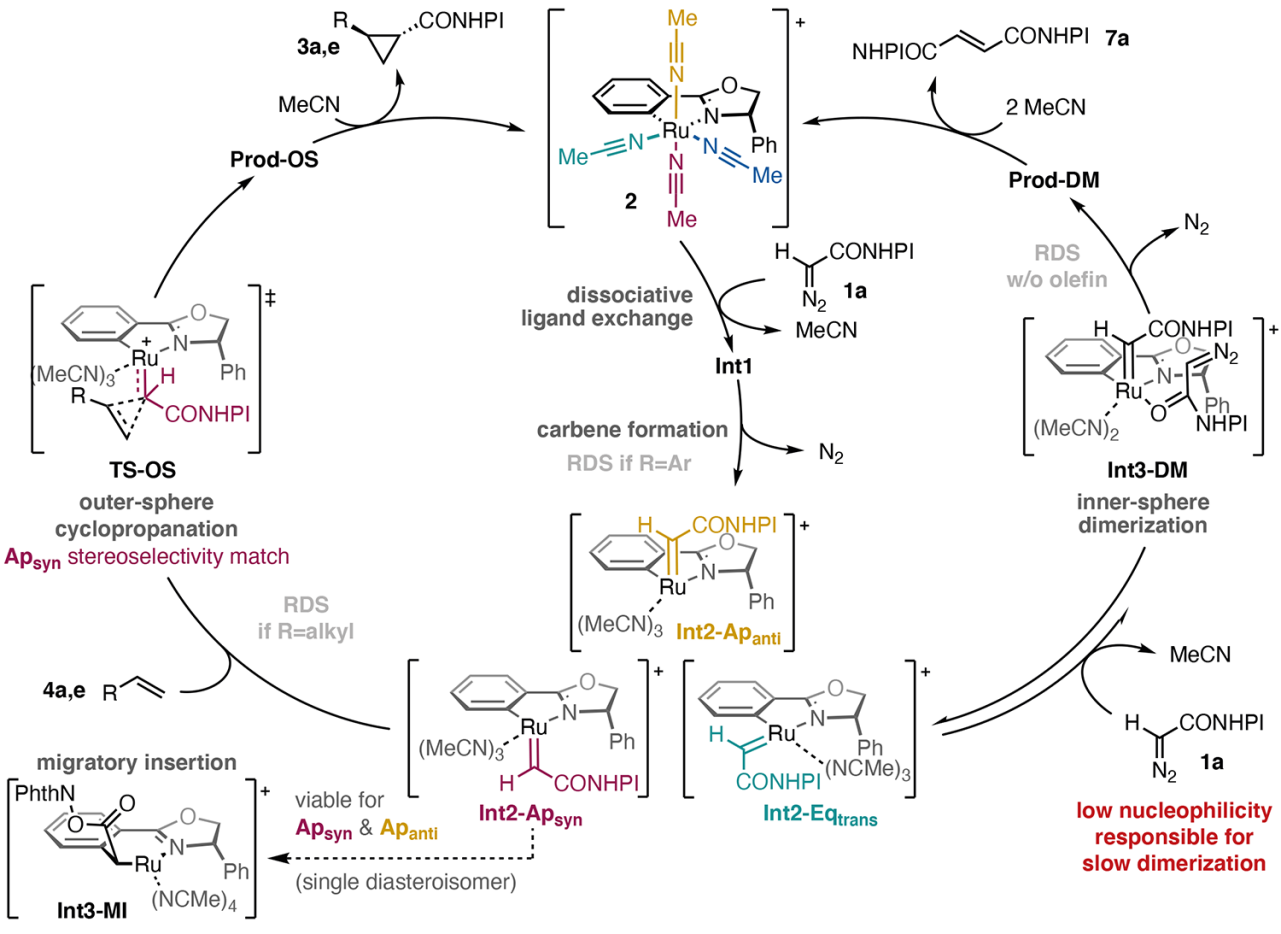
Current mechanistic model consistent with calculations and experiments.

To conclude this section, Ru-Pheox complex **2** reacts with NHPI-DA (**1a**) through a dissociative mechanism leading to three viable carbene complexes **Int2-Ap**_**anti**_, **Ap**_**syn**_, and **Eq**_**trans**_ (Figure 8). These complexes generally favor an outer-sphere mechanism in the cyclopropanation of olefins (Tables 2 and 3), among which the carbene **Int2-Ap**_**anti**_ is most consistent with the diastereo- and enantioselectivity observed in the experiments (Table 3).^5^ The apical carbenes **Int2-Ap**_**anti**_ and **Ap**_**syn**_ can undergo migratory insertion to yield a new organometallic catalyst **Int3-MI**, whose role in the catalysis or the deactivation of the catalyst is yet unclear. Alternatively, the carbenes **Int2** can engage NHPI-DA (**1a**) in a dimerization process through an inner-sphere mechanism, which has generally higher barriers than outer-sphere cyclopropanation. Nevertheless, the interplay between all of these options is quite delicate, in many cases involving small energy differences, and is difficult to reconcile only one of the carbenes **Int2** with all experimental facts in both olefin families.

## III Conclusions

In the present study, we have used a combined experimental–computational approach to investigate the mechanism of the cyclopropanation reaction of a redox-active diazocompound NHPI-DA with aromatic and aliphatic olefins catalyzed by the metallacyclic Ru-Pheox complex.

Kinetic experiments demonstrate that the enhanced reactivity displayed by NHPI-DA principally stems from its unusually low nucleophilicity. This results in a slower dimerization side reaction that allows challenging aliphatic olefins to compete effectively for a common metal carbene intermediate. The kinetic branching ratio from this intermediate has been proven to define the cyclopropanation/dimerization selectivity of the reaction. This paradigm explains the strong correlation between the relative nucleophilicity parameters of the olefin and the diazocompound with the selectivities that are observed. This model explains why the cyclopropanation of aromatic olefins can operate with NHPI-DA optimally without large excess of the reagent nor slow addition, and it is consistent with the kinetic orders determined and the in situ HRMS measurements reported herein.

Among various possibilities, DFT calculations reveal that the Ru-Pheox catalyst can form three different stereoisomeric carbenes with very similar barriers. The reactions of all of these possible stereoisomers with the diazocompound (dimerization), the olefin (cyclopropanation), and a new intramolecular migratory insertion of the ligand have been thoroughly evaluated. These studies confirmed that the dimerization is hindered by higher kinetic barriers than the cyclopropanation of the olefin. The migratory insertion process is kinetically viable and leads to a complex that could operate as a catalyst. Control experiments do not favor this extreme in the cyclopropanation of styrenes with redox-active carbenes, but this possibility should be considered in future mechanistic analyses of other carbene-transfer reactions using metallacyclic catalysts.

Comparison of the two possible cyclopropanation mechanisms shows that the reaction takes place preferably *via* an outer-sphere mechanism. Analysis of the stereochemical outcome reveals that the barriers of both the carbene formation and the outer-sphere cyclopropanation contribute to the observed selectivity of the reaction. The small energy differences obtained in many instances indicate that small alterations in the diazocompound, the olefin substrate, or the ligand employed might cause major changes to the selectivity.

These results indicate that future development of less nucleophilic diazocompound reagents could enhance cyclopropanation/dimerization selectivity in challenging systems. Techniques such as VTNA, benzhydrylium benchmarking, HRMS, and DFT calculations have been combined for the first time to gain mechanistic understanding in cyclopropanation catalysis. These tools pave the way for further developments in this field driven by mechanistic understanding.

## IV Computational Details

The geometries of all species were optimized with the B3LYP-D3(BJ) functional^27^ as implemented in the Gaussian 16 package.^28^ The LANL2DZ pseudopotential^29^ was used for Ru and the 6-31G(d,p) basis set for the other atoms. Thermochemical and solvation corrections (calculated with dichloromethane as solvent and the polarizable continuum model (PCM) method^30^) were added as single points at the same level of theory as the geometry optimization. The final electronic energies were calculated as a single-point at the LANL2TZ/6-311+G(2d,2p) level. Standard state corrections were added to account for the conversion from the 1 atm ideal gas to the 1 M standard state of the solutes. Thus, the correction term *RT* ln(24.5) = +1.9 kcal/mol was added to the energies of all complexes, except for dinitrogen, which is in the gaseous state.

## Supporting Information Available

Experimental procedures, kinetic monitoring, and data analysis (PDF)
Further computational results, absolute energies and energy corrections, and Cartesian coordinates of reported structures (PDF)
