# Supplementary material for: Combined Experimental and Computational Study of Ruthenium N-Hydroxyphthalimidoyl Carbenes in Alkene Cyclopropanation Reactions
Source: ACS Catal. 2021 Aug 18;11(17):10950–63. doi: 10.1021/acscatal.1c02540 (PMC8419840; doi:10.1021/acscatal.1c02540)
Supplement: Supplementary file 1 — cs1c02540_si_001.pdf [file cs1c02540_si_001.pdf]

Supporting information
Experimental part

Combined Experimental and Computational Study of Ruthenium *N*-Hydroxyphthalimidoyl Carbenes in Alkene Cyclopropanation Reactions

Ferran Planas[†], *Matteo Costantini*[†], *Marc Montesinos-Magraner, Fahmi Himo*, Abraham Mendoza**

Department of Organic Chemistry, Arrhenius Laboratory, Stockholm University, SE-106 91 Stockholm, Sweden.
[†]*The authors contributed equally to the work.*

Corresponding authors email: fahmi.himo@su.se; abraham.mendoza@su.se

Contents

1. General experimental S1
2. Kinetic Profiling Details S2
3. Synthesis of starting materials S5

Preparation of Ph_4NHPI-DA (**1c**): S5

4. Kinetic measurement – Dimerization reaction S6

Dimerization of ethyl diazoacetate (EDA) **2a**: S6

Dimerization of N-hydroxyphthalimidoyl diazoacetate (NHPI-DA) **1a**: S6

5. Determination of the Nucleophilicity Parameter (*N*) for NHPI-DA (1a) S8

General Experimental Details: S8

Kinetics and determination of N: S8

Procedure for kinetic measurements using (mfa)2$CH^+BF_4^-$(**8**) S9

Recording of the absorbance vs time for NHPI-DA (**1a**) and $(mfa)_2CH^+BF_4^-$(**8**) S10

Preliminary estimation of the nucleophilicity (N) of NHPI-DA (**1a**) S12

Validation of the experimental set-up: nucleophilicity (N) of ethyl diazoacetate (**1b**) S12

Qualitative nucleophilicity comparison between EDA (**1b**) and NHPI-DA (**1a**) vs. a reference benzhydrylium electrophile **8**. S13

6. Kinetic measurement – Cyclopropanation of 1-hexene (4d) S15

Determination of reaction order in olefin S16

7. Kinetic measurement – Cyclopropanation of 4-methylstyrene (4b) S19
8. Stoichiometry experiments S20
9. Sequential experiments with Ph_4-NHPI-DA (1c) S21

Synthesis of 1,3-dioxoisoindolin-2-yl (1R,2R,3R)-2-methyl-3-phenylcyclopropane-1-carboxylate **3b**: S21

Synthesis of 1,3-dioxo-4,5,6,7-tetraphenylisoindolin-2-yl (1R,2R)-2-(p-tolyl)cyclopropane-1-carboxylate **3c**: S21

Sequential experiment with different diazoreagents: S22

10. NMR spectra of synthesized compounds S23

^{1}H NMR (400 MHz, CH_2Cl_2) for (S)-RuPheox$(CH_3CN)_4$ S23

^{1}H NMR (400 MHz, $CDCl_3$) for Ph_4-NHPI-DA (**1c**) S24

^{13}C NMR (400 MHz, $CDCl_3$) for Ph_4-NHPI-DA (**1c**) S25

^{1}H NMR (400 MHz, $CDCl_3$) for compound (**3b**) S25

^{13}C NMR (400 MHz, $CDCl_3$) for compound (**3b**) S27

^{1}H NMR (400 MHz, $CDCl_3$) for compound (**3c**) S28

^{13}C NMR (400 MHz, $CDCl_3$) for compound (**3c**) S29

11. Chromatography data of enantioenriched compounds S30

11.a HPLC traces for the stoichiometric experiment S30

HPLC trace for compound **rac-3a** S30

HPLC traces for compound **3a** (entry 2) S30

HPLC traces for compound **3a** (entry 3) S31

HPLC traces for compound **3a** (entry 4) S31
HPLC traces for compound **3a** (entry 5) S32
11.b HPLC traces for compound **3b**.. S33
11.c HPLC traces for compound **3c** S35
11.d HPLC traces for compound **3b** from the sequential reaction.. S36
12. References S38

1. General experimental

Materials. All reactions were carried out using oven-dried glassware under an atmosphere of argon or nitrogen. Dry solvents were obtained using a solvent purifier equipped with activated alumina columns. All solvents used in work-up procedures, silica gel column cromatography, HPLC analysis and/or purification were obtained from commercial suppliers and used without further purification. When appropriate, degassing of anhydrous solvent was achieved through three freeze-pump-thaw cycles or by bubbling Ar for 30 minutes under sonication. Reactions were performed in common pyrex round bottom flasks, microwave vials 2 - 8 ml (VWR or Biotage®), or 5 - 20 ml flat bottom vials (Cronus, SMI-LabHut Ltd. or VWR®) crimped on top with 20 mm Sil/PTFE Septa. Reaction temperatures were maintained using water-ice baths. Slow additions were performed using Landgraf HLL LA-30 or Harvard Apparatus Pump11Elite syringe pumps.

Kinetic measurements. All experimental kinetic measurements were performed using the *Man on the Moon X103* kit according to the specifications by the manufacturer (for more specifics on the measurements, see Kinetic Profiling Details below).

Reagents were obtained from commercially available sources and used as received unless stated otherwise. NHPI-DA was prepared as previously reported by Mendoza and co-workers.[1] Catalyst (*S*)-Ru-Pheox(CH_3CN)$_4$$PF_6$, was prepared as described by Iwasa.[2] Commercially available alkenes were purchased from Sigma-Aldrich, Fluorochem or Acros Organics. Liquid alkenes were quickly distilled, using a Hickman head, prior to use.

Chromatography. Thin layer chromatography (TLC) was carried out on 0.25 mm E. Merck silica plates (60F-254), using UV light as visualizing agent and a vanillin or $KMnO_4$ solution and heat as developing agent. Flash silica gel chromatography was performed using Normasil 60 VWR Chemicals (60 Å, particle size 0.040-0.063 mm) or E. Merck silica gel (60 Å, particle size 0.043-0.063 mm).

Characterization. NMR spectra for the characterization of compounds were recorded –unless stated otherwise– at 25˚C on Bruker 400 Ultrashield (400/101 MHz $^1H/^{13}C$) or Bruker 500 Ultrashield (500/126 MHz $^1H/^{13}C$) instruments. Chemical shifts (δ) are reported in ppm, using the residual solvent peak in $CDCl_3$ (δH = 7.26 and δC = 77.16 ppm) or CD_2Cl_2 (δH = 5.32 and δC = 53.84 ppm) as internal reference, and coupling constants (*J*) are given in hertz (Hz). Data are reported as follows: chemical shift, multiplicity (s: singlet, d: doublet, t: triplet, q: quartet, hex: hexet; br: broad, m: multiplet), coupling constants (*J* in Hz) and integration. Carbon multiplicities were assigned by DEPT and edited HSQC techniques. NMR yields were measured using 1,1,2,2-tetrachloroethane as an internal standard.

High-resolution mass spectra (HRMS) were determined with a Bruker Daltonics microTOF Mass Spectrometer equipped with an electrospray (ESI) ion source.
For the *in-situ* analysis of dimerization and cyclopropanation reactions, the following settings were used: Source capillary, 137 nA; Source nebulizer, 0.4 bar; Capillary exit, 150.0 V; Skimmer 1, 50 V; Lens 1 transfer, 64.0 µs. For comparison, the standard parameters are reported: Source capillary, 88 nA; Source nebulizer, 0.3 bar; Capillary exit, 90.0 V; Skimmer 1, 20.0 V; Lens 1 transfer, 52.0 µs.

Determination of Enantiomeric Purity. HPLC analysis on chiral stationary phase was performed on an Agilent 1200-series instrument, employing Daicel Chiralpak columns IA, IC and IF, or a Waters e2695 separation module. The exact conditions for the analyses are specified within the characterization section. HPLC traces were compared to racemic samples prepared using the achiral Ru-dmPheox(CH_3CN)$_4$$PF_6$ as the catalyst.[2]

2. Kinetic Profiling Details

Crystalline NHPI-DA **1a** was used and EDA **1b** concentration was checked by ^{1}H-NMR (usually around 85% Wt, in CH_2Cl_2, SigmaAldrich) before use. Typically, a 0.15-0.21 M solution of the corresponding diazocompound in dry CH_2Cl_2 was prepared in a volumetric flask under argon. Alkenes were distilled using a Hickman head and a stock solution in dry CH_2Cl_2 was prepared under argon. A stock solution of (*S*)-Ru-Pheox (0.015 M) was prepared in the glovebox, and taken out just before the experiments to avoid decomposition of the catalyst (Note: we have observed that solutions of the catalyst change color yellow to green when stored for 1-2 hour under a positive pressure of argon; the resulting green solutions lead to lower and irreproducible activity).

A dried 12 mL "Man on the Moon" reactor was backfilled with argon (Note: the space between the valve and the sensor was also purged using the 3-way valve). The corresponding diazocompound (0.225 mmol) and alkene were charged into the reactor and the volume of the mixture was adjusted to 2.1 mL with dry CH_2Cl_2 (this will give a final concentration of diazocompound of 0.1 M, after the addition of the catalyst solution). The pressure of the reaction after the addition has been calculated to be around 0.5 bar relative to the initial pressure inside the vessel. The mixture is then placed in an ice-bath and stirred for 2 minutes. Then the valve is changed from the argon flow to the detector and the measurement is started. The system is allowed to stabilize, until a constant pressure reading is achieved (approximately 5-8 min). The solution of catalyst is then injected in the reaction mixture and the syringe is carefully removed from the setup.

A typical data set looks as shown in **Figure S1**:

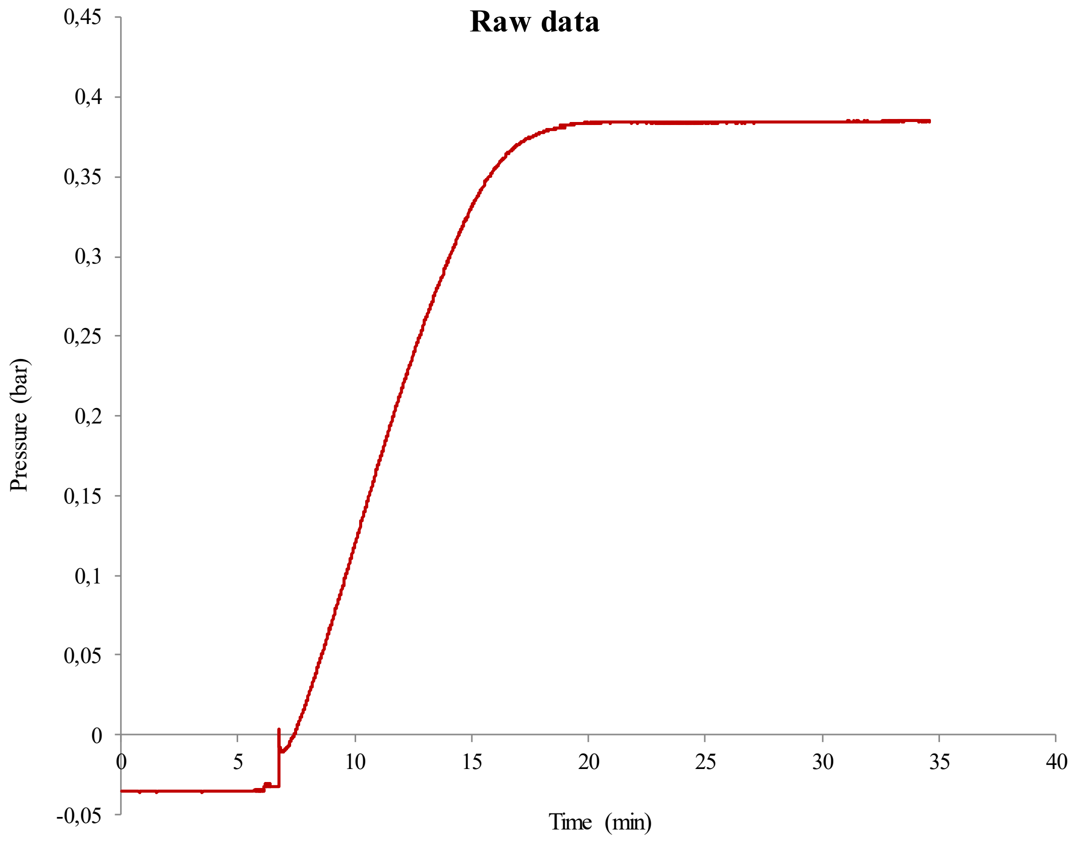

Figure S1: An example of raw pressure data set.

Note: The process of the injection of the catalyst through the septum also causes a variation in the pressure, but it is usually minor compare to the variation caused by the N_2 evolution. However, in some cases like in the example depicted above, this effect is more important. For a better comparison between experiments, the raw data is normalized from the first point after the injection spike (t=0) . In **Figure S1**, the first data point (for both time and pressure), was established after 7.00 minutes. The raw data is then transformed into the following normalized plot (**Figure S2**):

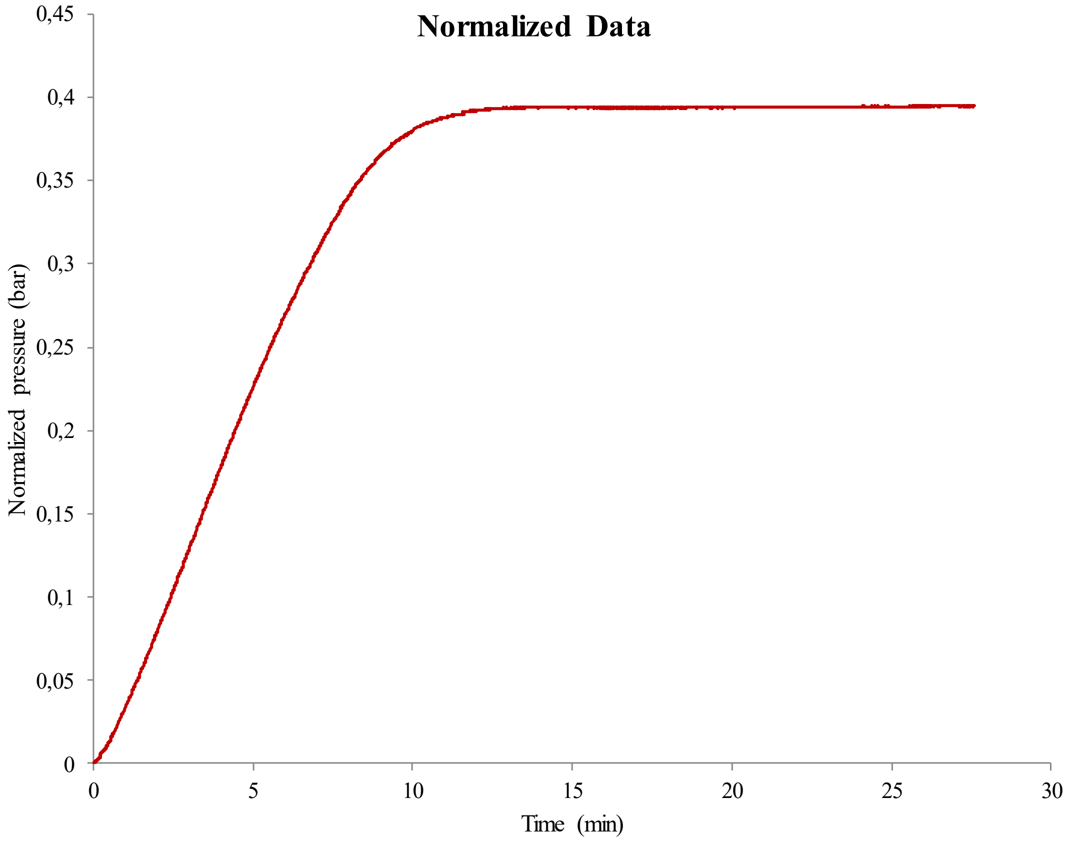

Figure S2: A pressure data set after normalization.

As a control, the dimerization reaction has been used as a control experiment between reaction sets. In **Figure S3**, three different runs are shown. The red and blue profiles show how the robustness of the method. In contrast, the grey profile shows a slightly slower reaction and lower final pressure, most likely due to a wrong placing of the septum.

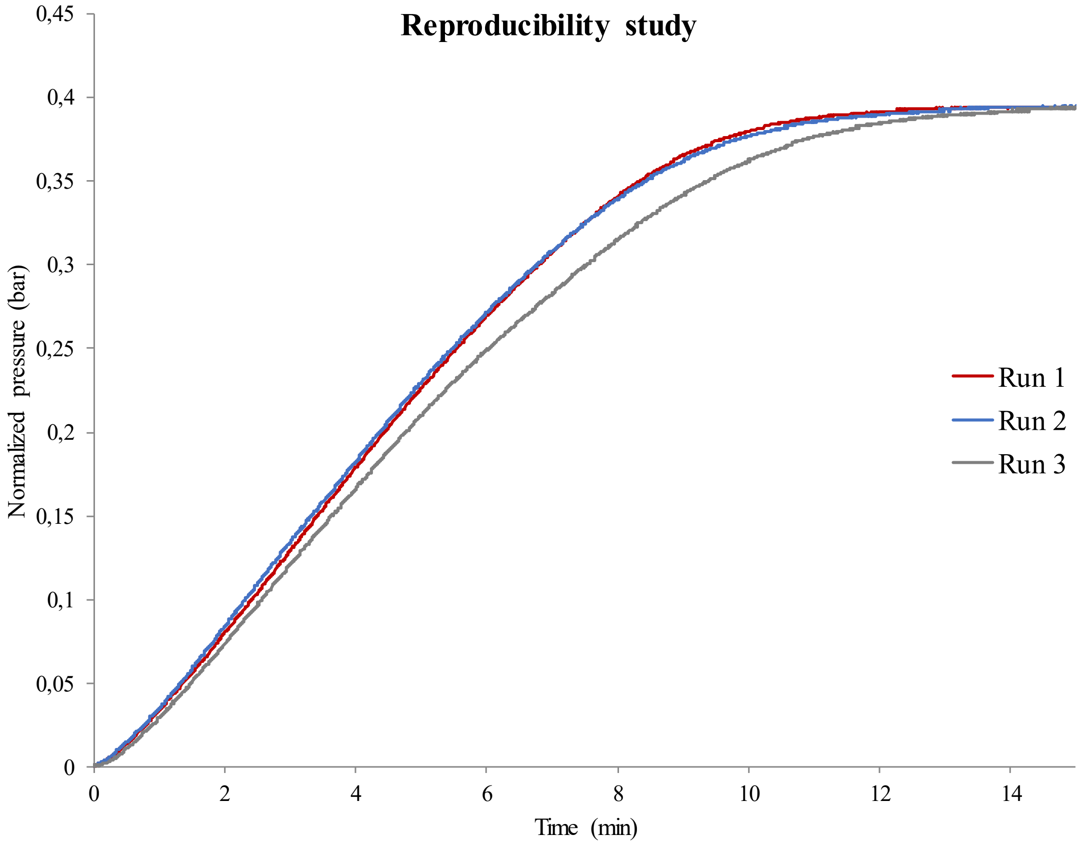

Figure S3: Three dimerization reaction profiles. The red and blue traces show the robustness of the technique. Initial concentrations: $[\mathbf{1a}]_0 = 0.1$ M; $[\mathbf{2}]_0 = 0.001$ M.

3. Synthesis of starting materials

*Preparation of Ph$_4$NHPI-DA (**1c**):*

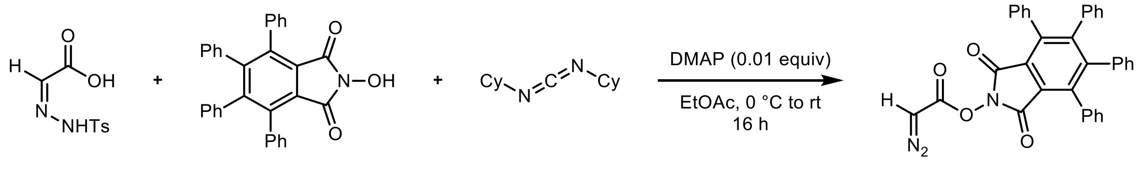

Under argon atmosphere at 0 °C, 2-(2-tosylhydrazono)acetic acid (1.455 g, 6.0 mmol, 1.2 equiv), 2-hydroxy-4,5,6,7-tetraphenylisoindoline-1,3-dione[3] (2.34 g, 5.0 mmol, 1 equiv) and *N*,*N*-dimethylpyridin-4-amine (6.11 mg, 0.050 mmol, 0.01 equiv) were suspended in dry ethyl acetate (20 mL). A solution of dicyclohexylmethanediimine (1.549 g, 7.5 mmol, 1.5 equiv) in 5 mL of dry ethyl acetate was added over 30 minutes. The reaction was warmed to room temperature and stirred overnight. After evaporation of the solvent, the crude was suspended in CH_2Cl_2 and filtered through a short plug of silica, concentrated, redissolved in the minimum amount of CH_2Cl_2, and triturated with pentane to give a slightly yellow solid. This was further purified by column chromatography (CH_2Cl_2) to afford the pure product **1c** (1.61 g, 60% yield).

Appearance: off-white solid.

TLC: R_f= 0.5 (5:1 pentane:ethyl acetate).

^{1}H NMR (400 MHz, CDCl$_3$): δ 7.24 – 7.16 (m, 6H), 7.16 – 7.05 (m, 4H), 6.96 – 6.84 (m, 6H), 6.79 – 6.68 (m, 4H), 5.03 (broad s, 1H).

^{13}C NMR (101 MHz, CDCl$_3$): δ 161.5, 148.8, 140.4, 137.8, 135.1, 130.8, 130.0, 127.7, 127.6, 127.5, 127.2, 126.6, 125.2, 44.9.

HRMS (ESI-TOF): calc'd for $[C_{34}H_{21}N_3O_4+Na]^+$: 558.1424; found: 558.1429.

4. Kinetic measurement – Dimerization reaction

Dimerization of ethyl diazoacetate (EDA) ***2a****:*

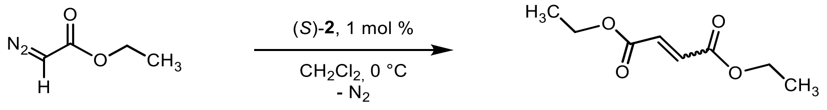

The measurement was performed following the general description in **Section 2**, employing ethyl diazoacetate **1b** (0.2 M solution in CH_2Cl_2, 0.225 mmol, 1.125 mL) and (*S*)-Ru-Pheox **2** (0.015 M solution in CH_2Cl_2, 2.25 μmol, 0.15 mL) in dry CH_2Cl_2 (total volume = 2.25 mL) at 0 °C.

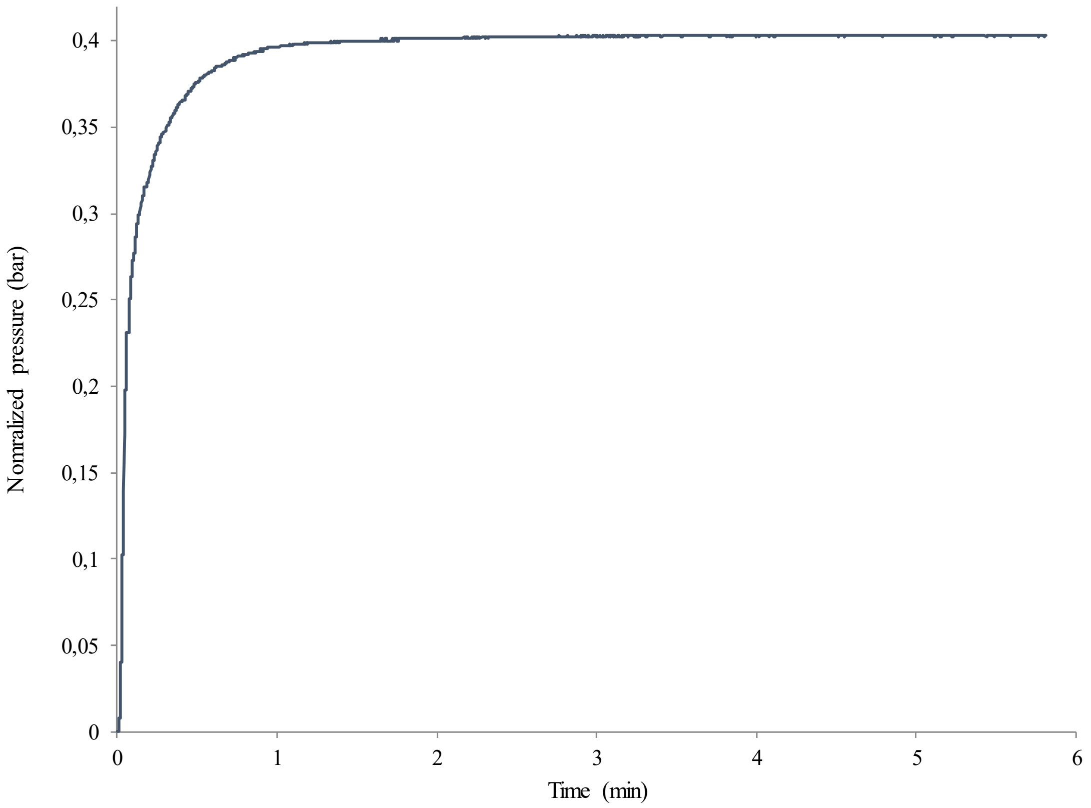

Figure S4: Dimerization profile of EDA **1b**. Initial concentrations: $[\mathbf{1b}]_0$ = 0.1 M; $[\mathbf{2}]_0$ = 0.001 M.

Dimerization of N-hydroxyphthalimidoyl diazoacetate (NHPI-DA) ***1a****:*

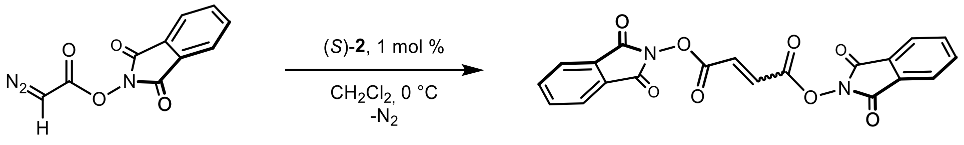

The measurement was performed following the general description in **Section 2**, employing NHPI-DA **1a** (0.205 M solution in CH_2Cl_2, 0.225 mmol, 1.098 mL) and (*S*)-Ru-Pheox **2** (0.015 M solution in CH_2Cl_2, 2.25 μmol, 0.15 mL) in dry CH_2Cl_2 (total volume = 2.25 mL) at 0 °C.

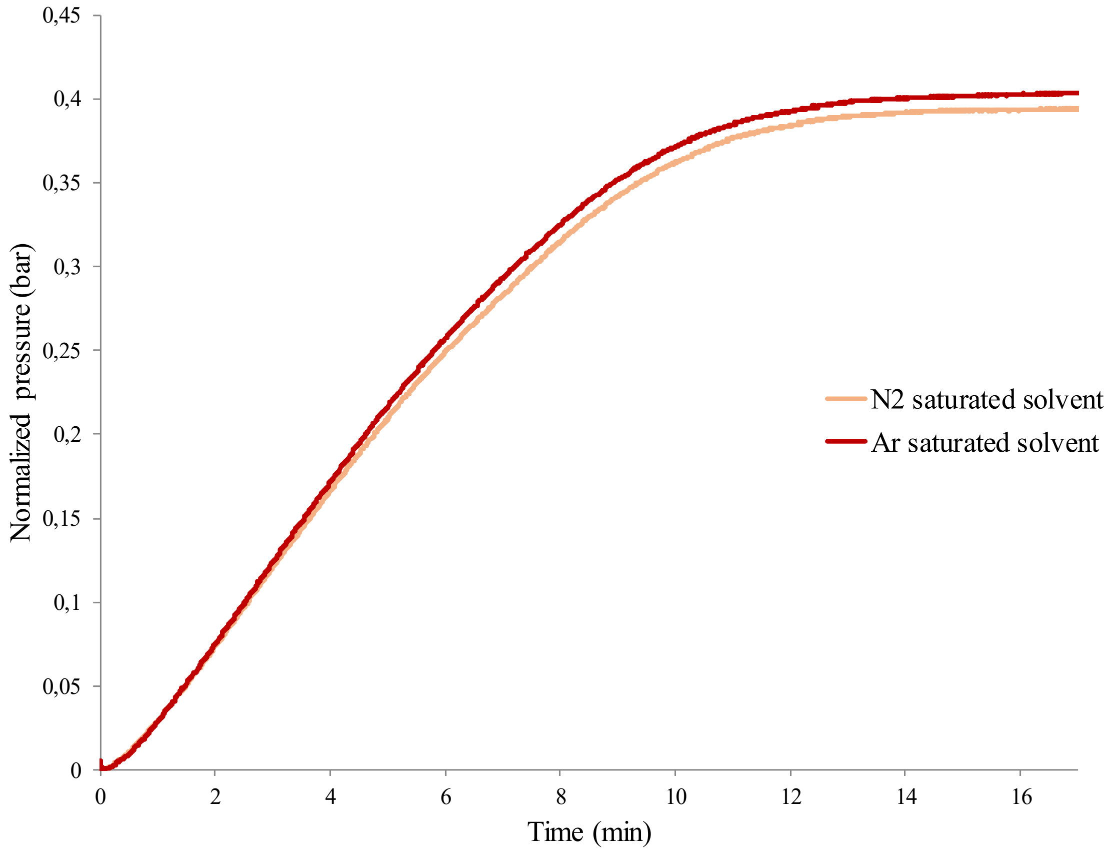

Figure S5: Dimerization profile of NHPI-DA **1a** in Ar- and N_2-saturated dichloromethane. Initial concentrations: [**1a**]$_0$ = 0.1 M; [**2**]$_0$ = 0.001 M.

To prove that the inizial induction period is not caused by the dissolution of N_2 in the reaction solvent, the same reaction was performed employig N_2-saturated dichloromethane. A similar profile was obtained.

5. Determination of the Nucleophilicity Parameter (*N*) for NHPI-DA (1a)

General Experimental Details:

UV-Vis measurements were performed in a quartz cuvette fitted with a septum and under Ar. All solutions were prepared under Ar using dry CH_2Cl_2 (distilled over CaH_2 and stored over 4 Å molecular sieves in a glovebox), and were kept in the glovebox. All the glassware was dried overnight in an oven. Stock solution were prepared in volumetric flasks, and the aliquots were added using glass Hamilton syringes. The temperature in the room where the experiments were performed was measured to be 19-21 °C. Benzhydrylium cation $(mfa)_2CH^+BF_4$ **8** was prepared following methods described in the literature.[4]

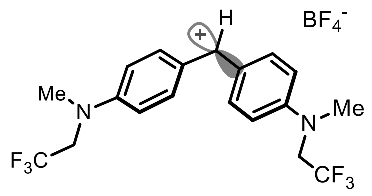

Benzhydrylium cation
*$(mfa)_2CH^+BF_4^-$ **(8)***

Figure S6: Structure of the benzhydrylium cation **8**.

Kinetics and determination of N:

According to the method described by Mayr and co-workers,[4] the apparent kinetic constant k_{obs} was calculated from the time profile of the absorbance data (eq. S1). Then, the bimolecular kinetic constant k_2 was calculated (eq. S2). The bimolecular kinetic constants calculated from the data of each run ($k_{2,i}$) were used to estimate the nucleophilicity parameter *N* for the run (N_i)(eq. S3). To apply this equation, we used the average of the slope parameter (s = 0.9275) obtained by Mayr and co-workers[4] for similar diazocompounds (**Table S1**) and the electrophilicity parameter for the benzhydrylium cation **8** (E = – 3.85). Mayr and co-workers determined that the slope parameter (s) has a very similar value across a series of different diazocompounds and recommended this approximation for preliminary estimation of nucleophilicity.[4] We averaged the results of three independent runs (n=3; eq. S4) taking the data on the initial 60 s (20 data points).

$$ln\frac{A_0 - A_{end}}{A_t - A_{end}} = k_{obs}t \qquad \text{(S1)}$$

$$k_2 = \frac{k_{obs}}{[\text{NHPI–DA}]} \qquad \text{(S2)}$$

$$N_i = \frac{logk_{2,i}}{\bar{s}} - E \qquad \text{(S3)}$$

$$\bar{N} = \frac{1}{n}\sum N_i \qquad \text{(S4)}$$

Table S1 – Nucleophile-dependent slope parameters by Mayr.[4]

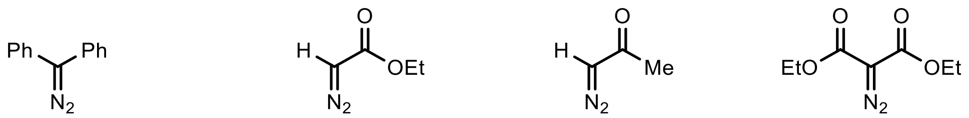

Diphenyldiazomethane | Ethyl diazoacetate **1b** | Diazoacetone | Diethyl diazomalonate

**Diazocompound**	**(s)**
Diphenyldiazomethane	0.92
Ethyl diazoacetate, EDA	0.95
Diazoacetone	0.91
Diethyl diazomalonate	0.93
**Average**	**0.9275**
$\sigma(s_{average})$	$1.708 \cdot 10^{-2}$

To obtain the confidence interval we used the partial derivatives of these functions (eq. S5-7). We calculated the standard error of the averaged nucleophilicity parameter N (eq. S8-10) and then estimated the confidence interval at 95% confidence of the normal distribution.

$$\frac{\partial k_2}{\partial k_{obs}} = \frac{1}{[\mathrm{NHPI{-}DA}]} \quad \text{(S5)}$$

$$\frac{\partial N_i}{\partial k_2} = \frac{1}{k_2 \bar{s}};\ \frac{\partial N_i}{\partial \bar{s}} = -\frac{\log k_2}{\bar{s}^2} \quad \text{(S6)}$$

$$\frac{\partial \bar{N}}{\partial N_i} = \frac{1}{n} \quad \text{(S7)}$$

$$\sigma_{k_2} \cong \sqrt{\left|\frac{\partial k_2}{\partial k_{obs}}\right|^2 \sigma_{k_{obs}}^2} = \frac{\sigma_{k_{obs}}}{[\mathrm{NHPI{-}DA}]} \quad \text{(S8)}$$

$$\sigma_{N_i} \cong \sqrt{\left|\frac{\partial N_i}{\partial k_2}\right|^2 \sigma_{k_2}^2 + \left|\frac{\partial N_i}{\partial \bar{s}}\right|^2 \sigma_{\bar{s}}^2} = \sqrt{\left(\frac{\sigma_{k_2}}{k_2 \bar{s}}\right)^2 - \left(\frac{\log k_2}{\bar{s}^2}\sigma_{\bar{s}}\right)^2} \quad \text{(S9)}$$

$$\sigma_{\bar{N}} \cong \sqrt{\left|\frac{\partial \bar{N}}{\partial N_i}\right|^2 \sigma_{N_i}^2} = \frac{1}{n}\sqrt{\sum \sigma_{N_i}^2};\ \Delta\bar{N} = \mathcal{N}(\alpha)\frac{\sigma_{\bar{N}}}{\sqrt{n}} \quad \text{(S10)}$$

*Procedure for kinetic measurements using $(mfa)_2CH^+BF_4^-$ (**8**)*

A quartz cuvette was charged with 2.0 mL of dry CH_2Cl_2 in a glovebox. After recording the blank, **8** was added from a stock solution and the UV-Vis spectrum of the blue solution was recorded. Next, partial UV-Vis spectrum (λ= 575-565 nm) was recorded every 2-2.4 s, first with **8** alone. After 1-2 minutes, the corresponding amount of diazoacetate **1** solution was added to the cuvette quickly under argon. The solution was made homogenous by shaking gently and placed in the spectrometer. The time elapsed

between the addition of the diazocompound and the first measurement was measured to be 6-10 s, depending on the experiment. The absorbance at t = 0 s is the average of the points obtained before the addition of the diazocompound, and it has been corrected with the corresponding dilution factor. The absorption of the diazocompound in this region of the spectrum is negligible.

*Recording of the absorbance vs time for NHPI-DA (**1a**) and $(mfa)_2CH^+BF_4^-$ (**8**)*

The procedure for kinetic measurements was followed for three different initial concentrations of **1a** and **8**. The variation of the absorbance at 569 nm with time (s) was plotted:

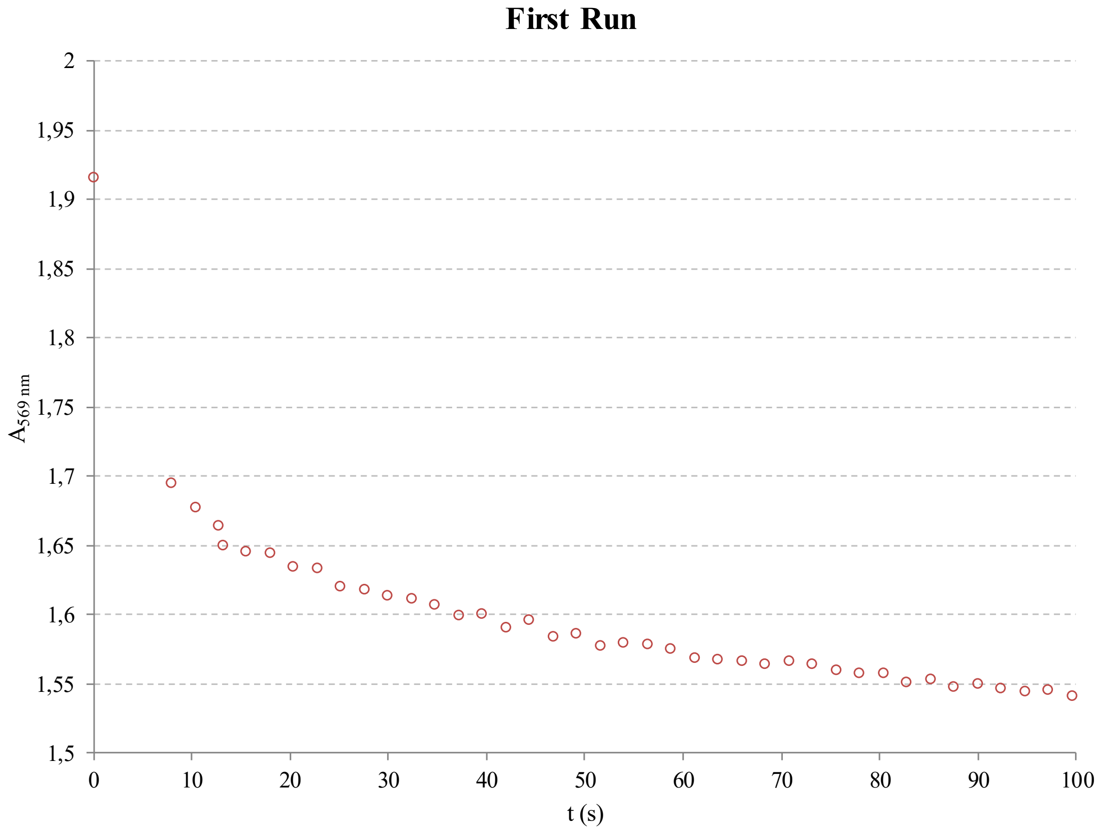

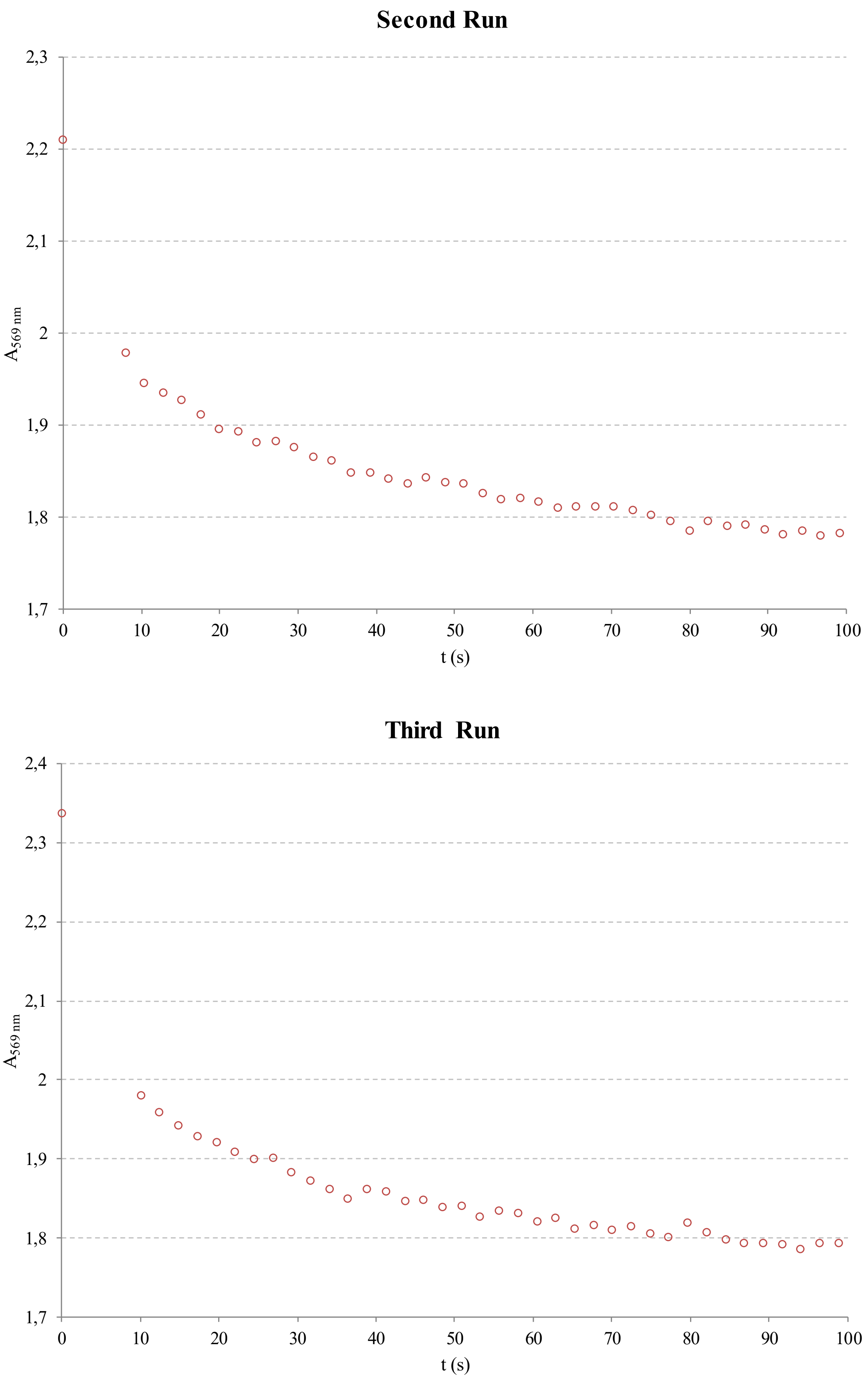

Figure S7: Variation of absorbance over time. Run 1; [**1a**]$_0$ = 2.113 mM, [**8**]$_0$ = 4.54·10^{-2} mM. Run 2; [**1a**]$_0$ = 2.721 mM, [**8**]$_0$ = 5.59·10^{-2} mM. Run 1; [**1a**]$_0$ = 2.568 mM, [**8**]$_0$ = 5.31·10^{-2} mM.

*Preliminary estimation of the nucleophilicity (N) of NHPI-DA (**1a**)*

As described in the *General Experimental Details* of this section, we used the kinetic data to obtain N_i for the corresponding initial concentrations in each run and calculated the statistics of the fit (**Table S2**).

Table S2 – Calculation of N through averaging of three independent kinetic runs (see **Figure S7**):

	#1	#2	#3
$[\mathbf{8}]_0$ (M)	$4.54 \cdot 10^{-5}$	$5.59 \cdot 10^{-5}$	$5.31 \cdot 10^{-5}$
$[\mathbf{1a}]_0$ (M)	$2.11 \cdot 10^{-3}$	$2.72 \cdot 10^{-3}$	$2.57 \cdot 10^{-3}$
k_{obs}	$0.00151(7) \pm 3.5 \cdot 10^{-5}$	$0.00166(9) \pm 4.8 \cdot 10^{-5}$	$0.00173(0) \pm 5.1 \cdot 10^{-5}$
σ (k_{obs})	$8.175 \cdot 10^{-5}$	$1.091 \cdot 10^{-4}$	$1.158 \cdot 10^{-4}$
R^2	0.9477	0.9248	0.9215
k_2	$0.71(8) \pm 0.017$	$0.61(3) \pm 0.018$	$0.67(3) \pm 0.020$
σ (k_2)	$3.868 \cdot 10^{-2}$	$4.010 \cdot 10^{-2}$	$4.509 \cdot 10^{-2}$
N_i	**3.69(5) ± 0.025**	**3.62(1) ± 0.031**	**3.66(5) ± 0.032**
σ (N_i)	$5.803 \cdot 10^{-2}$	$7.036 \cdot 10^{-2}$	$7.211 \cdot 10^{-2}$
$\bar{N}$	**3.66 ± 0.04**		
σ ($\bar{N}$)	$3.875 \cdot 10^{-2}$		

With these individual N_i values, we obtained an averaged nucleophilicity parameter for NHPI-DA, **1a** (eq. S11):

$$\bar{\boldsymbol{N}} = 3.66 \pm 0.04 \qquad \text{(S11)}$$

NOTE: This confidence interval should not be over-interpreted in absolute terms and only as an estimate of the consistency of our data. Further studies would be required to estimate N with high-accuracy using other benzhydrylium cations under stop-flow conditions.

*Validation of the experimental set-up: nucleophilicity (N) of ethyl diazoacetate (**1b**)*

We have reproduced in-house the nucleophilicity (N) value determined by Mayr[4] for the benchmark ethyl diazoacetate **1b** to validate our experimental set-up, using the same benzhydrylium cation **8**. The general procedure for kinetic measurements was followed, resulting in a faster decay of the absorbance over time (**Figure S8**), due to the higher nucleophilicity of **1b**.

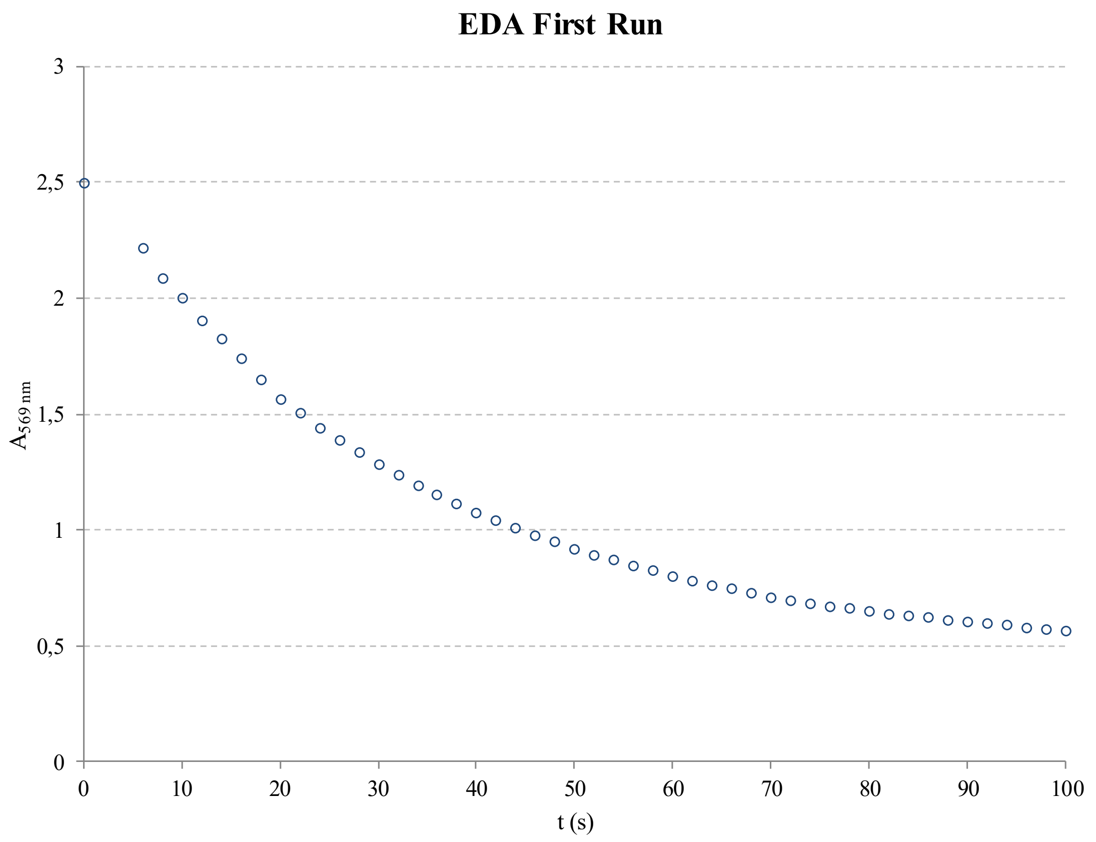

Figure S8: Variation of absorbance over time for EDA. [**1b**]$_0$ = 2.501 mM, [**8**]$_0$ = 5.29·10^{-2} mM.

Identical mathematical treatment resulted in a nucleophilicity value in agreement with the one reported by Mayr and co-workers (eq. S12-13):

$N_{\mathbf{1b}} = 4.91$ Mayr[37] (S12)

$N_{\mathbf{1b}} = 4.82(3)$ (*in-house*) (S13)

*Qualitative nucleophilicity comparison between EDA (**1b**) and NHPI-DA (**1a**) vs. a reference benzhydrylium electrophile **8**.*

For a better comparison of the nucleophilicity of diazocompounds **1a** and **1b**, we have plotted the variation of the conversion over time for both diazocompounds at equal concentrations (**Figure S9**):

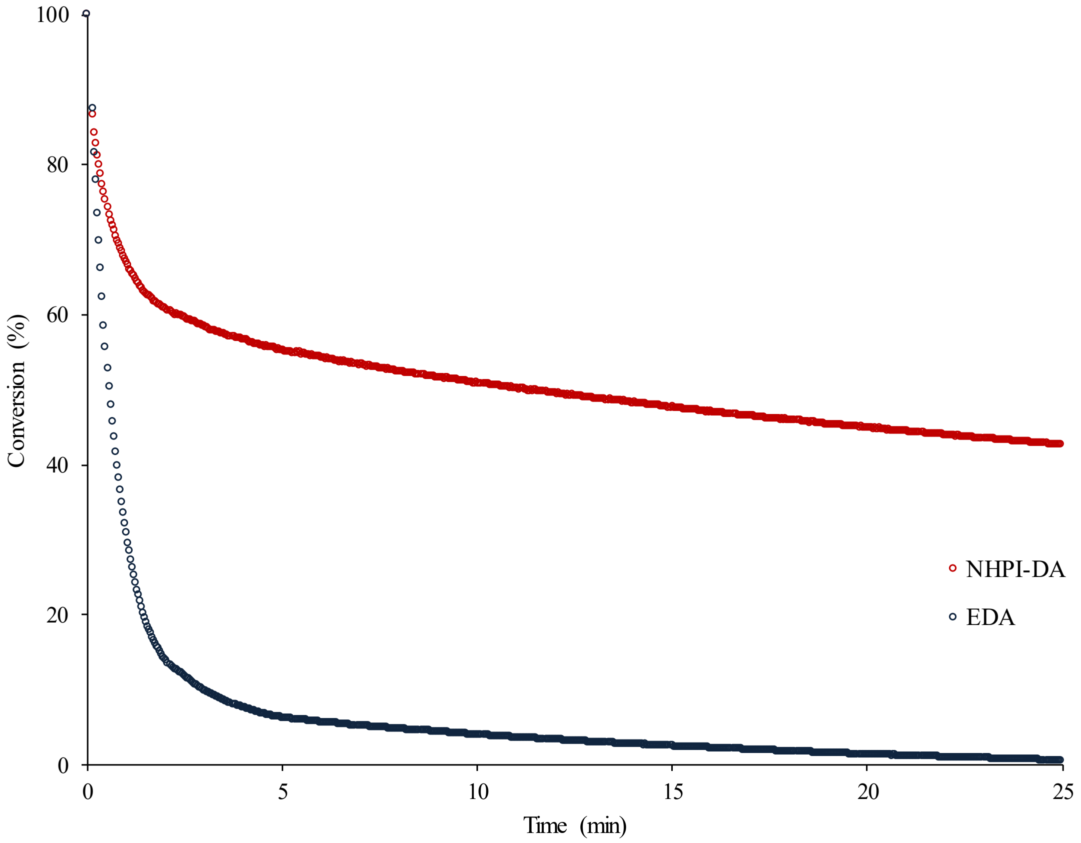

Figure S9: Comparison of the reactivity of **1a** (NHPI-DA, red) and **1b** (EDA, blue) with **8**.

6. Kinetic measurement – Cyclopropanation of 1-hexene (4d)

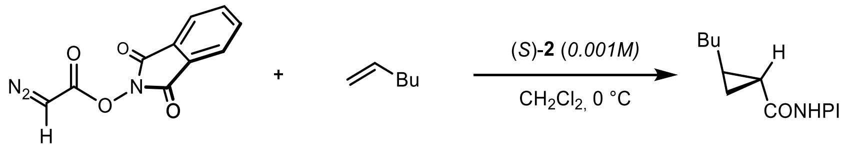

1a 0.1 M | **4d** 0.1 M *or* 0.5 M | **3d** *0.1 M:* 72%, 7:1 d.r., 97:3 e.r. *0.5 M:* > 95%, 7:1 d.r., 97:3 e.r.

Data set: [**4d**]$_0$ = 0.5 M

The measurement was performed following the general description in **Section 2**, employing 1-hexene **4d** (1.125 mmol, 0.14 mL), NHPI-DA **1a** (0.205 M solution in CH_2Cl_2, 0.225 mmol, 1.1 mL) and (*S*)-Ru-Pheox **2** (0.015 M solution in CH_2Cl_2, 2.25 µmol, 0.15 mL) in dry CH_2Cl_2 (total volume = 2.25 mL) at 0 °C.

Data set: [**4d**]$_0$ = 0.1 M

The measurement was performed following the general description in **Section 2**, employing 1-hexene **4d** (0.3 M solution in CH_2Cl_2, 0.225 mmol, 0.75 mL), NHPI-DA **1a** (0.205 M solution in CH_2Cl_2, 0.225 mmol, 1.1 mL) and (*S*)-Ru-Pheox **2** (0.015 M solution in CH_2Cl_2, 2.25 µmol, 0.15 mL) in dry CH_2Cl_2 (total volume = 2.25 mL) at 0 °C.

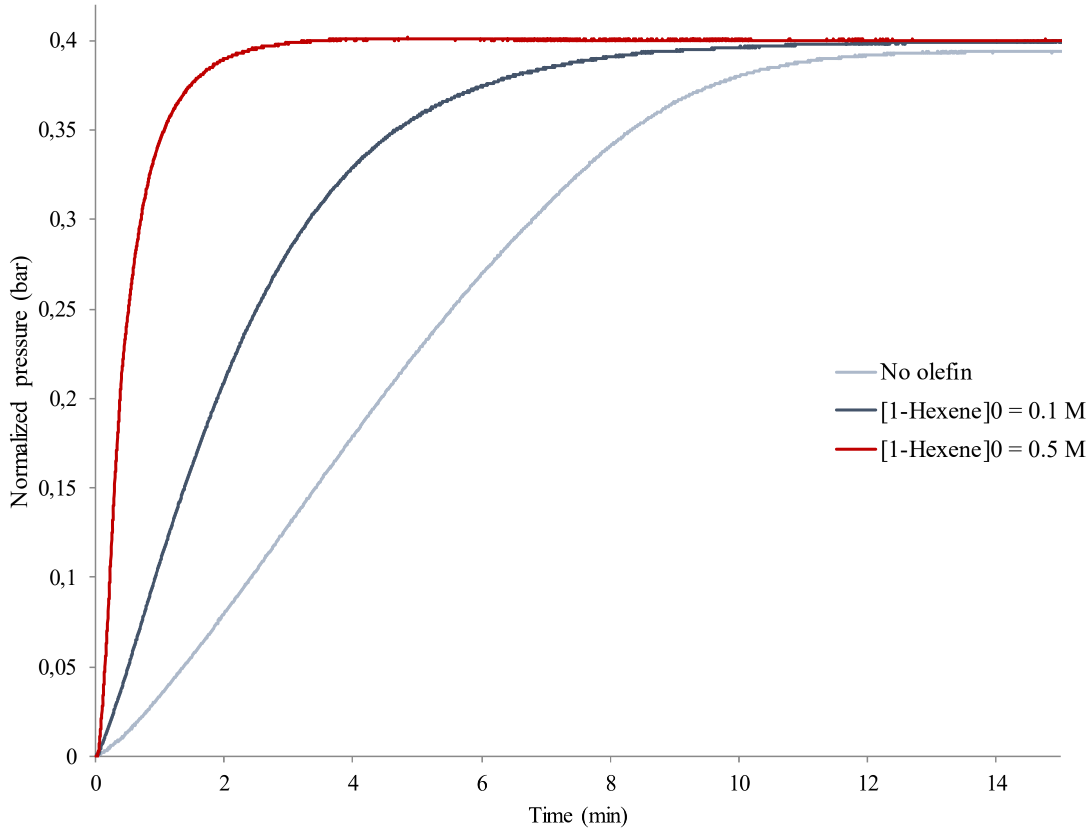

Figure S10: Kinetic profile of the cyclopropanation of 1-hexene **4d** with NHPI-DA **1a** and (*S*)-**2**. Initial concentrations: [**4d**]$_0$ = 0.5 M (red trace); [**4d**]$_0$ = 0.1 M (blue trace); [**1a**]$_0$ = 0.1 M; [**2**]$_0$ = 0.001 M.

Determination of reaction order in olefin

The overlay plot[5-7] was generated calculating the fraction of nitrogen derived from the cyclopropanation cycle. In order to do that, several reactions were analyzed by ^{1}H NMR to determine the concentration ratio of product **3d** to dimer **7a**. The reactions were monitored by N_2 evolution and stopped by addition of excess methanol once the right conversion was reached. The plot below summarizes the results:

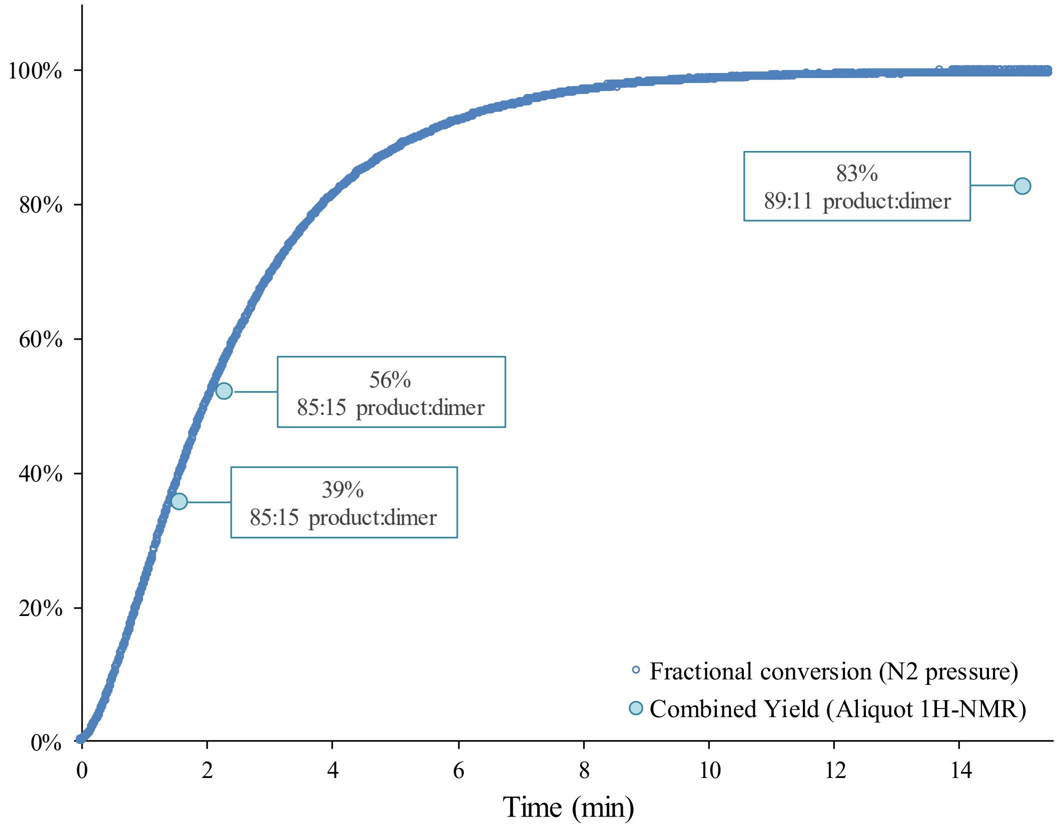

Figure S11: Summary of the product:dimer (**3d**: **7a**) ratio determination by ^{1}H NMR analysis.

From these experiments, it resulted that until 56% conversion, the ratio was constant with a value of 85:15 for the product **3d**. This allowed us to estimate the pressure of nitrogen generated by the cyclopropanation cycle using the ideal gas approximation (eq. S14):

$$P_{cyclopropanation} = P_{tot} * (product\ ratio) = P_{tot} * 0.85 \qquad \text{(S14)}$$

This pressure was then used in VTNA as shown by Burés and co-workers to obtain order in olefin = 1 (**Figure S14**). Additionally, the linearity of the plot informs us that the order in diazo = 0. Below are shown the VTNA plots, with order values 0 (**Figure S12**), 0.9 (**Figure S13**), 1 (**Figure S14**) and 1.1 (**Figure S15**) respectively.

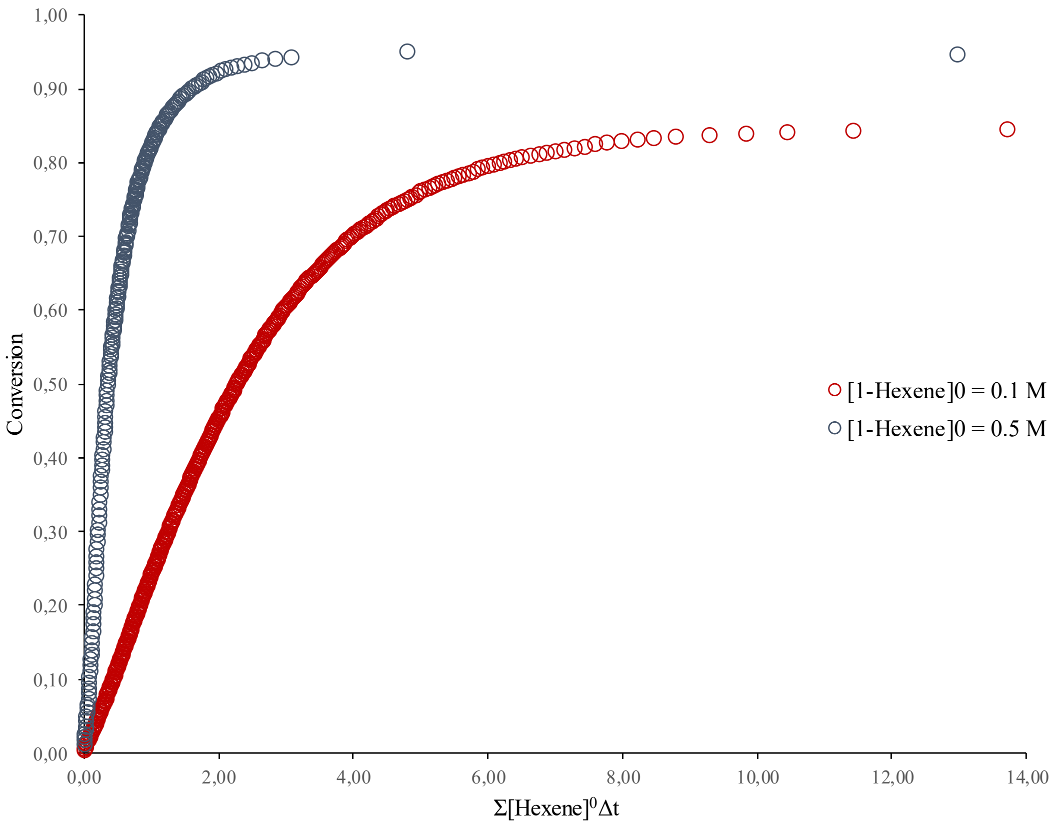

Figure S12: VTNA plot (order = 0). No overlay is observed.

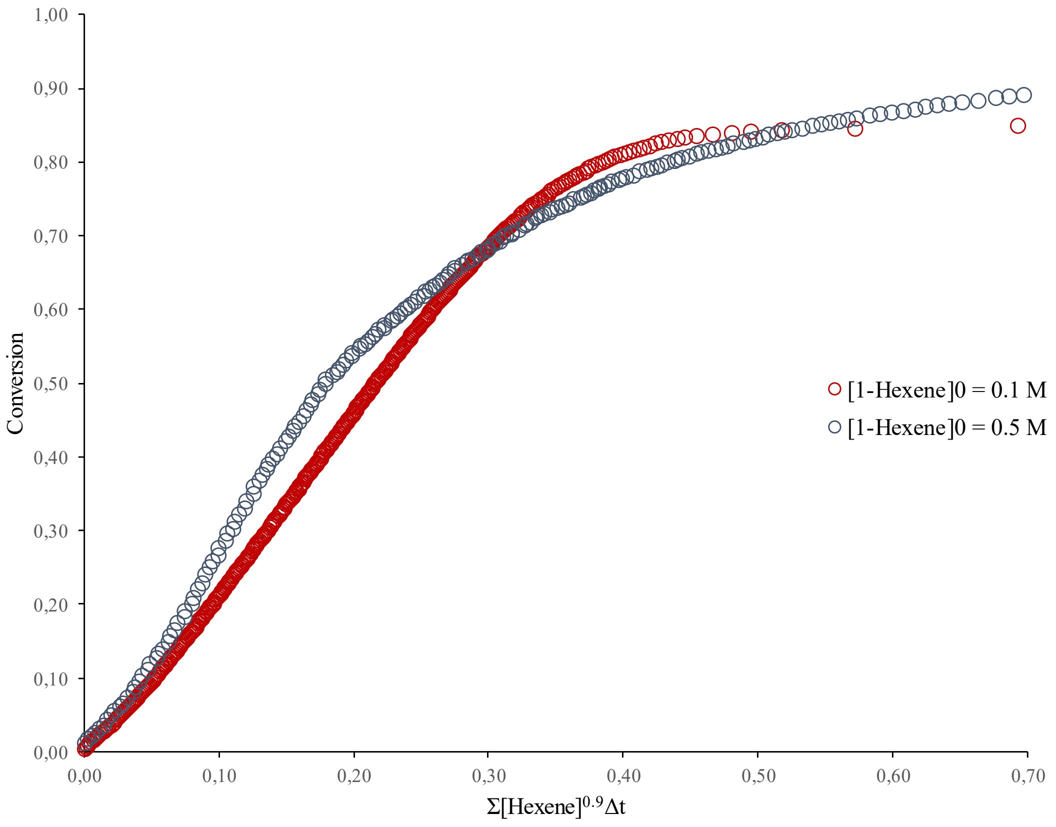

Figure S13: VTNA plot (order = 0.9). No overlay is observed.

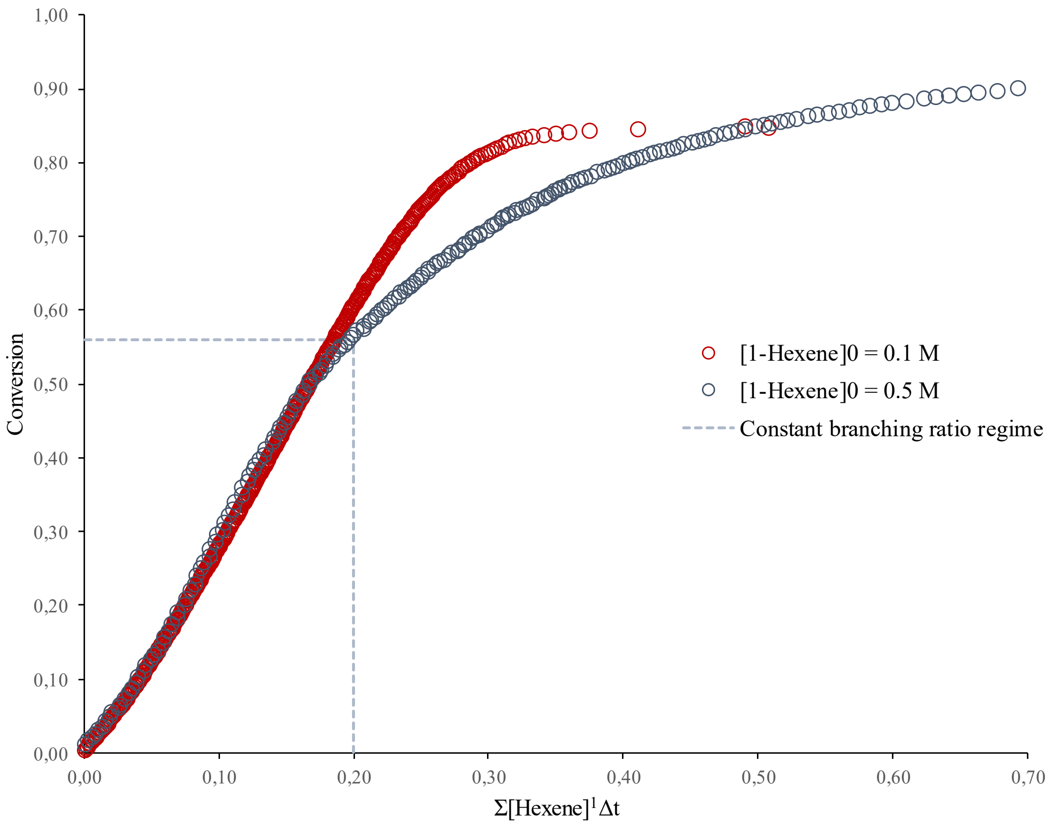

Figure S14: VTNA plot (order = 1). A clear overlay is observed within 56% conversion (constant branching ratio regime). The overlay is lost at higher conversion values.

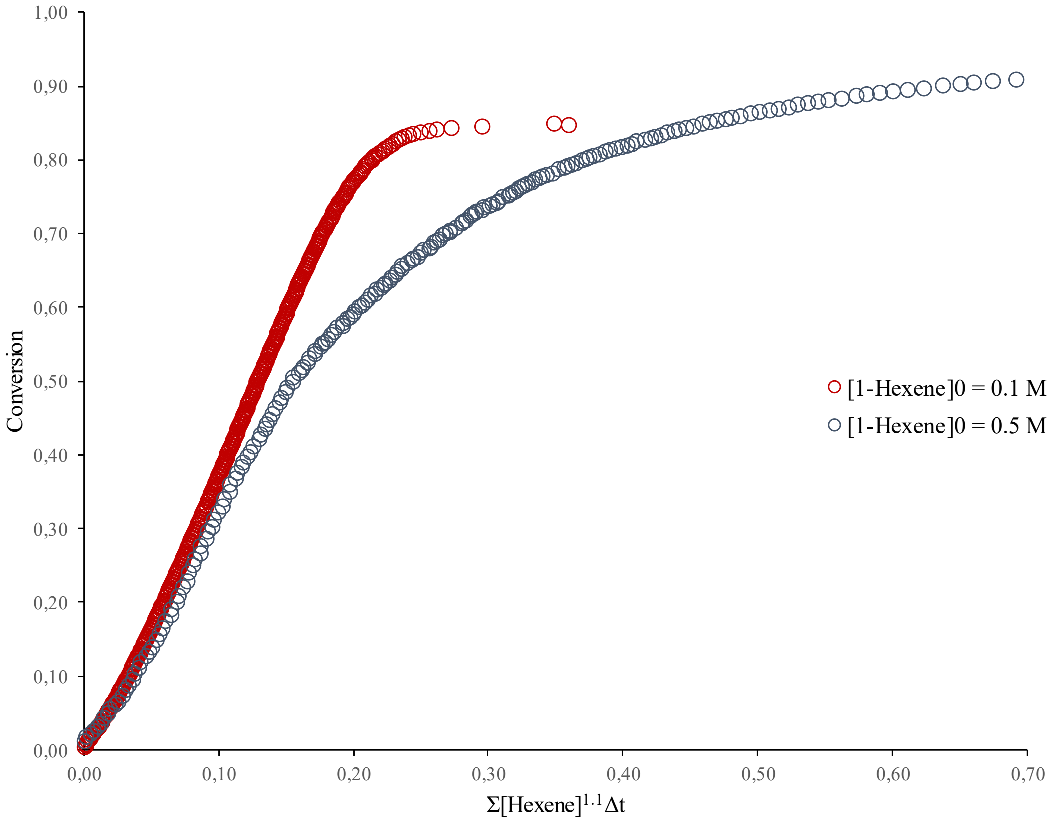

Figure S15: VTNA plot (order = 1.1). No overlay is observed.

7. Kinetic measurement – Cyclopropanation of 4-methylstyrene (4b)

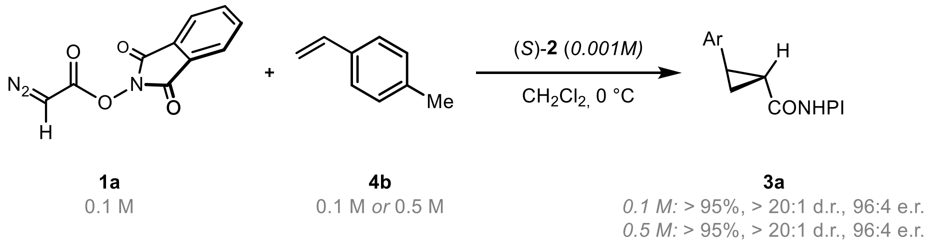

Data set: [**4b**]$_0$ = 0.5 M

The measurement was performed following the general description in **Section 2**, employing 4-methylstyrene **4b** (1.128 mmol, 0.15 mL), NHPI-DA **1a** (0.205 M solution in CH_2Cl_2, 0.225 mmol, 1.1 mL) and (*S*)-Ru-Pheox **2** (0.015 M solution in CH_2Cl_2, 2.25 µmol, 0.15 mL) in dry CH_2Cl_2 (total volume = 2.25 mL) at 0 °C.

Data set: [**4b**]$_0$ = 0.1 M

The measurement was performed following the general description in **Section 2**, employing 4-methylstyrene **4b** (0.3 M solution in CH_2Cl_2, 0.225 mmol, 0.75 mL), NHPI-DA **1a** (0.205 M solution in CH_2Cl_2, 0.225 mmol, 1.1 mL) and (*S*)-Ru-Pheox **2** (0.015 M solution in CH_2Cl_2, 2.25 µmol, 0.15 mL) in dry CH_2Cl_2 (total volume = 2.25 mL) at 0 °C.

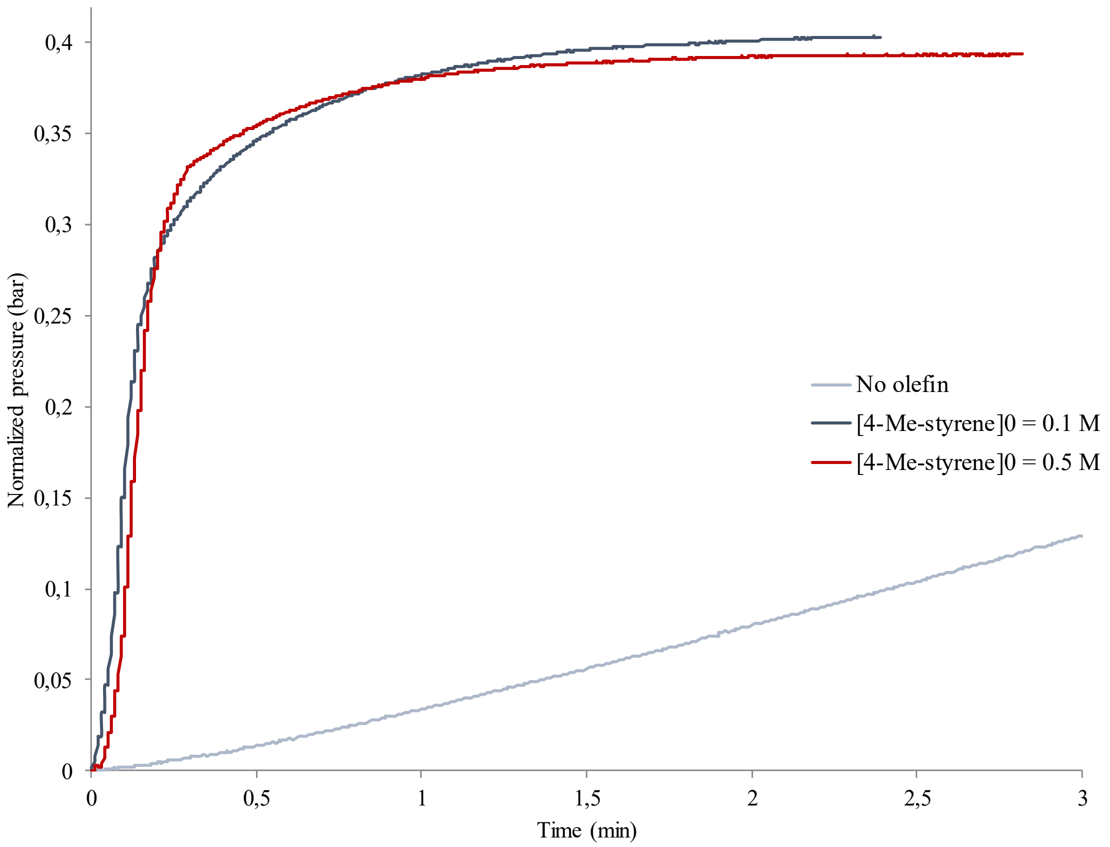

Figure S16: Kinetic profile of the cyclopropanation of 4-methylstyrene **4b** with NHPI-DA **1a** and (S)-**2**. Initial concentrations: [**4b**]$_0$ = 0.5 M (red trace); [**4b**]$_0$ = 0.1 M (blue trace); [**1a**]$_0$ = 0.1 M; [**2**]$_0$ = 0.001 M.

8. Stoichiometry experiments

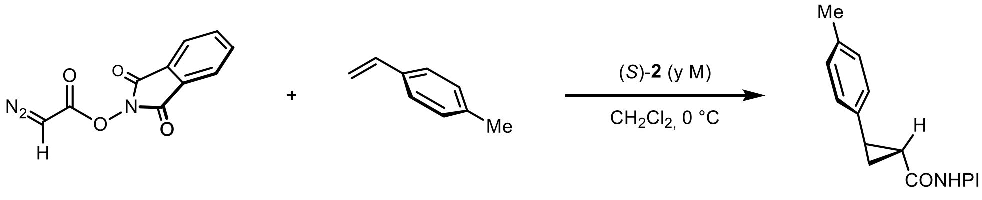

Entry	x (M)	y (M)	$[2]_0/[1a]_0$	Yield (%)	e.r.
1	0.1	0.001	0.01	97	96:4
2	0.2	0.1	0.5	95	93:7
3	0.1	0.1	1	96	94:6
4	0.05	0.1	2	97	96:4
5	0.02	0.1	5	95	96:4

To a solution of (*S*)-RuPheox **2** (0.100 mmol, 63.0 mg) in dry CH_2Cl_2 (total volume = 1.0 mL) was added 4-methylstyrene **4b** (66 µL, 0.500 mmol). The solution was cooled to 0 °C and to it was added the indicated amount of NHPI-DA **1a** (0.25 M in CH_2Cl_2) in one portion. The mixture was stirred for 10 minutes, then the solvent was removed, and the crude analyzed by ^{1}H-NMR. The crude product **3a** was purified by preparative TLC to determine the enantiomeric purity by HPLC (**Section 11.a**) using a Chiralpak-IA [hexane:*i*PrOH (90:10), 1.0 mL/min]: τ_{major} = 13.4 min, τ_{minor} = 12.1 min.

9. Sequential experiments with Ph_4-NHPI-DA (1c)

*Synthesis of 1,3-dioxoisoindolin-2-yl (1R,2R,3R)-2-methyl-3-phenylcyclopropane-1-carboxylate **3b**:*

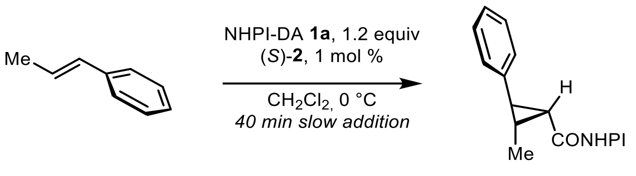

Under argon atmosphere at 0°C, to a solution of (*S*)-Ru-Pheox **2** (2,53 mg, 4,00 µmol) and *trans*-β-methylstyrene **4c** (52 µL, 0.4 mmol) in dry CH_2Cl_2 (2.4 mL), was added a solution of NHPI-DA **1a** (1.6 mL, 0,480 mmol) in dry CH_2Cl_2 over the course of 40 minutes using a syringe pump. After the addition was done, the solvent was removed and the product purified by column chromatography (pentane:ethyl acetate 4:1) to afford the pure product **3b** (61 mg, 48%, dr: > 20:1). Compound (*R,R,R*)-**3b** was obtained with a 82.7:17.3 enantiomeric ratio determined by HPLC (**Section 11.b**) using a Chiralpak-IA column [hexane/*i*-PrOH (90:10), 1.0 mL/min]: τ_{major} = 12.1 min, τ_{minor} = 10.6 min or a Chiralpak-IC column [hexane/*i*-PrOH (90:10), 1.0 mL/min]: τ_{major} = 10.0 min, τ_{minor} = 11.7 min.

***Note:** A similar reaction performed using Ph_4-NHPI-DA **1c** instead of NHPI-DA **1a** yielded no product.*

Appearance: white solid.

TLC: R_f= 0.6 (4:1 pentane:ethyl acetate).

^{1}H NMR (400 MHz, $CDCl_3$): δ 7.95 – 7.85 (m, 2H), 7.83 – 7.75 (m, 2H), 7.38 – 7.28 (m, 2H), 7.28 – 7.20 (m, 1H), 7.18 – 7.09 (m, 2H), 2.59 (dd, J = 7.0, 4.9 Hz, 1H), 2.35 (dd, J = 9.1, 4.9 Hz, 1H), 2.08 – 1.93 (m, 1H), 1.43 (d, J = 6.2 Hz, 3H).

^{13}C NMR (101 MHz, $CDCl_3$): δ 168.0, 162.2, 139.0, 134.9, 129.1, 128.8, 127.1, 126.4, 124.1, 34.6, 27.2, 26.0, 12.1.

HRMS (ESI-TOF): calc'd for $[C_{19}H_{15}NO_4+Na]^+$: 344.0893; found: 344.0896.

*Synthesis of 1,3-dioxo-4,5,6,7-tetraphenylisoindolin-2-yl (1R,2R)-2-(p-tolyl)cyclopropane-1-carboxylate **3c**:*

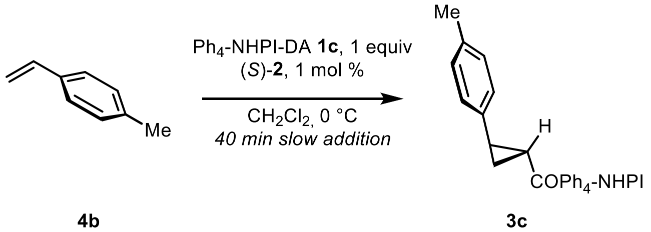

Under argon atmosphere at 0°C, to a solution of (*S*)-Ru-Pheox **2** (0.3 mg, 1,00 µmol) and *p*-methylstyrene **4b** (10 µL, 0.075 mmol) in dry CH_2Cl_2 (0.5 mL), was added Ph_4-NHPI-DA **1c** (27 mg, 0,050 mmol) in one portion. After the reaction was complete, the solvent was removed and the product purified by preparative TLC to analyze the stereoselectivity (^{1}H-NMR yield: 86%, dr: > 20:1). Compound (*R,R*)-**3c** was obtained with a 97.2:2.8 enantiomeric ratio determined by HPLC (**Section**

11.c) using a Chiralpak-IF column [hexane/*i*-PrOH (80:20), 1.0 mL/min]: τ_{major} = 6.9 min, τ_{minor} = 9.1 min.

Appearance: white solid.

TLC: R_f= 0.3 (4:1 pentane:ethyl acetate).

^{1}H NMR (400 MHz, $CDCl_3$): δ 7.23 – 7.17 (m, 6H), 7.16 – 7.10 (m, 4H), 7.10 – 7.03 (m, 2H), 7.01 – 6.96 (m, 2H), 6.94 – 6.86 (m, 6H), 6.75 (dd, J = 6.7, 2.7 Hz, 4H), 2.67 (ddd, J = 9.3, 6.9, 4.1 Hz, 1H), 2.30 (s, 3H), 2.09 – 2.02 (m, 1H), 1.75 – 1.66 (m, 1H), 1.49 (ddd, J = 8.3, 6.9, 4.8 Hz, 1H).

^{13}C NMR (101 MHz, $CDCl_3$): δ 169.7, 161.4, 148.7, 140.3, 137.9, 136.8, 135.6, 135.2, 130.8, 130.0, 129.4, 127.7, 127.5, 127.2, 126.6, 126.5, 125.3, 27.9, 21.2, 20.9, 18.0.

HRMS (ESI-TOF): calc'd for $[C_{43}H_{31}NO_4+Na]^+$: 648.2145; found: 648.2140.

Sequential experiment with different diazoreagents:

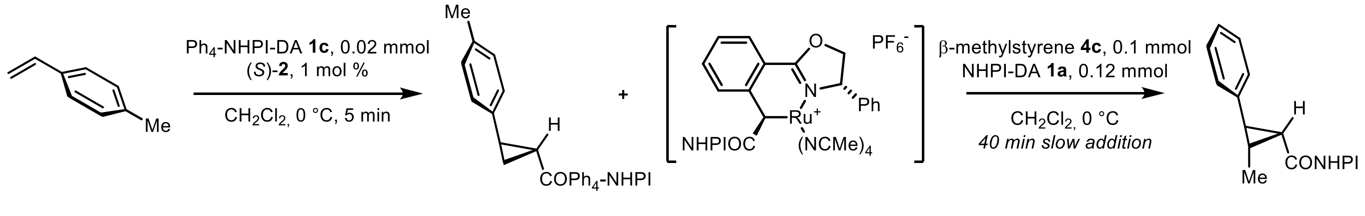

To a solution of *p*-methylstyrene **4b** (4.0 µL, 0.030 mmol, 0.30 equiv) and (*S*)-Ru-Pheox (0.6 mg, 0.001 mmol, 0.01 equiv) in dry CH_2Cl_2 (0.2 mL) at 0 °C, was added a solution of Ph_4-NHPI-DA **1c** (10.7 mg, 0.020 mmol, 0.20 equiv) in dry CH_2Cl_2 (0.2 mL) in one portion. The mixture was stirred at that temperature for 5 minutes, then *trans*-β-methylstyrene **4c** (13 µL, 0.100 mmol, 1.0 equiv) was added, followed by a solution of NHPI-DA **1a** (27.7 mg, 0.120 mmol, 1.2 equiv) in dry CH_2Cl_2 (0.6 mL) over 40 minutes. After complete consumption of NHPI-DA, the solvent was removed and the mixture purified by preparative TLC to analyze the stereoselectivity (^{1}H-NMR yield of **3b**: 47%, dr: > 20:1). Compound (*R,R,R*)-**3b** was obtained with a 81.6:18.3 enantiomeric ratio determined by HPLC (**Section 11.d**) using a Chiralpak-IA column [hexane/*i*-PrOH (90:10), 1.0 mL/min]: τ_{major} = 12.8 min, τ_{minor} = 10.7 min or a Chiralpak-IC column [hexane/*i*-PrOH (90:10), 1.0 mL/min]: τ_{major} = 10.4 min, τ_{minor} = 12.3 min.

10.NMR spectra of synthesized compounds

^{1}H NMR (400 MHz, CH_2Cl_2) for (S)-RuPheox(CH_3CN)$_4$

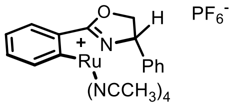

2

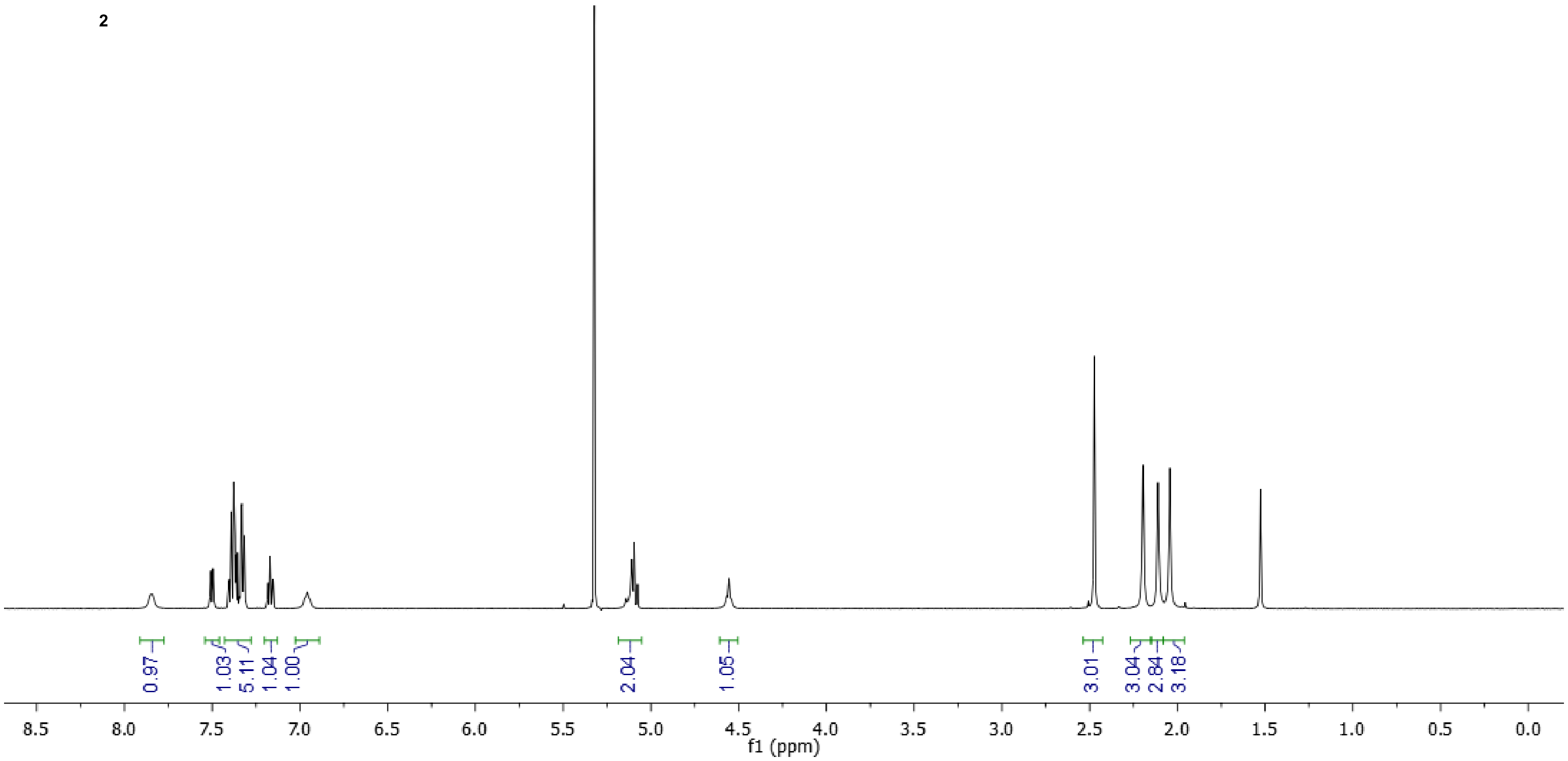

*^{1}H NMR (400 MHz, $CDCl_3$) for Ph_4-NHPI-DA (**1c**)*

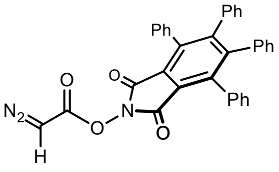

1c

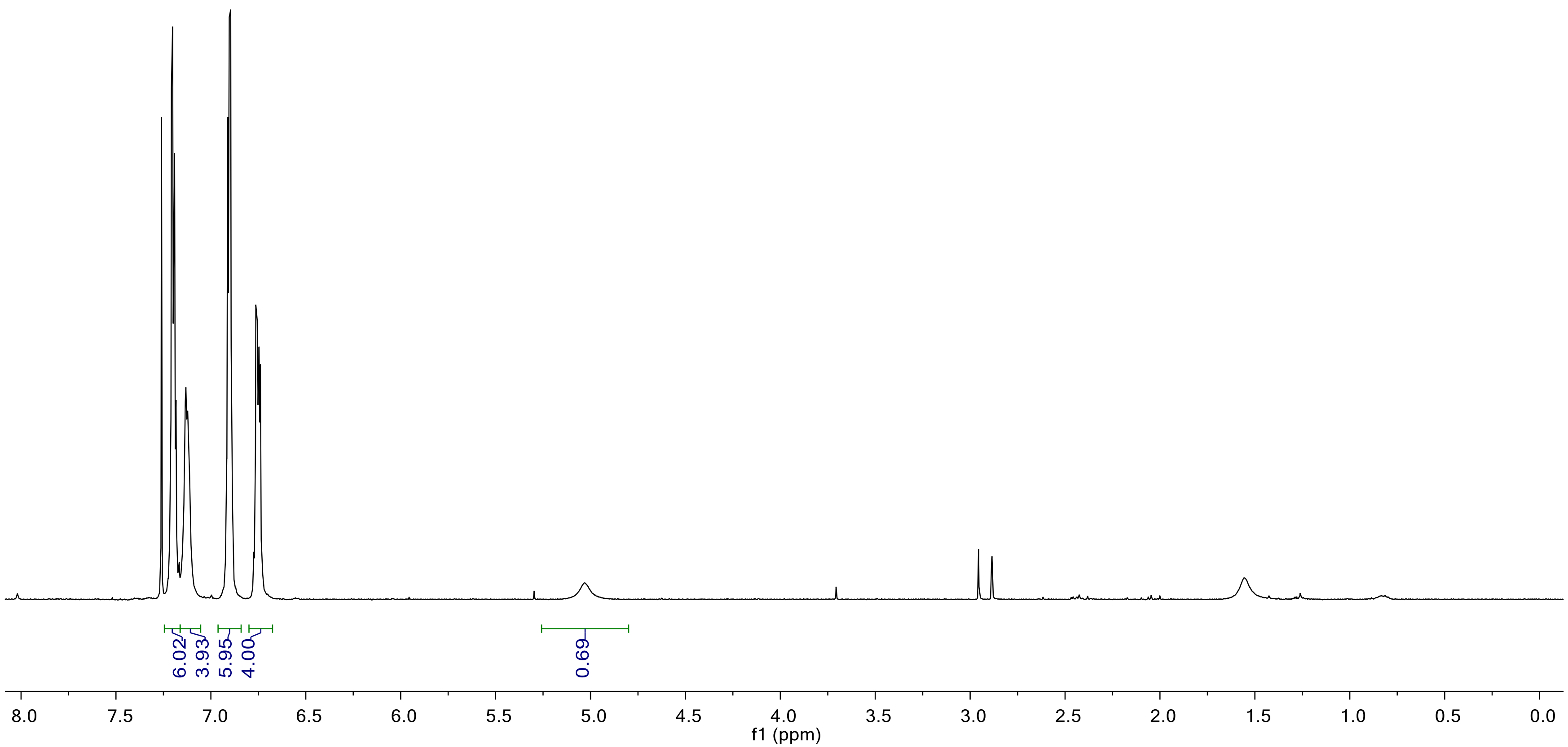

*^{13}C NMR (400 MHz, $CDCl_3$) for Ph_4-NHPI-DA (**1c**)*

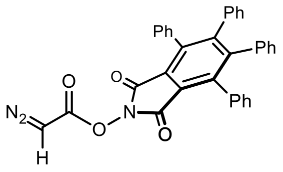

1c

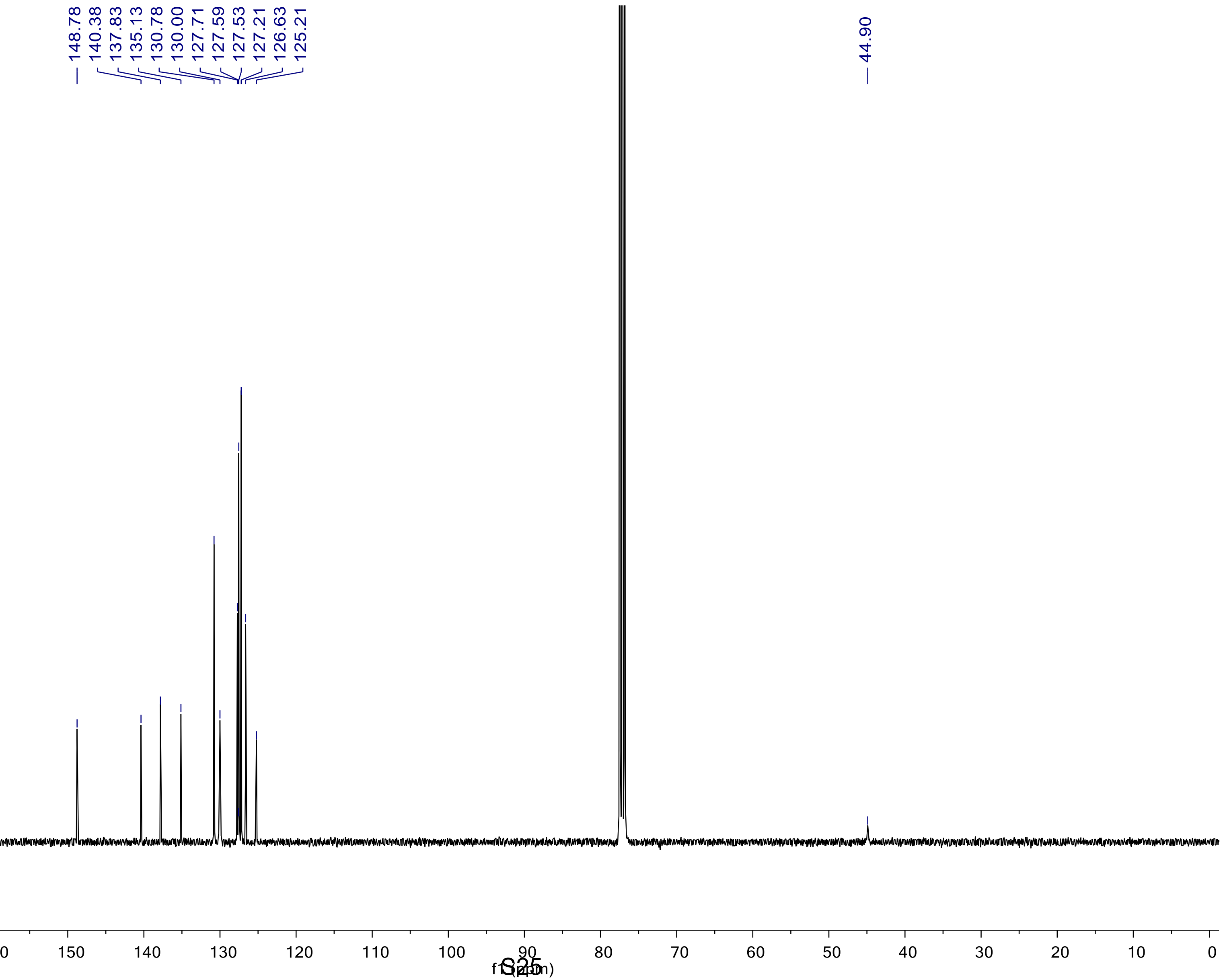

*^{1}H NMR (400 MHz, $CDCl_3$) for compound (**3b**)*

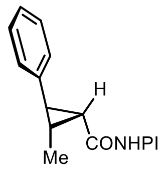

3b

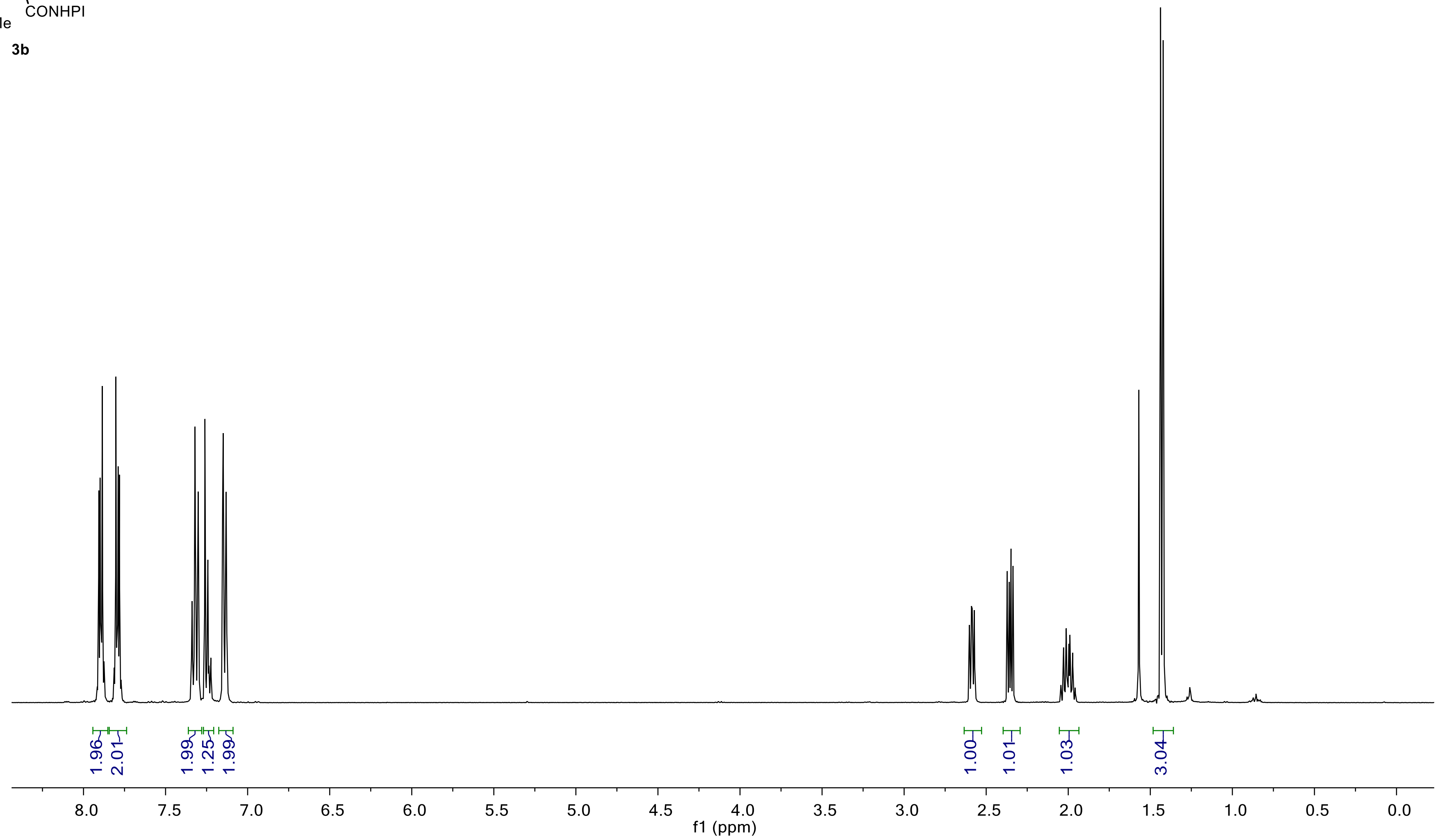

*^{13}C NMR (400 MHz, $CDCl_3$) for compound (**3b**)*

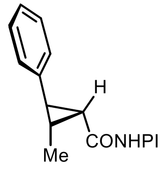

3b

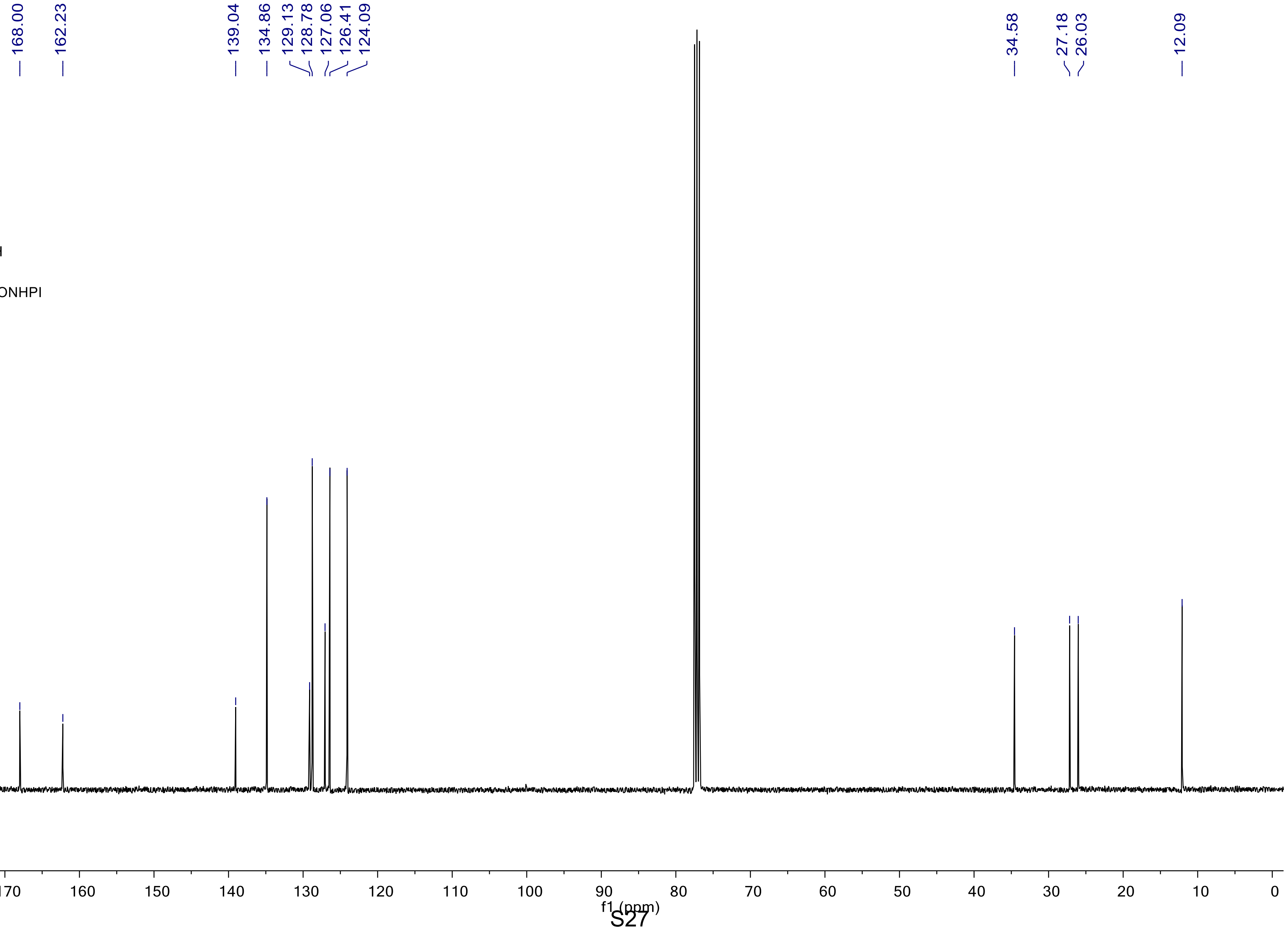

*^{1}H NMR (400 MHz, $CDCl_3$) for compound (**3c**)*

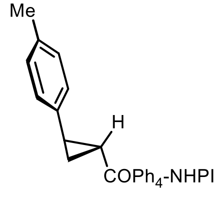

3c

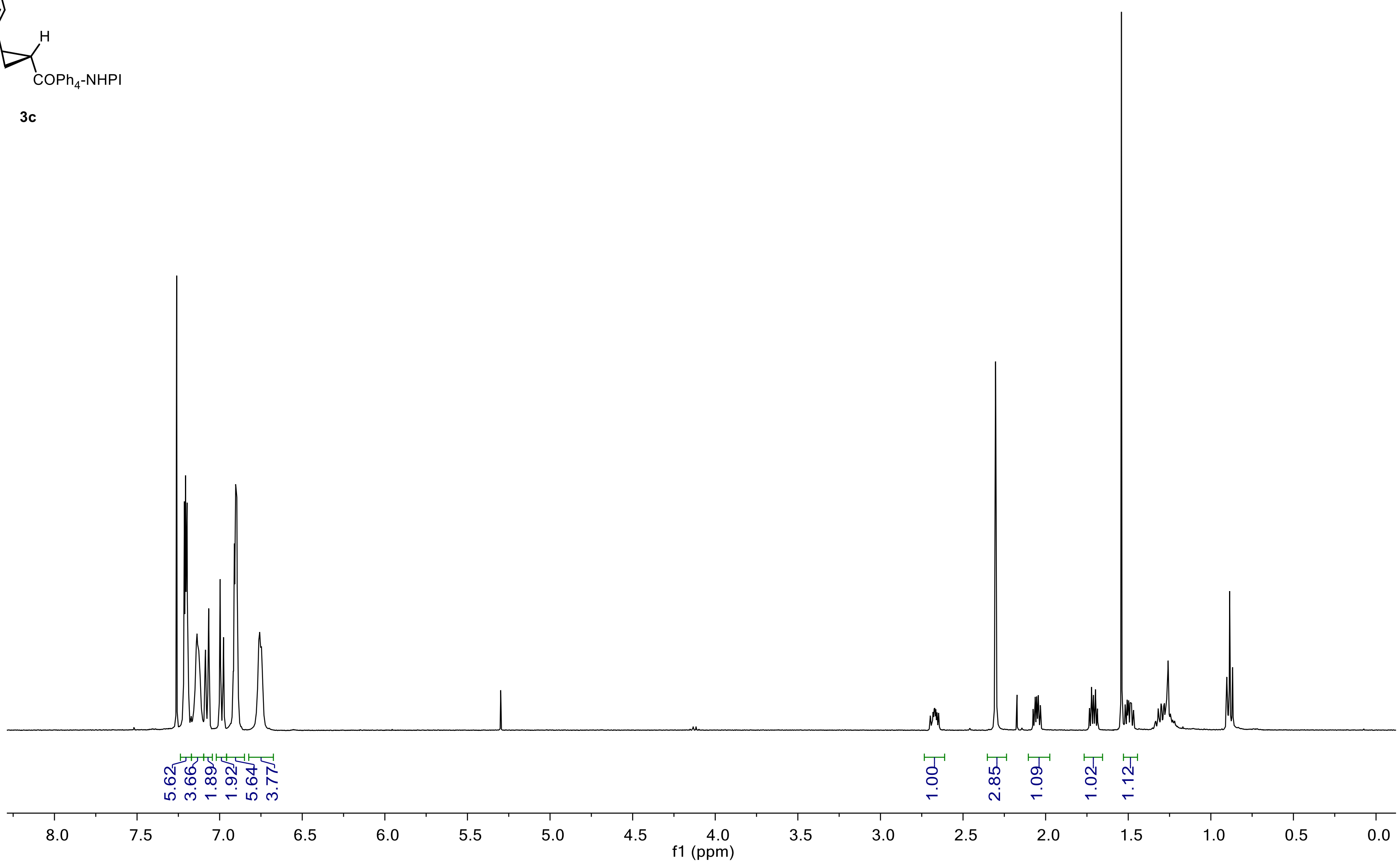

^{13}C NMR (400 MHz, $CDCl_3$) for compound (3c)

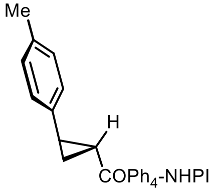

3c

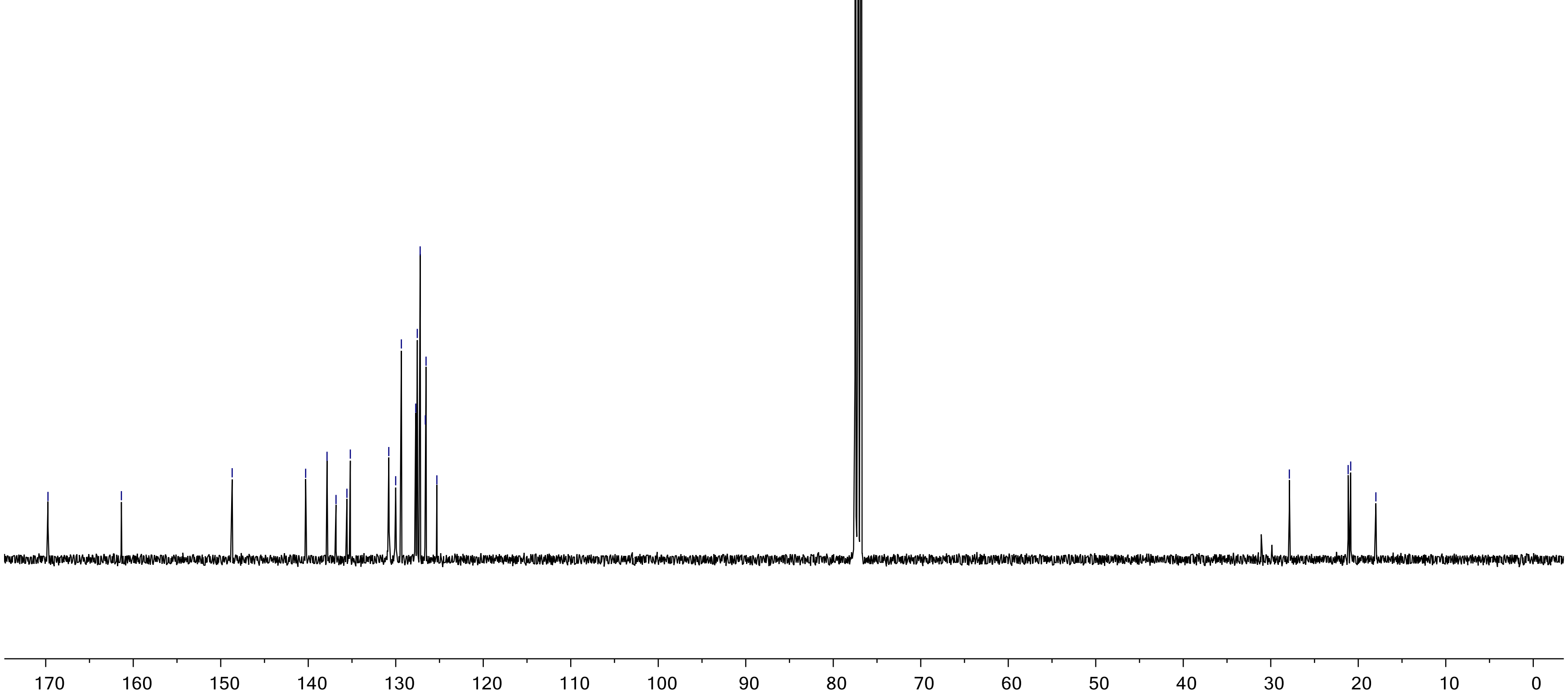

11. Chromatography data of enantioenriched compounds

***11.a** HPLC traces for the stoichiometric experiment*

*HPLC trace for compound **rac-3a***

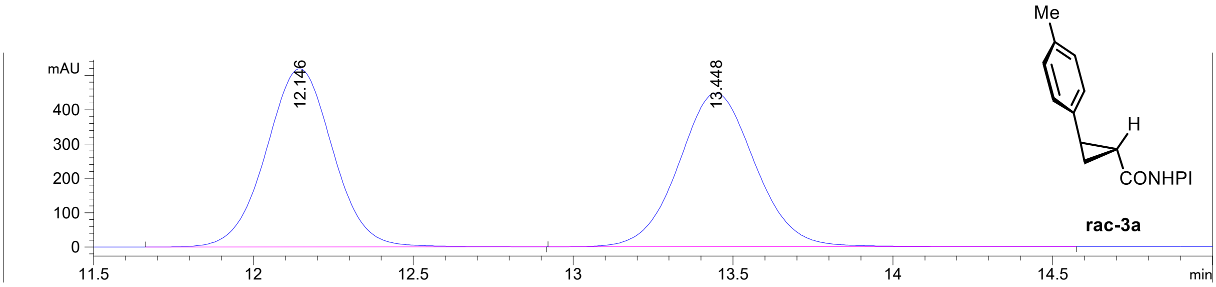

Signal 2: DAD1 B, Sig=210,8 Ref=360,100

Peak #	RetTime [min]	Type	Width [min]	Area [mAU*s]	Height [mAU]	Area %
1	12.146	BB	0.2253	7535.96436	519.23523	50.3129
2	13.448	BB	0.2560	7442.21826	447.60995	49.6871
Totals :				1.49782e4	966.84518	

*HPLC traces for compound **3a** (entry 2)*

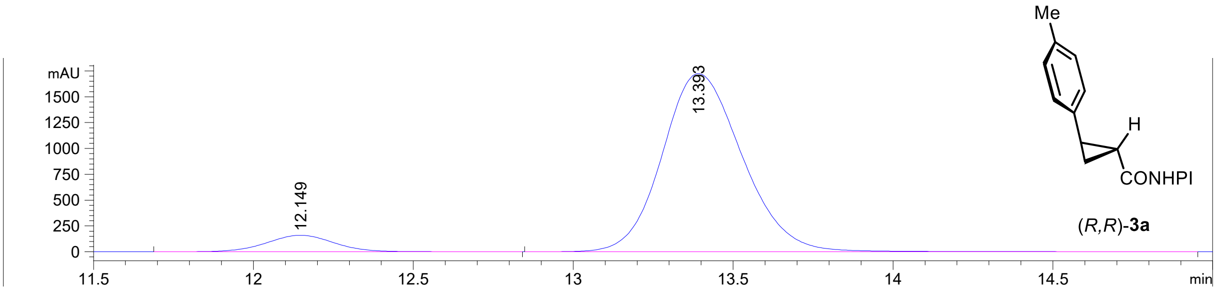

Signal 2: DAD1 B, Sig=210,8 Ref=360,100

Peak #	RetTime [min]	Type	Width [min]	Area [mAU*s]	Height [mAU]	Area %
1	12.149	BB	0.2283	2354.78589	159.41537	7.2747
2	13.393	BB	0.2672	3.00149e4	1723.41199	92.7253
Totals :				3.23697e4	1882.82736	

*HPLC traces for compound **3a** (entry 3)*

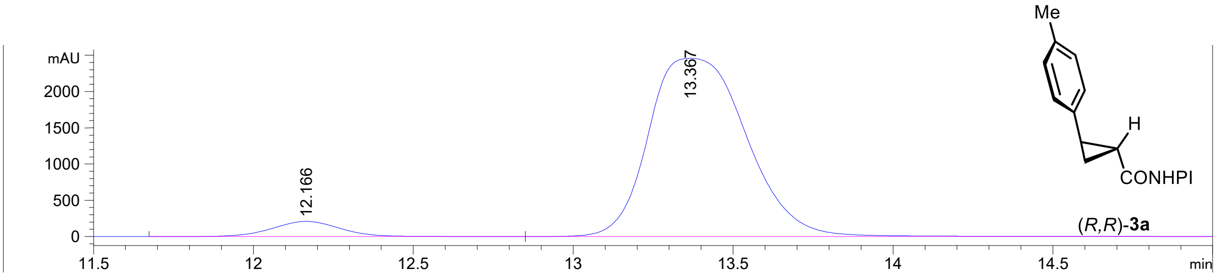

Signal 2: DAD1 B, Sig=210,8 Ref=360,100

Peak #	RetTime [min]	Type	Width [min]	Area [mAU*s]	Height [mAU]	Area %
1	12.166	BV	0.2287	3089.99707	208.78897	5.5833
2	13.367	VB	0.3340	5.22531e4	2458.10400	94.4167

Totals : 5.53431e4 2666.89297

*HPLC traces for compound **3a** (entry 4)*

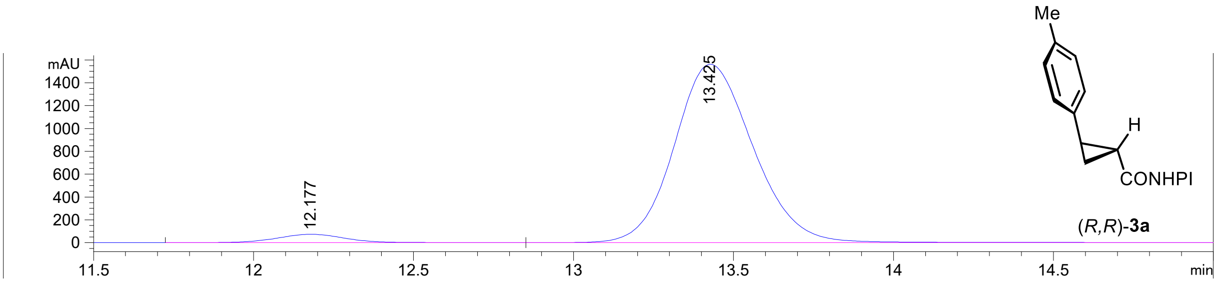

Signal 2: DAD1 B, Sig=210,8 Ref=360,100

Peak #	RetTime [min]	Type	Width [min]	Area [mAU*s]	Height [mAU]	Area %
1	12.177	BB	0.2258	1070.72693	72.69513	3.8356
2	13.425	BB	0.2644	2.68447e4	1562.78271	96.1644

Totals : 2.79154e4 1635.47784

*HPLC traces for compound **3a** (entry 5)*

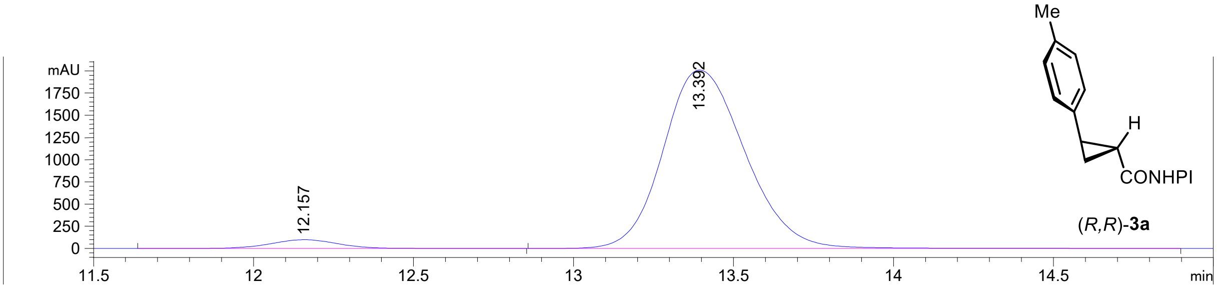

Signal 2: DAD1 B, Sig=210,8 Ref=360,100

Peak #	RetTime [min]	Type	Width [min]	Area [mAU*s]	Height [mAU]	Area %
1	12.157	BB	0.2259	1450.21570	98.39658	3.8947
2	13.392	BB	0.2757	3.57855e4	2010.46411	96.1053
Totals :				3.72357e4	2108.86069	

Analysis Conditions: Daicel Chiralpak-IA column (0.46 cm internal diameter x 25cm), temperature 25°C, eluent 90:10 Hexane:*i*-PrOH, flow rate 1 mL/min, λ: 210 nm.

11.b HPLC traces for compound ***3b***

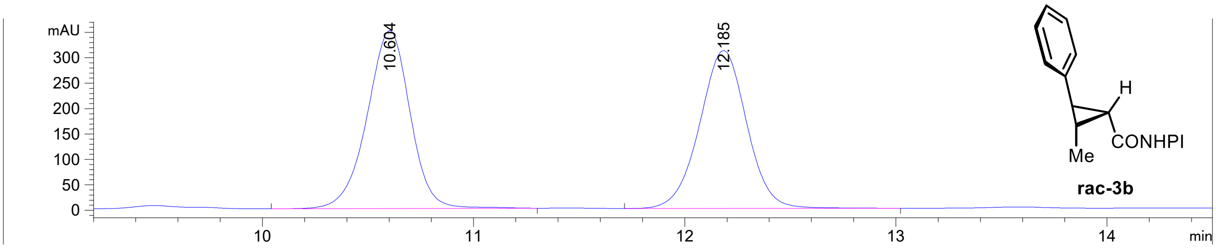

Signal 2: DAD1 B, Sig=210,8 Ref=360,100

Peak #	RetTime [min]	Type	Width [min]	Area [mAU*s]	Height [mAU]	Area %
1	10.604	BB	0.2169	4948.94580	350.15945	50.6801
2	12.185	BB	0.2369	4816.11377	310.65314	49.3199
Totals :				9765.05957	660.81259	

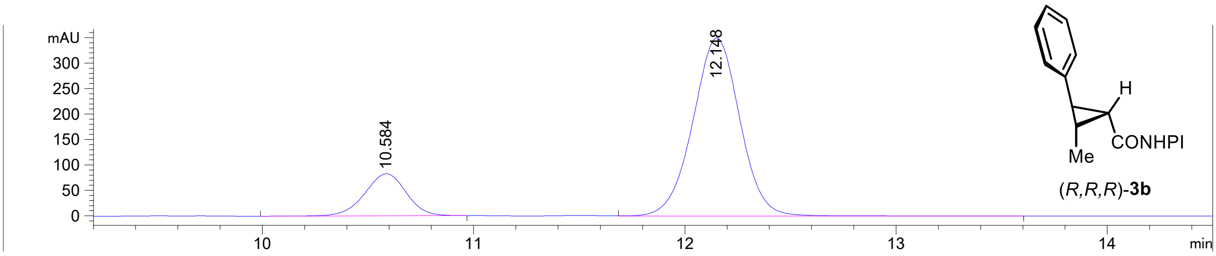

Signal 2: DAD1 B, Sig=210,8 Ref=360,100

Peak #	RetTime [min]	Type	Width [min]	Area [mAU*s]	Height [mAU]	Area %
1	10.584	BB	0.2097	1132.26587	82.73400	17.2877
2	12.148	VB	0.2386	5417.28418	350.00470	82.7123
Totals :				6549.55005	432.73870	

Analysis Conditions: Daicel Chiralpak-IA column (0.46 cm internal diameter x 25cm), temperature 25°C, eluent 90:10 Hexane:*i*-PrOH, flow rate 1 mL/min, λ: 210 nm.

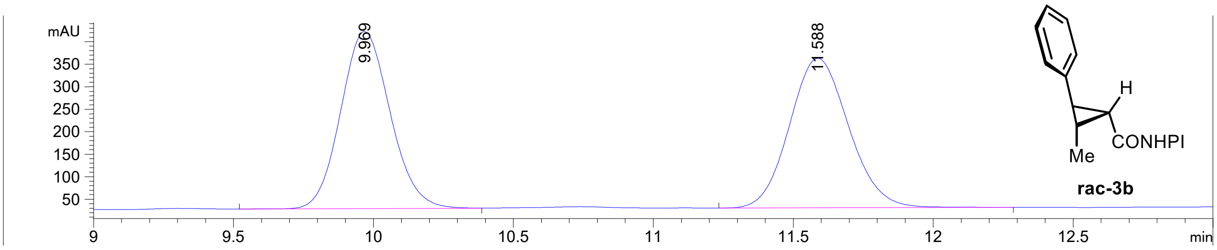

Signal 2: DAD1 B, Sig=210,8 Ref=360,100

Peak #	RetTime [min]	Type	Width [min]	Area [mAU*s]	Height [mAU]	Area %
1	9.969	BV	0.1894	4842.22363	393.94278	49.7638
2	11.588	BB	0.2296	4888.18848	332.28479	50.2362

Totals : 9730.41211 726.22757

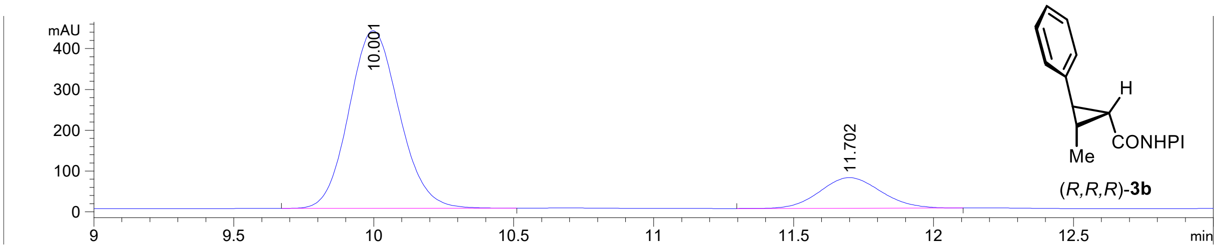

Signal 2: DAD1 B, Sig=210,8 Ref=360,100

Peak #	RetTime [min]	Type	Width [min]	Area [mAU*s]	Height [mAU]	Area %
1	10.001	BB	0.1936	5432.33496	435.21506	82.7310
2	11.702	BB	0.2344	1133.92542	75.01069	17.2690

Totals : 6566.26038 510.22575

Analysis Conditions: Daicel Chiralpak-IC column (0.46 cm internal diameter x 25cm), temperature 25°C, eluent 90:10 Hexane:*i*-PrOH, flow rate 1 mL/min, λ: 210 nm.

11.c HPLC traces for compound ***3c***

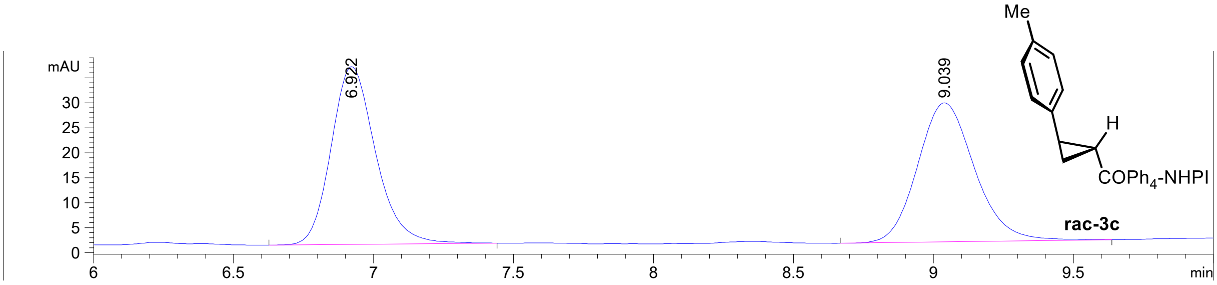

Signal 2: DAD1 B, Sig=210,8 Ref=360,100

Peak #	RetTime [min]	Type	Width [min]	Area [mAU*s]	Height [mAU]	Area %
1	6.922	BB	0.1689	393.21417	35.57966	49.9324
2	9.039	BB	0.2172	394.27890	27.84155	50.0676
Totals :				787.49307	63.42121	

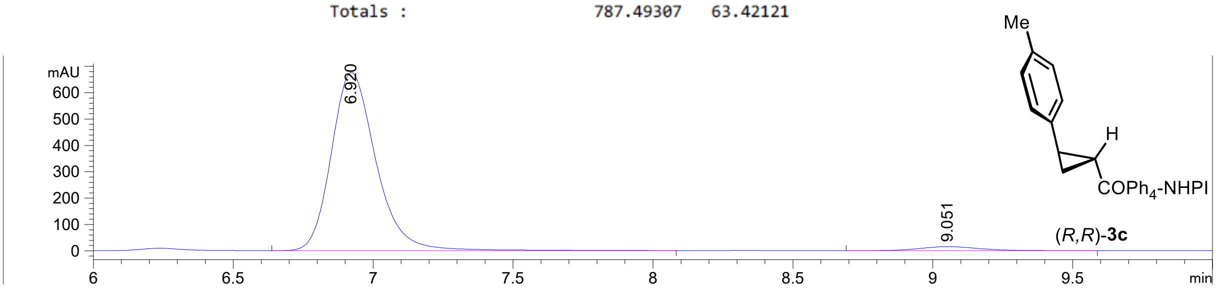

Signal 2: DAD1 B, Sig=210,8 Ref=360,100

Peak #	RetTime [min]	Type	Width [min]	Area [mAU*s]	Height [mAU]	Area %
1	6.920	BB	0.1691	7488.30518	676.62408	97.1879
2	9.051	BB	0.2239	216.67409	14.88134	2.8121
Totals :				7704.97926	691.50543	

Analysis Conditions: Daicel Chiralpak-IF column (0.46 cm internal diameter x 25cm), temperature 25°C, eluent 80:20 Hexane:*i*-PrOH, flow rate 1 mL/min, λ: 210 nm.

*11.d HPLC traces for compound **3b** from the sequential reaction*

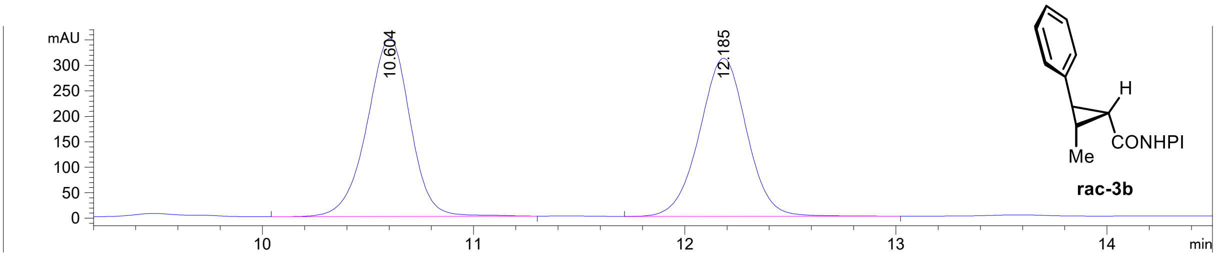

Signal 2: DAD1 B, Sig=210,8 Ref=360,100

Peak #	RetTime [min]	Type	Width [min]	Area [mAU*s]	Height [mAU]	Area %
1	10.604	BB	0.2169	4948.94580	350.15945	50.6801
2	12.185	BB	0.2369	4816.11377	310.65314	49.3199

Totals : 9765.05957 660.81259

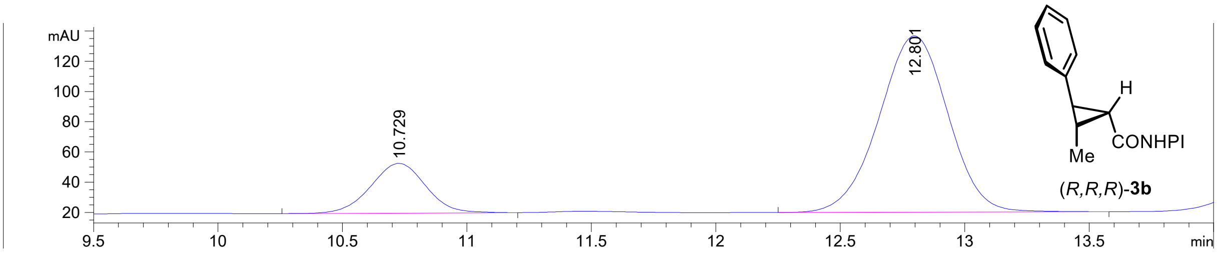

Signal 2: DAD1 B, Sig=210,8 Ref=360,100

Peak #	RetTime [min]	Type	Width [min]	Area [mAU*s]	Height [mAU]	Area %
1	10.729	BB	0.2373	505.55957	32.89753	18.3810
2	12.801	BB	0.2986	2244.89136	116.60703	81.6190

Totals : 2750.45093 149.50455

Analysis Conditions: Daicel Chiralpak-IA column (0.46 cm internal diameter x 25cm), temperature 25°C, eluent 90:10 Hexane:*i*-PrOH, flow rate 1 mL/min, λ: 210 nm.

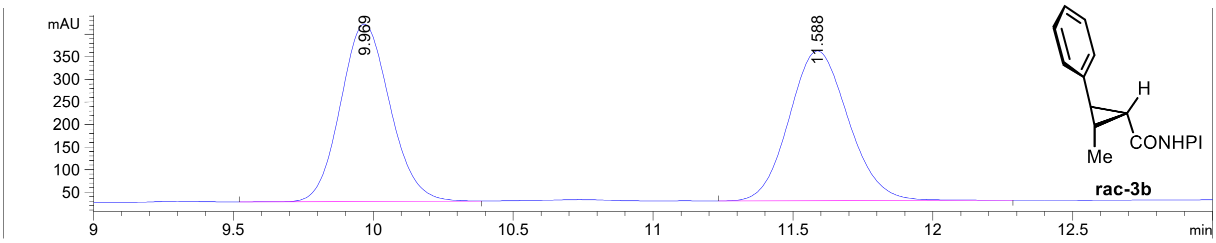

Signal 2: DAD1 B, Sig=210,8 Ref=360,100

Peak #	RetTime [min]	Type	Width [min]	Area [mAU*s]	Height [mAU]	Area %
1	9.969	BV	0.1894	4842.22363	393.94278	49.7638
2	11.588	BB	0.2296	4888.18848	332.28479	50.2362

Totals : 9730.41211 726.22757

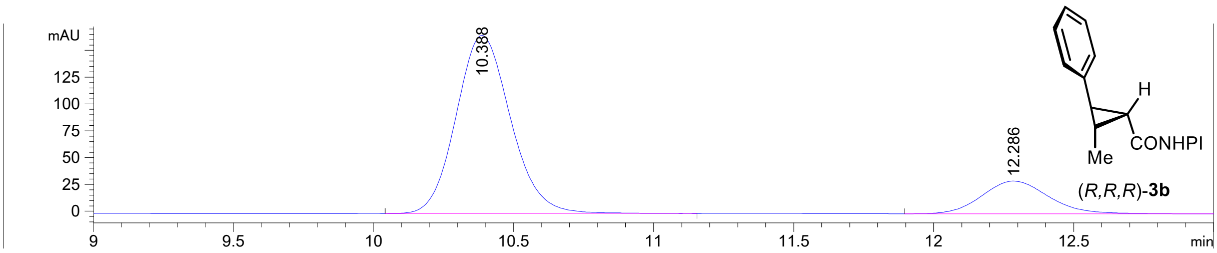

Signal 2: DAD1 B, Sig=210,8 Ref=360,100

Peak #	RetTime [min]	Type	Width [min]	Area [mAU*s]	Height [mAU]	Area %
1	10.388	BB	0.2076	2243.48926	166.09262	81.6265
2	12.286	BB	0.2573	504.99368	30.47677	18.3735

Totals : 2748.48294 196.56939

Analysis Conditions: Daicel Chiralpak-IC column (0.46 cm internal diameter x 25cm), temperature 25°C, eluent 90:10 Hexane:*i*-PrOH, flow rate 1 mL/min, λ: 210 nm.

12. References

1. Montesinos-Magraner, M.; Costantini, M.; Ramírez-Contreras, R.; Muratore, M. E.; Johansson, M. J.; Mendoza, A., General Cyclopropane Assembly by Enantioselective Transfer of a Redox-Active Carbene to Aliphatic Olefins. *Angewandte Chemie International Edition* **2019,** *58* (18), 5930-5935.
2. Chanthamath, S.; Phomkeona, K.; Shibatomi, K.; Iwasa, S., Highly stereoselective Ru(ii)–Pheox catalyzed asymmetric cyclopropanation of terminal olefins with succinimidyl diazoacetate. *Chemical Communications* **2012,** *48* (62), 7750-7752.
3. Nechab, M.; Einhorn, C.; Einhorn, J., New aerobic oxidation of benzylic compounds: efficient catalysis by N-hydroxy-3,4,5,6-tetraphenylphthalimide (NHTPPI)/CuCl under mild conditions and low catalyst loading. *Chemical Communications* **2004,** (13), 1500-1501.
4. Bug, T.; Hartnagel, M.; Schlierf, C.; Mayr, H., How Nucleophilic Are Diazo Compounds? *Chemistry – A European Journal* **2003,** *9* (17), 4068-4076.
5. Burés, J., A Simple Graphical Method to Determine the Order in Catalyst. *Angewandte Chemie International Edition* **2016,** *55* (6), 2028-2031.
6. Burés, J., Variable Time Normalization Analysis: General Graphical Elucidation of Reaction Orders from Concentration Profiles. *Angewandte Chemie International Edition* **2016,** *55* (52), 16084-16087.
7. Nielsen, C. D. T.; Burés, J., Visual kinetic analysis. *Chemical Science* **2019,** *10* (2), 348-353.
