# Supplementary material for: Combined Experimental and Computational Study of Ruthenium N-Hydroxyphthalimidoyl Carbenes in Alkene Cyclopropanation Reactions
Source: ACS Catal. 2021 Aug 18;11(17):10950–63. doi: 10.1021/acscatal.1c02540 (PMC8419840; doi:10.1021/acscatal.1c02540)
Supplement: Supplementary file 2 — cs1c02540_si_002.pdf [file cs1c02540_si_002.pdf]

Supporting Information
Computational part

Combined Experimental and Computational Study of Ruthenium *N*-Hydroxyphthalimidoyl Carbenes in Alkene Cyclopropanation Reactions

Ferran Planas[†], Matteo Costantini[†], Marc Montesinos-Magraner,
Fahmi Himo, Abraham Mendoza**

Department of Organic Chemistry, Arrhenius Laboratory, Stockholm University,
SE-106 91 Stockholm, Sweden.
[†]The authors contributed equally to the work.

Corresponding authors email: fahmi.himo@su.se; abraham.mendoza@su.se

Table of Contents

13. Calculated energies and energy corrections. *S39*

13.1. Determination of the most stable species in solution. S40

13.2. Carbene formation step. S41

13.3 Carbene isomerization. S42

13.4. Side-reactions. S42

13.5. Cyclopropanation reactions. S43

13.6. Reactivity of the migratory-insertion product. S45

14. Optimized geometries of the selectivity-determining transition states at the two apical positions. *S46*

15. Cartesian coordinates. *S53*

13. Calculated energies and energy corrections.

In Tables S3-S12, the reported values are defined as follows:

- **E_{el}**: Electronic energy calculated with the B3LYP-D3(BJ) functional using the 6-31G(d,p) basis set for all non-metal atoms, and LANL2DZ for ruthenium.

- **E_{solv}**: Single-point solvation energy calculated using the PCM method at the same level of theory as the geometry optimization.

- **E_{bb}**: Single-point large-basis set electronic energy, calculated with the 6-311+G(2d,2p) basis set for all non-metal atoms, and LANL2TZ for ruthenium.

- **G_{corr}**: Thermal correction to Gibbs free energy calculated at the same level of theory as the geometry optimization, and using the RRHO approximation as implemented in Gaussian 16.

- The total energy is calculated using **$E_{tot} = E_{bb} + (E_{solv} – E_{el}) + G_{corr} + SS_{corr}$** where **$SS_{corr}$** is the standard state correction amounting to +1.9 kcal/mol for all solutes, which is added to the energies of all species except in the case of the dichloromethane solvent (correction of +4.9 kcal/mol), and the gas molecule N_2 (no correction added).

- **E_{rel}**: The energy of the various species relative to Ru-Pheox$(AN)_4$

In Tables S5-S12, the energies for the **Ap_{syn}**, **Ap_{anti}** and **Eq_{trans}** positions are reported, and the energies for the **Eq_{cis}** position are left out, as formation of the carbene in this position is unfavorable.

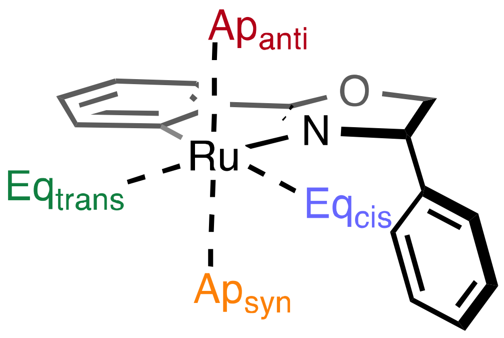

Figure S17. *Representation of the four positions at the Ru-Pheox complex.*

13.1. Determination of the most stable species in solution.

Table S3. Calculated energies and energy corrections for the different species that can form from the starting materials.

Species	Position of propene	E_{el} (a.u.)	E_{solv} (a.u.)	E_{bb} (a.u.)	G_{corr} (a.u.)	E_{rel} (kcal/mol)
**[Ru(Pheox)(AN)$_4$]**	-	-1333.77725	-1333.82998	-1334.14483	0.35328	0.0
**NHPI-DA**	-	-849.18112	-849.19216	-849.45201	0.10187	-
**Acetonitrile (AN)**	-	-132.76040	-132.76709	-132.80497	0.02144	-
**Propene (Prop)**	-	-117.92205	-117.92300	-117.95940	0.05486	-
**[Ru(Pheox)(Prop)(AN)$_3$]**	Ap_{anti}	-1318.93161	-1318.98420	-1319.28912	0.39330	7.5
	Ap_{syn}	-1318.93059	-1318.98211	-1319.28809	0.39205	7.0
	Eq_{cis}	-1318.93498	-1318.98624	-1319.29370	0.39054	3.2
	Eq_{trans}	-1318.93303	-1318.98513	-1319.29186	0.39470	6.4
**[Ru(Pheox)(Prop)$_2$(AN)$_2$]**	Ap_{anti}<br>Ap_{syn}	-1304.08709	-1304.13870	-1304.43449	0.43219	13.1
	Eq_{cis}<br>Eq_{trans}	-1304.08090	-1304.13141	-1304.42924	0.43096	16.3
	Ap_{anti}<br>Eq_{cis}	-1304.08209	-1304.13327	-1304.43024	0.43073	15.1
	Ap_{syn}<br>Eq_{cis}	-1304.08356	-1304.13444	-1304.43162	0.43038	14.2
**[Ru(Pheox)(DCM)(AN)$_3$]**	Eq_{cis}	-2160.69893	-2160.75185	-2161.10683	0.33686	4.8
**[Ru(Pheox)(DA-O)(AN)$_3$] Coordination via: Carbonyl-oxygen**	Ap_{anti}	-2050.19561	-2050.25282	-2050.78266	0.43930	9.2
	Eq_{cis}	-2050.20003	-2050.25901	-2050.78880	0.43843	3.7
**[Ru(Pheox)(DA-O)(AN)$_3$] Coordination via: Phthalimidoyl-oxygen**	Ap_{anti}	-2050.20560	-2050.25985	-2050.79148	0.44162	7.0
	Eq_{cis}	-2050.20003	-2050.26101	-2050.79080	0.43839	1.2

13.2. Carbene formation step.

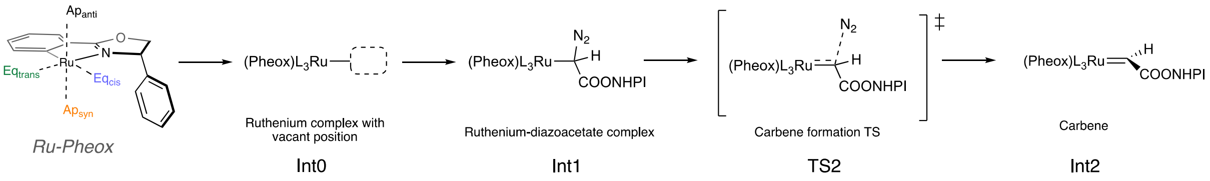

Figure S18. *Steps of the reaction mechanism leading to the formation of the carbene intermediate.*

Table S4. Calculated energies and energy corrections for the intermediates and transition state that form between the initial complex and the carbene intermediate.

Species		E_{el} (a.u.)	E_{solv} (a.u.)	E_{bb} (a.u.)	G_{corr} (a.u.)	E_{rel} (kcal/mol)
**RuPheox**		-1333.77725	-1333.82998	-1334.14483	0.35328	0.0
**Int0**	Ap_{anti}	-1200.96098	-1201.01846	-1201.28629	0.31099	16.2
	Ap_{syn}	-1200.96089	-1201.02010	-1201.28561	0.31357	15.2
	Eq_{cis}	-1200.98279	-1201.03765	-1201.30749	0.31298	4.8
	Eq_{trans}	-1200.96414	-1201.02239	-1201.28845	0.31338	14.9
**Int1**	Ap_{anti}	-2050.20188	-2050.20188	-2050.78904	0.44297	10.1
	Ap_{syn}	-2050.19913	-2050.25269	-2050.78667	0.44303	11.3
	Eq_{cis}	-2050.20628	-2 050.25880	-2050.79436	0.44047	5.5
	Eq_{trans}	-2050.19541	-2 050.24913	-2050.78342	0.44002	11.4
**TS2**	Ap_{anti}	-2050.19384	-2050.24592	-2050.78117	0.44079	14.3
	Ap_{syn}	-2050.19062	-2050.24403	-2050.77900	0.44133	15.1
	Eq_{cis}	-2050.18202	-2050.23418	-2050.77261	0.44204	20.4
	Eq_{trans}	-2050.18462	-2050.23748	-2050.77379	0.44032	18.1
**Int2**	Ap_{anti}	-1940.69395	-1940.74899	-1941.24717	0.43127	-20.5
	Ap_{syn}	-1940.69281	-1940.74723	-1941.24577	0.43080	-19.5
	Eq_{cis}	-1940.67184	-1940.72513	-1941.22780	0.43461	-5.1
	Eq_{trans}	-1940.69326	-1940.74721	-1941.24865	0.43118	-20.8

13.3 Carbene isomerization.

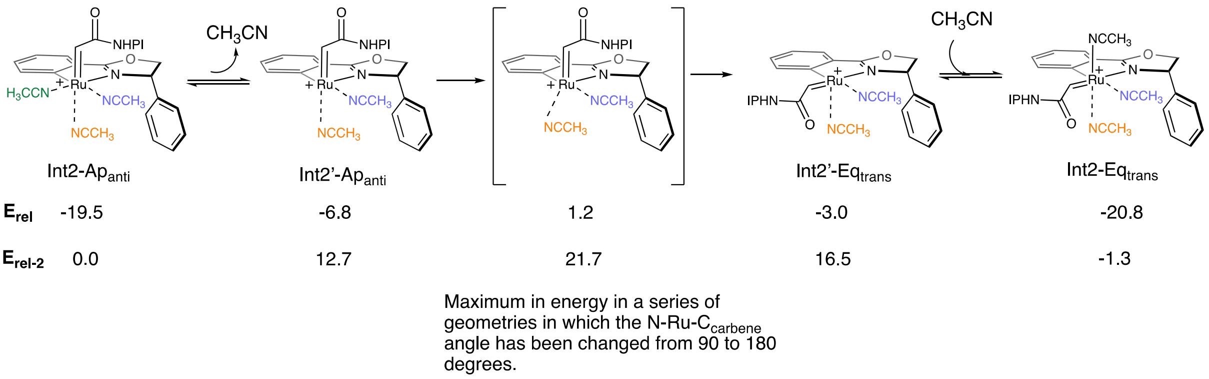

Figure S19. *Isomerization mechanism of* ***Int2-Ap$_{anti}$*** *to* ***Int2-Eq$_{trans}$.*** **E$_{rel-2}$** the energies relative to **Int2-Ap$_{anti}$**.

13.4. Side-reactions.

Table S5. Calculated energies and energy corrections for the side reactions at each carbene position, starting from the carbene intermediates. **E$_{rel-2}$** indicates the energies relative to each of the three considered carbene complexes.

Species	E$_{el}$ (a.u.)	E$_{solv}$ (a.u.)	E$_{bb}$ (a.u.)	G$_{corr}$ (a.u.)	E$_{rel}$ (kcal/mol)	E$_{rel-2}$ (kcal/mol)
**Int2-Ap$_{anti}$**	-1940.69395	-1940.74899	-1941.24717	0.43127	-20.5	**0.0**
**Int3-DM-Ap$_{anti}$**	-2657.13562	-2657.18963	-2657.90730	0.52065	-19.7	**0.8**
**TS-DM-Ap$_{anti}$**	-2657.11668	-2657.17228	-2657.88669	0.52349	-6.0	**14.5**
**Product-DM**	-2657.23090	-2657.28554	-2658.00493	0.52140	-80.9	**-60.4**
**TS3-MI-Ap$_{anti}$**	-1940.68083	-1940.73553	-1941.23417	0.43097	-12.3	**8.2**
**Int3-MI-Ap$_{anti}$**	-2073.55977	-2073.61361	-2074.15337	0.48138	-63.0	**-42.5**
**Int2-Ap$_{syn}$**	-1940.69281	-1940.74723	-1941.24577	0.43080	-19.5	**0.0**
**Int3-DM-Ap$_{syn}$**	-2657.12413	-2657.17909	-2657.89681	0.51779	-15.5	**4.0**
**TS-DM-Ap$_{syn}$**	-2657.11412	-2657.16864	-2657.88462	0.52247	-4.7	**14.8**
**Product-DM**	-2547.69897	-2547.75403	-2548.43522	0.51738	-88.2	**-69.7**
**TS3-MI-Ap$_{syn}$**	-1940.68303	-1940.73591	-1941.23625	0.43309	-11.1	**8.4**
**Int3-MI-Ap$_{syn}$**	-2073.56149	-2073.61490	-2074.15381	0.48110	-63.1	**-43.6**
**Int2-Eq$_{trans}$**	-1940.69326	-1940.74721	-1941.24865	0.43118	-20.8	**0.0**
**Int3-DM-Eq$_{trans}$**	-2657.12298	-2657.18062	-2657.89595	0.52234	-13.8	**7.0**
**TS-DM-Eq$_{trans}$**	-2657.10672	-2657.16207	-2657.87831	0.52386	-0.4	**20.4**
**Product-DM**	-2547.69880	-2547.75427	-2548.43533	0.52030	-86.7	**-65.9**
**TS3-MI-Eq$_{trans}$**	-1940.64671	-1940.70175	-1941.20069	0.43498	11.0	**31.8**

13.5. Cyclopropanation reactions.

In tables S6-S11 the $\mathbf{E_{rel-2}}$ value indicates the energies relative to each of the three considered carbene complexes.

Table S6. Calculated energies and energy corrections for the inner-sphere and outer-sphere mechanisms for the propene substrate at the Ap_{anti} position.

Species	E_{el} (a.u.)	E_{solv} (a.u.)	E_{bb} (a.u.)	G_{corr} (a.u.)	E_{rel} (kcal/mol)	E_{rel-2} (kcal/mol)
**Int2-Ap$_{anti}$**	-1940.69395	-1940.74899	-1941.24717	0.43127	-20.5	0.0
**Int2-Ap$_{anti}$-DCM**	-2767.61470	-2767.66986	-2768.20745	0.41523	-14.4	6.1
**Int3-IS-Ap$_{anti}$**	-1925.85210	-1925.90654	-1926.39468	0.46938	-16.4	4.1
**TS4-IS-Ap$_{anti}$**	-1925.8460	-1925.90066	-1926.38812	0.47257	-10.5	10.0
**Int4-IS -Ap$_{anti}$**	-1925.85672	-1925.91173	-1926.39769	0.47439	-15.5	5.0
**TS5-IS-Ap$_{anti}$**	-1925.84946	-1925.90400	-1926.38836	0.4800	-5.8	14.7
**Product**	-1925.90056	-1925.95315	-1926.43339	0.48032	-32.7	-12.2
**TS-OS-Ap$_{anti}$**	-2058.63194	-2058.68304	-2059.21457	0.51152	-8.4	12.1
**Product**	-2058.68237	-2058.73263	-2059.26155	0.51710	-33.9	-13.4

Table S7. Calculated energies and energy corrections for the inner-sphere and outer-sphere mechanisms for the styrene substrate at the $\mathbf{Ap_{anti}}$ position.

Species	E_{el} (a.u.)	E_{solv} (a.u.)	E_{bb} (a.u.)	G_{corr} (a.u.)	E_{rel} (kcal/mol)	E_{rel-2} (kcal/mol)
**Int2-Ap$_{anti}$**	-1940.69395	-1940.74899	-1941.24717	0.43127	-20.5	0.0
**Int3-IS-Ap$_{anti}$**	-2117.62022	-2117.67443	-2118.21256	0.52106	-16.4	4.1
**TS4-IS-Ap$_{anti}$**	-2117.61849	-2117.67246	-2118.20948	0.52353	-12.8	7.7
**Int4-IS -Ap$_{anti}$**	-2117.63031	-2117.68341	-2118.22033	0.52622	-17.4	3.1
**TS5-IS-Ap$_{anti}$**	-2117.61667	-2117.67378	-2118.20603	0.53006	-8.5	12.0
**Product**	-2117.66282	-2117.71791	-2118.24637	0.53028	-32.4	-11.9
**TS-retro-[2+2]**	-2117.60309	-2117.65736	-2118.19852	0.52400	-5.8	14.7
**TS-OS-Ap$_{anti}$**	-2250.40087	-2250.45173	-2251.03565	0.55672	-14.5	6.0
**Product**	-2250.44396	-2250.49517	-2251.07430	0.56588	-33.2	-11.7

Table S8. Calculated energies and energy corrections for the inner-sphere and outer-sphere mechanisms for the propene substrate at the $\mathbf{Ap_{syn}}$ position.

Species	E_{el} (a.u.)	E_{solv} (a.u.)	E_{bb} (a.u.)	G_{corr} (a.u.)	E_{rel} (kcal/mol)	E_{rel-2} (kcal/mol)
**Int2-Ap$_{syn}$**	-1940.69281	-1940.74723	-1941.24577	0.43080	-19.5	0.0
**Int3-IS-Ap$_{syn}$**	-1925.85318	-1925.90515	-1926.39567	0.46895	-15.8	3.7
**TS4-IS-Ap$_{syn}$**	-1925.84301	-1925.89513	-1926.38576	0.47206	-7.7	11.8
**Int4-IS-Ap$_{syn}$**	-1925.85298	-1925.90557	-1926.39478	0.47331	-12.9	6.6
**TS5-IS-Ap$_{syn}$**	-1925.84456	-1925.89932	-1926.38334	0.48036	-2.6	16.9
**Product**	-1925.89422	-1925.94727	-1926.42696	0.48047	-28.9	-9.4
**TS-OS-Ap$_{syn}$**	-2058.63010	-2058.67984	-2059.21205	0.51167	-5.9	13.6
**Product**	-2058.67823	-2058.72825	-2059.25677	0.51678	-30.9	-11.4

Table S9. Calculated energies and energy corrections for the inner-sphere and outer-sphere mechanisms for the styrene substrate at the **Ap_{syn}** position.

Species	E_{el} (a.u.)	E_{solv} (a.u.)	E_{bb} (a.u.)	G_{corr} (a.u.)	E_{rel} (kcal/mol)	E_{rel-2} (kcal/mol)
**Int2-Ap_{syn}**	-1940.69281	-1940.74723	-1941.24577	0.43080	-19.5	0.0
**Int3-IS-Ap_{syn}**	-2117.62261	-2117.67423	-2118.21545	0.52112	-16.6	2.9
**TS4-IS-Ap_{syn}**	-2117.60758	-2117.65963	-2118.20064	0.52250	-6.7	12.8
**Int4-IS-Ap_{syn}**	-2117.62335	-2117.67635	-2118.21581	0.52428	-15.7	3.8
**TS5-IS-Ap_{syn}**	-2117.61603	-2117.66976	-2118.20474	0.53122	-4.8	14.7
**Product**	-2117.66679	-2117.71897	-2118.25180	0.52714	-36.0	-16.5
**TS-OS-Ap_{syn}**	-2250.40702	-2250.45570	-2251.03931	0.56060	-13.0	6.5
**Product**	-2250.44700	-2250.49580	-2251.07593	0.56803	-31.4	-11.9

Table S10. Calculated energies and energy corrections for the inner-sphere and outer-sphere mechanisms for the propene substrate at the **Eq_{trans}** position.

Species	E_{el} (a.u.)	E_{solv} (a.u.)	E_{bb} (a.u.)	G_{corr} (a.u.)	E_{rel} (kcal/mol)	E_{rel-2} (kcal/mol)
**Int2-Eq_{trans}**	-1940.69326	-1940.74721	-1941.24865	0.43118	-20.8	0.0
**Int3-IS-Eq_{trans}**	-1925.85100	-1925.90351	-1926.39389	0.47143	-13.4	7.4
**TS4-IS-Eq_{trans}**	-1925.84337	-1925.89639	-1926.38703	0.47431	-7.6	13.2
**Int4-IS -Eq_{trans}**	-1925.85068	-1925.90456	-1926.39322	0.47517	-11.5	9.3
**TS5-IS-Eq_{trans}**	-1925.83720	-1925.89000	-1926.37901	0.47745	-0.5	20.3
**Product**	-1925.89376	-1925.94859	-1926.43040	0.47347	-36.5	-15.7
**TS-OS-Eq_{trans}**	-2058.62204	-2058.67513	-2059.20624	0.50981	-5.5	15.3
**Product**	-2058.67896	-2058.73071	-2059.25861	0.51822	-32.2	-11.4

Table S11. Calculated energies and energy corrections for the inner-sphere and outer-sphere mechanisms for the styrene substrate at the **Eq_{trans}** position.

Species	E_{el} (a.u.)	E_{solv} (a.u.)	E_{bb} (a.u.)	G_{corr} (a.u.)	E_{rel} (kcal/mol)	E_{rel-2} (kcal/mol)
**Int2-Eq_{trans}**	-1940.69326	-1940.74721	-1941.24865	0.43118	-20.8	0.0
**Int3-IS-Eq_{trans}**	-2117.62330	-2117.67319	-2118.21587	0.52185	-15.3	5.5
**TS4-IS-Eq_{trans}**	-2117.61128	-2117.66200	-2118.20405	0.52565	-6.0	14.8
**Int4-IS -Eq_{trans}**	-2117.61403	-2117.66553	-2118.20639	0.52599	-7.8	13.0
**TS5-IS-Eq_{trans}**	-2117.59371	-2117.64772	-2118.18771	0.52482	0.9	21.7
**Product**	-2117.65093	-2117.70028	-2118.23872	0.52386	-28.0	-7.2
**TS-OS-Eq_{trans}**	-2250.38980	-2250.44230	-2251.02628	0.55978	-7.7	13.1
**Product**	-2250.43861	-2250.48969	-2251.06973	0.56746	-29.3	-8.5

13.6. Reactivity of the migratory-insertion product.

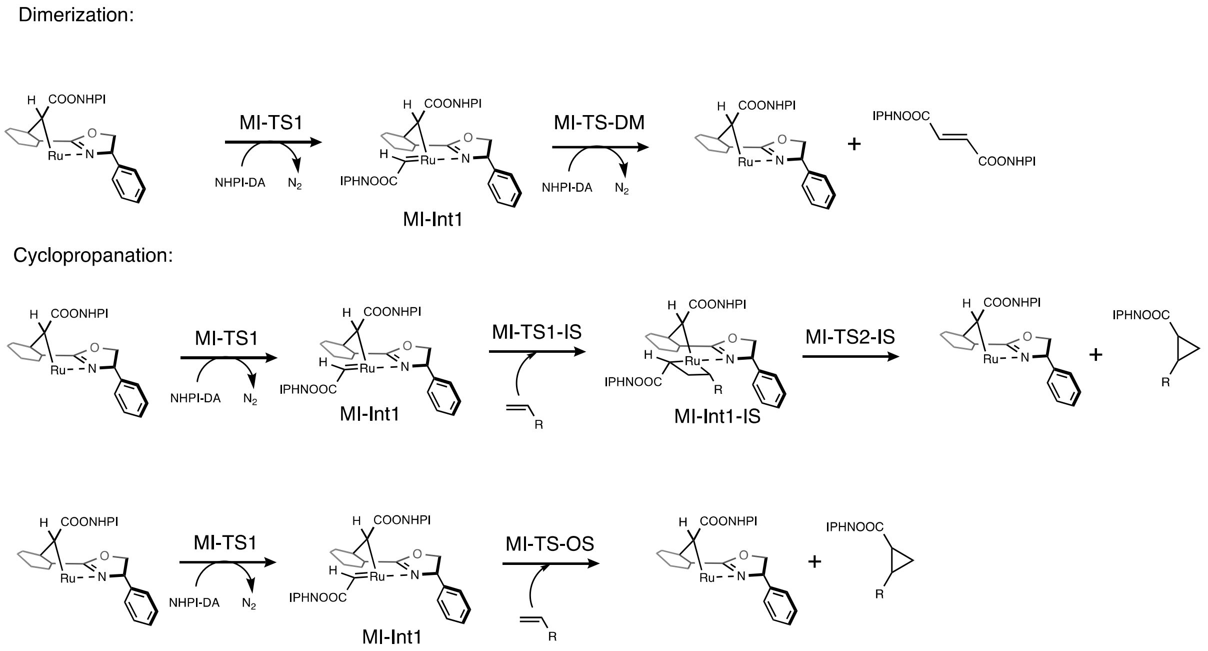

Figure S20. *Possible reactions that can take place starting from the migratory insertion product.*

Table S12. Calculated energies, energy corrections and relative energies for the cyclopropanation and dimerization reactions starting from the migratory insertion product. $\mathbf{E_{rel\text{-}2}}$ value indicates the relative energies relative to migratory insertion product **Int3-MI-Ap$_{anti}$**, and $\mathbf{E_{rel\text{-}3}}$ the relative energies relative to the carbene complex formed at the migratory insertion product.

Species	E_{el} (a.u.)	E_{solv} (a.u.)	E_{bb} (a.u.)	G_{corr} (a.u.)	E_{rel} (kcal/mol)	$E_{rel\text{-}2}$ (kcal/mol)	$E_{rel\text{-}3}$ (kcal/mol)
**Int3-MI-Ap$_{anti}$**	-2073.55977	-2073.61361	-2074.15337	0.48138	-63.0	**0.0**	
**MI-Ap$_{anti}$-TS1**	-2789.96827	-2790.02357	-2790.78368	0.56587	-48.1	**14.9**	
**MI-Ap$_{anti}$-Int1**	-2680.46869	-2680.52321	-2681.24850	0.55909	-78.1	**-15.1**	**0.0**
**MI-Ap$_{anti}$-TS1-IS**	-2857.38618	-2857.43893	-2 858.20553	0.64886	-68.2		**9.9**
**MI-Ap$_{anti}$-Int2-IS**	-2857.38964	-2857.44285	-2858.20745	0.64983	-69.1		**9.0**
**MI-Ap$_{anti}$-TS2-IS**	-2857.36978	-2857.42566	-2858.18820	0.64753	-60.1		**18.0**
**Product-IS**	-2857.42388	-2858.23698	-2857.47728	0.64730	-89.3		**-11.2**
**MI-Ap$_{anti}$-TS-OS**	-2990.17946	-2990.23037	-2991.03998	0.68727	-72.6		**5.5**
**Product-OS**	-2990.22425	-2990.27684	-2991.08092	0.69058	-97.2		**-19.1**
**MI- Ap$_{anti}$-TS-DM**	-3396.88041	-3396.93854	-3397.87672	0.64851	-60.2		**17.9**
**Product-DM**	-3287.48140	-3287.53796	-3288.44073	0.64720	-148.5		**-70.4**

14. Optimized geometries of the selectivity-determining transition states at the two apical positions.

The different geometries of the transition states leading to each diastereomer can be organized as follows:

- **Inner-sphere**: There are 8 different conformations of the reductive elimination transition state depending on (1) the relative orientation of the NHPI and the substituent of the olefin (cis or trans) and (2) which carbon of the olefin reacts with the carbene. Figure S5A is a schematic representation of the different conformations.

- **Outer-sphere**: The face of the complex (**Ap$_{anti}$**, in the example below) can be divided in 4 quadrants (Q1-Q4). The ester group of the carbene can be located in either of the quadrants, and adopt two different conformations (shown in red). Each of the 8 resulting carbene conformations can react with the styrene substrate in 4 different ways, depending on the face of each of the reacting molecules (see Figure S5B, bottom). That makes a total of 36 different transition states (8 per diastereomer).

This is the total number of searched transition states. However, in some cases the geometry optimization resulted in the formation of the preceding intermediate, or in the formation of a different conformation. In Figures S6-S10, all successfully optimized TS geometries for the **Ap$_{anti}$** and **Ap$_{syn}$** position are shown.

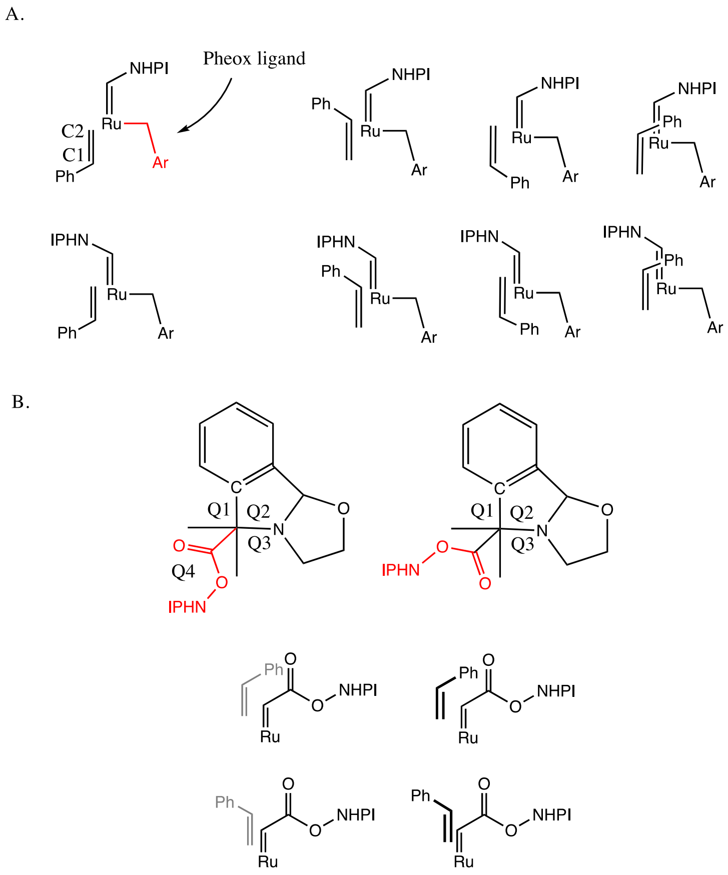

Figure S21. *Representation of the different TS conformations for the cyclopropanation reaction.*

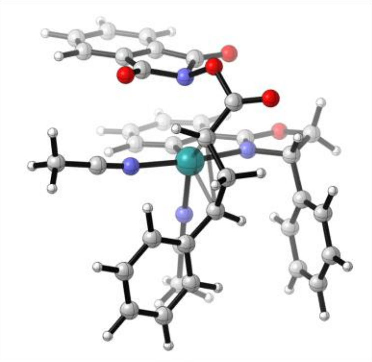

(*R,R*): +1.1

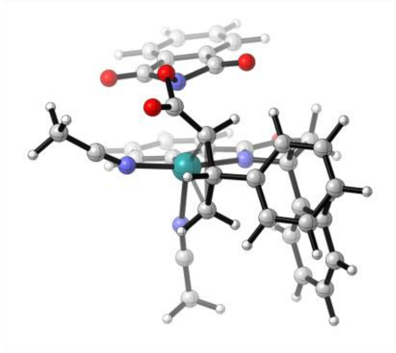

(*R,R*)': +5.7

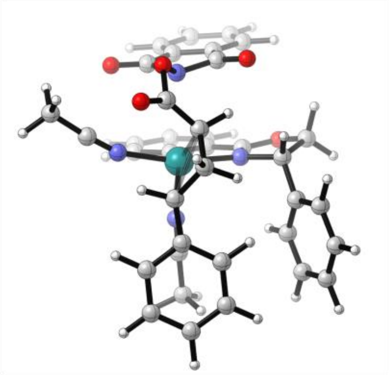

(*S,S*): +8.5

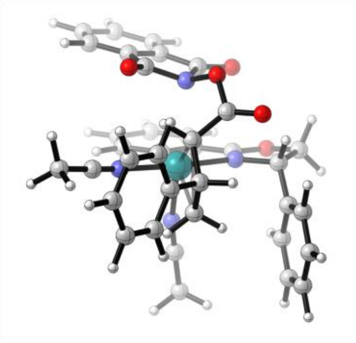

(*S,S*)': +4.4

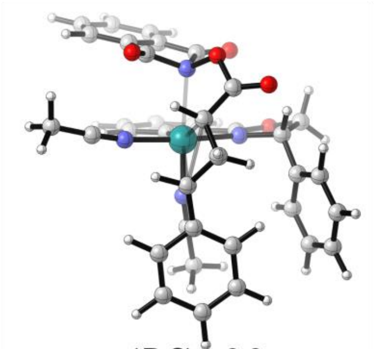

(*R,S*): +8.8

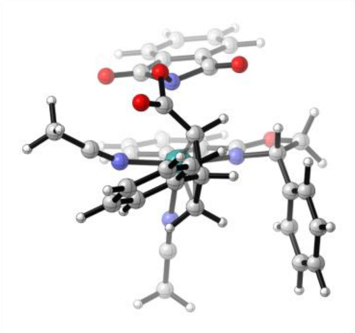

(*R,S*)': +7.0

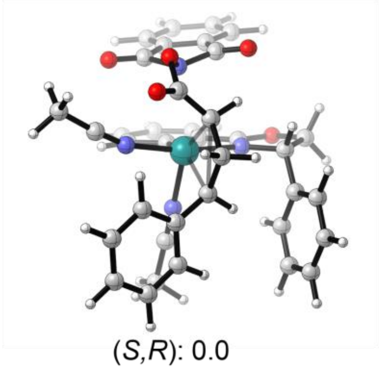

(*S,R*): 0.0

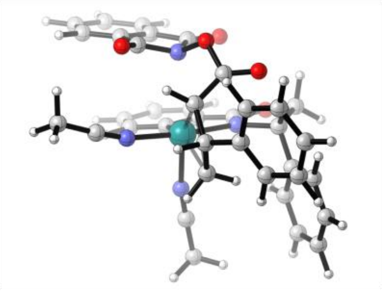

(*S,R*)': +6.6

Figure S22. *Different inner-sphere reductive-elimination TSs at the* ***Ap_{anti} position****. The values indicate the energy of each TS relative to the most stable reductive-elimination* ***Ap_{anti}-TS****.*

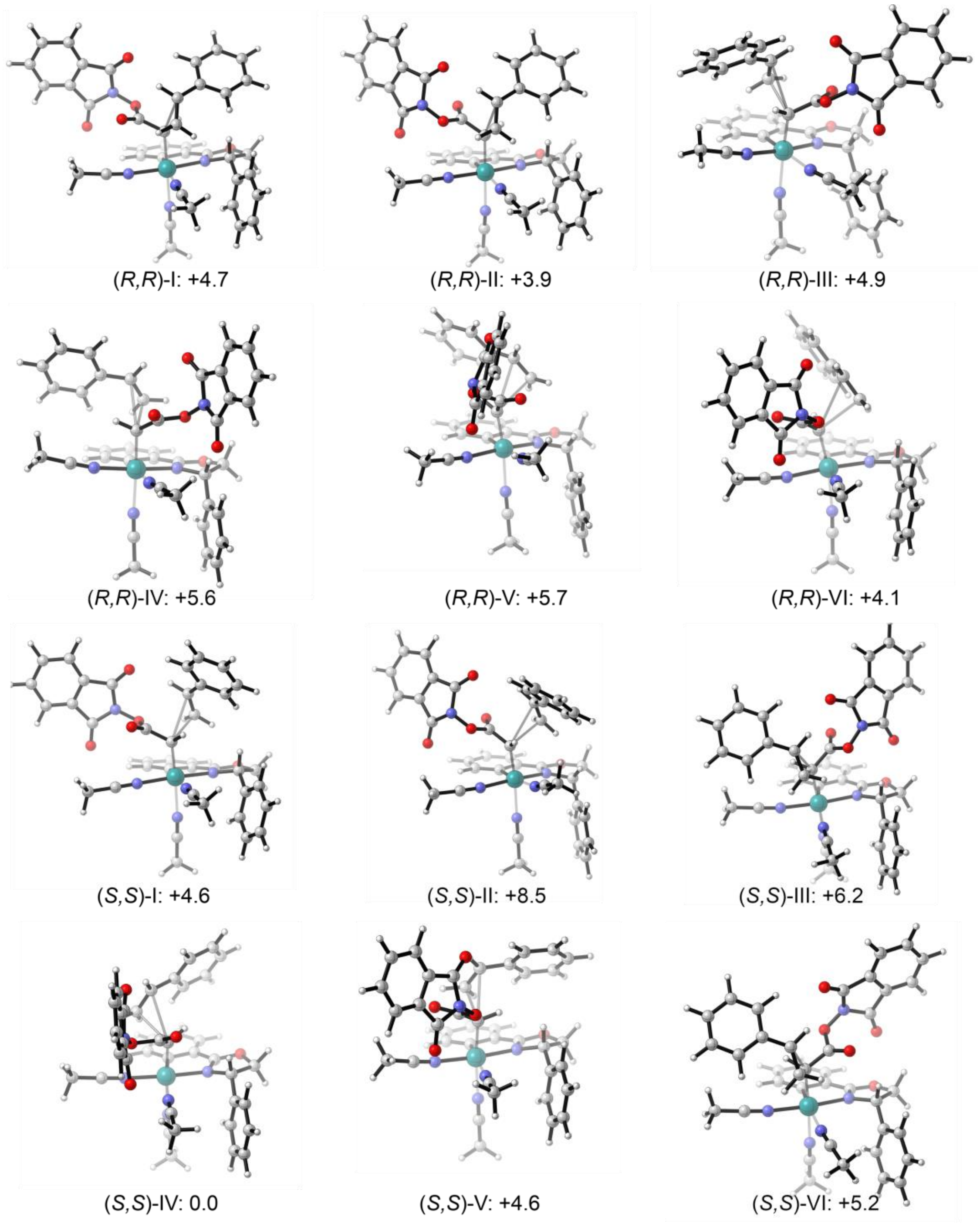

Figure S23. *Different outer-sphere cyclopropanation TSs for formation of the (R,R) and (S,S) products at the* ***Ap$_{anti}$*** *position. The values indicate the energy of each TS relative to the most stable outer-sphere* ***Ap$_{anti}$-TS***.

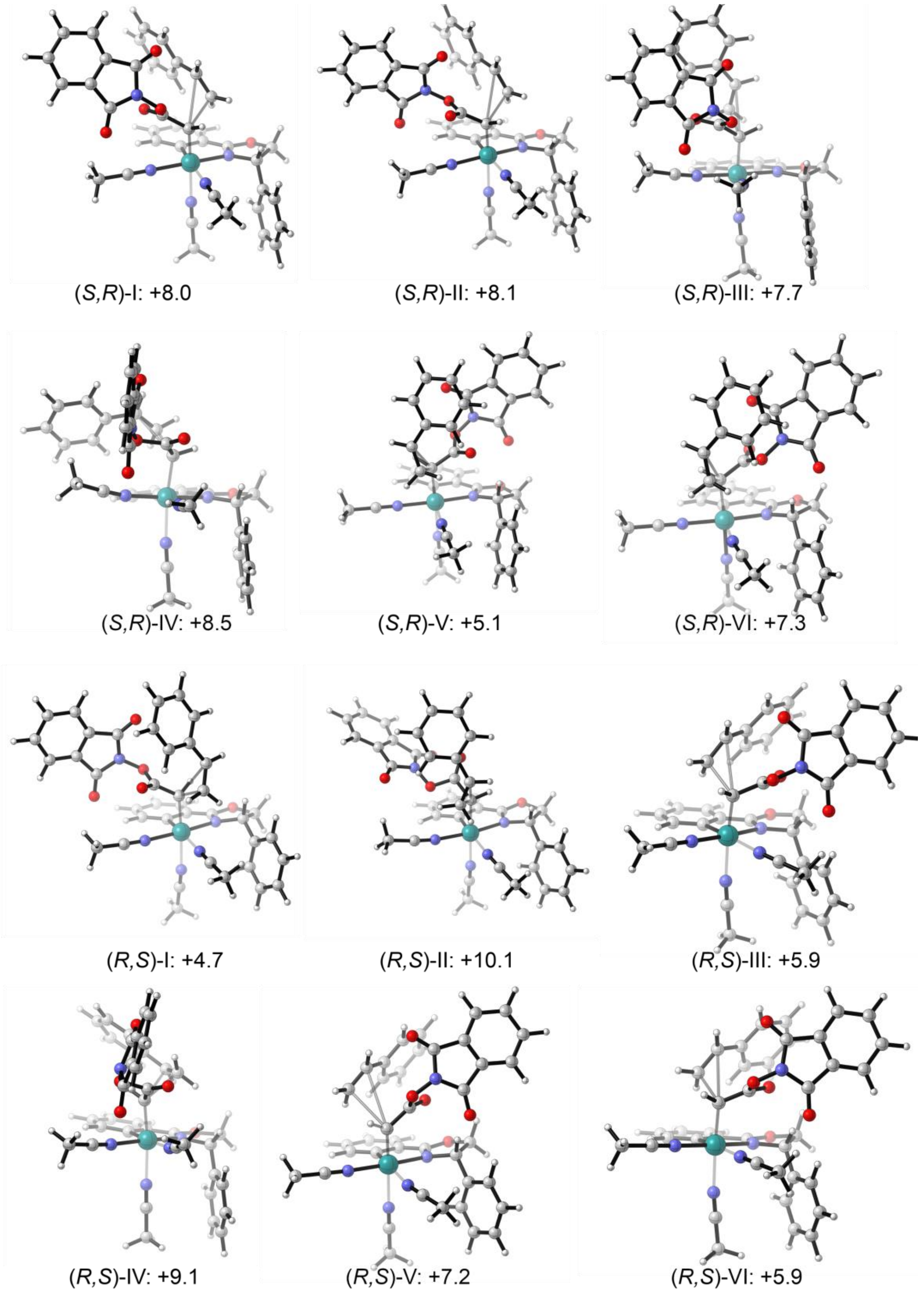

Figure S24. *Different outer-sphere cyclopropanation TSs for formation of the (R,S) and (S,R) products at the **Ap**$_{anti}$ position. The values indicate the energy of each TS relative to the most stable outer-sphere **Ap**$_{anti}$**-TS**.*

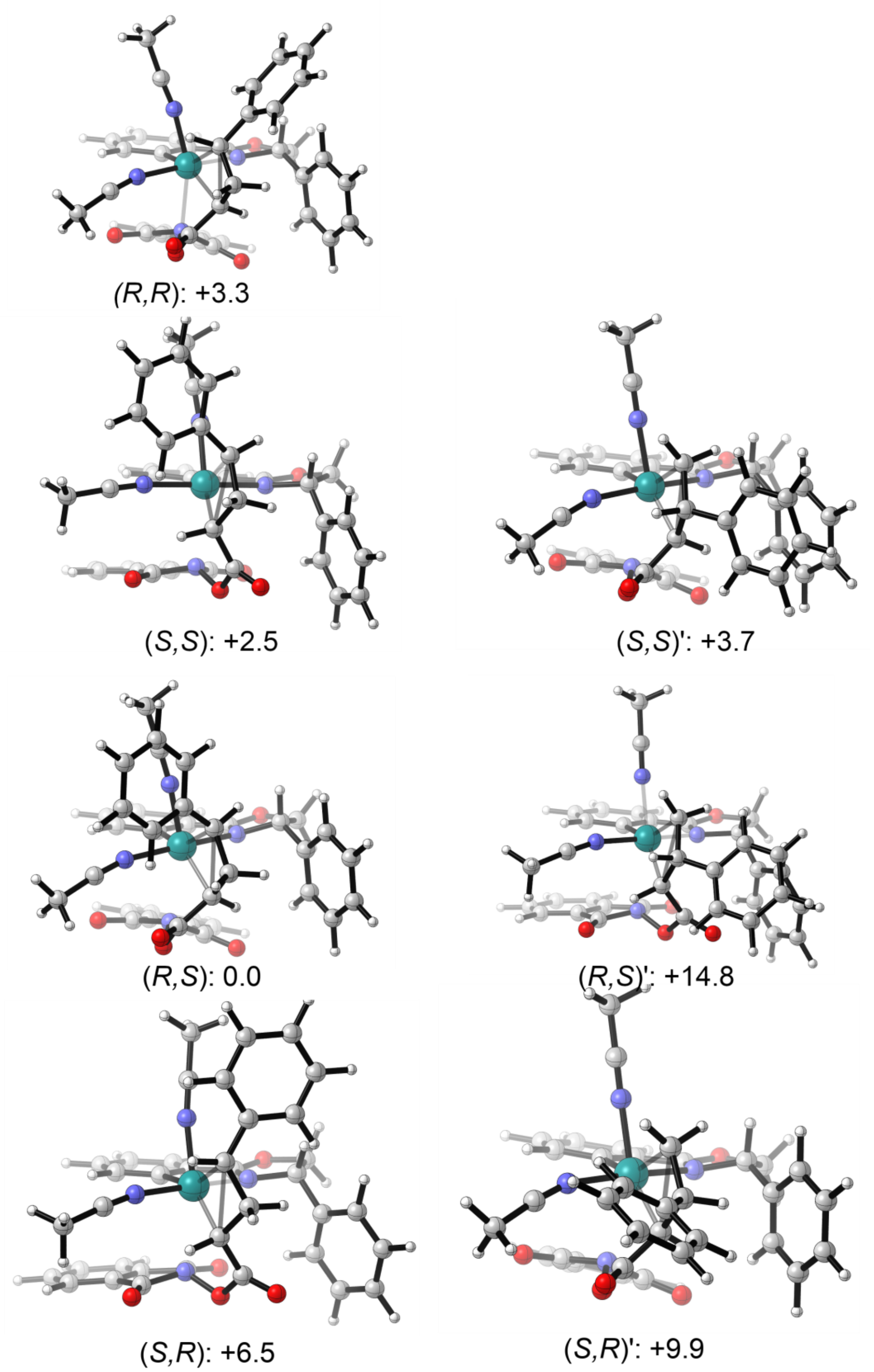

Figure S25. *Different inner-sphere reductive-elimination TSs at the* ***Ap$_{syn}$*** *position. The values indicate the energy of each TS relative to the most stable reductive-elimination* ***Ap$_{syn}$-TS****.*

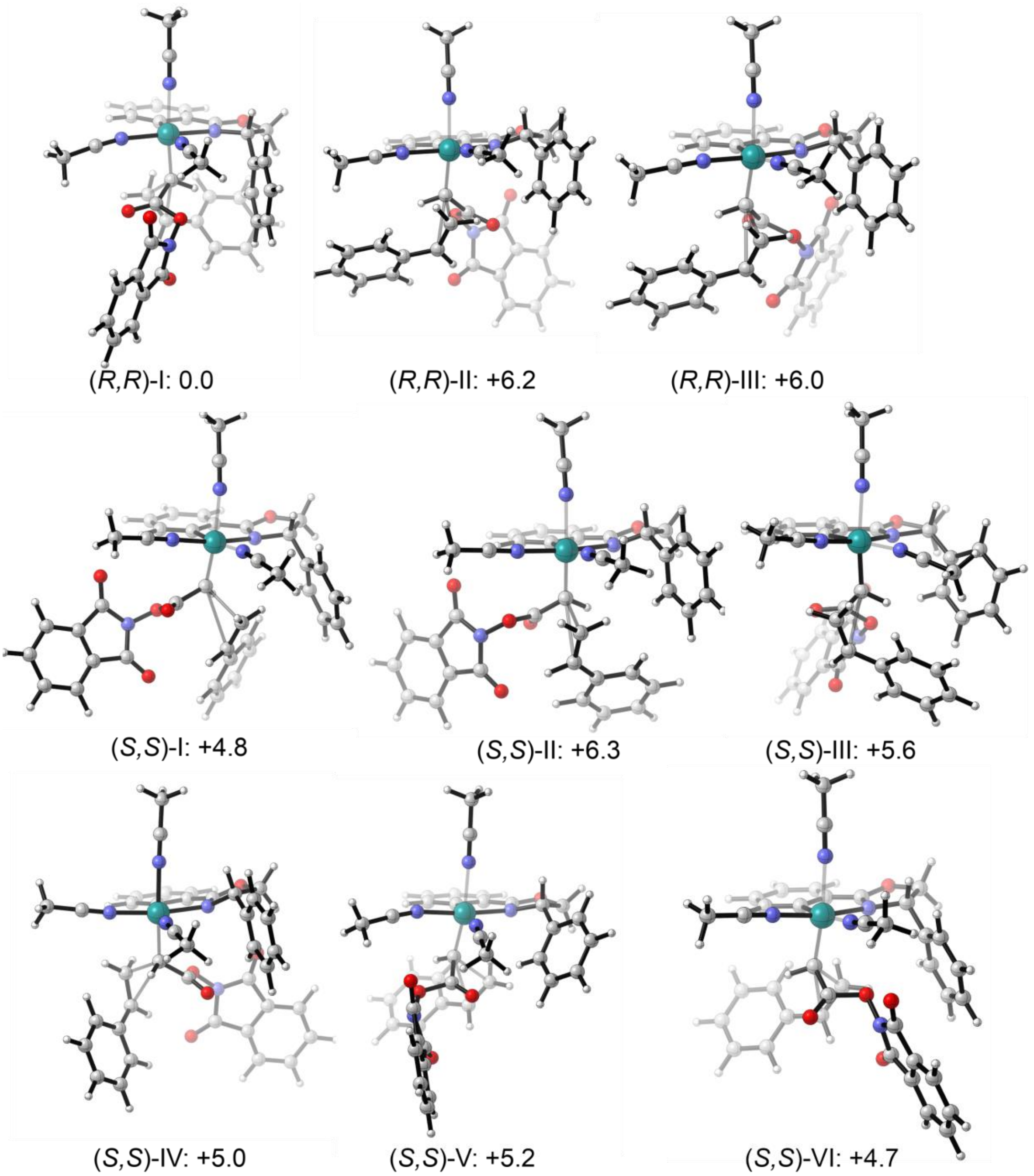

Figure S26. *Different outer-sphere cyclopropanation TSs for formation of the (R,R) and (S,S) products at the* ***Ap$_{syn}$*** *position. The values indicate the energy of each TS relative to the most stable outer-sphere* ***Ap$_{syn}$-TS***.

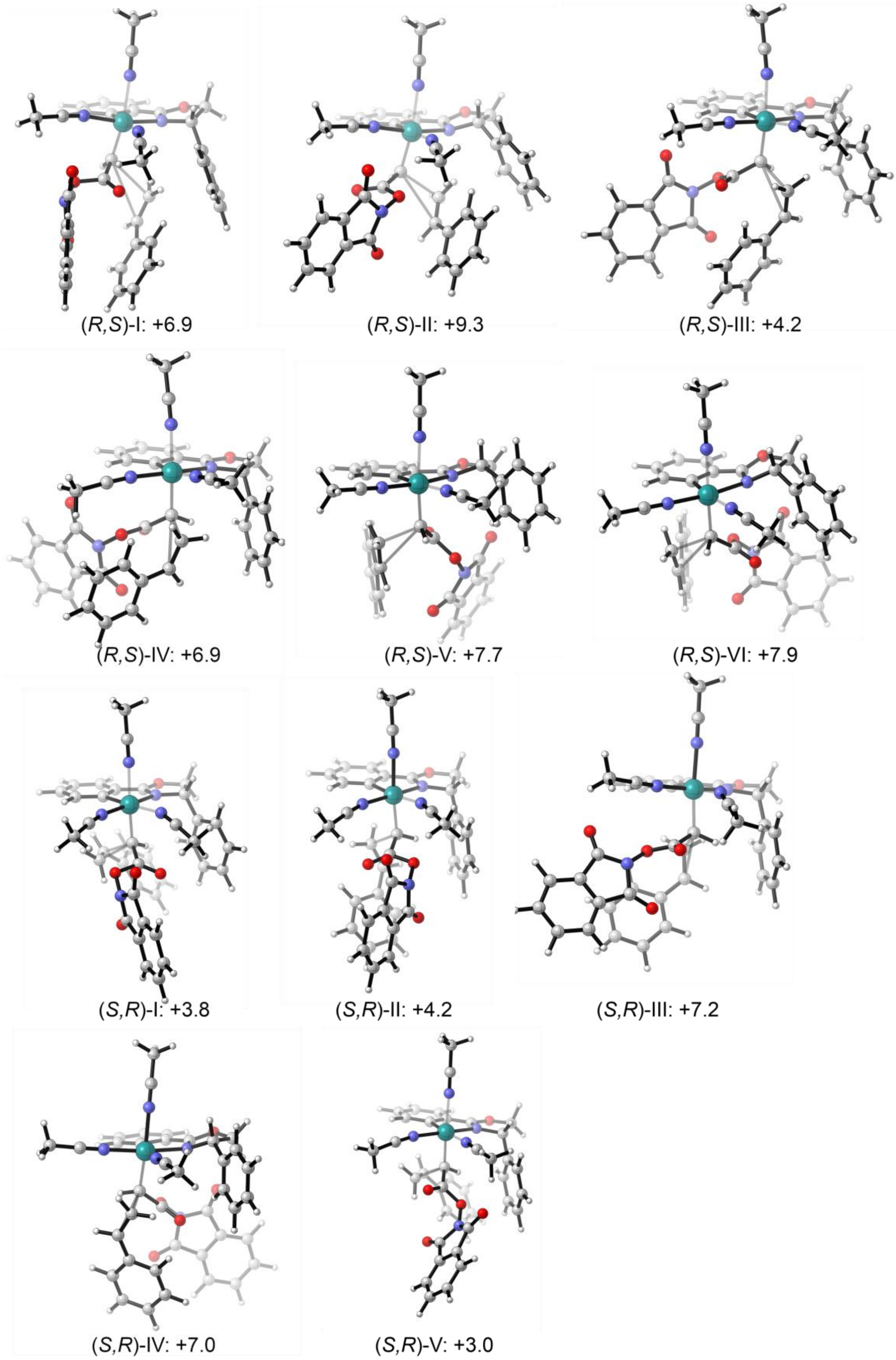

Figure S27. *Different outer-sphere cyclopropanation TSs for formation of the (R,S) and (S,R) products at the* ***Ap****$_{syn}$ position. The values indicate the energy of each TS relative to the most stable outer-sphere* ***AP****$_{syn}$**-TS**.

15. Cartesian coordinates.

[Ru(Pheox)(AN)$_4$]

Ru	0.6922	0.6449	0.0782
C	3.1823	-0.7442	-1.2234
C	1.9681	-0.8436	-0.5353
C	1.5407	-2.1569	-0.2014
C	2.2721	-3.3074	-0.5254
C	3.4740	-3.1660	-1.2097
C	3.9212	-1.8846	-1.5549
H	3.5639	0.2317	-1.5087
H	1.8987	-4.2872	-0.2432
H	4.0602	-4.0398	-1.4741
H	4.8610	-1.7764	-2.0904
C	-1.6055	-1.3306	1.4305
C	-1.5197	-2.8733	1.6518
H	-2.3718	-3.4138	1.2385
C	0.2795	-2.1757	0.5036
N	-0.3327	-1.0571	0.7538
H	-1.6271	-0.8020	2.3888
O	-0.3274	-3.2935	0.9278
C	-2.8001	-0.8954	0.6068
C	-3.7848	-0.0757	1.1620
C	-2.9497	-1.3407	-0.7118
C	-4.9030	0.3015	0.4139
H	-3.6812	0.2604	2.1908
C	-4.0661	-0.9704	-1.4597
H	-2.1895	-1.9811	-1.1497
C	-5.0440	-0.1425	-0.9011
H	-5.6718	0.9234	0.8634
H	-4.1871	-1.3442	-2.4725
H	-5.9173	0.1385	-1.4811
H	-1.3837	-3.1436	2.7012
C	-0.8400	0.4009	-2.7013
N	-0.2718	0.5154	-1.7001
C	-1.5789	0.2502	-3.9457
H	-2.6504	0.2168	-3.7295
H	-1.3744	1.0911	-4.6141
H	-1.2864	-0.6771	-4.4467
C	2.3008	0.6324	2.8310
N	1.6894	0.6889	1.8496
C	3.0784	0.5552	4.0600
H	3.5763	1.5100	4.2514
H	2.4263	0.3220	4.9066
H	3.8372	-0.2276	3.9717
C	-1.7977	2.6944	0.9927
C	-2.9960	3.4722	1.2748
H	-3.8684	2.9355	0.8916
H	-3.1100	3.6157	2.3528
H	-2.9349	4.4519	0.7933
N	-0.8619	2.0566	0.7539
C	2.5503	2.9923	-1.0590
N	1.8632	2.1576	-0.6423
C	3.4242	4.0313	-1.5876
H	4.4338	3.9136	-1.1837
H	3.4723	3.9621	-2.6781
H	3.0477	5.0208	-1.3131

[Ru(Pheox)(AN)$_3$]

Empty position: Ap$_{anti}$

Ru	-0.7851	0.6978	-0.5150
C	-3.5131	-0.5208	0.4222
C	-2.2137	-0.7030	-0.0610
C	-1.8045	-2.0461	-0.2848
C	-2.6393	-3.1472	-0.0639
C	-3.9299	-2.9238	0.4076
C	-4.3583	-1.6142	0.6485
H	-3.8805	0.4801	0.6276
H	-2.2800	-4.1525	-0.2608
H	-4.5978	-3.7592	0.5881
H	-5.3650	-1.4439	1.0203
C	1.6190	-1.4126	-1.3574
C	1.5035	-2.9580	-1.5326
H	2.2396	-3.5151	-0.9530
C	-0.4444	-2.1459	-0.7662
N	0.2477	-1.0580	-0.9615
H	1.8570	-0.9345	-2.3121
O	0.1805	-3.2975	-1.0171
C	2.6377	-0.9775	-0.3250
C	3.7414	-0.2083	-0.7004
C	2.5001	-1.3635	1.0134
C	4.6939	0.1794	0.2448
H	3.8626	0.0768	-1.7425
C	3.4531	-0.9851	1.9581
H	1.6471	-1.9651	1.3141
C	4.5495	-0.2060	1.5779
H	5.5573	0.7626	-0.0620
H	3.3538	-1.3150	2.9882
H	5.2948	0.0820	2.3125
H	1.5383	-3.2693	-2.5784
C	0.1717	0.6525	2.4262
N	-0.1972	0.6915	1.3296
C	0.6669	0.6011	3.7932
H	1.7552	0.4936	3.7800
H	0.4032	1.5180	4.3276
H	0.2297	-0.2519	4.3201
C	1.8658	2.5892	-1.2875
C	3.1156	3.3035	-1.5038
H	3.9064	2.8243	-0.9199
H	3.3862	3.2779	-2.5631
H	3.0161	4.3459	-1.1895
N	0.8851	2.0034	-1.0994
C	-2.7728	3.1705	-0.0502
N	-2.0307	2.2920	-0.1946
C	-3.7192	4.2638	0.1213
H	-4.6094	4.0840	-0.4884
H	-4.0195	4.3383	1.1704
H	-3.2649	5.2100	-0.1856

Empty position: Eq$_{trans}$

Ru	0.6042	0.7452	-0.7981
C	3.2659	-0.7231	-1.5024
C	2.2070	-0.5148	-0.6137
C	2.2269	-1.2686	0.5895
C	3.2408	-2.1823	0.9058
C	4.2778	-2.3625	-0.0028
C	4.2848	-1.6336	-1.1997
H	3.3084	-0.1771	-2.4427
H	3.2107	-2.7333	1.8408
H	5.0775	-3.0632	0.2124
H	5.0981	-1.7795	-1.9054
C	-0.8945	0.0214	1.9547
C	-0.3772	-0.8758	3.1220
H	-1.0829	-1.6561	3.4068
C	1.0884	-0.9708	1.4213
N	0.2186	-0.0925	1.0039
H	-0.9882	1.0626	2.2774
O	0.8244	-1.5271	2.6067
C	-2.2099	-0.4308	1.3570
C	-3.3201	0.4158	1.3670
C	-2.3340	-1.7124	0.8083
C	-4.5399	-0.0050	0.8320
H	-3.2318	1.4063	1.8062
C	-3.5509	-2.1373	0.2774
H	-1.4760	-2.3788	0.7995
C	-4.6564	-1.2820	0.2822
H	-5.4029	0.6537	0.8641
H	-3.6453	-3.1434	-0.1209
H	-5.6062	-1.6167	-0.1227
H	-0.0863	-0.2972	4.0009
C	-0.9781	-1.5814	-2.2816
N	-0.4051	-0.7254	-1.7530
C	-1.7165	-2.6520	-2.9326
H	-2.7105	-2.7311	-2.4835
H	-1.8212	-2.4432	-4.0010
H	-1.1891	-3.6021	-2.8087
C	2.4187	2.9657	0.5918
C	3.3183	3.9313	1.2062
H	2.8165	4.4459	2.0305
H	4.2051	3.4206	1.5921
H	3.6317	4.6725	0.4654
N	1.7116	2.1956	0.0926
C	-2.2200	2.5174	-1.0561
N	-1.1856	2.0066	-0.9561
C	-3.5373	3.1255	-1.1704
H	-3.6077	4.0034	-0.5223
H	-3.7235	3.4329	-2.2030
H	-4.2945	2.3955	-0.8703

Empty position: Eq$_{cis}$

Ru	-0.7135	0.5802	-0.4967
C	-2.5295	-0.1957	1.8533
C	-1.4722	-0.5285	1.0052
C	-0.8494	-1.7898	1.1919
C	-1.2634	-2.6881	2.1832
C	-2.3203	-2.3327	3.0146
C	-2.9451	-1.0915	2.8450
H	-3.0350	0.7580	1.7480
H	-0.7581	-3.6427	2.2925
H	-2.6580	-3.0119	3.7898
H	-3.7705	-0.8171	3.4961
C	1.7650	-1.4845	-1.3059
C	1.9318	-2.9586	-0.8547
H	2.9402	-3.2129	-0.5308

C	0.2407	-2.0206	0.2731
N	0.5106	-1.1187	-0.6207
H	1.6234	-1.4213	-2.3885
O	1.0395	-3.0967	0.2944
C	2.8722	-0.5380	-0.8881
C	3.2355	0.5204	-1.7240
C	3.4894	-0.6637	0.3629
C	4.2077	1.4398	-1.3246
H	2.7612	0.6219	-2.6968
C	4.4721	0.2421	0.7575
H	3.2035	-1.4705	1.0320
C	4.8326	1.2981	-0.0853
H	4.4880	2.2528	-1.9869
H	4.9641	0.1203	1.7180
H	5.6072	1.9962	0.2173
H	1.5972	-3.6656	-1.6176
C	1.4203	1.8987	1.4653
N	0.6313	1.4409	0.7527
C	2.4098	2.4825	2.3573
H	3.2007	2.9533	1.7684
H	1.9408	3.2277	3.0057
H	2.8568	1.6988	2.9741
C	-2.7542	-0.8643	-2.4730
C	-3.6901	-1.5692	-3.3371
H	-3.1462	-2.1873	-4.0570
H	-4.3381	-2.2133	-2.7357
H	-4.3108	-0.8533	-3.8833
N	-2.0101	-0.3032	-1.7846
C	-2.6642	3.1013	-0.1667
N	-1.9669	2.1819	-0.2804
C	-3.5438	4.2520	-0.0149
H	-4.5768	3.9668	-0.2336
H	-3.4916	4.6316	1.0096
H	-3.2443	5.0483	-0.7023

Empty position: Ap_{syn}

Ru	0.5949	0.6522	-0.0862
C	3.3778	-0.4164	-1.0388
C	2.0858	-0.6719	-0.5683
C	1.7305	-2.0385	-0.4055
C	2.5961	-3.0955	-0.7080
C	3.8712	-2.8006	-1.1824
C	4.2541	-1.4650	-1.3445
H	3.7146	0.6071	-1.1728
H	2.2708	-4.1225	-0.5740
H	4.5628	-3.6006	-1.4239
H	5.2514	-1.2385	-1.7121
C	-1.6594	-1.5842	0.8439
C	-1.5613	-3.1367	0.7228
H	-2.2094	-3.5322	-0.0621
C	0.3823	-2.2122	0.0878
N	-0.3551	-1.1624	0.3198
H	-1.7348	-1.2813	1.8951
O	-0.1801	-3.3973	0.3293
C	-2.8270	-0.9962	0.0834
C	-4.0031	-0.6584	0.7588
C	-2.7577	-0.8155	-1.3024
C	-5.1020	-0.1560	0.0592
H	-4.0623	-0.7950	1.8357
C	-3.8479	-0.2985	-2.0014
H	-1.8460	-1.0775	-1.8318
C	-5.0251	0.0287	-1.3227
H	-6.0158	0.0892	0.5918
H	-3.7840	-0.1610	-3.0763
H	-5.8793	0.4157	-1.8695
H	-1.7471	-3.6633	1.6591
C	2.2583	3.2101	-1.0706
N	1.6522	2.2984	-0.6899
C	3.0250	4.3457	-1.5632
H	2.4956	5.2796	-1.3547
H	4.0038	4.3766	-1.0762
H	3.1717	4.2550	-2.6435
C	1.7913	0.7572	2.7668
C	2.3839	0.7635	4.0969
H	1.6815	0.3481	4.8252
H	3.2963	0.1602	4.1019
H	2.6344	1.7863	4.3927
N	1.3205	0.7550	1.7083
C	-2.2322	2.3815	0.3481
N	-1.1946	1.8721	0.2826
C	-3.5478	3.0000	0.4115
H	-4.0554	2.6983	1.3312
H	-3.4576	4.0892	0.3880
H	-4.1438	2.6597	-0.4389

[Ru(Pheox)(Prop)$_2$(AN)$_2$]

Olefins coordinated at: Eq_{trans} and Eq_{cis}

Ru	0.7724	-0.5600	0.3533
C	2.3796	1.9045	1.6144
C	1.4608	1.3899	0.6908
C	0.9477	2.3338	-0.2424
C	1.3222	3.6839	-0.2695
C	2.2417	4.1482	0.6617
C	2.7600	3.2520	1.5996
H	2.8298	1.2759	2.3700
H	0.8853	4.3467	-1.0099
H	2.5484	5.1885	0.6639
H	3.4771	3.6037	2.3364
C	-1.5896	0.3286	-1.8699
C	-1.5959	1.6174	-2.7319
H	-2.5796	2.0737	-2.8334
C	-0.0443	1.7922	-1.1338
N	-0.3593	0.5313	-1.0726
H	-1.4888	-0.5500	-2.5037
O	-0.7437	2.5418	-1.9961
C	-2.8201	0.1828	-0.9968
C	-3.6698	-0.9153	-1.1510
C	-3.1486	1.1722	-0.0607
C	-4.8261	-1.0367	-0.3766
H	-3.4356	-1.6766	-1.8906
C	-4.3111	1.0638	0.6997
H	-2.5009	2.0341	0.0672
C	-5.1504	-0.0451	0.5490
H	-5.4770	-1.8952	-0.5076
H	-4.5691	1.8486	1.4046
H	-6.0588	-0.1256	1.1378
C	-0.6892	-2.8438	-0.1873
C	0.2528	-2.9392	-1.1469
H	-1.6922	-2.4928	-0.4036
H	-0.5085	-3.2037	0.8180
H	1.2268	-3.3387	-0.8859
C	2.2850	-1.0918	2.2675
C	1.7822	-2.3300	2.0066
H	3.2738	-0.8108	1.9201
H	1.8086	-0.4416	2.9898
H	0.8583	-2.6177	2.5039
C	0.0562	-2.6559	-2.6062
H	0.3980	-3.5118	-3.1983
H	-0.9935	-2.4826	-2.8520
H	0.6441	-1.7884	-2.9258
C	2.5557	-3.4297	1.3379
H	1.9088	-4.1431	0.8233
H	3.2921	-3.0348	0.6334
H	3.0973	-3.9980	2.1047
H	-1.1462	1.4620	-3.7167
C	-1.5451	-0.2057	2.5156
N	-0.7092	-0.3408	1.7260
C	-2.6091	-0.0472	3.4952
H	-3.5619	-0.3331	3.0424
H	-2.4152	-0.6756	4.3689
H	-2.6689	0.9966	3.8157
C	3.2348	-0.6793	-1.6681

N 2.3270 -0.7057 -0.9482
C 4.3821 -0.6367 -2.5639
H 5.1366 -1.3616 -2.2454
H 4.0735 -0.8743 -3.5859
H 4.8252 0.3632 -2.5509

Olefins coordinated at: Ap_{syn} *and* Eq_{cis}

Ru -0.7077 -0.4949 0.0541
C -2.6009 1.3965 -1.5326
C -1.4426 1.2289 -0.7665
C -0.6825 2.3965 -0.4919
C -1.0506 3.6668 -0.9538
C -2.2056 3.7956 -1.7180
C -2.9726 2.6606 -2.0042
H -3.2257 0.5401 -1.7678
H -0.4345 4.5289 -0.7176
H -2.5091 4.7681 -2.0904
H -3.8744 2.7635 -2.6018
C 2.1352 0.9159 1.2764
C 2.3557 2.4339 1.5154
H 3.3441 2.7838 1.2204
C 0.5106 2.1331 0.2825
N 0.8072 0.9135 0.6222
H 2.0733 0.3793 2.2254
O 1.3714 3.0882 0.6602
C 3.1807 0.2649 0.3947
C 3.7634 -0.9512 0.7581
C 3.5528 0.8626 -0.8164
C 4.6829 -1.5799 -0.0851
H 3.5059 -1.4084 1.7093
C 4.4727 0.2418 -1.6578
H 3.1131 1.8123 -1.1096
C 5.0340 -0.9867 -1.2976
H 5.1286 -2.5247 0.2096
H 4.7523 0.7137 -2.5944
H 5.7497 -1.4711 -1.9539
C 0.5695 -2.7869 0.6405
C -0.0741 -2.5613 1.8057
H 1.6206 -2.5484 0.5168
H 0.0828 -3.3192 -0.1669
H -1.1096 -2.8846 1.8972
C 0.8491 -0.6072 -1.9711
C -0.2594 -1.2097 -2.4760
H 1.7222 -1.1793 -1.6764
H 0.9628 0.4678 -2.0137
H -1.0571 -0.5776 -2.8594
C 0.5607 -2.0315 3.0564
H 0.5554 -2.8061 3.8323
H 1.5975 -1.7352 2.8862
H 0.0112 -1.1740 3.4567
C -0.3741 -2.6778 -2.7573
H -0.3982 -2.8285 -3.8434
H 0.4772 -3.2364 -2.3623
H -1.2989 -3.1040 -2.3583
H 2.1463 2.7320 2.5460
C -3.2902 -2.2475 -0.6315
N -2.3345 -1.6258 -0.4171
C -4.4913 -3.0208 -0.9174
H -4.4126 -4.0195 -0.4785
H -5.3693 -2.5195 -0.4999
H -4.6245 -3.1205 -1.9986
C -2.1848 0.4841 2.6707
N -1.6347 0.0524 1.7468
C -2.8804 1.0337 3.8261
H -3.4180 0.2425 4.3562
H -2.1627 1.4928 4.5120
H -3.5960 1.7952 3.5031

Olefins coordinated at: Ap_{anti} *and* Eq_{cis}

Ru 0.7603 -0.5455 -0.1770
C 2.8867 1.4983 0.8187
C 1.6590 1.2523 0.1954
C 0.9011 2.3887 -0.1882
C 1.3468 3.7035 0.0078
C 2.5785 3.9097 0.6179
C 3.3389 2.8059 1.0235
H 3.5073 0.6691 1.1427
H 0.7317 4.5383 -0.3134
H 2.9471 4.9167 0.7810
H 4.2996 2.9691 1.5045
C -2.0618 0.7346 -1.5027
C -2.2859 2.2131 -1.9345
H -3.2471 2.6166 -1.6182
C -0.3655 2.0501 -0.7933
N -0.7085 0.8028 -0.9215
H -2.0526 0.0755 -2.3756
O -1.2361 2.9593 -1.2508
C -3.0887 0.2308 -0.5094
C -3.8860 -0.8762 -0.8088
C -3.2736 0.8943 0.7104
C -4.8438 -1.3309 0.1011
H -3.7619 -1.3855 -1.7609
C -4.2393 0.4527 1.6130
H -2.6691 1.7658 0.9457
C -5.0210 -0.6677 1.3146
H -5.4548 -2.1937 -0.1435
H -4.3996 0.9934 2.5415
H -5.7736 -1.0099 2.0176
C -0.8117 -2.6098 0.0664
C -0.1322 -3.0070 -1.0309
H -1.8018 -2.1729 -0.0016
H -0.4466 -2.8428 1.0595
H 0.8106 -3.5322 -0.8888
C 1.3253 -0.2527 -2.3941
C 2.3386 -1.0626 -1.9471
H 1.5047 0.8098 -2.5205
H 0.4578 -0.6556 -2.9000
H 2.2189 -2.1394 -2.0330
C -0.6710 -2.9830 -2.4310
H -1.0067 -3.9893 -2.7104
H -1.5269 -2.3094 -2.5222
H 0.0876 -2.6967 -3.1654
C 3.7374 -0.5809 -1.6930
H 4.3596 -0.8464 -2.5569
H 3.7749 0.5008 -1.5601
H 4.1847 -1.0561 -0.8163
H -2.1511 2.3631 -3.0085
C 2.9192 -2.2790 1.4227
N 2.1618 -1.6597 0.8010
C 3.8856 -3.0419 2.2014
H 3.3781 -3.8234 2.7740
H 4.6168 -3.5108 1.5368
H 4.4126 -2.3806 2.8953
C -0.6875 -0.0534 2.6363
N -0.1615 -0.2867 1.6333
C -1.3747 0.2338 3.8860
H -2.4395 0.0154 3.7694
H -0.9660 -0.3826 4.6912
H -1.2496 1.2880 4.1488

Olefins coordinated at: Ap_{anti} *and* Ap_{syn}

Ru 0.6740 0.5738 0.2459
C 3.3605 -0.6910 -0.8301
C 2.0835 -0.8646 -0.2877
C 1.6617 -2.2019 -0.0696
C 2.4627 -3.3124 -0.3739
C 3.7252 -3.1013 -0.9141
C 4.1660 -1.7909 -1.1399
H 3.7391 0.3100 -1.0162
H 2.0918 -4.3155 -0.1877
H 4.3644 -3.9428 -1.1583
H 5.1539 -1.6274 -1.5621
C -1.7174 -1.5753 1.1235
C -1.5744 -3.1083 1.3356
H -2.3511 -3.6914 0.8413
C 0.3287 -2.2921 0.4738
N -0.3475 -1.2041 0.7148
H -1.9647 -1.0776 2.0637
O -0.2966 -3.4504 0.7227
C -2.7317 -1.1748 0.0736
C -3.7232 -0.2365 0.3690
C -2.6763 -1.7244 -1.2132
C -4.6362 0.1649 -0.6087
H -3.7856 0.1724 1.3742
C -3.5824 -1.3234 -2.1932
H -1.9203 -2.4677 -1.4512
C -4.5612 -0.3717 -1.8949
H -5.4166 0.8797 -0.3637
H -3.5284 -1.7545 -3.1877
H -5.2702 -0.0635 -2.6565
C 2.0623 0.3113 2.0295
C 0.9929 1.0551 2.4915
H 2.1333 -0.7417 2.2797
H 2.9848 0.7822 1.7123
H 1.0696 2.1394 2.4621
C 0.2859 0.0069 -2.1366
C -0.6086 1.0139 -1.9472
H 1.2408 0.1863 -2.6184
H -0.0049 -1.0262 -2.0039
H -1.5999 0.7557 -1.5874
C -0.0536 0.4970 3.4145
H -1.0516 0.8804 3.1875
H -0.0739 -0.5942 3.3762
H 0.1815 0.7932 4.4444

C	-0.4081	2.4231	-2.4193
H	-0.6494	3.1524	-1.6418
H	0.6163	2.5924	-2.7575
H	-1.0812	2.6131	-3.2643
H	-1.5185	-3.3826	2.3919
C	2.5810	3.0215	-0.5137
N	1.8625	2.1501	-0.2506
C	3.4826	4.1101	-0.8657
H	3.6141	4.7829	-0.0136
H	4.4595	3.7107	-1.1536
H	3.0730	4.6795	-1.7051
C	-1.9404	2.5390	0.9172
N	-1.0006	1.8942	0.7158
C	-3.1481	3.3215	1.1390
H	-3.9593	2.9071	0.5338
H	-3.4345	3.2873	2.1938
H	-2.9836	4.3636	0.8517

[Ru(Pheox)(Prop)(AN)$_3$]

Olefin coordinated at: Eq$_{trans}$

Ru	-0.7068	0.7318	0.3268
C	-3.2265	-1.0014	1.3360
C	-2.0785	-0.8538	0.5470
C	-1.7710	-1.9727	-0.2782
C	-2.5428	-3.1417	-0.3278
C	-3.6739	-3.2389	0.4720
C	-4.0048	-2.1645	1.3014
H	-3.5493	-0.2127	2.0034
H	-2.2467	-3.9532	-0.9853
H	-4.2879	-4.1329	0.4541
H	-4.8858	-2.2289	1.9343
C	1.3220	-0.8339	-1.8085
C	1.0417	-2.1606	-2.5816
H	1.8631	-2.8747	-2.5223
C	-0.5754	-1.8134	-1.0677
N	0.1218	-0.7248	-0.9709
H	1.3656	0.0134	-2.4995
O	-0.1117	-2.7449	-1.9124
C	2.6012	-0.8734	-0.9972
C	3.7230	-0.1537	-1.4165
C	2.6971	-1.6796	0.1425
C	4.9226	-0.2244	-0.7041
H	3.6607	0.4579	-2.3136
C	3.8944	-1.7559	0.8532
H	1.8318	-2.2467	0.4708
C	5.0091	-1.0232	0.4367
H	5.7911	0.3297	-1.0479
H	3.9669	-2.4043	1.7217
H	5.9420	-1.0884	0.9872
C	-2.0776	1.7326	1.8292
C	-1.1212	2.6773	1.5200
H	-3.0677	1.8050	1.3914
H	-1.9898	1.1227	2.7200
H	-0.2396	2.7419	2.1539
C	-1.3930	3.8801	0.6573
H	-0.5541	4.1155	-0.0021
H	-2.2871	3.7376	0.0466
H	-1.5617	4.7528	1.3005
H	0.7696	-1.9909	-3.6261
C	0.9702	-0.4171	2.7749
N	0.3592	0.0132	1.8916
C	1.7608	-0.9706	3.8640
H	2.8006	-1.0730	3.5410
H	1.7194	-0.3125	4.7363
H	1.3744	-1.9543	4.1454
C	-2.5629	1.6328	-2.0983
N	-1.8542	1.3697	-1.2217
C	-3.4692	1.9667	-3.1880
H	-3.8815	2.9688	-3.0392
H	-2.9381	1.9415	-4.1436
H	-4.2920	1.2468	-3.2204
C	2.0105	2.5438	-0.3623
C	3.2805	3.2225	-0.5766
H	4.0974	2.5232	-0.3795
H	3.3520	3.5732	-1.6098
H	3.3659	4.0812	0.0948

N	1.0095	1.9933	-0.1786

Olefin coordinated at: Eq$_{cis}$

Ru	-0.7726	-0.4992	0.2698
C	-2.5521	0.9922	-1.8357
C	-1.4868	1.0158	-0.9296
C	-0.8230	2.2585	-0.7706
C	-1.1948	3.4205	-1.4603
C	-2.2604	3.3593	-2.3504
C	-2.9300	2.1438	-2.5343
H	-3.1020	0.0732	-2.0089
H	-0.6496	4.3452	-1.2979
H	-2.5685	4.2428	-2.8992
H	-3.7606	2.0946	-3.2336
C	1.8813	1.2325	1.4212
C	2.0234	2.7784	1.4308
H	3.0206	3.1288	1.1676
C	0.2922	2.1911	0.1412
N	0.5895	1.0676	0.7262
H	1.8141	0.8451	2.4403
O	1.0994	3.2304	0.3988
C	3.0090	0.5310	0.6877
C	3.8309	-0.3838	1.3505
C	3.2648	0.8259	-0.6580
C	4.8877	-1.0067	0.6824
H	3.6496	-0.6070	2.3987
C	4.3302	0.2205	-1.3219
H	2.6372	1.5385	-1.1846
C	5.1411	-0.7038	-0.6552
H	5.5176	-1.7157	1.2102
H	4.5359	0.4770	-2.3570
H	5.9733	-1.1718	-1.1718
C	0.5190	-1.7079	2.0287
C	-0.3188	-2.6812	1.5888
H	1.5441	-1.6634	1.6778
H	0.2499	-1.0665	2.8605
H	-1.2985	-2.7771	2.0524
C	0.0873	-3.7843	0.6542
H	-0.6484	-3.9344	-0.1413
H	1.0582	-3.5792	0.1973
H	0.1642	-4.7280	1.2078
H	1.7059	3.2223	2.3785
C	1.1980	-1.4739	-2.0285
N	0.4649	-1.1341	-1.2004
C	2.1383	-1.9093	-3.0496
H	3.1507	-1.8883	-2.6376
H	1.8979	-2.9248	-3.3761
H	2.0921	-1.2392	-3.9128
C	-2.6880	0.6608	2.5415
N	-1.9921	0.1932	1.7430
C	-3.5679	1.2566	3.5374
H	-4.2505	0.5018	3.9377
H	-2.9792	1.6719	4.3602
H	-4.1547	2.0593	3.0818
C	-3.0903	-2.4384	-0.7837
C	-4.1609	-3.2766	-1.3067
H	-3.8219	-4.3125	-1.3960
H	-5.0241	-3.2454	-0.6358
H	-4.4690	-2.9190	-2.2936

N -2.2426 -1.7602 -0.3786

Olefin coordinated at: Ap_{syn}

Ru	0.5902	0.5892	-0.0079
C	3.4024	-0.6001	-0.7005
C	2.0454	-0.7947	-0.4106
C	1.6089	-2.1458	-0.3479
C	2.4552	-3.2398	-0.5820
C	3.7892	-3.0012	-0.8870
C	4.2555	-1.6810	-0.9394
H	3.8054	0.4066	-0.7517
H	2.0656	-4.2512	-0.5191
H	4.4651	-3.8281	-1.0767
H	5.3013	-1.4957	-1.1703
C	-1.7923	-1.5932	0.7904
C	-1.7488	-3.1397	0.6458
H	-2.3625	-3.4895	-0.1884
C	0.2230	-2.2740	0.0324
N	-0.5140	-1.2098	0.1691
H	-1.7530	-1.3021	1.8485
O	-0.3579	-3.4425	0.3392
C	-3.0194	-0.9661	0.1717
C	-3.9592	-0.3239	0.9814
C	-3.2557	-1.0556	-1.2055
C	-5.1206	0.2208	0.4281
H	-3.7868	-0.2586	2.0526
C	-4.4032	-0.4960	-1.7644
H	-2.5361	-1.5620	-1.8415
C	-5.3412	0.1422	-0.9479
H	-5.8535	0.6982	1.0717
H	-4.5734	-0.5685	-2.8340
H	-6.2438	0.5617	-1.3811
C	-0.2657	0.3682	-2.1177
C	0.6013	1.4261	-2.2440
H	0.0218	-0.6155	-2.4760
H	-1.3198	0.5274	-1.9323
H	0.2117	2.4273	-2.0717
C	1.9201	1.3472	-2.9563
H	2.6923	1.9433	-2.4634
H	2.2747	0.3190	-3.0360
H	1.7961	1.7515	-3.9689
H	-2.0208	-3.6773	1.5539
C	1.2380	0.0434	3.0849
N	0.9655	0.2964	1.9888
C	1.5921	-0.2797	4.4602
H	1.6375	0.6320	5.0622
H	0.8438	-0.9504	4.8916
H	2.5671	-0.7745	4.4873
C	2.6670	3.0161	0.2131
N	1.8948	2.1614	0.0903
C	3.6512	4.0806	0.3570
H	4.6474	3.6527	0.5021
H	3.6630	4.7046	-0.5411
H	3.4069	4.7082	1.2186
C	-2.1116	2.5413	0.4091
C	-3.3517	3.3029	0.4552
H	-4.1440	2.7247	-0.0271
H	-3.6360	3.4958	1.4932
H	-3.2273	4.2580	-0.0621

N -1.1353 1.9217 0.3573

Olefin coordinated at: Ap_{anti}

Ru	-0.7574	0.5489	-0.2425
C	-3.3577	-0.9804	0.5850
C	-2.0393	-1.0077	0.1140
C	-1.4979	-2.2935	-0.1622
C	-2.2168	-3.4835	0.0189
C	-3.5213	-3.4124	0.4927
C	-4.0847	-2.1605	0.7703
H	-3.8328	-0.0305	0.8123
H	-1.7556	-4.4382	-0.2146
H	-4.1004	-4.3175	0.6411
H	-5.1072	-2.1072	1.1348
C	1.7712	-1.2852	-1.4081
C	1.7728	-2.8209	-1.6803
H	2.6176	-3.3379	-1.2251
C	-0.1542	-2.2389	-0.6875
N	0.4243	-1.0866	-0.8554
H	1.8609	-0.7265	-2.3450
O	0.5525	-3.3161	-1.0559
C	2.8628	-0.8294	-0.4619
C	3.8050	0.1171	-0.8689
C	2.9646	-1.3823	0.8202
C	4.8320	0.5156	-0.0094
H	3.7430	0.5340	-1.8710
C	3.9909	-0.9916	1.6788
H	2.2429	-2.1288	1.1405
C	4.9245	-0.0353	1.2689
H	5.5721	1.2359	-0.3456
H	4.0802	-1.4497	2.6596
H	5.7295	0.2610	1.9338
C	-1.2506	0.3588	-2.4630
C	-2.1198	1.3201	-2.0042
H	-0.3578	0.6459	-3.0115
H	-1.5676	-0.6728	-2.5555
H	-3.1017	0.9955	-1.6677
C	-1.9593	2.7874	-2.2894
H	-2.2615	3.4094	-1.4435
H	-0.9276	3.0323	-2.5503
H	-2.5993	3.0575	-3.1386
H	1.7205	-3.0608	-2.7445
C	0.5703	0.0603	2.6381
N	0.0852	0.2595	1.6076
C	1.2026	-0.1912	3.9242
H	2.2875	-0.1122	3.8152
H	0.8604	0.5397	4.6618
H	0.9490	-1.1947	4.2775
C	-2.6577	2.6243	1.2918
N	-1.9743	1.9096	0.6888
C	-3.5322	3.5126	2.0456
H	-4.3034	3.9244	1.3884
H	-4.0180	2.9611	2.8557
H	-2.9567	4.3379	2.4740
C	1.7075	2.7646	-0.7681
C	2.8718	3.6303	-0.8906
H	3.7309	3.1435	-0.4210
H	3.0972	3.8140	-1.9448
H	2.6856	4.5883	-0.3977

N 0.7948 2.0611 -0.6600

Carbene formation.

Int1-Ap$_{anti}$

Ru	1.4947	-0.5513	0.7139
C	3.8323	-2.4770	-0.1121
C	2.8896	-1.5004	-0.4586
C	2.8965	-1.0787	-1.8187
C	3.7773	-1.5948	-2.7786
C	4.6947	-2.5651	-2.3920
C	4.7155	-3.0017	-1.0611
H	3.8841	-2.8379	0.9114
H	3.7355	-1.2377	-3.8031
H	5.3874	-2.9827	-3.1150
H	5.4321	-3.7626	-0.7628
C	0.2616	1.4281	-1.6451
C	0.5037	1.3157	-3.1754
H	0.7039	2.2700	-3.6612
C	1.8889	-0.0782	-2.1012
N	1.1014	0.3226	-1.1469
H	-0.7813	1.2353	-1.3912
O	1.7021	0.4881	-3.3014
C	0.6806	2.7580	-1.0500
C	-0.2418	3.5326	-0.3421
C	1.9848	3.2365	-1.2244
C	0.1355	4.7648	0.1966
H	-1.2566	3.1675	-0.2237
C	2.3586	4.4730	-0.6996
H	2.7071	2.6456	-1.7808
C	1.4364	5.2377	0.0210
H	-0.5914	5.3648	0.7365
H	3.3636	4.8497	-0.8675
H	1.7259	6.2030	0.4247
H	-0.3089	0.7949	-3.6888
C	-0.7153	1.4327	2.2262
N	0.0363	0.6783	1.7752
C	-1.6637	2.3953	2.7635
H	-1.2186	3.3938	2.7453
H	-2.5583	2.3898	2.1357
H	-1.9307	2.1359	3.7912
C	3.6404	1.7232	1.3035
N	2.8730	0.8798	1.1102
C	4.5858	2.8037	1.5396
H	4.9559	2.7667	2.5678
H	5.4344	2.7153	0.8553
H	4.0868	3.7611	1.3673
C	-5.0866	0.3196	-0.1310
C	-5.4088	-1.0185	-0.4024
C	-6.7178	-1.4717	-0.3556
C	-7.7105	-0.5400	-0.0226
C	-7.3897	0.7959	0.2465
C	-6.0643	1.2475	0.1934
C	-3.6175	0.5003	-0.2530
C	-4.1596	-1.7589	-0.7106
H	-6.9567	-2.5083	-0.5657
H	-8.7463	-0.8585	0.0287
H	-8.1821	1.4920	0.5010
H	-5.8078	2.2813	0.3980
O	-2.9167	1.4630	-0.0106
O	-3.9340	-2.9304	-0.8827
O	-1.8350	-1.0794	-0.7529
C	-1.4191	-1.5775	0.4892
O	-2.0789	-1.5120	1.4864
C	-0.0514	-2.1407	0.3762
H	0.1630	-2.9087	1.1158
N	0.2149	-2.6345	-0.8868
N	0.5143	-2.9397	-1.9197
N	-3.1665	-0.7326	-0.7567
C	2.6798	-2.8279	4.6003
H	3.4126	-2.2426	5.1631
H	3.1223	-3.7962	4.3487
H	1.8019	-2.9934	5.2312
C	2.3007	-2.1166	3.3866
N	2.0061	-1.5475	2.4201

TS2-Ap$_{anti}$

Ru	1.5021	-0.5492	0.7298
C	3.7712	-2.5081	-0.1732
C	2.8156	-1.5333	-0.4905
C	2.7447	-1.1454	-1.8591
C	3.5746	-1.6820	-2.8528
C	4.5081	-2.6466	-2.4939
C	4.5975	-3.0580	-1.1573
H	3.8769	-2.8462	0.8533
H	3.4795	-1.3460	-3.8806
H	5.1607	-3.0819	-3.2434
H	5.3246	-3.8177	-0.8829
C	0.1567	1.4036	-1.6049
C	0.3034	1.2397	-3.1426
H	0.4850	2.1750	-3.6706
C	1.7323	-0.1378	-2.1094
N	1.0136	0.3039	-1.1230
H	-0.8704	1.2320	-1.2841
O	1.4829	0.3920	-3.3137
C	0.6230	2.7474	-1.0813
C	-0.2600	3.5682	-0.3747
C	1.9264	3.1959	-1.3257
C	0.1561	4.8160	0.0948
H	-1.2759	3.2267	-0.2056
C	2.3387	4.4478	-0.8705
H	2.6177	2.5692	-1.8822
C	1.4565	5.2586	-0.1501
H	-0.5403	5.4512	0.6346
H	3.3418	4.8003	-1.0933
H	1.7757	6.2357	0.1991
H	-0.5443	0.7123	-3.5874
C	-0.6228	1.5276	2.2582
N	0.1396	0.7712	1.8304
C	-1.5949	2.4830	2.7641
H	-1.1814	3.4931	2.7011
H	-2.4932	2.4220	2.1442
H	-1.8460	2.2570	3.8036
C	3.7131	1.8109	1.1932
N	2.9575	0.9493	1.0438
C	4.6442	2.9142	1.3735
H	5.0488	2.9063	2.3891
H	5.4700	2.8255	0.6622
H	4.1197	3.8569	1.1965
C	-5.0664	0.2941	-0.0959
C	-5.3714	-1.0504	-0.3536
C	-6.6750	-1.5189	-0.3054
C	-7.6798	-0.5957	0.0143
C	-7.3760	0.7469	0.2698
C	-6.0561	1.2138	0.2158
C	-3.5995	0.4893	-0.2154
C	-4.1115	-1.7788	-0.6476
H	-6.9006	-2.5607	-0.5044
H	-8.7119	-0.9261	0.0664
H	-8.1775	1.4360	0.5146
H	-5.8125	2.2526	0.4104
O	-2.9106	1.4621	0.0254
O	-3.8722	-2.9504	-0.7965
O	-1.7945	-1.0735	-0.6799
C	-1.3820	-1.4667	0.5983
O	-2.0742	-1.3486	1.5719
C	0.0293	-1.9483	0.5983
H	0.1136	-2.7862	1.2941
N	0.1388	-2.8271	-0.8331
N	0.6628	-3.0950	-1.7791
N	-3.1313	-0.7396	-0.7143
C	2.9339	-2.7537	4.5778
H	3.8197	-2.2578	4.9848
H	3.1888	-3.7906	4.3406
H	2.1462	-2.7482	5.3365
C	2.4814	-2.0609	3.3789
N	2.1293	-1.5071	2.4237

Int2-Ap$_{anti}$

Ru	1.4893	-0.7014	0.6800
C	3.4257	-2.8915	-0.4939
C	2.5707	-1.8091	-0.6989
C	2.4030	-1.3656	-2.0346
C	3.0551	-1.9621	-3.1193
C	3.9091	-3.0351	-2.8793
C	4.0884	-3.4941	-1.5709
H	3.5853	-3.2799	0.5072
H	2.8897	-1.5901	-4.1255
H	4.4273	-3.5153	-3.7025
H	4.7512	-4.3354	-1.3875
C	0.0847	1.3952	-1.4718
C	0.0631	1.2479	-3.0188
H	0.2757	2.1744	-3.5511
C	1.4802	-0.2562	-2.1563
N	0.9282	0.2480	-1.0927
H	-0.9109	1.2601	-1.0510
O	1.1379	0.3028	-3.3196
C	0.6502	2.7131	-0.9857
C	-0.1382	3.5618	-0.2042
C	1.9419	3.1163	-1.3434
C	0.3608	4.7917	0.2300
H	-1.1491	3.2565	0.0481
C	2.4366	4.3505	-0.9231
H	2.5569	2.4676	-1.9610
C	1.6502	5.1882	-0.1266
H	-0.2626	5.4490	0.8292
H	3.4271	4.6706	-1.2341
H	2.0336	6.1514	0.1955
H	-0.8676	0.8064	-3.3823

C	-0.4057	1.4545	2.3860
N	0.3442	0.7150	1.9100
C	-1.3638	2.3876	2.9553
H	-0.9738	3.4056	2.8743
H	-2.2953	2.3088	2.3881
H	-1.5470	2.1504	4.0063
C	4.0047	1.5952	0.8484
N	3.1870	0.7802	0.8146
C	5.0164	2.6399	0.8871
H	5.5789	2.5852	1.8228
H	5.7082	2.5187	0.0491
H	4.5265	3.6140	0.8108
C	-4.9930	0.2309	0.0153
C	-5.3030	-1.0699	-0.4067
C	-6.6107	-1.5278	-0.4389
C	-7.6144	-0.6398	-0.0289
C	-7.3055	0.6596	0.3914
C	-5.9815	1.1165	0.4175
C	-3.5233	0.4218	-0.0531
C	-4.0437	-1.7727	-0.7639
H	-6.8399	-2.5363	-0.7649
H	-8.6498	-0.9641	-0.0349
H	-8.1063	1.3220	0.7031
H	-5.7337	2.1218	0.7405
O	-2.8298	1.3523	0.3156
O	-3.8186	-2.9193	-1.0532
O	-1.7114	-1.0778	-0.6533
C	-1.3286	-1.5629	0.5957
O	-2.0778	-1.6130	1.5371
C	0.1019	-1.9374	0.6460
H	0.2451	-3.0057	0.8458
N	-3.0528	-0.7412	-0.6879
C	3.4341	-2.9783	4.2744
H	2.8078	-3.8271	4.5633
H	3.5474	-2.3133	5.1350
H	4.4193	-3.3489	3.9777
C	2.8158	-2.2627	3.1673
N	2.3273	-1.6989	2.2830

Int1-Ap$_{syn}$

Ru	1.2727	0.7657	0.6878
C	-0.7768	2.5405	-0.8900
C	0.4192	1.8137	-0.8675
C	1.1927	1.8349	-2.0592
C	0.8023	2.5245	-3.2171
C	-0.3966	3.2256	-3.2026
C	-1.1767	3.2295	-2.0381
H	-1.4252	2.5481	-0.0220
H	1.4326	2.5056	-4.1009
H	-0.7258	3.7658	-4.0841
H	-2.1157	3.7771	-2.0290
C	3.9606	-0.2604	-0.9758
C	4.4537	0.2334	-2.3668
H	4.7165	-0.5755	-3.0486
C	2.4224	1.0894	-1.9428
N	2.7078	0.4936	-0.8211
H	4.6617	0.0284	-0.1873
O	3.3176	0.9486	-2.9327
C	3.7079	-1.7518	-0.8911
C	4.2951	-2.5205	0.1153
C	2.8602	-2.3763	-1.8172
C	4.0335	-3.8896	0.2077
H	4.9649	-2.0441	0.8262
C	2.5989	-3.7430	-1.7309
H	2.4107	-1.7909	-2.6156
C	3.1804	-4.5024	-0.7119
H	4.5024	-4.4807	0.9888
H	1.9449	-4.2159	-2.4566
H	2.9771	-5.5659	-0.6418
H	5.2850	0.9382	-2.2899
C	2.9028	-1.0772	2.9290
N	2.3494	-0.3627	2.2029
C	3.6037	-1.9938	3.8174
H	3.8186	-2.9233	3.2826
H	4.5437	-1.5499	4.1571
H	2.9855	-2.2219	4.6899
C	2.6074	3.5686	1.3957
N	2.1605	2.5190	1.1990
C	3.1559	4.8962	1.6364
H	2.8738	5.2452	2.6337
H	4.2469	4.8748	1.5648
H	2.7661	5.5974	0.8929
C	-5.3637	-0.0390	0.0642
C	-5.3618	-1.2477	-0.6460
C	-6.5179	-1.9954	-0.8059
C	-7.6931	-1.4963	-0.2279
C	-7.6957	-0.2886	0.4800
C	-6.5229	0.4622	0.6359
C	-3.9869	0.5233	0.0769
C	-3.9823	-1.5183	-1.1261
H	-6.5054	-2.9300	-1.3557
H	-8.6176	-2.0551	-0.3294
H	-8.6225	0.0705	0.9152
H	-6.5159	1.3997	1.1812
O	-3.5380	1.4828	0.6685
O	-3.4916	-2.4785	-1.6662
O	-1.9004	-0.3085	-0.8917
C	-1.2808	-0.9719	0.1499
O	-1.8522	-1.4176	1.1098
C	0.1837	-1.0175	-0.1279
N	-3.2723	-0.3308	-0.7845
C	-2.3437	1.4202	3.5386
H	-3.1676	1.7979	2.9279
H	-2.1348	2.1138	4.3574
H	-2.6262	0.4486	3.9540
C	-1.1718	1.2627	2.6918
N	-0.2560	1.1196	1.9978
N	0.7214	-2.0899	0.5512
N	1.1476	-2.8939	1.2024
H	0.4750	-1.0549	-1.1737

TS2-Ap$_{syn}$

Ru	1.2389	0.7125	0.6971
C	-0.7416	2.6055	-0.8545
C	0.4392	1.8572	-0.8373
C	1.2589	1.9321	-1.9931
C	0.9292	2.7055	-3.1154
C	-0.2534	3.4351	-3.0980
C	-1.0800	3.3801	-1.9681
H	-1.4254	2.5647	-0.0146
H	1.5919	2.7283	-3.9750
H	-0.5354	4.0400	-3.9534
H	-2.0079	3.9461	-1.9598
C	3.9492	-0.2784	-0.9341
C	4.4798	0.2464	-2.3019
H	4.7024	-0.5492	-3.0135
C	2.4656	1.1465	-1.8813
N	2.7000	0.4817	-0.7880
H	4.6330	-0.0109	-0.1227
O	3.3900	1.0454	-2.8472
C	3.6960	-1.7712	-0.8899
C	4.2966	-2.5674	0.0866
C	2.8462	-2.3717	-1.8289
C	4.0488	-3.9418	0.1346
H	4.9687	-2.1094	0.8077
C	2.5996	-3.7434	-1.7875
H	2.3832	-1.7639	-2.6021
C	3.1973	-4.5316	-0.8009
H	4.5298	-4.5541	0.8916
H	1.9435	-4.1971	-2.5235
H	3.0063	-5.5992	-0.7670
H	5.3484	0.8996	-2.1939
C	2.8311	-1.1788	2.8942
N	2.2993	-0.4264	2.1905
C	3.5099	-2.1329	3.7594
H	3.7733	-3.0217	3.1795
H	4.4199	-1.6891	4.1725
H	2.8542	-2.4281	4.5830
C	2.5803	3.5860	1.4806
N	2.1617	2.5248	1.2907
C	3.0926	4.9301	1.7078
H	2.8172	5.2740	2.7085
H	4.1821	4.9401	1.6158
H	2.6675	5.6139	0.9676
C	-5.3663	-0.0560	0.0423
C	-5.3526	-1.2435	-0.7025
C	-6.4982	-2.0048	-0.8722
C	-7.6749	-1.5421	-0.2676
C	-7.6894	-0.3555	0.4750
C	-6.5271	0.4096	0.6402
C	-3.9981	0.5265	0.0554
C	-3.9741	-1.4773	-1.2063
H	-6.4766	-2.9227	-1.4492
H	-8.5914	-2.1128	-0.3755
H	-8.6171	-0.0242	0.9300
H	-6.5295	1.3313	1.2119
O	-3.5554	1.4754	0.6703
O	-3.4776	-2.4122	-1.7838
O	-1.9084	-0.2373	-0.9465
C	-1.2686	-0.9127	0.0798
O	-1.8435	-1.4373	0.9965
C	0.2113	-0.8166	-0.1522
N	-3.2798	-0.2888	-0.8372
C	-2.3774	1.3937	3.5520
H	-3.2104	1.7260	2.9272
H	-2.1751	2.1338	4.3308
H	-2.6398	0.4415	4.0218
C	-1.2091	1.2143	2.7048

N	-0.2961	1.0586	2.0107
N	0.6792	-2.2823	0.5249
N	1.1692	-2.9400	1.2768
H	0.4734	-1.0277	-1.1893

Int2-Ap$_{syn}$

Ru	-1.1400	-0.4669	0.6164
C	0.4720	-2.8329	-0.6831
C	-0.6406	-1.9937	-0.7044
C	-1.5941	-2.1846	-1.7327
C	-1.4672	-3.1946	-2.6951
C	-0.3582	-4.0327	-2.6400
C	0.6062	-3.8430	-1.6431
H	1.2558	-2.6875	0.0523
H	-2.2217	-3.3099	-3.4669
H	-0.2342	-4.8210	-3.3750
H	1.4817	-4.4860	-1.6199
C	-3.9695	0.4228	-0.8466
C	-4.5344	-0.0843	-2.2009
H	-4.3822	0.6407	-3.0049
C	-2.6821	-1.2321	-1.6892
N	-2.6855	-0.2939	-0.7865
H	-4.5927	0.0729	-0.0133
O	-3.7379	-1.2680	-2.5104
C	-3.8403	1.9240	-0.7514
C	-4.6605	2.6438	0.1210
C	-2.9176	2.6152	-1.5449
C	-4.5606	4.0349	0.2037
H	-5.3861	2.1139	0.7332
C	-2.8024	4.0008	-1.4526
H	-2.2887	2.0646	-2.2381
C	-3.6239	4.7148	-0.5763
H	-5.2200	4.5861	0.8679
H	-2.0782	4.5250	-2.0679
H	-3.5417	5.7951	-0.5120
H	-5.5814	-0.3828	-2.1629
C	-2.4243	1.8473	2.6293
N	-2.0266	0.9398	2.0345
C	-2.9179	3.0143	3.3431
H	-3.3146	3.7288	2.6164
H	-3.7125	2.7249	4.0361
H	-2.1043	3.4794	3.9061
C	-2.9038	-3.0677	1.9508
N	-2.3733	-2.0939	1.6242
C	-3.5604	-4.3042	2.3508
H	-3.2292	-4.5983	3.3503
H	-4.6453	-4.1693	2.3593
H	-3.3054	-5.0978	1.6429
C	5.4715	0.0965	0.0219
C	5.4831	1.2408	-0.7882
C	6.6301	2.0015	-0.9490
C	7.7815	1.5846	-0.2671
C	7.7708	0.4414	0.5405
C	6.6075	-0.3243	0.6958
C	4.1072	-0.4949	0.0025
C	4.1277	1.4330	-1.3717
H	6.6281	2.8853	-1.5775
H	8.6979	2.1571	-0.3659
H	8.6794	0.1444	1.0540
H	6.5913	-1.2136	1.3165
O	3.6373	-1.4167	0.6413
O	3.6623	2.3222	-2.0392
O	2.0590	0.1679	-1.1125
C	1.3514	0.9053	-0.1615
O	1.8773	1.5661	0.6937
C	-0.0946	0.6833	-0.3934
N	3.4232	0.2676	-0.9557
C	2.4500	-1.2603	3.4928
H	3.2914	-1.5824	2.8737
H	2.2153	-2.0277	4.2353
H	2.7128	-0.3315	4.0068
C	1.3072	-1.0319	2.6237
N	0.4114	-0.8407	1.9181
H	-0.4675	1.1696	-1.3008

Int1-Eq$_{trans}$

Ru	-1.3540	-0.6309	0.2977
C	-2.0908	-3.1135	-1.5301
C	-2.3774	-1.8330	-1.0409
C	-3.5588	-1.2325	-1.5607
C	-4.4065	-1.8581	-2.4837
C	-4.0876	-3.1357	-2.9292
C	-2.9271	-3.7537	-2.4518
H	-1.1955	-3.6459	-1.2180
H	-5.2934	-1.3438	-2.8412
H	-4.7258	-3.6446	-3.6436
H	-2.6673	-4.7475	-2.8066
C	-3.4110	1.9345	0.2217
C	-4.6306	2.1488	-0.7227
H	-4.4096	2.8682	-1.5152
C	-3.7839	0.1004	-1.0532
N	-2.9199	0.6313	-0.2406
H	-3.7381	1.8341	1.2646
O	-4.8542	0.8505	-1.3480
C	-2.3847	3.0404	0.1419
C	-2.2879	3.9851	1.1664
C	-1.5445	3.1502	-0.9712
C	-1.3694	5.0339	1.0786
H	-2.9365	3.9040	2.0347
C	-0.6132	4.1833	-1.0520
H	-1.6133	2.4104	-1.7611
C	-0.5259	5.1315	-0.0295
H	-1.3162	5.7736	1.8719
H	0.0402	4.2565	-1.9161
H	0.1907	5.9440	-0.0990
H	-5.5456	2.4365	-0.2050
C	-3.2522	-1.7328	2.6031
N	-2.5251	-1.3352	1.7918
C	-4.1719	-2.2324	3.6158
H	-4.5567	-3.2134	3.3228
H	-3.6608	-2.3238	4.5785
H	-5.0143	-1.5438	3.7283
C	0.4873	0.4432	-2.0584
N	-0.2328	0.0687	-1.2350
C	1.4334	0.9036	-3.0608
H	2.0023	0.0493	-3.4386
H	0.9111	1.3827	-3.8931
H	2.1221	1.6120	-2.5929
C	5.6194	-0.7725	0.0232
C	5.1070	0.4542	-0.4241
C	5.8818	1.3354	-1.1631
C	7.1977	0.9507	-1.4526
C	7.7080	-0.2745	-1.0069
C	6.9213	-1.1580	-0.2555
C	4.5589	-1.4904	0.7746
C	3.6952	0.5827	0.0186
H	5.4796	2.2837	-1.5028
H	7.8330	1.6119	-2.0326
H	8.7305	-0.5441	-1.2497
H	7.3076	-2.1094	0.0934
O	4.5067	-2.6034	1.2326
O	2.8628	1.4318	-0.2358
O	2.2420	-0.9402	1.2333
C	1.6054	-1.6825	0.2356
O	2.0545	-1.8429	-0.8649
C	0.3006	-2.1813	0.7203
N	3.5010	-0.5313	0.8570
C	1.2448	2.7807	2.2913
H	0.6698	3.7094	2.3148
H	2.0402	2.8651	1.5461
H	1.6822	2.5917	3.2752
C	0.3747	1.6841	1.9015
N	-0.3098	0.8191	1.5518
N	0.2147	-2.3041	2.0787
N	0.0608	-2.3607	3.1873
H	-0.0493	-3.0866	0.2348

TS2-Eq$_{trans}$

Ru	-1.3056	-0.6689	0.3451
C	-1.7744	-3.0192	-1.7152
C	-2.1895	-1.8144	-1.1366
C	-3.3937	-1.2633	-1.6560
C	-4.1504	-1.8781	-2.6621
C	-3.7112	-3.0852	-3.1938
C	-2.5198	-3.6442	-2.7214
H	-0.8444	-3.4904	-1.4104
H	-5.0614	-1.4065	-3.0176
H	-4.2793	-3.5812	-3.9736
H	-2.1620	-4.5780	-3.1468
C	-3.5589	1.8243	0.2667
C	-4.7873	1.9649	-0.6854
H	-4.6574	2.7675	-1.4149
C	-3.7486	0.0130	-1.0755
N	-2.9807	0.5527	-0.1810
H	-3.8848	1.7168	1.3082
O	-4.8461	0.7043	-1.4149
C	-2.5826	2.9767	0.1849
C	-2.4204	3.8460	1.2656
C	-1.8434	3.1949	-0.9833
C	-1.5363	4.9249	1.1821
H	-2.9922	3.6829	2.1753
C	-0.9500	4.2605	-1.0648
H	-1.9637	2.5164	-1.8220
C	-0.7946	5.1306	0.0178
H	-1.4346	5.6065	2.0214
H	-0.3815	4.4238	-1.9756
H	-0.1081	5.9686	-0.0506

H	-5.7341	2.0942	-0.1601
C	-3.2103	-2.1302	2.4343
N	-2.4848	-1.6000	1.7025
C	-4.1319	-2.7938	3.3458
H	-4.1934	-3.8594	3.1073
H	-3.7916	-2.6787	4.3788
H	-5.1291	-2.3548	3.2503
C	0.4534	0.8251	-1.8505
N	-0.2075	0.2855	-1.0715
C	1.3046	1.5044	-2.8121
H	1.7756	0.7690	-3.4702
H	0.7151	2.1989	-3.4168
H	2.0790	2.0498	-2.2667
C	5.6174	-0.7520	-0.0598
C	5.1336	0.5199	-0.3998
C	5.9223	1.4358	-1.0792
C	7.2224	1.0399	-1.4206
C	7.7041	-0.2302	-1.0822
C	6.9035	-1.1490	-0.3896
C	4.5496	-1.4979	0.6547
C	3.7331	0.6504	0.0777
H	5.5427	2.4191	-1.3353
H	7.8678	1.7278	-1.9568
H	8.7149	-0.5073	-1.3625
H	7.2678	-2.1350	-0.1231
O	4.4822	-2.6367	1.0419
O	2.9248	1.5466	-0.0815
O	2.2530	-0.9133	1.1982
C	1.5753	-1.6051	0.1864
O	2.0154	-1.7492	-0.9205
C	0.2154	-2.0198	0.6281
N	3.5144	-0.5258	0.8119
C	1.1398	2.7604	2.4467
H	0.5930	3.6993	2.3278
H	2.0015	2.7537	1.7731
H	1.4776	2.6587	3.4813
C	0.2738	1.6534	2.0784
N	-0.4021	0.7789	1.7353
N	0.3710	-2.2537	2.2811
N	0.0039	-2.3002	3.3298
H	0.0264	-3.0628	0.3675

Int2-Eq$_{trans}$

Ru	-0.9989	-0.6842	0.5795
C	-0.4148	-3.2992	-1.0330
C	-1.3035	-2.2660	-0.7211
C	-2.5550	-2.2698	-1.3848
C	-2.9144	-3.2680	-2.3013
C	-2.0153	-4.2905	-2.5800
C	-0.7680	-4.2965	-1.9479
H	0.5705	-3.3242	-0.5805
H	-3.8862	-3.2283	-2.7828
H	-2.2765	-5.0719	-3.2857
H	-0.0571	-5.0872	-2.1709
C	-4.1614	0.7080	-0.0495
C	-5.1958	0.2148	-1.1064
H	-5.2417	0.8795	-1.9730
C	-3.4286	-1.1596	-1.0650
N	-3.0400	-0.2120	-0.2724
H	-4.5457	0.5512	0.9671
O	-4.6763	-1.0690	-1.5525
C	-3.8116	2.1702	-0.2118
C	-4.5255	3.1385	0.5013
C	-2.8204	2.5765	-1.1116
C	-4.2668	4.4970	0.3071
H	-5.2934	2.8300	1.2063
C	-2.5482	3.9318	-1.2942
H	-2.2579	1.8227	-1.6508
C	-3.2754	4.8971	-0.5915
H	-4.8369	5.2398	0.8564
H	-1.7738	4.2390	-1.9908
H	-3.0725	5.9523	-0.7455
H	-6.1975	0.0563	-0.7068
C	-2.4357	-2.4377	2.8283
N	-1.9213	-1.7571	2.0476
C	-3.0848	-3.2995	3.8057
H	-2.8981	-4.3479	3.5565
H	-2.6924	-3.0953	4.8057
H	-4.1637	-3.1214	3.8033
C	0.4338	0.7187	-1.9048
N	-0.0825	0.2774	-0.9698
C	1.0749	1.2587	-3.0919
H	0.4551	2.0476	-3.5270
H	2.0479	1.6651	-2.8052
H	1.2086	0.4616	-3.8286
C	5.9894	0.0657	0.4678
C	5.5008	0.9676	-0.4881
C	6.3360	1.5420	-1.4338
C	7.6903	1.1836	-1.4012
C	8.1774	0.2824	-0.4473
C	7.3278	-0.2916	0.5082
C	4.8628	-0.3797	1.3299
C	4.0379	1.1408	-0.2871
H	5.9502	2.2394	-2.1693
H	8.3740	1.6097	-2.1281
H	9.2310	0.0232	-0.4501
H	7.6950	-0.9919	1.2503
O	4.7971	-1.2154	2.1966
O	3.2147	1.7445	-0.9445
O	2.4857	0.1444	1.2980
C	1.9677	-0.9748	0.6506
O	2.5364	-1.5564	-0.2353
C	0.6421	-1.3046	1.2175
H	0.7156	-2.0063	2.0577
N	3.7750	0.4332	0.8984
C	-1.3826	3.6946	2.4272
H	-1.8571	4.2654	1.6244
H	-0.3920	4.1073	2.6365
H	-1.9974	3.7627	3.3284
C	-1.2576	2.3086	2.0083
N	-1.1555	1.2138	1.6541

Int1-Eq$_{cis}$

Ru	1.2929	-0.5897	0.2555
C	2.5500	-3.3949	0.6111
C	2.5772	-2.1437	-0.0097
C	3.6406	-1.8929	-0.9153
C	4.6356	-2.8393	-1.1934
C	4.5805	-4.0757	-0.5596
C	3.5393	-4.3456	0.3369
H	1.7580	-3.6363	1.3126
H	5.4288	-2.6021	-1.8956
H	5.3378	-4.8265	-0.7588
H	3.4991	-5.3134	0.8298
C	2.6480	1.4079	-2.0435
C	4.0550	1.2647	-2.6818
H	4.7968	1.8954	-2.1830
C	3.5672	-0.5832	-1.5179
N	2.6016	0.2189	-1.1806
H	1.8670	1.3099	-2.8092
O	4.4197	-0.1281	-2.4478
C	2.4143	2.6890	-1.2805
C	1.5292	3.6547	-1.7706
C	3.0362	2.8966	-0.0425
C	1.2591	4.8092	-1.0313
H	1.0361	3.4950	-2.7257
C	2.7628	4.0444	0.6986
H	3.7102	2.1396	0.3440
C	1.8702	5.0022	0.2077
H	0.5669	5.5493	-1.4193
H	3.2433	4.1959	1.6607
H	1.6553	5.8940	0.7876
H	4.0760	1.4444	-3.7560
C	-0.7587	-1.5922	-1.9528
N	0.0723	-1.2569	-1.2215
C	-1.8464	-1.9814	-2.8346
H	-2.3041	-1.0827	-3.2574
H	-1.4811	-2.6212	-3.6419
H	-2.5954	-2.5170	-2.2452
C	3.1174	0.3965	2.6619
N	2.4585	0.0626	1.7685
C	3.9475	0.8117	3.7834
H	3.3170	1.1408	4.6143
H	4.5686	-0.0240	4.1186
H	4.6002	1.6376	3.4859
C	-5.2841	-0.7618	-0.2813
C	-5.6569	0.5722	-0.0628
C	-6.9051	1.0454	-0.4365
C	-7.7819	0.1382	-1.0466
C	-7.4107	-1.1943	-1.2638
C	-6.1489	-1.6664	-0.8790
C	-3.8994	-0.9664	0.2146
C	-4.5257	1.2862	0.5818
H	-7.1835	2.0792	-0.2643
H	-8.7659	0.4731	-1.3578
H	-8.1133	-1.8713	-1.7385
H	-5.8549	-2.6979	-1.0405
O	-3.1675	-1.9335	0.1271
O	-4.3497	2.4506	0.8363
O	-2.2893	0.5731	1.1826
C	-1.5741	1.0342	0.0651
O	-2.0106	0.9937	-1.0568
C	-0.2612	1.5178	0.4570
H	0.3167	2.1138	-0.2391
N	-0.0340	1.7952	1.7386
N	0.2144	2.0026	2.8192
N	-3.5866	0.2442	0.8631
C	-1.8143	-2.7232	2.9584

H	-2.5972	-3.0321	2.2609
H	-2.2407	-2.0315	3.6905
H	-1.4194	-3.5977	3.4825
C	-0.7625	-2.0631	2.2020
N	0.0553	-1.5374	1.5729

TS2-Eq$_{cis}$

Ru	1.0471	-0.5768	0.2885
C	2.1870	-3.4258	1.0999
C	2.2612	-2.2934	0.2860
C	3.2800	-2.2770	-0.6970
C	4.1907	-3.3274	-0.8692
C	4.0928	-4.4372	-0.0358
C	3.0939	-4.4807	0.9442
H	1.4203	-3.4946	1.8667
H	4.9541	-3.2692	-1.6390
H	4.7852	-5.2652	-0.1459
H	3.0218	-5.3506	1.5919
C	2.6662	1.0133	-2.1331
C	3.7806	0.4526	-3.0639
H	4.6635	1.0897	-3.1157
C	3.2790	-1.0696	-1.4927
N	2.3871	-0.1490	-1.2749
H	1.7729	1.2634	-2.7141
O	4.1737	-0.8123	-2.4556
C	3.0782	2.2182	-1.3106
C	2.3428	3.4048	-1.3623
C	4.2038	2.1502	-0.4802
C	2.7096	4.5053	-0.5838
H	1.4837	3.4768	-2.0255
C	4.5860	3.2534	0.2807
H	4.7840	1.2329	-0.4335
C	3.8328	4.4312	0.2395
H	2.1283	5.4206	-0.6329
H	5.4787	3.2004	0.8972
H	4.1310	5.2903	0.8322
H	3.4171	0.2369	-4.0710
C	-1.0223	-2.1750	-1.5411
N	-0.1843	-1.6306	-0.9623
C	-2.1223	-2.8241	-2.2340
H	-2.4166	-2.2229	-3.0985
H	-1.8321	-3.8237	-2.5673
H	-2.9657	-2.8929	-1.5409
C	2.8791	0.9338	2.3792
N	2.2516	0.3468	1.6023
C	3.6910	1.6539	3.3471
H	3.1335	1.7911	4.2775
H	4.6063	1.0937	3.5583
H	3.9539	2.6322	2.9369
C	-5.4770	-0.3179	-0.1348
C	-5.6742	1.0648	-0.2581
C	-6.8747	1.5869	-0.7135
C	-7.8863	0.6775	-1.0504
C	-7.6906	-0.7034	-0.9261
C	-6.4755	-1.2233	-0.4612
C	-4.1018	-0.5681	0.3659
C	-4.4309	1.7645	0.1555
H	-7.0167	2.6579	-0.8071
H	-8.8383	1.0483	-1.4156
H	-8.4943	-1.3806	-1.1956
H	-6.3165	-2.2915	-0.3600
O	-3.4954	-1.6132	0.5113
O	-4.1027	2.9221	0.0953
O	-2.2654	0.9373	0.8720
C	-1.5579	0.9312	-0.3330
O	-2.0732	0.6571	-1.3887
C	-0.1051	1.1252	-0.1449
H	0.2730	1.8147	-0.8998
N	0.0060	2.3413	1.1739
N	0.6463	2.7595	1.9849
N	-3.6118	0.7143	0.6776
C	-2.0714	-2.0458	3.4003
H	-2.9325	-2.3205	2.7853
H	-2.3642	-1.2552	4.0968
H	-1.7311	-2.9150	3.9696
C	-1.0182	-1.5704	2.5177
N	-0.2020	-1.1941	1.7897

Int2-Eq$_{cis}$

Ru	-1.4935	-0.5560	0.4104
C	-3.4380	-2.5946	-1.1898
C	-3.1573	-1.3022	-0.7389
C	-4.0969	-0.2984	-1.0804
C	-5.2548	-0.5490	-1.8290
C	-5.5014	-1.8498	-2.2547
C	-4.5934	-2.8656	-1.9322
H	-2.7557	-3.4100	-0.9634
H	-5.9395	0.2593	-2.0675
H	-6.3899	-2.0756	-2.8350
H	-4.7901	-3.8803	-2.2688
C	-2.4771	2.5504	0.5267
C	-3.7063	3.2166	-0.1611
H	-3.4179	3.9009	-0.9611
C	-3.7295	1.0068	-0.5798
N	-2.6330	1.1517	0.1051
H	-2.5679	2.6015	1.6182
O	-4.4517	2.1158	-0.7642
C	-1.1377	3.1197	0.1231
C	-0.2728	3.6470	1.0856
C	-0.7497	3.1257	-1.2229
C	0.9624	4.1811	0.7129
H	-0.5732	3.6531	2.1305
C	0.4785	3.6629	-1.5962
H	-1.4105	2.7031	-1.9745
C	1.3376	4.1914	-0.6282
H	1.6264	4.5869	1.4691
H	0.7650	3.6803	-2.6438
H	2.2955	4.6087	-0.9212
H	-4.3707	3.7255	0.5378
C	-3.2713	-1.0935	3.0131
N	-2.5886	-0.8860	2.1037
C	-4.1375	-1.3696	4.1502
H	-3.6060	-1.9798	4.8857
H	-4.4471	-0.4335	4.6230
H	-5.0274	-1.9097	3.8148
C	0.3189	-0.0892	-2.1533
N	-0.4512	-0.2651	-1.3123
C	1.3510	0.1553	-3.1442
H	2.1330	-0.5987	-3.0173
H	0.9435	0.1039	-4.1568
H	1.7747	1.1474	-2.9640
C	5.3950	-0.1238	0.5320
C	4.9473	-0.9353	-0.5197
C	5.7700	-1.2530	-1.5900
C	7.0683	-0.7263	-1.5839
C	7.5147	0.0849	-0.5336
C	6.6792	0.3974	0.5474
C	4.2916	0.0395	1.5148
C	3.5397	-1.3284	-0.2576
H	5.4173	-1.8849	-2.3981
H	7.7404	-0.9497	-2.4059
H	8.5254	0.4786	-0.5592
H	7.0159	1.0249	1.3653
O	4.1868	0.7390	2.4896
O	2.7453	-1.9232	-0.9645
O	1.9927	-0.7596	1.5138
C	1.3218	0.3395	0.9455
O	1.8427	1.0594	0.1319
C	-0.0709	0.3453	1.4100
H	-0.2105	0.9094	2.3385
N	3.2863	-0.8627	1.0439
C	1.0305	-4.4573	0.2022
H	0.5355	-5.2649	-0.3437
H	1.3427	-4.8252	1.1832
H	1.9068	-4.1153	-0.3557
C	0.1320	-3.3256	0.3555
N	-0.5549	-2.4031	0.4624

Side Reactions.

Int3-DM-Ap_{anti}

Ru 0.3783 1.2837 -0.3797
C -0.8918 4.1216 -0.3944
C -0.0338 3.2091 0.2183
C 0.7081 3.6479 1.3410
C 0.6059 4.9505 1.8404
C -0.2529 5.8451 1.2073
C -0.9958 5.4281 0.0977
H -1.4899 3.8214 -1.2476
H 1.1889 5.2472 2.7064
H -0.3507 6.8601 1.5776
H -1.6718 6.1276 -0.3860
C 2.4896 0.5628 2.1045
C 2.9265 1.4953 3.2795
H 4.0031 1.6672 3.3057
C 1.5548 2.6168 1.9030
N 1.6130 1.4533 1.3245
H 1.9000 -0.2779 2.4740
O 2.2787 2.7778 3.0104
C 3.6689 0.0222 1.3270
C 4.1144 -1.2805 1.5738
C 4.3850 0.8384 0.4441
C 5.2822 -1.7510 0.9704
H 3.5405 -1.9221 2.2350
C 5.5443 0.3634 -0.1663
H 4.0292 1.8432 0.2414
C 6.0020 -0.9288 0.1047
H 5.6238 -2.7610 1.1718
H 6.1001 1.0031 -0.8460
H 6.9087 -1.2951 -0.3655
H 2.5792 1.1500 4.2546
C -1.7019 1.2610 -2.8325
N -0.8882 1.2947 -2.0117
C -2.7634 1.1998 -3.8233
H -3.7170 1.3669 -3.3129
H -2.6140 1.9643 -4.5903
H -2.7702 0.2133 -4.2951
C 2.8431 3.0634 -2.2686
N 2.0794 2.3873 -1.7253
C 3.8083 3.9191 -2.9451
H 4.8238 3.6449 -2.6467
H 3.7130 3.8099 -4.0286
H 3.6288 4.9634 -2.6753
C -6.1234 -1.4405 0.1528
C -6.1948 -0.4084 -0.7940
C -7.2003 -0.3704 -1.7473
C -8.1439 -1.4066 -1.7311
C -8.0710 -2.4375 -0.7870
C -7.0526 -2.4685 0.1755
C -4.9390 -1.2120 1.0199
C -5.0590 0.5238 -0.5722
H -7.2509 0.4329 -2.4742
H -8.9451 -1.4116 -2.4627
H -8.8160 -3.2262 -0.8024
H -6.9874 -3.2634 0.9103
O -4.4210 -1.9162 1.8581
O -4.6524 1.4536 -1.2354
O -3.2447 0.5219 1.0400
C -2.2104 -0.0040 0.2542
O -2.4140 -0.7895 -0.6356
C -0.9321 0.5851 0.7147
N -4.4851 0.0753 0.6402
C 3.1571 -2.3126 -1.5952
C 3.2495 -3.6047 -1.0615
C 4.3107 -4.4393 -1.3703
C 5.2835 -3.9439 -2.2511
C 5.1859 -2.6558 -2.7880
C 4.1159 -1.8125 -2.4587
C 1.9452 -1.6530 -1.0573
C 2.0762 -3.8388 -0.1815
H 4.3770 -5.4368 -0.9499
H 6.1251 -4.5719 -2.5244
H 5.9554 -2.3040 -3.4674
H 4.0377 -0.8056 -2.8514
O 1.5244 -0.5353 -1.3300
O 1.7057 -4.8064 0.4303
O 0.0970 -2.4917 0.2485
C 0.0023 -2.3789 1.6488
O 0.9614 -2.2251 2.3702
C -1.3886 -2.4491 1.9889
H -2.1983 -2.6294 1.2998
N -1.7138 -2.2794 3.2520
N -1.9986 -2.1225 4.3343
N 1.3909 -2.5802 -0.1919
H -0.8636 0.6554 1.8060

TS-DM-Ap_{anti}

Ru -0.1786 0.7173 0.4214
C 2.1401 2.7695 0.6579
C 1.0144 2.3223 -0.0386
C 0.5767 3.0991 -1.1398
C 1.2308 4.2718 -1.5407
C 2.3487 4.6930 -0.8299
C 2.7953 3.9394 0.2626
H 2.5403 2.1951 1.4838
H 0.8605 4.8326 -2.3931
H 2.8741 5.5960 -1.1227
H 3.6760 4.2628 0.8109
C -2.3074 1.1392 -2.1833
C -2.3331 2.3301 -3.1911
H -3.2041 2.9741 -3.0519
C -0.6005 2.5666 -1.7869
N -1.1753 1.4998 -1.3120
H -2.0701 0.2063 -2.6990
O -1.1451 3.1136 -2.8797
C -3.6241 0.9697 -1.4608
C -4.4994 -0.0513 -1.8439
C -4.0266 1.8860 -0.4821
C -5.7767 -0.1335 -1.2857
H -4.1777 -0.7798 -2.5818
C -5.2980 1.7987 0.0816
H -3.3380 2.6646 -0.1703
C -6.1814 0.7968 -0.3293
H -6.4520 -0.9242 -1.5962
H -5.6072 2.5179 0.8346
H -7.1742 0.7346 0.1039
H -2.2662 2.0184 -4.2341
C 1.6347 -0.2524 2.8704
N 0.9178 0.1028 2.0330
C 2.5833 -0.6956 3.8789
H 3.5597 -0.2597 3.6477
H 2.2620 -0.3799 4.8750
H 2.6624 -1.7862 3.8563
C -1.6437 3.0999 2.2949
N -1.2288 2.2057 1.6914
C -2.1618 4.2329 3.0485
H -2.1593 4.0066 4.1181
H -1.5352 5.1111 2.8698
H -3.1848 4.4576 2.7343
C 6.4093 -1.3953 -0.4016
C 6.3455 -0.7045 0.8173
C 7.3468 -0.8228 1.7686
C 8.4275 -1.6622 1.4668
C 8.4902 -2.3526 0.2505
C 7.4749 -2.2275 -0.7073
C 5.1907 -1.0788 -1.1892
C 5.0854 0.0844 0.8614
H 7.2903 -0.2843 2.7083
H 9.2298 -1.7814 2.1876
H 9.3396 -2.9966 0.0480
H 7.5139 -2.7595 -1.6514
O 4.7397 -1.5595 -2.2020
O 4.5710 0.6945 1.7754
O 3.2710 0.3059 -0.7511
C 2.3676 -0.6705 -0.3489
O 2.7144 -1.6355 0.2936
C 0.9999 -0.3059 -0.8225
N 4.5719 -0.0328 -0.4476
C -4.3829 -1.5978 1.4339
C -4.8305 -2.7400 0.7524
C -6.1257 -3.2092 0.9038
C -6.9671 -2.5073 1.7780
C -6.5157 -1.3747 2.4660
C -5.2103 -0.8969 2.2959
C -2.9860 -1.3237 1.0352
C -3.7238 -3.2771 -0.0845
H -6.4651 -4.0903 0.3705
H -7.9851 -2.8508 1.9297
H -7.1918 -0.8576 3.1390
H -4.8557 -0.0103 2.8081
O -2.1995 -0.4807 1.4275
O -3.6228 -4.2799 -0.7420
O -1.3803 -2.5671 -0.1835
C -0.9594 -2.2150 -1.4456
O -1.6631 -1.9563 -2.3818
C 0.5169 -2.1576 -1.3695
H 1.0181 -2.7072 -0.5758
N 1.1279 -2.3329 -2.5735
N 1.6992 -2.2943 -3.5418
N -2.7073 -2.2830 0.0553
H 1.0323 0.1069 -1.8356

Product-DM-Ap_{anti}

Ru 0.4088 -0.4458 0.5078
C -1.9183 -2.4990 0.5028

C -0.8223 -1.9495 -0.1579
C -0.4633 -2.5012 -1.4136
C -1.2008 -3.5279 -2.0165
C -2.3093 -4.0392 -1.3494
C -2.6551 -3.5283 -0.0944
H -2.2412 -2.1102 1.4583
H -0.8962 -3.9148 -2.9838
H -2.8976 -4.8324 -1.7981
H -3.5194 -3.9267 0.4292
C 2.7127 -0.8275 -1.9263
C 2.4499 -1.4510 -3.3210
H 3.2736 -2.0530 -3.7013
C 0.7531 -1.9432 -1.9564
N 1.4010 -1.0378 -1.2789
H 2.9158 0.2381 -2.0222
O 1.3079 -2.3392 -3.1050
C 3.8376 -1.5050 -1.1699
C 5.0502 -0.8374 -0.9856
C 3.7134 -2.8256 -0.7182
C 6.1235 -1.4681 -0.3530
H 5.1598 0.1819 -1.3445
C 4.7867 -3.4618 -0.0966
H 2.7799 -3.3603 -0.8620
C 5.9942 -2.7821 0.0943
H 7.0568 -0.9322 -0.2153
H 4.6904 -4.4964 0.2207
H 6.8310 -3.2812 0.5729
H 2.1474 -0.6992 -4.0541
C -1.6227 0.3282 2.8597
N -0.7831 0.0390 2.1171
C -2.7266 0.6818 3.7357
H -3.5846 0.0552 3.4741
H -2.4526 0.5309 4.7829
H -2.9915 1.7300 3.5734
C 1.9847 -2.6376 2.1387
N 1.3808 -1.8520 1.5437
C 2.7712 -3.6138 2.8740
H 3.7876 -3.6268 2.4707
H 2.7997 -3.3532 3.9354
H 2.3287 -4.6077 2.7631
C -6.7999 0.7023 -0.3647
C -6.5187 -0.0169 0.8046
C -7.4712 -0.1831 1.7982
C -8.7281 0.4002 1.5901
C -9.0075 1.1208 0.4226
C -8.0406 1.2821 -0.5788
C -5.5873 0.7135 -1.2208
C -5.1136 -0.5004 0.7576
H -7.2460 -0.7431 2.6993
H -9.4989 0.2940 2.3464
H -9.9898 1.5630 0.2932
H -8.2459 1.8400 -1.4858
O -5.3270 1.3083 -2.2363
O -4.4502 -1.0481 1.6140
O -3.3719 -0.2048 -0.9465
C -2.6136 0.7804 -0.3481
O -3.0308 1.4966 0.5292
C -1.2659 0.8333 -0.9601
N -4.6926 -0.1749 -0.5490
C 4.7640 1.7916 1.0712
C 5.1610 2.9085 0.3191
C 6.4802 3.3333 0.2993
C 7.4013 2.6128 1.0722
C 7.0034 1.5023 1.8270
C 5.6717 1.0689 1.8305
C 3.3157 1.5739 0.8603
C 3.9751 3.4758 -0.3835
H 6.7783 4.1954 -0.2872
H 8.4410 2.9227 1.0901
H 7.7412 0.9691 2.4175
H 5.3577 0.1995 2.3961
O 2.5570 0.7568 1.3623
O 3.8388 4.4742 -1.0383
O 1.6124 2.8624 -0.1403
C 0.8946 2.0999 -1.0822
O 1.3216 1.8036 -2.1646
C -0.3797 1.7455 -0.4444
H -0.6636 2.3191 0.4307
N -3.0625 3.6090 -1.7959
N -4.0482 4.0615 -1.5856
N 2.9459 2.5225 -0.0991
H -1.0996 0.3270 -1.9025

Int3-DM-Ap$_{syn}$

Ru -0.5863 1.3114 -0.4690
C 1.6371 3.5127 -0.3892
C 0.4455 3.0017 0.1220
C -0.2010 3.7252 1.1513
C 0.2986 4.9394 1.6384
C 1.4789 5.4410 1.0980
C 2.1445 4.7231 0.0977
H 2.1953 2.9639 -1.1393
H -0.2279 5.4671 2.4274
H 1.8889 6.3776 1.4610
H 3.0782 5.1073 -0.3033
C -2.9708 1.4609 1.8590
C -3.2515 2.6440 2.8382
H -3.2190 2.3393 3.8851
C -1.3893 3.0709 1.6581
N -1.7486 1.9178 1.1777
H -3.7742 1.3698 1.1201
O -2.1638 3.5904 2.6155
C -2.7892 0.1284 2.5524
C -3.7137 -0.9006 2.3634
C -1.7061 -0.0767 3.4156
C -3.5510 -2.1284 3.0074
H -4.5629 -0.7431 1.7027
C -1.5392 -1.3020 4.0592
H -0.9935 0.7260 3.5864
C -2.4592 -2.3333 3.8498
H -4.2649 -2.9277 2.8406
H -0.6993 -1.4482 4.7322
H -2.3297 -3.2890 4.3473
H -4.1893 3.1624 2.6318
C -2.1637 3.7786 -2.2513
N -1.7607 2.8336 -1.7214
C -2.6544 4.9793 -2.9130
H -3.7225 5.1080 -2.7189
H -2.1155 5.8512 -2.5317
H -2.4932 4.9054 -3.9917
C 5.7926 -0.5589 -0.6783
C 5.7144 -1.4425 0.4080
C 6.6452 -2.4535 0.5881
C 7.6730 -2.5588 -0.3583
C 7.7541 -1.6745 -1.4409
C 6.8085 -0.6557 -1.6168
C 4.6451 0.3833 -0.6148
C 4.5183 -1.0976 1.2173
H 6.5713 -3.1356 1.4279
H 8.4185 -3.3401 -0.2534
H 8.5626 -1.7833 -2.1565
H 6.8638 0.0327 -2.4531
O 4.2409 1.1920 -1.4249
O 3.9794 -1.6693 2.1349
O 2.7843 0.5604 0.9288
C 1.8221 -0.2068 0.2726
O 2.1117 -1.1010 -0.4811
C 0.4831 0.2982 0.6489
N 4.0654 0.1276 0.6476
C 2.4037 0.2346 -3.8603
H 3.3769 0.5888 -3.5084
H 2.1804 0.6650 -4.8399
H 2.4235 -0.8561 -3.9399
C 1.3985 0.6246 -2.8865
N 0.6328 0.9123 -2.0693
H 0.2378 0.1404 1.7029
C -3.9520 -1.5799 -1.8741
C -4.3154 -2.8355 -1.3687
C -5.4187 -3.5187 -1.8528
C -6.1595 -2.9052 -2.8735
C -5.7959 -1.6519 -3.3786
C -4.6786 -0.9659 -2.8815
C -2.7393 -1.1064 -1.1614
C -3.3482 -3.2136 -0.3074
H -5.6907 -4.4906 -1.4560
H -7.0287 -3.4111 -3.2807
H -6.3886 -1.2058 -4.1704
H -4.3857 0.0037 -3.2691
O -2.1539 -0.0412 -1.3377
O -3.2615 -4.1887 0.3940
O -1.4164 -2.0842 0.6289
C -0.3313 -2.8849 0.1649
O -0.2626 -3.2812 -0.9684
C 0.5621 -3.0570 1.2753
H 0.3535 -2.7442 2.2872
N 1.7256 -3.6116 1.0218
N 2.7258 -4.0783 0.7785
N -2.4572 -2.0983 -0.2485

TS-DM-Ap$_{syn}$

Ru -0.5233 0.8479 -0.8999
C 1.9790 2.3724 -1.9056
C 0.7665 2.3997 -1.2101
C 0.2980 3.6618 -0.7627
C 0.9925 4.8546 -1.0098
C 2.1911 4.7983 -1.7105
C 2.6772 3.5590 -2.1481
H 2.4004 1.4317 -2.2394

H 0.5935 5.7980 -0.6499
H 2.7506 5.7061 -1.9105
H 3.6213 3.5161 -2.6845
C -2.5890 2.6099 1.1619
C -2.7906 4.1467 1.0911
H -2.9442 4.6170 2.0615
C -0.9314 3.5788 -0.0120
N -1.4901 2.4207 0.1945
H -3.4821 2.0765 0.8283
O -1.5348 4.6427 0.5433
C -2.2014 2.1319 2.5499
C -3.0440 1.2834 3.2732
C -0.9950 2.5496 3.1319
C -2.6825 0.8356 4.5460
H -3.9760 0.9475 2.8295
C -0.6313 2.1060 4.4037
H -0.3333 3.2172 2.5889
C -1.4724 1.2411 5.1117
H -3.3418 0.1650 5.0878
H 0.3063 2.4342 4.8413
H -1.1883 0.8927 6.0996
H -3.5909 4.4311 0.4017
C -1.8462 2.1662 -3.6727
N -1.4914 1.6146 -2.7204
C -2.2783 2.8691 -4.8726
H -3.3439 3.1054 -4.8088
H -1.7133 3.7997 -4.9758
H -2.1033 2.2483 -5.7555
C 5.6772 -1.5233 -0.5186
C 5.7287 -1.6249 0.8788
C 6.6888 -2.3968 1.5136
C 7.6094 -3.0753 0.7034
C 7.5594 -2.9727 -0.6918
C 6.5861 -2.1889 -1.3261
C 4.5387 -0.6441 -0.8977
C 4.6253 -0.8137 1.4555
H 6.7189 -2.4705 2.5951
H 8.3742 -3.6919 1.1640
H 8.2870 -3.5105 -1.2909
H 6.5409 -2.1024 -2.4063
O 4.0691 -0.3957 -1.9897
O 4.2106 -0.7145 2.5861
O 2.8814 0.5229 0.4515
C 1.8026 -0.3665 0.5225
O 1.9496 -1.5589 0.4089
C 0.5566 0.4142 0.7240
N 4.0864 -0.1302 0.3315
C 1.9227 -2.3383 -3.2262
H 2.9476 -1.9666 -3.3082
H 1.5266 -2.5647 -4.2196
H 1.9201 -3.2495 -2.6209
C 1.1148 -1.3239 -2.5698
N 0.4997 -0.5178 -2.0115
H 0.7174 1.2485 1.4087
C -4.1600 -2.5834 -1.3472
C -4.7467 -3.1860 -0.2225
C -5.6853 -4.1972 -0.3522
C -6.0240 -4.6004 -1.6511
C -5.4384 -4.0004 -2.7722
C -4.4929 -2.9755 -2.6348
C -3.1971 -1.5570 -0.8846
C -4.1889 -2.5678 1.0099
H -6.1309 -4.6587 0.5222
H -6.7503 -5.3940 -1.7916
H -5.7209 -4.3379 -3.7639
H -4.0325 -2.5072 -3.4978
O -2.3895 -0.8841 -1.4986
O -4.3252 -2.8276 2.1773
O -2.4653 -0.8515 1.2803
C -1.2985 -1.5941 1.4381
O -1.1428 -2.7104 1.0360
C -0.3328 -0.7091 2.1321
H -0.7503 0.0837 2.7455
N 0.5989 -1.4241 2.8263
N 1.4711 -1.9532 3.2942
N -3.3934 -1.4944 0.4991

Product-DM-Ap$_{syn}$

Ru -0.7926 -1.1698 0.7281
C 1.4372 -3.2355 1.0998
C 0.1159 -2.9851 0.7311
C -0.7053 -4.0755 0.3653
C -0.2325 -5.3947 0.3747
C 1.0859 -5.6288 0.7496
C 1.9088 -4.5534 1.1066
H 2.1093 -2.4253 1.3591
H -0.8918 -6.2086 0.0904
H 1.4774 -6.6404 0.7602
H 2.9403 -4.7411 1.3908
C -3.7655 -2.2500 -0.3894
C -4.2363 -3.7292 -0.4744
H -4.6845 -3.9866 -1.4337
C -2.0451 -3.6752 0.0057
N -2.3479 -2.4121 0.0088
H -4.2949 -1.7167 0.4059
O -3.0226 -4.5274 -0.3154
C -3.8709 -1.4546 -1.6710
C -4.2220 -0.1039 -1.6180
C -3.4760 -2.0171 -2.8929
C -4.1646 0.6858 -2.7679
H -4.4972 0.3544 -0.6742
C -3.4296 -1.2321 -4.0441
H -3.1777 -3.0611 -2.9435
C -3.7679 0.1229 -3.9811
H -4.4142 1.7384 -2.6998
H -3.1198 -1.6728 -4.9863
H -3.7175 0.7359 -4.8751
H -4.9161 -4.0043 0.3345
C -1.8921 -1.9308 3.6636
N -1.5287 -1.6116 2.6132
C -2.3433 -2.3344 4.9866
H -3.4299 -2.2328 5.0568
H -2.0704 -3.3784 5.1649
H -1.8752 -1.7064 5.7494
C 5.7052 0.2956 -0.1032
C 5.7240 0.5759 -1.4766
C 6.7494 1.3099 -2.0511
C 7.7692 1.7655 -1.2043
C 7.7519 1.4841 0.1668
C 6.7130 0.7383 0.7396
C 4.4849 -0.4926 0.2120
C 4.5195 -0.0265 -2.1091
H 6.7536 1.5220 -3.1146
H 8.5872 2.3465 -1.6175
H 8.5578 1.8494 0.7949
H 6.6945 0.5109 1.8001
O 4.0312 -0.8455 1.2844
O 4.0860 0.0406 -3.2308
O 2.6547 -1.2786 -1.1652
C 1.6618 -0.3065 -1.0282
O 1.9003 0.8517 -0.8039
C 0.3570 -0.9630 -1.2092
N 3.9217 -0.7616 -1.0431
C 2.5098 1.4997 2.6154
H 3.4281 0.9286 2.7679
H 2.1759 1.9508 3.5528
H 2.6741 2.2909 1.8804
C 1.4885 0.6123 2.0923
N 0.6947 -0.0949 1.6394
H 0.3960 -1.9360 -1.6836
C -1.3822 4.8894 0.9122
C -2.6832 4.7623 0.4072
C -3.5028 5.8663 0.2298
C -2.9767 7.1197 0.5693
C -1.6766 7.2471 1.0734
C -0.8571 6.1256 1.2562
C -0.7555 3.5452 0.9870
C -2.9576 3.3303 0.1287
H -4.5077 5.7572 -0.1630
H -3.5849 8.0084 0.4370
H -1.2978 8.2327 1.3233
H 0.1520 6.2146 1.6433
O 0.3649 3.2136 1.2989
O -3.9151 2.7916 -0.3754
O -1.5477 1.3538 0.2728
C -1.0200 1.2073 -1.0298
O -0.8614 2.1212 -1.7762
C -0.8504 -0.2629 -1.2475
H -1.6605 -0.6889 -1.8266
N -1.8063 2.6622 0.6258

Int3-DM-Eq$_{trans}$

Ru -0.7504 1.2872 -0.5030
C -0.0452 4.3104 -0.3400
C -0.8753 3.2570 0.0496
C -1.9607 3.5685 0.9069
C -2.2097 4.8754 1.3469
C -1.3700 5.9051 0.9371
C -0.2893 5.6160 0.0984
H 0.8092 4.1189 -0.9821
H -3.0502 5.0659 2.0066
H -1.5481 6.9219 1.2704
H 0.3754 6.4153 -0.2167
C -3.2994 0.2781 1.6770
C -4.3324 1.2151 2.3502
H -4.5051 1.0041 3.4048
C -2.7627 2.4348 1.3240
N -2.5209 1.2468 0.8708

H -3.7868 -0.4470 1.0240
O -3.7331 2.5428 2.2481
C -2.3827 -0.4527 2.6416
C -2.0364 -1.7854 2.4084
C -1.8431 0.2084 3.7538
C -1.1491 -2.4475 3.2597
H -2.4807 -2.3087 1.5701
C -0.9801 -0.4598 4.6198
H -2.1018 1.2457 3.9480
C -0.6207 -1.7880 4.3681
H -0.8811 -3.4800 3.0589
H -0.5906 0.0521 5.4950
H 0.0518 -2.3075 5.0441
H -5.2842 1.2398 1.8125
C -2.6310 1.9163 -3.0082
N -1.9482 1.7053 -2.0994
C -3.4947 2.1620 -4.1542
H -2.9327 2.6697 -4.9429
H -3.8732 1.2120 -4.5438
H -4.3378 2.7928 -3.8590
C 1.0359 0.8592 2.0997
N 0.4075 0.9306 1.1330
C 1.8325 0.7767 3.3103
H 1.9042 -0.2665 3.6237
H 2.8343 1.1584 3.1072
H 1.3557 1.3574 4.1030
C 5.7717 -0.3353 -0.0133
C 6.1670 0.4276 -1.1211
C 7.3981 0.2379 -1.7288
C 8.2351 -0.7505 -1.1936
C 7.8413 -1.5120 -0.0867
C 6.5962 -1.3123 0.5236
C 4.4111 0.0867 0.4045
C 5.0741 1.3727 -1.4657
H 7.6931 0.8315 -2.5871
H 9.2048 -0.9314 -1.6453
H 8.5126 -2.2705 0.3025
H 6.2837 -1.8980 1.3812
O 3.6657 -0.3848 1.2430
O 4.9199 2.1160 -2.4010
O 2.8635 1.7251 -0.5222
C 2.0751 0.9360 -1.3530
O 2.4379 -0.1268 -1.7914
C 0.7449 1.5244 -1.6023
H 0.6932 2.0125 -2.5836
N 4.1435 1.2153 -0.3907
C 0.0407 -2.7789 -0.0852
C -0.3042 -4.1406 -0.0315
C 0.4698 -5.0536 0.6653
C 1.6200 -4.5694 1.3079
C 1.9774 -3.2195 1.2295
C 1.1875 -2.3001 0.5271
C -0.9979 -2.0718 -0.8649
C -1.5402 -4.3695 -0.8298
H 0.1931 -6.1015 0.7037
H 2.2481 -5.2577 1.8640
H 2.8835 -2.8631 1.7072
H 1.4799 -1.2639 0.4618
O -1.2327 -0.8855 -1.0579
O -2.2098 -5.3498 -1.0178
O -2.9223 -2.7783 -2.1091
C -4.0173 -2.4479 -1.2954
O -4.0162 -2.5780 -0.0958
C -5.0999 -1.9402 -2.0986
N -1.7958 -3.0773 -1.3915
N -4.9151 -1.6599 -3.3662
N -4.7508 -1.3945 -4.4572
H -6.0766 -1.7481 -1.6797

TS-DM-Eq$_{trans}$

Ru 0.5126 0.2970 0.6508
C -1.3412 0.9056 3.1171
C -0.2897 1.2868 2.2773
C 0.3817 2.4924 2.6257
C 0.0177 3.2729 3.7306
C -1.0391 2.8659 4.5352
C -1.7103 1.6821 4.2209
H -1.9113 0.0078 2.9215
H 0.5666 4.1853 3.9414
H -1.3383 3.4587 5.3930
H -2.5397 1.3539 4.8414
C 2.7796 2.7971 -0.0765
C 3.2283 3.9295 0.8805
H 3.3214 4.9051 0.4051
C 1.4725 2.8560 1.7512
N 1.8073 2.0807 0.7718
H 3.6080 2.1300 -0.3201
O 2.1597 4.0078 1.8697
C 2.1211 3.2728 -1.3580
C 2.3400 2.5946 -2.5590
C 1.2548 4.3741 -1.3455
C 1.7135 3.0118 -3.7346
H 2.9898 1.7262 -2.5678
C 0.6408 4.8028 -2.5210
H 1.0599 4.9018 -0.4164
C 0.8678 4.1209 -3.7201
H 1.8948 2.4776 -4.6622
H -0.0083 5.6735 -2.5033
H 0.3938 4.4581 -4.6368
H 4.1524 3.6821 1.4109
C 2.2993 -1.3311 2.7238
N 1.6680 -0.7166 1.9721
C 3.0700 -2.1723 3.6253
H 2.6428 -2.1372 4.6313
H 3.0375 -3.2012 3.2549
H 4.1088 -1.8342 3.6712
C -1.2889 1.9134 -1.4312
N -0.6205 1.3248 -0.6937
C -2.1530 2.6300 -2.3522
H -1.8643 3.6827 -2.3769
H -2.0475 2.2128 -3.3572
H -3.1865 2.5304 -2.0104
C -6.1229 -0.0162 -0.9514
C -6.1941 -1.3971 -0.7148
C -7.2206 -2.1691 -1.2362
C -8.1886 -1.5159 -2.0107
C -8.1187 -0.1376 -2.2459
C -7.0778 0.6363 -1.7152
C -4.9109 0.5278 -0.2816
C -5.0293 -1.7964 0.1146
H -7.2659 -3.2364 -1.0497
H -9.0064 -2.0872 -2.4375
H -8.8838 0.3378 -2.8507
H -7.0159 1.7045 -1.8922
O -4.3957 1.6217 -0.3417
O -4.5978 -2.8851 0.4219
O -3.1775 -0.5698 1.0384
C -2.2007 -0.9375 0.1115
O -2.4576 -1.0697 -1.0612
C -0.8936 -1.1453 0.8002
H -1.0319 -1.5428 1.8080
N -4.4408 -0.5607 0.4891
C 4.7163 -1.2716 -1.1979
C 5.0628 -2.6316 -1.2652
C 6.3731 -3.0346 -1.4694
C 7.3418 -2.0320 -1.6138
C 6.9972 -0.6765 -1.5492
C 5.6718 -0.2753 -1.3369
C 3.2558 -1.1660 -0.9743
C 3.8372 -3.4592 -1.0975
H 6.6297 -4.0870 -1.5226
H 8.3767 -2.3097 -1.7836
H 7.7704 0.0749 -1.6698
H 5.4001 0.7737 -1.2922
O 2.5177 -0.1962 -0.9606
O 3.6441 -4.6423 -1.1921
O 1.4960 -2.7392 -0.9498
C 0.8645 -3.2054 0.1834
O 1.3952 -3.6835 1.1454
C -0.5736 -2.9139 -0.0209
N 2.8388 -2.4866 -0.7696
N -1.3954 -3.7684 0.6568
N -2.1264 -4.3100 1.3162
H -0.9252 -2.7327 -1.0336

Product-DM-Eq$_{trans}$

Ru -0.2206 0.9177 -0.2888
C 1.6236 0.6905 -2.8441
C 1.0484 1.4580 -1.8386
C 1.3517 2.8493 -1.8307
C 2.2504 3.4254 -2.7351
C 2.8443 2.6224 -3.7022
C 2.5117 1.2678 -3.7614
H 1.4160 -0.3656 -2.9340
H 2.4588 4.4893 -2.6787
H 3.5444 3.0473 -4.4133
H 2.9505 0.6398 -4.5312
C -0.9985 3.8530 0.7476
C -0.1866 5.1649 0.5583
H 0.4087 5.4607 1.4227
C 0.5768 3.6054 -0.8746
N -0.3792 2.9865 -0.2616
H -2.0491 4.0136 0.4797
O 0.7533 4.8837 -0.5349
C -0.9377 3.0807 2.0549
C -1.6813 1.8834 2.0886
C -0.0803 3.3798 3.1089
C -1.5371 0.9929 3.1541

H	-2.4099	1.6668	1.3062
C	0.0471	2.4934	4.1848
H	0.5045	4.2937	3.1077
C	-0.6658	1.2953	4.2032
H	-2.1092	0.0723	3.1596
H	0.7086	2.7439	5.0087
H	-0.5565	0.6084	5.0361
H	-0.8115	6.0008	0.2450
C	-2.6614	0.7462	-2.3206
N	-1.7437	0.8257	-1.6238
C	-3.8495	0.5895	-3.1406
H	-3.7492	1.1389	-4.0799
H	-3.9932	-0.4738	-3.3530
H	-4.7097	0.9616	-2.5783
C	2.2230	1.1409	1.7333
N	1.3067	1.0911	1.0329
C	3.3924	1.1498	2.5916
H	3.3327	1.9699	3.3110
H	3.4445	0.1967	3.1254
H	4.2795	1.2532	1.9612
C	5.9119	-1.6351	0.1666
C	5.7027	-3.0090	-0.0176
C	6.6512	-3.9436	0.3672
C	7.8308	-3.4625	0.9508
C	8.0417	-2.0903	1.1324
C	7.0788	-1.1518	0.7387
C	4.7214	-0.8935	-0.3264
C	4.3697	-3.2142	-0.6448
H	6.4779	-5.0043	0.2221
H	8.5951	-4.1643	1.2682
H	8.9672	-1.7500	1.5853
H	7.2366	-0.0867	0.8706
O	4.4301	0.2815	-0.2352
O	3.7390	-4.2147	-0.8738
O	2.6316	-1.6469	-1.3000
C	1.7896	-1.5378	-0.2028
O	2.1738	-1.6764	0.9317
C	0.4111	-1.2503	-0.6560
H	0.1512	-1.5132	-1.6737
N	3.9399	-1.8878	-0.9470
C	-6.1356	-1.2388	-0.0271
C	-5.9989	-2.5874	0.3330
C	-7.0325	-3.4927	0.1520
C	-8.2221	-3.0095	-0.4098
C	-8.3604	-1.6629	-0.7669
C	-7.3124	-0.7524	-0.5765
C	-4.8663	-0.5289	0.2743
C	-4.6376	-2.8051	0.8921
H	-6.9160	-4.5337	0.4329
H	-9.0517	-3.6899	-0.5713
H	-9.2958	-1.3200	-1.1968
H	-7.4145	0.2941	-0.8432
O	-4.5108	0.6048	0.0125
O	-4.0612	-3.8058	1.2320
O	-2.7513	-1.2783	1.1601
C	-1.9856	-1.5226	0.0130
O	-2.4624	-1.9216	-1.0158
C	-0.5845	-1.1735	0.3193
N	-4.0960	-1.4845	0.9579
H	-0.2984	-1.2172	1.3656

TS-MI-Ap$_{anti}$

Ru	1.6357	-0.7655	0.6309
C	0.6935	-3.6120	-0.6621
C	0.6615	-2.2138	-0.6854
C	0.2236	-1.5764	-1.8767
C	-0.1500	-2.3055	-3.0032
C	-0.0948	-3.7004	-2.9561
C	0.3276	-4.3489	-1.7921
H	0.9997	-4.1286	0.2425
H	-0.4784	-1.7888	-3.8990
H	-0.3901	-4.2816	-3.8233
H	0.3605	-5.4336	-1.7624
C	0.3705	1.9135	-0.8525
C	-0.2745	2.0160	-2.2652
H	0.3656	2.5309	-2.9850
C	0.1965	-0.1245	-1.8025
N	0.7206	0.4828	-0.7835
H	-0.3894	2.1048	-0.0906
O	-0.4171	0.6300	-2.7126
C	1.5354	2.8519	-0.6557
C	1.3637	4.0186	0.0950
C	2.7752	2.6000	-1.2524
C	2.4137	4.9275	0.2423
H	0.4012	4.2186	0.5581
C	3.8306	3.4956	-1.0914
H	2.9097	1.6911	-1.8287
C	3.6520	4.6654	-0.3470
H	2.2642	5.8381	0.8144
H	4.7913	3.2911	-1.5547
H	4.4694	5.3713	-0.2364
H	-1.2688	2.4572	-2.2380
C	2.9266	-2.9233	2.6242
N	2.4829	-2.1385	1.8968
C	3.4870	-3.9056	3.5425
H	2.8372	-4.0150	4.4155
H	3.5809	-4.8761	3.0470
H	4.4766	-3.5828	3.8777
C	4.3722	-0.7730	-1.2753
N	3.4808	-0.6652	-0.5448
C	5.4894	-0.9049	-2.2009
H	6.3935	-1.1968	-1.6596
H	5.2641	-1.6675	-2.9516
H	5.6720	0.0472	-2.7070
C	-4.9888	1.1275	0.1641
C	-5.4756	-0.1722	0.3614
C	-6.8058	-0.4073	0.6712
C	-7.6483	0.7069	0.7834
C	-7.1624	2.0050	0.5864
C	-5.8168	2.2343	0.2703
C	-3.5361	1.0601	-0.1422
C	-4.3574	-1.1370	0.1905
H	-7.1717	-1.4167	0.8242
H	-8.6952	0.5629	1.0296
H	-7.8402	2.8468	0.6827
H	-5.4304	3.2362	0.1182
O	-2.7208	1.9480	-0.2879
O	-4.2934	-2.3287	0.3624
O	-1.9954	-0.8179	-0.2849
C	-1.4368	-0.9136	0.9873
O	-2.0076	-0.5335	1.9775
C	-0.0827	-1.5062	1.0586
H	-0.1271	-2.4131	1.6671
N	-3.2792	-0.3183	-0.2533
C	2.8093	1.7238	2.3161
N	2.4111	0.7956	1.7519
C	3.2944	2.9073	3.0087
H	2.4496	3.4728	3.4110
H	3.9574	2.6213	3.8296
H	3.8336	3.5415	2.3008

Int3-MI-Ap$_{anti}$

Ru	-1.3247	0.3431	1.0613
C	-0.2952	4.0049	-0.6765
C	-0.2114	2.6089	-0.6500
C	-0.5026	1.9175	-1.8475
C	-0.8065	2.6071	-3.0270
C	-0.8830	3.9985	-3.0261
C	-0.6347	4.6951	-1.8418
H	-0.0719	4.5583	0.2308
H	-0.9944	2.0443	-3.9349
H	-1.1239	4.5315	-3.9399
H	-0.6846	5.7797	-1.8282
C	-0.8364	-1.7179	-1.3243
C	-0.1405	-1.5916	-2.7049
H	-0.6607	-2.1130	-3.5083
C	-0.5327	0.4532	-1.8598
N	-0.9081	-0.3107	-0.8845
H	-0.1960	-2.2672	-0.6327
O	-0.1777	-0.1661	-2.9943
C	-2.2005	-2.3681	-1.4044
C	-2.3375	-3.7280	-1.1108
C	-3.3205	-1.6464	-1.8293
C	-3.5723	-4.3639	-1.2508
H	-1.4709	-4.2931	-0.7771
C	-4.5577	-2.2761	-1.9613
H	-3.2235	-0.5856	-2.0370
C	-4.6864	-3.6383	-1.6777
H	-3.6632	-5.4238	-1.0325
H	-5.4208	-1.7076	-2.2945
H	-5.6467	-4.1315	-1.7936
H	0.9078	-1.8899	-2.6587
C	-1.5756	1.5535	4.0179
N	-1.5470	1.0958	2.9547
C	-1.5976	2.1308	5.3551
H	-0.5807	2.3862	5.6665
H	-2.2078	3.0383	5.3633
H	-2.0178	1.4164	6.0685
C	-3.4218	2.3745	-0.2105
N	-2.7282	1.6177	0.3232
C	-4.2086	3.3574	-0.9398
H	-3.5552	3.8596	-1.6598
H	-5.0299	2.8717	-1.4736
H	-4.6229	4.0999	-0.2525
C	4.7188	-1.3086	-0.3235
C	5.3644	-0.0663	-0.2657
C	6.7362	0.0283	-0.0978
C	7.4566	-1.1690	0.0191
C	6.8125	-2.4101	-0.0397

C 5.4247 -2.4969 -0.2163
C 3.2589 -1.0837 -0.5024
C 4.3473 1.0119 -0.4172
H 7.2259 0.9951 -0.0562
H 8.5324 -1.1344 0.1562
H 7.3988 -3.3188 0.0506
H 4.9181 -3.4547 -0.2718
O 2.3443 -1.8913 -0.5245
O 4.4508 2.2115 -0.3772
O 1.9223 0.9219 -0.6468
C 1.4915 1.2896 0.6613
O 2.1918 1.0547 1.6159
C 0.1494 1.8990 0.6222
H 0.1029 2.5866 1.4678
N 3.1381 0.2972 -0.6464
C 0.9888 -1.5595 2.1390
N 0.0941 -0.9330 1.7612
C 2.1701 -2.2801 2.5826
H 2.4765 -2.9804 1.8021
H 2.9719 -1.5528 2.7419
H 1.9742 -2.8182 3.5134
C -3.6582 -1.9142 1.6124
N -2.8314 -1.1244 1.4373
C -4.6972 -2.9150 1.8049
H -5.1108 -2.8467 2.8145
H -5.4914 -2.7598 1.0709
H -4.2780 -3.9116 1.6475

TS-MI-Ap$_{syn}$

Ru -1.2234 -0.4382 0.7404
C 0.0603 -3.2679 -0.2776
C -0.8296 -2.2118 -0.4916
C -1.8532 -2.3870 -1.4638
C -1.9876 -3.5721 -2.1872
C -1.0919 -4.6153 -1.9454
C -0.0823 -4.4632 -0.9909
H 0.8781 -3.1449 0.4199
H -2.7866 -3.6767 -2.9139
H -1.1783 -5.5420 -2.5027
H 0.6116 -5.2785 -0.8099
C -3.8438 0.6199 -0.9677
C -4.3982 0.0749 -2.3139
H -4.0857 0.6915 -3.1606
C -2.7824 -1.2771 -1.5658
N -2.7055 -0.2835 -0.7340
H -4.5671 0.4579 -0.1582
O -3.7730 -1.2364 -2.4644
C -3.4931 2.0882 -1.0148
C -4.4431 3.0318 -0.6108
C -2.2549 2.5259 -1.4962
C -4.1669 4.3978 -0.6992
H -5.4055 2.6982 -0.2307
C -1.9694 3.8888 -1.5659
H -1.5065 1.7995 -1.7900
C -2.9262 4.8287 -1.1742
H -4.9180 5.1215 -0.3981
H -1.0014 4.2167 -1.9310
H -2.7071 5.8897 -1.2417
H -5.4788 -0.0623 -2.3289
C -2.0284 2.3915 2.0598
N -1.7709 1.3295 1.6807
C -2.3426 3.7479 2.4798
H -3.0419 3.7341 3.3201
H -1.4277 4.2623 2.7864
H -2.7943 4.2824 1.6394
C -3.3273 -2.2281 2.5909
N -2.6485 -1.4826 2.0225
C -4.1721 -3.1745 3.3071
H -4.2059 -4.1246 2.7666
H -3.7694 -3.3507 4.3083
H -5.1881 -2.7808 3.3978
C 5.5608 0.4240 -1.0467
C 5.3424 0.9075 0.2506
C 6.3900 1.0892 1.1406
C 7.6786 0.7663 0.6953
C 7.8959 0.2816 -0.6001
C 6.8326 0.1046 -1.4955
C 4.2541 0.3316 -1.7520
C 3.8865 1.1438 0.4382
H 6.2128 1.4682 2.1413
H 8.5235 0.8931 1.3643
H 8.9057 0.0386 -0.9142
H 6.9900 -0.2709 -2.5007
O 3.9567 -0.1368 -2.8221
O 3.2555 1.4300 1.4357
O 1.9860 0.8113 -1.0286
C 1.5079 -0.4145 -0.5369
O 2.2366 -1.2334 -0.0392
C 0.0389 -0.3721 -0.6940
N 3.3433 0.9502 -0.8467
C 2.4595 -0.8011 3.5512
H 3.0844 0.0484 3.2595
H 2.9907 -1.7307 3.3284
H 2.2412 -0.7572 4.6213
C 1.2325 -0.7450 2.7742
N 0.2871 -0.6868 2.1110
H -0.2608 -0.1524 -1.7219

Int3-MI-Ap$_{syn}$

Ru 1.1760 0.0155 0.9744
C -0.0294 3.5295 -0.9061
C 0.7150 2.3437 -0.9285
C 2.0966 2.4456 -1.2373
C 2.6780 3.6782 -1.5674
C 1.9129 4.8405 -1.5495
C 0.5610 4.7580 -1.2063
H -1.0834 3.4839 -0.6628
H 3.7322 3.7129 -1.8187
H 2.3623 5.7947 -1.8034
H -0.0483 5.6568 -1.1893
C 3.8645 -0.7298 -0.5904
C 4.8528 0.0887 -1.4588
H 5.1763 -0.4329 -2.3593
C 2.9634 1.2744 -1.1301
N 2.7818 0.2509 -0.3546
H 4.3099 -0.9928 0.3716
O 4.0916 1.2568 -1.8668
C 3.3278 -1.9816 -1.2491
C 3.4675 -3.2229 -0.6278
C 2.6593 -1.9004 -2.4767
C 2.9466 -4.3748 -1.2201
H 3.9793 -3.2873 0.3286
C 2.1368 -3.0464 -3.0707
H 2.5374 -0.9374 -2.9647
C 2.2792 -4.2876 -2.4423
H 3.0662 -5.3370 -0.7317
H 1.6237 -2.9746 -4.0247
H 1.8795 -5.1824 -2.9095
H 5.7234 0.4359 -0.8960
C -0.0427 -2.6154 -0.3132
N 0.5439 -1.7312 0.1433
C -0.8301 -3.6940 -0.8829
H -0.3496 -4.6569 -0.6952
H -1.8235 -3.6685 -0.4272
H -0.9177 -3.5482 -1.9626
C 2.9798 -1.6042 3.1975
N 2.3686 -1.0107 2.4126
C 3.7437 -2.3524 4.1878
H 4.7838 -2.0147 4.1972
H 3.3155 -2.2012 5.1826
H 3.7195 -3.4201 3.9526
C -5.3849 0.1839 -0.9049
C -5.1400 -0.8194 0.0418
C -6.1629 -1.3575 0.8076
C -7.4548 -0.8535 0.6051
C -7.6988 0.1495 -0.3403
C -6.6601 0.6827 -1.1164
C -4.0994 0.5474 -1.5625
C -3.6870 -1.1385 0.0385
H -5.9656 -2.1401 1.5327
H -8.2809 -1.2472 1.1882
H -8.7102 0.5191 -0.4742
H -6.8388 1.4583 -1.8531
O -3.8386 1.3949 -2.3788
O -3.0543 -1.8944 0.7566
O -1.8167 -0.2104 -1.1860
C -1.3070 0.9085 -0.4536
O -2.0504 1.5691 0.2332
C 0.1351 0.9905 -0.6815
N -3.1660 -0.3749 -1.0084
C -2.8326 -0.0827 3.3011
H -3.3208 -1.0411 3.1100
H -3.4137 0.7118 2.8235
H -2.7767 0.1028 4.3767
C -1.5057 -0.1028 2.7078
N -0.4739 -0.0969 2.1865
H 0.4001 0.3193 -1.4994
C 2.0678 4.3350 2.3665
H 1.2567 4.7318 2.9831
H 3.0154 4.4704 2.8945
H 2.0975 4.8825 1.4196
C 1.8360 2.9278 2.0792
N 1.6347 1.8303 1.7740

TS-MI-Eq$_{trans}$

Ru 1.1647 0.3749 0.7496
C -0.8611 3.2481 -0.4529

C	0.1404	2.2672	-0.4057
C	1.1765	2.3742	-1.4011
C	1.1044	3.3830	-2.3831
C	0.0716	4.3075	-2.4139
C	-0.9059	4.2446	-1.4291
H	-1.6703	3.2339	0.2671
H	1.8972	3.4341	-3.1191
H	0.0416	5.0689	-3.1857
H	-1.7219	4.9600	-1.4100
C	4.1462	0.2291	-0.8810
C	4.3849	0.8662	-2.2736
H	4.2775	0.1367	-3.0812
C	2.4167	1.5790	-1.4447
N	2.7696	0.6692	-0.5940
H	4.8049	0.6795	-0.1282
O	3.3189	1.8468	-2.4107
C	4.3180	-1.2703	-0.8430
C	5.4139	-1.8351	-0.1858
C	3.3943	-2.1075	-1.4795
C	5.5945	-3.2204	-0.1715
H	6.1336	-1.1901	0.3115
C	3.5636	-3.4900	-1.4532
H	2.5336	-1.6721	-1.9754
C	4.6669	-4.0508	-0.8032
H	6.4591	-3.6476	0.3271
H	2.8409	-4.1332	-1.9460
H	4.8053	-5.1274	-0.7956
H	5.3367	1.3882	-2.3675
C	2.2737	2.4915	2.8685
N	1.8985	1.7069	2.1045
C	2.7368	3.4759	3.8363
H	2.5648	4.4851	3.4514
H	2.1937	3.3586	4.7784
H	3.8063	3.3462	4.0240
C	-0.3381	-1.6822	-1.1678
N	0.3144	-0.9773	-0.5254
C	-1.2264	-2.5295	-1.9431
H	-0.6710	-3.3301	-2.4381
H	-1.9752	-2.9481	-1.2633
H	-1.7334	-1.9154	-2.6926
C	-5.8663	-0.2661	0.1352
C	-5.3119	-1.5509	0.0552
C	-6.0126	-2.6070	-0.5066
C	-7.2982	-2.3413	-0.9961
C	-7.8509	-1.0573	-0.9176
C	-7.1377	0.0047	-0.3457
C	-4.8743	0.6419	0.7707
C	-3.9418	-1.5207	0.6341
H	-5.5772	-3.5990	-0.5610
H	-7.8767	-3.1425	-1.4444
H	-8.8483	-0.8832	-1.3078
H	-7.5563	1.0031	-0.2813
O	-4.8645	1.8409	0.9100
O	-3.0694	-2.3656	0.6354
O	-2.6284	0.2804	1.5850
C	-1.8449	0.6384	0.4706
O	-2.2172	0.4325	-0.6596
C	-0.5941	1.2033	0.9833
H	-0.7454	1.9425	1.7665
N	-3.8437	-0.2374	1.2066
C	3.1991	-3.2702	2.6908
H	2.4407	-4.0231	2.9234
H	3.7360	-3.0141	3.6083
H	3.9045	-3.6774	1.9610
C	2.5674	-2.0850	2.1331
N	2.0625	-1.1517	1.6713

Cyclopropanation of styrene

Ap_{anti} position

Int3-IS-Ap_{anti}

Ru	-1.0293	-0.7462	-0.0617
C	-0.8835	-3.2350	1.8148
C	-0.5090	-1.9181	1.5548
C	0.2795	-1.2519	2.5187
C	0.6862	-1.8694	3.7064
C	0.3018	-3.1861	3.9434
C	-0.4784	-3.8617	2.9993
H	-1.4836	-3.7861	1.0985
H	1.2939	-1.3224	4.4199
H	0.6094	-3.6877	4.8548
H	-0.7707	-4.8913	3.1860
C	0.6851	1.9826	0.8908
C	1.4401	2.1918	2.2379
H	0.9980	2.9750	2.8556
C	0.6168	0.1083	2.1559
N	0.2255	0.5843	1.0093
H	1.3789	2.0506	0.0488
O	1.2875	0.9291	2.9553
C	-0.4680	2.9355	0.6743
C	-0.4746	3.8075	-0.4170
C	-1.5572	2.9360	1.5556
C	-1.5617	4.6555	-0.6413
H	0.3693	3.8138	-1.1014
C	-2.6425	3.7817	1.3354
H	-1.5583	2.2571	2.4029
C	-2.6506	4.6395	0.2305
H	-1.5589	5.3233	-1.4966
H	-3.4797	3.7826	2.0278
H	-3.4962	5.2974	0.0575
C	-1.2134	0.7882	-2.2009
C	-2.3453	1.1092	-1.5161
H	-1.2279	0.1268	-3.0612
H	-0.2835	1.3094	-2.0145
H	-2.2628	1.8683	-0.7473
H	2.5065	2.3555	2.1004
C	-2.9455	-2.9309	-1.3931
N	-2.2207	-2.1589	-0.9249
C	-3.8888	-3.8753	-1.9717
H	-3.4528	-4.3547	-2.8526
H	-4.1471	-4.6470	-1.2411
H	-4.7951	-3.3384	-2.2664
C	-3.7453	0.0480	1.8000
N	-2.7754	-0.1687	1.2111
C	-4.9785	0.3336	2.5160
H	-5.1281	-0.3960	3.3162
H	-4.9342	1.3357	2.9512
H	-5.8180	0.2830	1.8170
C	5.6625	0.4086	-0.2458
C	5.8783	-0.3497	-1.4056
C	7.0403	-0.2188	-2.1498
C	7.9940	0.7036	-1.6989
C	7.7792	1.4601	-0.5407
C	6.6024	1.3214	0.2071

C 4.3322 0.0610 0.3190
C 4.6966 -1.2219 -1.6376
H 7.1965 -0.8081 -3.0467
H 8.9149 0.8362 -2.2571
H 8.5374 2.1664 -0.2187
H 6.4272 1.9031 1.1056
O 3.7011 0.5587 1.2261
O 4.3919 -1.9315 -2.5638
O 2.6094 -1.4898 -0.4211
C 1.7362 -0.6692 -1.1101
O 2.0535 0.3818 -1.6118
C 0.3880 -1.2776 -1.1448
H 0.2932 -2.0487 -1.9192
N 3.9138 -1.0347 -0.4620
C -3.7167 0.6706 -1.8043
C -4.0405 -0.2552 -2.8103
C -4.7612 1.2331 -1.0511
C -5.3645 -0.6219 -3.0365
H -3.2550 -0.6819 -3.4249
C -6.0863 0.8638 -1.2740
H -4.5187 1.9812 -0.3018
C -6.3923 -0.0730 -2.2627
H -5.6017 -1.3208 -3.8334
H -6.8826 1.3207 -0.6937
H -7.4237 -0.3559 -2.4472

TS4-IS-Ap$_{anti}$

Ru 0.4748 0.1359 -0.0716
C -1.7509 0.4024 2.1179
C -1.0703 -0.3896 1.1922
C -1.4604 -1.7457 1.0815
C -2.5173 -2.2867 1.8225
C -3.1919 -1.4673 2.7224
C -2.7994 -0.1335 2.8733
H -1.4814 1.4438 2.2493
H -2.7914 -3.3293 1.6976
H -4.0164 -1.8623 3.3058
H -3.3248 0.5005 3.5818
C 1.0791 -2.9463 -1.2387
C 0.3019 -4.2534 -0.8952
H 0.9145 -4.9771 -0.3548
C -0.6306 -2.5158 0.1788
N 0.3426 -1.9370 -0.4577
H 0.9833 -2.7198 -2.3063
O -0.7746 -3.8304 -0.0053
C 2.5458 -2.9620 -0.8690
C 3.5302 -2.8977 -1.8581
C 2.9271 -2.9882 0.4785
C 4.8813 -2.8426 -1.5096
H 3.2407 -2.8788 -2.9056
C 4.2747 -2.9336 0.8280
H 2.1654 -3.0205 1.2512
C 5.2553 -2.8537 -0.1657
H 5.6378 -2.7869 -2.2856
H 4.5641 -2.9593 1.8746
H 6.3048 -2.8087 0.1068
C 1.8523 0.4086 -2.0651
C 2.6395 0.4316 -0.8872
H 1.7711 1.3107 -2.6608
H 1.8276 -0.5271 -2.6100
H 3.0680 -0.5155 -0.5814
H -0.1526 -4.7263 -1.7663
C -0.0789 3.2318 0.4817
N 0.2364 2.1246 0.3672
C -0.5181 4.6131 0.5651
H -0.2924 5.0307 1.5497
H -0.0117 5.2037 -0.2035
H -1.5978 4.6314 0.3907
C 2.3221 -0.0786 2.7294
N 1.6380 -0.1364 1.8001
C 3.2054 0.0181 3.8813
H 3.8322 0.9081 3.7750
H 2.6216 0.0941 4.8022
H 3.8466 -0.8659 3.9373
C -4.3576 -0.3360 -0.8187
C -4.4852 0.7490 0.0578
C -5.4333 0.7541 1.0683
C -6.2635 -0.3683 1.1804
C -6.1262 -1.4591 0.3138
C -5.1622 -1.4591 -0.7028
C -3.2922 -0.0359 -1.8105
C -3.4739 1.7793 -0.2957
H -5.5258 1.6008 1.7393
H -7.0269 -0.3942 1.9511
H -6.7843 -2.3144 0.4264
H -5.0541 -2.2944 -1.3859
O -2.8815 -0.6768 -2.7526
O -3.2398 2.8585 0.2015
O -2.0421 2.0464 -2.2107
C -0.6760 1.7871 -2.2972
O 0.0026 2.6621 -2.7709
C -0.2479 0.4517 -1.8047
H -0.5431 -0.3593 -2.4737
N -2.7762 1.2107 -1.3859
C 3.3083 1.6165 -0.3455
C 4.3160 1.4225 0.6162
C 2.9990 2.9284 -0.7475
C 4.9796 2.5080 1.1828
H 4.5851 0.4075 0.8958
C 3.6627 4.0104 -0.1791
H 2.2332 3.0992 -1.4966
C 4.6482 3.8072 0.7926
H 5.7671 2.3433 1.9123
H 3.4214 5.0187 -0.5013
H 5.1670 4.6558 1.2269

Int4-IS-Ap$_{anti}$

Ru -0.3820 -0.5911 -0.0360
C 1.4353 -1.0392 2.4166
C 0.9483 -0.1471 1.4615
C 1.4124 1.1851 1.4868
C 2.3467 1.6271 2.4278
C 2.8214 0.7217 3.3728
C 2.3736 -0.6037 3.3585
H 1.0889 -2.0654 2.4367
H 2.6945 2.6540 2.4062
H 3.5437 1.0427 4.1157
H 2.7538 -1.3059 4.0947
C -0.5586 2.5290 -1.2456
C 0.5166 3.6327 -1.0447
H 0.1034 4.6330 -0.9245
C 0.8317 2.0005 0.4459
N -0.0783 1.4836 -0.3227
H -0.5240 2.1617 -2.2747
O 1.1824 3.2656 0.2056
C -1.9735 2.9587 -0.9095
C -2.8843 3.2270 -1.9363
C -2.3860 3.0829 0.4229
C -4.1932 3.6093 -1.6397
H -2.5714 3.1268 -2.9726
C -3.6928 3.4670 0.7200
H -1.6927 2.8564 1.2263
C -4.6005 3.7280 -0.3100
H -4.8929 3.8101 -2.4445
H -4.0045 3.5639 1.7555
H -5.6183 4.0246 -0.0774
C -1.5072 -0.9318 -2.1439
C -2.3073 -0.3682 -0.9581
H 1.2740 3.6109 -1.8313
C 0.3335 -3.6815 0.0299
N -0.1590 -2.6417 0.1662
C 1.0266 -4.9295 -0.2290
H 0.7814 -5.2569 -1.2434
H 2.1002 -4.7240 -0.1723
H 0.7473 -5.6995 0.4940
C -2.3943 -0.4956 2.6817
N -1.6111 -0.4667 1.8324
C -3.4086 -0.5561 3.7233
H -3.6122 0.4464 4.1089
H -4.3260 -0.9717 3.2977
H -3.0671 -1.1906 4.5453
C 3.9222 0.8323 -0.7559
C 4.1984 -0.2799 0.0510
C 5.0615 -0.1918 1.1296
C 5.6569 1.0529 1.3803
C 5.3705 2.1659 0.5834
C 4.4869 2.0712 -0.5013
C 2.9886 0.4290 -1.8410
C 3.4313 -1.4453 -0.4611
H 5.2674 -1.0568 1.7494
H 6.3505 1.1561 2.2084
H 5.8467 3.1157 0.8039
H 4.2633 2.9263 -1.1293
O 2.4934 1.0921 -2.7290
O 3.3588 -2.5843 -0.0557
O 2.2003 -1.8198 -2.4884
C 0.7949 -1.9476 -2.4876
O 0.3662 -2.9945 -2.9024
C 0.0565 -0.7708 -2.0092
N 2.7289 -0.9318 -1.5764
H -2.5107 0.6924 -1.0407
H 0.4427 0.1382 -2.4628
H -1.7419 -1.9757 -2.3400
H -1.8021 -0.3221 -3.0049
C -3.4315 -1.0990 -0.3840
C -4.4978 -0.3530 0.1582
C -3.4853 -2.5042 -0.3155
C -5.5832 -0.9926 0.7501

H -4.4645 0.7314 0.1045
C -4.5664 -3.1401 0.2840
H -2.6705 -3.0939 -0.7191
C -5.6186 -2.3888 0.8195
H -6.4066 -0.4066 1.1471
H -4.5983 -4.2240 0.3288
H -6.4664 -2.8903 1.2754

TS5-IS-Ap$_{anti}$

Ru -0.2634 -0.3463 -0.0094
C 1.5910 -1.0657 2.4024
C 1.1776 -0.1173 1.4709
C 1.7811 1.1579 1.4887
C 2.7987 1.4787 2.3920
C 3.2046 0.5151 3.3135
C 2.6034 -0.7476 3.3161
H 1.1361 -2.0496 2.4200
H 3.2591 2.4607 2.3655
H 3.9878 0.7453 4.0280
H 2.9232 -1.4922 4.0394
C -0.1125 2.7859 -1.1644
C 0.9475 3.8704 -0.8080
H 0.5083 4.8136 -0.4834
C 1.2518 2.0644 0.4876
N 0.2982 1.6659 -0.2979
H -0.0108 2.4872 -2.2108
O 1.6909 3.3129 0.3233
C -1.5468 3.1774 -0.8850
C -2.4625 3.3075 -1.9315
C -1.9802 3.3634 0.4341
C -3.8022 3.5986 -1.6666
H -2.1305 3.1670 -2.9566
C -3.3161 3.6565 0.7002
H -1.2758 3.2538 1.2535
C -4.2325 3.7663 -0.3503
H -4.5076 3.6890 -2.4861
H -3.6449 3.8051 1.7246
H -5.2741 3.9890 -0.1430
C -2.0479 -0.3238 -2.2227
C -2.4784 -0.0896 -0.7915
H 1.6619 4.0425 -1.6146
C -0.3114 -3.5154 0.1274
N -0.4508 -2.3723 0.2339
C -0.0895 -4.9291 -0.1108
H -0.3393 -5.1369 -1.1556
H 0.9694 -5.1470 0.0529
H -0.7071 -5.5427 0.5494
C -1.9626 0.2006 2.6333
N -1.2950 0.0453 1.7026
C -2.8017 0.3816 3.8074
H -3.2857 1.3616 3.7701
H -3.5731 -0.3931 3.8173
H -2.2005 0.3141 4.7181
C 3.7327 0.2485 -1.0255
C 3.8653 -0.8686 -0.1908
C 4.8721 -0.9422 0.7604
C 5.7519 0.1424 0.8518
C 5.6140 1.2634 0.0219
C 4.5939 1.3335 -0.9327
C 2.5944 0.0398 -1.9398
C 2.8048 -1.8473 -0.5159
H 4.9668 -1.8091 1.4035
H 6.5579 0.1163 1.5777
H 6.3160 2.0851 0.1177
H 4.4821 2.1910 -1.5868
O 2.2164 0.7000 -2.8782
O 2.6334 -2.9767 -0.1316
O 1.3539 -2.0089 -2.3957
C -0.0608 -1.8623 -2.4974
O -0.6664 -2.8472 -2.8426
C -0.5397 -0.5359 -2.1434
N 1.9375 -1.1580 -1.4537
H -2.6010 0.9502 -0.5161
H 0.0680 0.2484 -2.5833
H -2.5399 -1.1905 -2.6688
H -2.2879 0.5530 -2.8315
C -3.4190 -0.9879 -0.1402
C -4.2985 -0.4676 0.8330
C -3.5190 -2.3562 -0.4687
C -5.2285 -1.2851 1.4693
H -4.2688 0.5975 1.0456
C -4.4404 -3.1717 0.1776
H -2.8618 -2.7742 -1.2233
C -5.2933 -2.6446 1.1538
H -5.9168 -0.8623 2.1956
H -4.5065 -4.2229 -0.0857
H -6.0171 -3.2850 1.6476

Product-IS-Ap$_{anti}$

Ru -0.2122 0.2396 0.1877
C -2.5458 0.0267 2.1797
C -1.5917 -0.6166 1.3857
C -1.5670 -2.0348 1.4092
C -2.4794 -2.7860 2.1589
C -3.4317 -2.1196 2.9247
C -3.4547 -0.7194 2.9352
H -2.5988 1.1095 2.1981
H -2.4364 -3.8703 2.1324
H -4.1477 -2.6816 3.5149
H -4.1957 -0.2026 3.5390
C 1.1238 -2.5859 -0.9784
C 0.6094 -4.0354 -0.7216
H 1.3878 -4.7177 -0.3803
C -0.5549 -2.5970 0.5393
N 0.2073 -1.7939 -0.1407
H 0.9768 -2.3144 -2.0257
O -0.3714 -3.9034 0.3556
C 2.5723 -2.3666 -0.5974
C 3.5265 -2.0970 -1.5824
C 2.9792 -2.4762 0.7384
C 4.8727 -1.9423 -1.2394
H 3.2125 -2.0034 -2.6181
C 4.3216 -2.3264 1.0813
H 2.2413 -2.6874 1.5069
C 5.2732 -2.0584 0.0921
H 5.6059 -1.7361 -2.0127
H 4.6332 -2.4383 2.1161
H 6.3197 -1.9484 0.3580
C 2.2473 2.1391 -2.8730
C 2.4929 1.3549 -1.6360
H 2.2614 3.2214 -2.8042
H 2.5711 1.7143 -3.8162
H 2.9393 0.3792 -1.7882
H 0.0877 -4.4514 -1.5846
C -1.0016 3.2682 0.8447
N -0.7411 2.1599 0.6281
C -1.3110 4.6687 1.0846
H -1.7064 5.1193 0.1695
H -2.0544 4.7650 1.8810
H -0.4044 5.2032 1.3828
C 1.7380 0.1399 2.6568
N 1.0259 0.2214 1.7493
C 2.6518 0.0121 3.7796
H 3.5627 -0.4902 3.4428
H 2.9146 1.0022 4.1587
H 2.1882 -0.5739 4.5781
C -3.6439 -0.3908 -0.9172
C -3.5892 1.0056 -1.0228
C -4.6176 1.8057 -0.5370
C -5.7059 1.1636 0.0595
C -5.7582 -0.2356 0.1612
C -4.7248 -1.0361 -0.3308
C -2.4272 -0.9755 -1.4971
C -2.3366 1.4060 -1.6785
H -4.5754 2.8852 -0.6318
H -6.5302 1.7541 0.4457
H -6.6189 -0.7012 0.6296
H -4.7462 -2.1159 -0.2448
O -2.1515 -2.0987 -1.8086
O -1.9713 2.4656 -2.1186
O -0.7975 -0.0317 -2.9051
C 0.5676 0.2634 -2.9408
O 1.2390 -0.4438 -3.6492
C 1.0563 1.4497 -2.2137
H 0.3283 2.0970 -1.7463
N -1.4939 0.1833 -1.6814
C 2.8692 2.0064 -0.3524
C 3.8502 1.3987 0.4403
C 2.3201 3.2248 0.0681
C 4.2762 1.9988 1.6248
H 4.2849 0.4576 0.1219
C 2.7298 3.8133 1.2625
H 1.5719 3.7215 -0.5425
C 3.7113 3.2033 2.0474
H 5.0588 1.5280 2.2128
H 2.3040 4.7636 1.5719
H 4.0479 3.6759 2.9651

TS-OS-Ap$_{anti}$

Ru -1.1536 -0.4762 0.9063
C -2.9185 1.6712 2.3544
C -2.6342 0.9163 1.2084
C -3.5134 1.0963 0.1066
C -4.5926 1.9880 0.1230
C -4.8266 2.7370 1.2724
C -3.9911 2.5700 2.3841
H -2.2911 1.5690 3.2357

H -5.2253 2.0914 -0.7532
H -5.6521 3.4399 1.3100
H -4.1792 3.1506 3.2832
C -1.8547 -1.1082 -2.2474
C -3.0944 -0.6487 -3.0672
H -3.8102 -1.4620 -3.2129
C -3.1516 0.2842 -1.0323
N -2.1662 -0.5561 -0.9231
H -0.9406 -0.6210 -2.6117
O -3.7341 0.3663 -2.2349
C -1.6420 -2.6024 -2.2558
C -0.6481 -3.1627 -3.0622
C -2.4525 -3.4416 -1.4843
C -0.4703 -4.5473 -3.1061
H -0.0107 -2.5138 -3.6569
C -2.2657 -4.8222 -1.5140
H -3.2133 -3.0023 -0.8477
C -1.2758 -5.3798 -2.3272
H 0.2941 -4.9738 -3.7488
H -2.8957 -5.4678 -0.9091
H -1.1377 -6.4560 -2.3578
H -2.8485 -0.1914 -4.0252
C 0.8782 -3.0380 0.1800
N 0.2030 -2.1470 0.4730
C 1.7222 -4.1554 -0.2089
H 2.7648 -3.8823 -0.0328
H 1.4670 -5.0397 0.3812
H 1.5574 -4.3765 -1.2660
C -3.3674 -2.6766 2.1538
N -2.5601 -1.9645 1.7321
C -4.3902 -3.5703 2.6793
H -4.0804 -3.9671 3.6498
H -5.3317 -3.0277 2.8005
H -4.5469 -4.4041 1.9894
C 5.5889 0.8455 -0.0933
C 5.5164 -0.5388 0.1145
C 6.5964 -1.3667 -0.1488
C 7.7651 -0.7670 -0.6371
C 7.8366 0.6154 -0.8455
C 6.7420 1.4470 -0.5725
C 4.2827 1.4517 0.2704
C 4.1611 -0.8822 0.6222
H 6.5322 -2.4368 0.0157
H 8.6299 -1.3835 -0.8596
H 8.7554 1.0489 -1.2265
H 6.7870 2.5188 -0.7312
O 3.8705 2.5829 0.1528
O 3.6563 -1.9599 0.8514
O 2.1953 0.5023 1.0414
C 1.4660 0.4946 -0.1586
O 1.9839 0.2233 -1.2130
C 0.0262 0.7736 0.0316
H -0.3484 1.3284 -0.8289
N 3.5485 0.3723 0.8249
C 1.1876 0.0929 4.8840
H 0.8063 0.9351 5.4682
H 2.2186 0.3046 4.5858
H 1.1772 -0.8051 5.5077
C 0.3746 -0.1085 3.6938
N -0.2422 -0.2589 2.7262
C 0.6057 3.4271 0.1792
C 0.2422 2.7333 1.2937
H 1.6683 3.4952 -0.0465
H 1.0046 2.3785 1.9735
H -0.7833 2.6805 1.6318
C -0.2989 3.9986 -0.8051
C 0.2411 4.5350 -1.9895
C -1.6997 3.9953 -0.6398
C -0.5893 5.0497 -2.9812
H 1.3191 4.5388 -2.1237
C -2.5251 4.5047 -1.6331
H -2.1418 3.5936 0.2637
C -1.9744 5.0334 -2.8063
H -0.1591 5.4615 -3.8885
H -3.6009 4.4908 -1.4919
H -2.6241 5.4333 -3.5786

Product-OS-Ap_{anti}

Ru -1.5405 -0.6155 0.8898
C -4.2034 0.9087 0.2619
C -3.0351 0.2843 -0.1911
C -2.7874 0.3509 -1.5914
C -3.6270 1.0249 -2.4850
C -4.7706 1.6477 -1.9915
C -5.0548 1.5820 -0.6229
H -4.4535 0.8869 1.3187
H -3.3815 1.0594 -3.5419
H -5.4387 2.1754 -2.6639
H -5.9499 2.0657 -0.2410
C 0.2255 -1.6824 -1.7002
C 0.1560 -1.1403 -3.1470
H 0.2097 -1.9082 -3.9173
C -1.5733 -0.3381 -1.9766
N -0.8238 -0.8652 -1.0534
H 1.1890 -1.4673 -1.2421
O -1.1624 -0.5052 -3.2391
C -0.0869 -3.1566 -1.5472
C 0.6715 -3.9393 -0.6738
C -1.1506 -3.7428 -2.2461
C 0.3757 -5.2935 -0.5015
H 1.5009 -3.4842 -0.1431
C -1.4378 -5.0969 -2.0868
H -1.7522 -3.1413 -2.9221
C -0.6751 -5.8763 -1.2109
H 0.9760 -5.8966 0.1730
H -2.2490 -5.5478 -2.6508
H -0.8911 -6.9343 -1.0952
H 0.9026 -0.3632 -3.3282
C 1.3073 -1.5525 2.3511
N 0.2634 -1.3828 1.8810
C 2.6312 -1.7160 2.9297
H 2.5639 -2.1671 3.9228
H 3.2386 -2.3413 2.2719
H 3.0949 -0.7281 3.0030
C -2.9592 -3.3711 0.6715
N -2.4397 -2.3423 0.7747
C -3.6007 -4.6711 0.5506
H -4.3363 -4.8050 1.3486
H -4.1037 -4.7465 -0.4169
H -2.8445 -5.4576 0.6096
C 5.2755 -0.2996 -0.1146
C 5.5040 1.0710 -0.3017
C 6.7744 1.6140 -0.1925
C 7.8249 0.7389 0.1161
C 7.5973 -0.6301 0.3015
C 6.3104 -1.1722 0.1858
C 3.8259 -0.5748 -0.2846
C 4.2095 1.7354 -0.6030
H 6.9410 2.6756 -0.3382
H 8.8330 1.1278 0.2138
H 8.4327 -1.2807 0.5381
H 6.1257 -2.2322 0.3229
O 3.1948 -1.5983 -0.0982
O 3.9095 2.8970 -0.7125
O 1.9393 0.8895 -0.7458
C 1.4621 1.2880 0.5102
O 2.1464 1.2444 1.5023
C 0.0995 1.8151 0.3710
H -0.4023 1.6303 -0.5672
N 3.2956 0.6440 -0.7359
C -3.5728 0.1925 5.0113
H -3.0863 -0.3798 5.8061
H -4.6266 -0.0962 4.9612
H -3.5108 1.2573 5.2538
C -2.9227 -0.0657 3.7337
N -2.4112 -0.2636 2.7125
C -0.0733 3.2450 0.9300
C -0.7079 2.1003 1.6447
H 0.8288 3.6339 1.3921
H -0.2540 1.7716 2.5720
H -1.7861 2.0227 1.5803
C -0.8590 4.2293 0.1369
C -0.3542 5.5250 -0.0248
C -2.0791 3.8940 -0.4672
C -1.0571 6.4715 -0.7698
H 0.5938 5.7918 0.4337
C -2.7774 4.8391 -1.2147
H -2.4936 2.8980 -0.3627
C -2.2706 6.1312 -1.3679
H -0.6539 7.4728 -0.8846
H -3.7185 4.5587 -1.6767
H -2.8166 6.8667 -1.9503

Ap_{syn} position

Int3-IS-Ap_{syn}

Ru -1.3574 0.1841 0.1378
C -1.5105 0.3685 3.2486
C -1.9633 -0.2254 2.0705
C -3.0152 -1.1674 2.1711
C -3.6076 -1.5008 3.3943
C -3.1413 -0.8891 4.5540
C -2.0962 0.0372 4.4761
H -0.6952 1.0824 3.2239
H -4.4132 -2.2274 3.4260
H -3.5820 -1.1343 5.5144
H -1.7278 0.5043 5.3852
C -3.2117 -2.2439 -1.2966

C	-4.4144	-2.9898	-0.6554
H	-4.3824	-4.0694	-0.7979
C	-3.3854	-1.7523	0.9023
N	-2.7890	-1.3615	-0.1861
H	-3.5424	-1.6384	-2.1438
O	-4.2940	-2.7232	0.7752
C	-2.0729	-3.1436	-1.7314
C	-1.5757	-3.0883	-3.0350
C	-1.4845	-4.0250	-0.8133
C	-0.4871	-3.8783	-3.4136
H	-2.0432	-2.4299	-3.7627
C	-0.4003	-4.8149	-1.1887
H	-1.8691	-4.0878	0.2015
C	0.1072	-4.7354	-2.4890
H	-0.1066	-3.8220	-4.4284
H	0.0526	-5.4888	-0.4687
H	0.9580	-5.3437	-2.7776
H	-5.3790	-2.5949	-0.9829
C	-3.6937	2.8169	0.2218
N	-3.0726	1.8461	0.1342
C	-4.4496	4.0564	0.3176
H	-3.8901	4.8525	-0.1819
H	-5.4249	3.9429	-0.1631
H	-4.5994	4.3224	1.3673
C	5.4045	0.1745	0.8239
C	5.5932	-1.0458	0.1597
C	6.8165	-1.3857	-0.3961
C	7.8611	-0.4600	-0.2708
C	7.6736	0.7581	0.3932
C	6.4342	1.0936	0.9541
C	3.9969	0.2550	1.2945
C	4.3154	-1.8066	0.1772
H	6.9514	-2.3315	-0.9092
H	8.8326	-0.6895	-0.6961
H	8.5030	1.4531	0.4734
H	6.2807	2.0343	1.4717
O	3.3804	1.1727	1.7956
O	3.9849	-2.8302	-0.3671
O	2.1219	-1.2737	1.0808
C	1.4373	-0.7443	-0.0065
O	1.9795	-0.1159	-0.8818
C	-0.0026	-1.0743	0.1221
N	3.4783	-1.0254	1.0265
C	1.4811	3.5966	1.5784
H	2.5030	3.2135	1.6365
H	1.1638	3.9214	2.5734
H	1.4243	4.4403	0.8859
C	0.6158	2.5288	1.1077
N	-0.0794	1.6850	0.7300
H	-0.1913	-2.1471	0.2142
C	-1.5709	0.5187	-2.4814
C	-0.5487	1.3922	-2.2787
H	-1.3502	-0.5217	-2.6800
H	-2.6008	0.8401	-2.5984
H	0.4556	0.9809	-2.2187
C	-0.6297	2.8527	-2.1916
C	0.5462	3.5675	-1.9085
C	-1.8183	3.5727	-2.4029
C	0.5335	4.9580	-1.8131
H	1.4723	3.0172	-1.7679
C	-1.8318	4.9612	-2.3076
H	-2.7293	3.0443	-2.6621
C	-0.6582	5.6594	-2.0034
H	1.4547	5.4962	-1.6102
H	-2.7511	5.5072	-2.4992
H	-0.6700	6.7429	-1.9413

TS4-IS-Ap$_{syn}$

Ru	-1.2456	0.3347	0.2192
C	-0.7752	0.8006	3.2778
C	-1.4872	0.1625	2.2625
C	-2.5099	-0.7375	2.6420
C	-2.8234	-0.9954	3.9813
C	-2.0953	-0.3472	4.9752
C	-1.0738	0.5403	4.6205
H	0.0259	1.4895	3.0351
H	-3.6165	-1.6932	4.2304
H	-2.3164	-0.5335	6.0209
H	-0.5014	1.0345	5.4005
C	-3.5893	-1.9434	-0.6242
C	-4.6480	-2.5702	0.3323
H	-4.6920	-3.6573	0.2664
C	-3.1733	-1.3708	1.5230
N	-2.7830	-1.1290	0.3061
H	-4.0750	-1.2887	-1.3549
O	-4.1948	-2.2194	1.6714
C	-2.7525	-2.9684	-1.3605
C	-2.8301	-3.0803	-2.7512
C	-1.8947	-3.8200	-0.6513
C	-2.0474	-4.0169	-3.4310
H	-3.5001	-2.4285	-3.3066
C	-1.1117	-4.7535	-1.3281
H	-1.8310	-3.7441	0.4308
C	-1.1831	-4.8497	-2.7208
H	-2.1122	-4.0922	-4.5117
H	-0.4441	-5.4034	-0.7716
H	-0.5696	-5.5735	-3.2471
H	-5.6461	-2.1472	0.1995
C	-3.3541	2.9297	0.6376
N	-2.7555	1.9482	0.5206
C	-4.0830	4.1831	0.7625
H	-5.1526	4.0170	0.6091
H	-3.9289	4.6087	1.7577
H	-3.7154	4.8849	0.0084
C	5.4768	-0.0041	0.6953
C	5.6392	-1.1762	-0.0567
C	6.8694	-1.5311	-0.5873
C	7.9487	-0.6708	-0.3445
C	7.7875	0.4988	0.4078
C	6.5408	0.8497	0.9424
C	4.0545	0.1092	1.1136
C	4.3282	-1.8714	-0.1528
H	6.9839	-2.4391	-1.1691
H	8.9268	-0.9137	-0.7464
H	8.6435	1.1433	0.5786
H	6.4076	1.7525	1.5286
O	3.4607	1.0160	1.6602
O	3.9744	-2.8304	-0.7924
O	2.1257	-1.3108	0.7046
C	1.5087	-0.6638	-0.3602
O	2.1104	0.0236	-1.1478
C	0.0570	-0.9578	-0.2760
N	3.4910	-1.1200	0.7216
C	2.0157	3.6123	0.7051
H	2.9440	3.1240	1.0135
H	1.7208	4.3455	1.4608
H	2.1532	4.1217	-0.2529
C	0.9882	2.5957	0.5649
N	0.1809	1.7801	0.4263
H	-0.1801	-2.0173	-0.3929
C	-0.7710	-0.2019	-2.1302
C	-1.6464	0.8987	-2.0189
H	0.2403	-0.0334	-2.4803
H	-1.2033	-1.1718	-2.3395
H	-2.7103	0.6809	-2.0619
C	-1.2839	2.2882	-2.3293
C	-2.3107	3.2375	-2.4681
C	0.0477	2.7081	-2.5012
C	-2.0214	4.5716	-2.7473
H	-3.3437	2.9161	-2.3676
C	0.3346	4.0404	-2.7818
H	0.8589	1.9956	-2.3977
C	-0.6950	4.9798	-2.8985
H	-2.8287	5.2879	-2.8670
H	1.3661	4.3477	-2.9268
H	-0.4651	6.0160	-3.1252

Int4-IS-Ap$_{syn}$

Ru	1.0786	-0.3291	0.3547
C	0.3856	-1.4678	3.1416
C	1.1311	-0.5627	2.3883
C	1.9305	0.3838	3.0653
C	1.9708	0.4525	4.4611
C	1.1999	-0.4440	5.1981
C	0.4099	-1.3914	4.5395
H	-0.2278	-2.2169	2.6566
H	2.5893	1.1979	4.9505
H	1.2130	-0.4069	6.2822
H	-0.1926	-2.0834	5.1207
C	3.4532	2.0895	0.2379
C	4.0669	2.8440	1.4493
H	3.6989	3.8695	1.5273
C	2.6680	1.2590	2.1811
N	2.4675	1.2042	0.8989
H	4.2118	1.4573	-0.2402
O	3.5988	2.1107	2.6187
C	2.8505	2.9971	-0.8077
C	3.3448	2.9913	-2.1147
C	1.7826	3.8434	-0.4831
C	2.7667	3.8019	-3.0940
H	4.1804	2.3448	-2.3707
C	1.1996	4.6484	-1.4595
H	1.3948	3.8581	0.5319
C	1.6879	4.6247	-2.7690
H	3.1563	3.7876	-4.1068
H	0.3636	5.2905	-1.2018
H	1.2317	5.2499	-3.5297
H	5.1565	2.8396	1.4633

C	3.3211	-2.8792	0.6302
N	2.6492	-1.9394	0.6664
C	4.1523	-4.0711	0.5458
H	4.1040	-4.4641	-0.4738
H	5.1896	-3.8268	0.7900
H	3.7929	-4.8305	1.2452
C	-5.5084	-0.2041	0.4181
C	-5.7356	0.9509	-0.3427
C	-6.9812	1.2280	-0.8834
C	-8.0089	0.3061	-0.6418
C	-7.7832	-0.8464	0.1199
C	-6.5212	-1.1180	0.6649
C	-4.0836	-0.2276	0.8452
C	-4.4671	1.7239	-0.4327
H	-7.1456	2.1239	-1.4718
H	-8.9972	0.4875	-1.0516
H	-8.6005	-1.5396	0.2905
H	-6.3388	-2.0063	1.2601
O	-3.4455	-1.0944	1.4099
O	-4.1758	2.7081	-1.0659
O	-2.2418	1.2970	0.4418
C	-1.5622	0.6742	-0.6114
O	-2.1251	-0.0446	-1.4035
C	-0.1340	1.0184	-0.5124
N	-3.5878	1.0213	0.4377
C	-2.3011	-3.4653	-0.1983
H	-3.0671	-3.1964	0.5341
H	-1.9949	-4.5064	-0.0683
H	-2.7101	-3.3276	-1.2035
C	-1.1719	-2.5698	-0.0219
N	-0.2968	-1.8238	0.0951
H	0.0288	2.0508	-0.2211
C	0.6906	0.6086	-1.8175
C	1.8869	-0.3391	-1.6221
H	-0.0221	0.1756	-2.5159
H	1.1039	1.5445	-2.1942
H	2.8433	0.1745	-1.5823
C	1.9639	-1.6174	-2.3290
C	3.2327	-2.1919	-2.5404
C	0.8295	-2.3122	-2.7913
C	3.3660	-3.4273	-3.1675
H	4.1161	-1.6532	-2.2084
C	0.9636	-3.5463	-3.4176
H	-0.1566	-1.8835	-2.6545
C	2.2288	-4.1131	-3.6029
H	4.3533	-3.8470	-3.3360
H	0.0810	-4.0670	-3.7756
H	2.3279	-5.0726	-4.1003

TS5-IS-Ap$_{syn}$

Ru	0.3991	-0.3520	0.1397
C	-1.5092	-2.6225	1.1579
C	-1.1830	-1.2679	1.1322
C	-1.9974	-0.3681	1.8512
C	-3.1382	-0.7909	2.5419
C	-3.4567	-2.1473	2.5408
C	-2.6407	-3.0562	1.8584
H	-0.8955	-3.3418	0.6269
H	-3.7493	-0.0707	3.0759
H	-4.3325	-2.4987	3.0760
H	-2.8886	-4.1138	1.8728
C	0.0532	2.6199	1.7225
C	-1.2275	3.1403	2.4120
H	-1.8084	3.7932	1.7551
C	-1.4861	0.9889	1.8756
N	-0.4116	1.3025	1.2112
H	0.8246	2.4089	2.4751
O	-2.0148	1.9344	2.6578
C	0.6306	3.5691	0.7060
C	1.9054	4.1060	0.9092
C	-0.0982	3.9420	-0.4287
C	2.4574	4.9902	-0.0189
H	2.4717	3.8277	1.7945
C	0.4575	4.8117	-1.3647
H	-1.0861	3.5305	-0.6034
C	1.7366	5.3368	-1.1624
H	3.4482	5.4003	0.1474
H	-0.1076	5.0759	-2.2526
H	2.1671	6.0155	-1.8916
H	-1.0525	3.6307	3.3683
C	1.7842	-1.5877	2.7050
N	1.2438	-1.0774	1.8191
C	2.4542	-2.2600	3.8073
H	2.7168	-1.5381	4.5856
H	1.7990	-3.0231	4.2365
H	3.3693	-2.7280	3.4344
C	-3.4716	0.3468	-1.1007
C	-3.4452	-1.0537	-1.1141
C	-4.5102	-1.7989	-0.6280
C	-5.6073	-1.0969	-0.1179
C	-5.6290	0.3049	-0.0961
C	-4.5556	1.0509	-0.5925
C	-2.2401	0.8568	-1.7250
C	-2.1765	-1.5117	-1.7167
H	-4.4842	-2.8818	-0.6451
H	-6.4608	-1.6460	0.2659
H	-6.4989	0.8165	0.3023
H	-4.5676	2.1353	-0.5978
O	-1.9482	1.9682	-2.0914
O	-1.8245	-2.6063	-2.0716
O	-0.5516	-0.3310	-2.9880
C	0.7957	0.0434	-2.7311
O	1.6166	-0.4105	-3.4910
C	0.9645	0.9089	-1.5731
N	-1.3608	-0.3013	-1.8429
C	1.2816	-3.8141	-2.7550
H	2.0320	-4.5658	-2.4984
H	1.5953	-3.2661	-3.6488
H	0.3191	-4.2949	-2.9488
C	1.1190	-2.8518	-1.6819
N	0.9577	-2.0218	-0.8938
H	0.2306	1.7045	-1.5272
C	2.3853	1.3231	-1.2514
C	2.6188	0.6061	0.0562
H	2.5048	1.2211	0.9438
H	3.0821	1.0104	-2.0314
C	3.6369	-0.4291	0.1620
C	4.3064	-0.6269	1.3880
C	4.0140	-1.2291	-0.9383
C	5.3020	-1.5921	1.5171
H	4.0642	0.0176	2.2285
C	4.9968	-2.2022	-0.8012
H	3.5205	-1.0910	-1.8939
C	5.6417	-2.3945	0.4256
H	5.8271	-1.7065	2.4611
H	5.2753	-2.8084	-1.6579
H	6.4170	-3.1478	0.5223
H	2.4682	2.4041	-1.1295

Product-IS-Ap$_{syn}$

Ru	0.5160	-0.6270	0.4846
C	-2.1748	-1.9022	1.2454
C	-1.2744	-0.8402	1.3774
C	-1.6793	0.2602	2.1760
C	-2.9355	0.3217	2.7879
C	-3.8204	-0.7400	2.6203
C	-3.4329	-1.8453	1.8567
H	-1.9126	-2.7688	0.6502
H	-3.2067	1.1939	3.3742
H	-4.8024	-0.7107	3.0799
H	-4.1235	-2.6739	1.7276
C	1.2827	2.3457	1.8396
C	0.3350	3.2544	2.6768
H	-0.0104	4.1228	2.1126
C	-0.6840	1.3089	2.2437
N	0.4233	1.1757	1.5796
H	2.1400	2.0174	2.4396
O	-0.8289	2.4227	2.9639
C	1.7959	2.9901	0.5721
C	3.1696	3.1427	0.3646
C	0.8994	3.4243	-0.4143
C	3.6503	3.7095	-0.8191
H	3.8675	2.8186	1.1327
C	1.3792	3.9892	-1.5949
H	-0.1671	3.2850	-0.2745
C	2.7545	4.1301	-1.8022
H	4.7194	3.8255	-0.9678
H	0.6787	4.3162	-2.3571
H	3.1242	4.5707	-2.7226
H	0.7615	3.5729	3.6281
C	2.0598	-2.0590	2.7892
N	1.4668	-1.5199	1.9525
C	2.8270	-2.7402	3.8206
H	2.9912	-2.0758	4.6737
H	2.2922	-3.6300	4.1644
H	3.7951	-3.0408	3.4105
C	-3.9447	-0.4639	-1.3278
C	-3.9800	0.7874	-0.6995
C	-5.1553	1.3039	-0.1775
C	-6.3116	0.5229	-0.2997
C	-6.2777	-0.7263	-0.9315
C	-5.0861	-1.2388	-1.4608
C	-2.5498	-0.7632	-1.7373
C	-2.6153	1.3666	-0.6894
H	-5.1689	2.2718	0.3111
H	-7.2503	0.8904	0.1016
H	-7.1911	-1.3065	-1.0110
H	-5.0500	-2.2059	-1.9499

O -2.0656 -1.7524 -2.2364
O -2.2005 2.3940 -0.2033
O -0.4712 0.4219 -1.2573
C 0.2700 0.5874 -2.4859
O -0.2830 0.6345 -3.5389
C 1.6911 0.6120 -2.1159
N -1.8428 0.4234 -1.4124
C -0.1560 -4.3754 -2.1337
H 0.6853 -4.6013 -2.7951
H -1.0208 -4.0793 -2.7345
H -0.4020 -5.2717 -1.5576
C 0.1714 -3.2728 -1.2451
N 0.3738 -2.3568 -0.5660
H 1.8930 1.1382 -1.1915
C 2.7768 0.5817 -3.1477
C 2.5125 -0.6682 -2.3544
H 2.4719 0.5840 -4.1890
H 3.6636 1.1609 -2.9161
H 1.9850 -1.4477 -2.8949
C 3.3853 -1.1668 -1.2537
C 3.9768 -0.3002 -0.3216
C 3.6145 -2.5433 -1.1264
C 4.7740 -0.8001 0.7071
H 3.8318 0.7711 -0.4024
C 4.4104 -3.0444 -0.0984
H 3.1690 -3.2251 -1.8446
C 4.9952 -2.1732 0.8233
H 5.2332 -0.1123 1.4108
H 4.5843 -4.1135 -0.0248
H 5.6373 -2.5596 1.6093

TS-OS-Ap$_{syn}$

Ru -1.6860 -1.2945 -0.2018
C -3.7122 -0.3359 -2.3850
C -3.1919 -0.2246 -1.0873
C -3.8075 0.7454 -0.2488
C -4.8537 1.5738 -0.6733
C -5.3241 1.4473 -1.9776
C -4.7546 0.4882 -2.8240
H -3.2916 -1.0667 -3.0703
H -5.2851 2.2987 0.0106
H -6.1301 2.0806 -2.3328
H -5.1291 0.3846 -3.8390
C -1.8180 0.2366 2.7547
C -2.9308 1.1992 3.2592
H -2.5429 2.1001 3.7335
C -3.2399 0.7751 1.0807
N -2.2728 -0.0370 1.3783
H -1.8232 -0.6979 3.3225
O -3.6547 1.5986 2.0565
C -0.4219 0.8255 2.7869
C 0.6375 0.0980 3.3306
C -0.1774 2.1067 2.2735
C 1.9238 0.6376 3.3735
H 0.4528 -0.8963 3.7277
C 1.1045 2.6491 2.3171
H -0.9865 2.6824 1.8324
C 2.1571 1.9165 2.8710
H 2.7389 0.0558 3.7903
H 1.2790 3.6397 1.9122
H 3.1557 2.3412 2.9053
H -3.6444 0.7057 3.9236
C 0.5052 -3.0694 1.6269
N -0.2389 -2.4642 0.9798
C 1.4375 -3.8288 2.4466
H 1.1200 -3.8050 3.4929
H 1.4705 -4.8688 2.1106
H 2.4280 -3.3776 2.3553
C -4.1143 -3.3701 0.7274
N -3.2154 -2.7016 0.4385
C -5.2548 -4.2055 1.0787
H -5.1179 -5.2157 0.6834
H -5.3602 -4.2612 2.1656
H -6.1690 -3.7796 0.6557
C 5.3638 0.1332 -0.8740
C 5.3169 -0.8549 0.1194
C 6.4364 -1.6009 0.4500
C 7.6213 -1.3320 -0.2500
C 7.6675 -0.3460 -1.2417
C 6.5307 0.4068 -1.5688
C 4.0163 0.7506 -0.9964
C 3.9366 -0.9148 0.6750
H 6.3915 -2.3631 1.2202
H 8.5182 -1.8978 -0.0202
H 8.5995 -0.1623 -1.7661
H 6.5562 1.1731 -2.3359
O 3.5980 1.5912 -1.7642
O 3.4536 -1.6577 1.5030
O 1.9220 0.3562 0.1484
C 1.1473 -0.4024 -0.7558
O 1.6500 -1.2180 -1.4854
C -0.2665 0.0318 -0.6134
N 3.2672 0.1331 0.0241
C -0.1474 -3.6240 -3.8951
H -0.6460 -3.3350 -4.8246
H -0.2108 -4.7099 -3.7839
H 0.9059 -3.3321 -3.9478
C -0.7616 -2.9577 -2.7567
N -1.1957 -2.3980 -1.8407
C -0.5012 0.8165 -2.4965
C 0.3107 1.9068 -2.2357
H -0.1043 0.0016 -3.0903
H -1.5790 0.9039 -2.4807
H 1.3761 1.8155 -2.4364
H -0.3203 0.9601 -0.0523
C -0.0877 3.1063 -1.5408
C 0.9144 4.0221 -1.1582
C -1.4265 3.3572 -1.1647
C 0.5877 5.1597 -0.4270
H 1.9455 3.8122 -1.4258
C -1.7440 4.4875 -0.4257
H -2.2110 2.6616 -1.4397
C -0.7393 5.3921 -0.0556
H 1.3637 5.8622 -0.1404
H -2.7739 4.6688 -0.1352
H -0.9936 6.2766 0.5202

Product-OS-Ap$_{syn}$

Ru -1.4514 -1.3534 0.2576
C -3.1064 -1.9183 -2.3386
C -2.9609 -1.2263 -1.1297
C -3.9972 -0.3088 -0.8010
C -5.1025 -0.0741 -1.6271
C -5.2039 -0.7734 -2.8266
C -4.2065 -1.6915 -3.1747
H -2.3503 -2.6379 -2.6402
H -5.8608 0.6447 -1.3312
H -6.0497 -0.6091 -3.4856
H -4.2895 -2.2381 -4.1102
C -2.6766 0.9162 2.3659
C -3.8521 1.8964 2.0981
H -3.5022 2.8904 1.8100
C -3.7533 0.3877 0.4446
N -2.6823 0.1128 1.1290
H -2.9064 0.2511 3.2072
O -4.5558 1.3320 0.9474
C -1.3441 1.5795 2.6210
C -0.6343 1.3105 3.7927
C -0.7923 2.4432 1.6678
C 0.6163 1.8920 4.0102
H -1.0594 0.6409 4.5356
C 0.4624 3.0101 1.8748
H -1.3372 2.6658 0.7574
C 1.1701 2.7367 3.0472
H 1.1540 1.6880 4.9314
H 0.8828 3.6653 1.1196
H 2.1445 3.1863 3.2116
H -4.5618 1.9712 2.9212
C 1.1645 -1.2512 2.3382
N 0.1591 -1.3559 1.7772
C 2.4492 -1.0871 2.9968
H 3.2027 -1.6579 2.4474
H 2.7106 -0.0268 2.9777
H 2.3990 -1.4368 4.0310
C -3.1295 -3.5920 1.6152
N -2.4769 -2.7679 1.1289
C -3.9451 -4.6349 2.2215
H -4.7818 -4.8847 1.5628
H -3.3439 -5.5336 2.3861
H -4.3410 -4.2943 3.1824
C 5.5587 -0.7500 -0.1725
C 5.6616 0.4789 -0.8407
C 6.8058 1.2567 -0.7531
C 7.8582 0.7682 0.0327
C 7.7564 -0.4592 0.6989
C 6.5979 -1.2414 0.6030
C 4.2235 -1.3388 -0.4488
C 4.3913 0.7318 -1.5663
H 6.8756 2.2063 -1.2721
H 8.7686 1.3507 0.1285
H 8.5898 -0.8087 1.2994
H 6.5115 -2.1945 1.1135
O 3.6801 -2.3168 0.0266
O 3.9696 1.7115 -2.1317
O 2.3201 -0.4942 -1.6889
C 1.6091 0.1824 -0.6808

O 2.1383 0.5081 0.3504
C 0.2368 0.4635 -1.1173
N 3.6759 -0.4973 -1.4399
C 1.6530 -3.9655 -1.9903
H 1.7527 -3.6189 -3.0226
H 1.4608 -5.0417 -1.9915
H 2.5777 -3.7522 -1.4459
C 0.5683 -3.2564 -1.3312
N -0.2621 -2.6604 -0.7863
C -0.2500 0.2213 -2.5309
C 0.1192 1.6150 -2.1839
H 0.4265 -0.3242 -3.1766
H -1.2921 -0.0455 -2.6424
H 1.0908 1.9549 -2.5331
H -0.4556 0.6366 -0.2873
C -0.8630 2.6876 -1.8760
C -0.3917 3.9655 -1.5440
C -2.2390 2.4352 -1.8118
C -1.2744 4.9596 -1.1244
H 0.6733 4.1728 -1.6029
C -3.1218 3.4263 -1.3835
H -2.6314 1.4645 -2.0870
C -2.6418 4.6893 -1.0305
H -0.8951 5.9432 -0.8663
H -4.1831 3.2068 -1.3265
H -3.3281 5.4618 -0.6984

Eq_{trans} position

Int3-IS-Eq_{trans}

Ru 0.7803 -0.5424 0.0209
C 0.1988 -2.5934 2.2720
C 1.0465 -2.1841 1.2394
C 2.1778 -2.9857 0.9638
C 2.4653 -4.1456 1.6969
C 1.6126 -4.5264 2.7264
C 0.4834 -3.7500 3.0059
H -0.6938 -2.0232 2.5076
H 3.3467 -4.7297 1.4520
H 1.8195 -5.4202 3.3053
H -0.1884 -4.0480 3.8059
C 3.7954 -1.1449 -1.7223
C 4.6722 -2.4260 -1.6219
H 5.7311 -2.2207 -1.4660
C 3.0089 -2.5173 -0.1247
N 2.6799 -1.4706 -0.8139
H 3.4193 -1.0160 -2.7411
O 4.1683 -3.1152 -0.4430
C 4.5087 0.1242 -1.2935
C 4.6709 1.1877 -2.1859
C 5.0276 0.2389 0.0024
C 5.3258 2.3575 -1.7907
H 4.2862 1.1018 -3.1990
C 5.6987 1.3962 0.3932
H 4.9126 -0.5819 0.7041
C 5.8402 2.4641 -0.4987
H 5.4431 3.1751 -2.4947
H 6.1279 1.4598 1.3891
H 6.3653 3.3638 -0.1936
C 1.0579 0.9090 -1.9675
C 0.1890 1.7578 -1.3376
H 2.1253 1.0859 -1.9429
H 0.6879 0.1977 -2.6962
H -0.8740 1.6112 -1.5035
H 4.5419 -3.0917 -2.4789
C -1.0433 -2.4840 -1.7466
N -0.3848 -1.7433 -1.1507
C -1.8837 -3.4128 -2.4837
H -1.7044 -4.4320 -2.1302
H -2.9278 -3.1401 -2.3085
H -1.6576 -3.3582 -3.5521
C 2.4554 1.3592 1.9367
N 1.9052 0.6182 1.2409
C 3.1456 2.3086 2.7928
H 2.5417 3.2152 2.8828
H 3.3138 1.8761 3.7827
H 4.1052 2.5662 2.3377
C -6.1359 -0.3357 -0.5157
C -6.0749 0.9660 0.0047
C -7.2167 1.6345 0.4180
C -8.4381 0.9578 0.2993
C -8.4991 -0.3407 -0.2199
C -7.3410 -1.0091 -0.6394
C -4.7632 -0.7882 -0.8640
C -4.6583 1.4074 0.0214
H -7.1594 2.6397 0.8209
H -9.3529 1.4467 0.6174
H -9.4603 -0.8383 -0.2955
H -7.3784 -2.0161 -1.0401
O -4.3599 -1.8604 -1.2561
O -4.1223 2.3878 0.4849
O -2.5967 0.3163 -0.5803
C -2.1540 -0.1233 0.6739
O -2.8748 -0.6492 1.4792
C -0.7246 0.1850 0.8629
H -0.6084 0.9453 1.6451
N -3.9726 0.3650 -0.6583
C 0.5434 2.9285 -0.5274
C -0.4717 3.5764 0.1979
C 1.8580 3.4216 -0.4529
C -0.1768 4.6836 0.9903
H -1.4923 3.2038 0.1459
C 2.1463 4.5357 0.3299
H 2.6553 2.9433 -1.0110
C 1.1324 5.1674 1.0577
H -0.9691 5.1741 1.5465
H 3.1626 4.9164 0.3660
H 1.3590 6.0405 1.6619

TS4-IS-Eq_{trans}

Ru 0.8270 -0.5569 0.0576
C 0.3674 -2.7245 2.1044
C 1.2267 -2.2913 1.0942
C 2.3545 -3.0691 0.7835
C 2.6490 -4.2535 1.4696
C 1.7952 -4.6662 2.4880
C 0.6635 -3.9041 2.7994
H -0.5254 -2.1605 2.3565
H 3.5292 -4.8299 1.2035
H 2.0025 -5.5792 3.0358
H -0.0037 -4.2333 3.5908
C 3.7998 -0.9743 -1.7939
C 4.7241 -2.2248 -1.8438
H 5.7817 -1.9936 -1.7202
C 3.1398 -2.5076 -0.2916
N 2.7375 -1.4240 -0.8740
H 3.3760 -0.7694 -2.7809
O 4.3029 -3.0309 -0.7036
C 4.4828 0.2779 -1.2758
C 4.5016 1.4488 -2.0383
C 5.1078 0.2726 -0.0220
C 5.1181 2.6060 -1.5541
H 4.0345 1.4581 -3.0199
C 5.7419 1.4184 0.4544
H 5.1055 -0.6320 0.5791
C 5.7388 2.5929 -0.3054
H 5.1217 3.5085 -2.1568
H 6.2547 1.3918 1.4118
H 6.2344 3.4842 0.0663
C 0.9663 0.7509 -1.6141
C -0.0253 1.5286 -0.8771
H 1.9642 1.1602 -1.6992
H 0.6179 0.2160 -2.4921
H -1.0038 1.4971 -1.3484
H 4.5735 -2.8225 -2.7461
C -1.0542 -2.4795 -1.6699
N -0.3802 -1.7445 -1.0861
C -1.9193 -3.4036 -2.3833
H -1.5967 -4.4314 -2.1955
H -2.9395 -3.2621 -2.0158
H -1.8781 -3.2043 -3.4576
C 2.5359 1.2789 2.0173
N 1.9897 0.5542 1.3017
C 3.2070 2.2270 2.8887
H 2.7711 3.2175 2.7266
H 3.0846 1.9372 3.9356
H 4.2698 2.2602 2.6375
C -6.0909 -0.5525 -0.2851
C -6.0827 0.8449 -0.1609
C -7.2408 1.5536 0.1186
C -8.4235 0.8187 0.2765
C -8.4318 -0.5757 0.1529
C -7.2579 -1.2851 -0.1337
C -4.7121 -1.0226 -0.5780
C -4.6955 1.3328 -0.3585
H -7.2241 2.6336 0.2153
H -9.3492 1.3380 0.5016
H -9.3638 -1.1155 0.2849
H -7.2540 -2.3655 -0.2274
O -4.2625 -2.1443 -0.6707
O -4.1987 2.4261 -0.2157
O -2.6070 0.1958 -0.7447
C -2.1100 0.2190 0.5699
O -2.8133 -0.0018 1.5220
C -0.6741 0.5257 0.6574
H -0.4993 1.2134 1.4836
N -3.9833 0.1727 -0.7703
C 0.3186 2.8625 -0.2945

C	-0.7344	3.6740	0.1574
C	1.6337	3.3355	-0.1926
C	-0.4747	4.9258	0.7110
H	-1.7603	3.3238	0.0690
C	1.8905	4.5886	0.3645
H	2.4665	2.7413	-0.5485
C	0.8396	5.3852	0.8238
H	-1.2996	5.5431	1.0516
H	2.9146	4.9460	0.4225
H	1.0412	6.3617	1.2525

Int4-IS-Eq$_{trans}$

Ru	0.8184	-0.5977	0.0894
C	0.3105	-2.7915	1.9779
C	1.2241	-2.3677	1.0174
C	2.3639	-3.1344	0.7439
C	2.6253	-4.3197	1.4399
C	1.7262	-4.7298	2.4215
C	0.5813	-3.9703	2.6872
H	-0.5975	-2.2296	2.1809
H	3.5150	-4.8979	1.2126
H	1.9082	-5.6442	2.9759
H	-0.1206	-4.3024	3.4464
C	3.8474	-0.9701	-1.7465
C	4.7876	-2.2103	-1.8071
H	5.8387	-1.9704	-1.6498
C	3.1780	-2.5452	-0.2947
N	2.7789	-1.4469	-0.8475
H	3.4316	-0.7543	-2.7347
O	4.3482	-3.0544	-0.6996
C	4.5115	0.2810	-1.2029
C	4.5298	1.4611	-1.9507
C	5.1224	0.2641	0.0577
C	5.1347	2.6157	-1.4463
H	4.0711	1.4800	-2.9360
C	5.7435	1.4081	0.5550
H	5.1195	-0.6479	0.6477
C	5.7421	2.5912	-0.1912
H	5.1385	3.5256	-2.0377
H	6.2448	1.3735	1.5183
H	6.2283	3.4809	0.1964
C	0.9799	0.6848	-1.5158
C	-0.0742	1.4917	-0.7651
H	1.9625	1.1305	-1.6026
H	0.6365	0.2177	-2.4350
H	-0.9496	1.5406	-1.4147
H	4.6670	-2.7854	-2.7282
C	-1.0832	-2.4244	-1.7198
N	-0.4064	-1.7338	-1.0871
C	-1.9442	-3.2948	-2.5017
H	-1.6273	-4.3343	-2.3804
H	-2.9675	-3.1713	-2.1371
H	-1.8889	-3.0230	-3.5594
C	2.5041	1.2816	2.0346
N	1.9786	0.5196	1.3424
C	3.1426	2.2786	2.8746
H	2.7452	3.2616	2.6030
H	2.9380	2.0761	3.9292
H	4.2206	2.2678	2.6980
C	-6.0791	-0.5533	-0.2642
C	-6.0981	0.8407	-0.1115
C	-7.2629	1.5173	0.2150
C	-8.4243	0.7531	0.3904
C	-8.4056	-0.6385	0.2383
C	-7.2248	-1.3151	-0.0955
C	-4.6985	-0.9852	-0.6048
C	-4.7271	1.3624	-0.3376
H	-7.2670	2.5953	0.3336
H	-9.3545	1.2467	0.6519
H	-9.3214	-1.2016	0.3848
H	-7.1998	-2.3931	-0.2116
O	-4.2302	-2.0969	-0.7317
O	-4.2513	2.4632	-0.1838
O	-2.6276	0.2821	-0.8068
C	-2.0937	0.2833	0.5008
O	-2.7710	0.0003	1.4556
C	-0.6760	0.6665	0.5377
H	-0.4953	1.2252	1.4536
N	-4.0019	0.2271	-0.7947
C	0.3206	2.8774	-0.2942
C	1.6409	3.3341	-0.2320
C	-0.7139	3.7314	0.1180
C	1.9241	4.6159	0.2472
H	2.4644	2.7079	-0.5517
C	-0.4301	5.0109	0.5904
H	-1.7459	3.3923	0.0623
C	0.8920	5.4567	0.6641
H	2.9553	4.9565	0.2768
H	-1.2427	5.6606	0.8993
H	1.1126	6.4542	1.0306

TS5-IS-Eq$_{trans}$

Ru	0.6783	-0.1586	-0.6538
C	-1.0291	-2.4305	-1.7960
C	0.2573	-2.0462	-1.4194
C	1.3192	-2.9336	-1.6591
C	1.1368	-4.1870	-2.2572
C	-0.1516	-4.5587	-2.6240
C	-1.2208	-3.6833	-2.3923
H	-1.8803	-1.7799	-1.6360
H	1.9849	-4.8442	-2.4213
H	-0.3309	-5.5235	-3.0858
H	-2.2255	-3.9827	-2.6774
C	3.8489	-0.9910	0.0360
C	4.7031	-2.0867	-0.6523
H	5.3563	-2.6256	0.0319
C	2.5548	-2.3861	-1.1762
N	2.5472	-1.1951	-0.6387
H	4.2492	-0.0021	-0.1901
O	3.7197	-3.0325	-1.1734
C	3.7429	-1.1733	1.5391
C	4.3508	-0.2569	2.4023
C	3.0735	-2.2789	2.0781
C	4.2861	-0.4334	3.7856
H	4.8750	0.6023	1.9917
C	3.0180	-2.4630	3.4588
H	2.5895	-2.9962	1.4232
C	3.6192	-1.5375	4.3166
H	4.7593	0.2876	4.4444
H	2.5143	-3.3352	3.8653
H	3.5758	-1.6821	5.3914
C	2.0057	1.4404	0.2026
C	0.9537	2.4692	0.5717
H	5.2723	-1.7022	-1.5028
C	1.1661	0.7448	-3.6812
N	1.0224	0.4348	-2.5760
C	1.3304	1.1299	-5.0752
H	1.3588	0.2375	-5.7065
H	0.4911	1.7588	-5.3856
H	2.2612	1.6891	-5.2042
C	-0.0681	-0.9778	2.3161
N	0.2877	-0.7035	1.2529
C	-0.5456	-1.3300	3.6394
H	0.2462	-1.8402	4.1937
H	-0.8406	-0.4243	4.1763
H	-1.4171	-1.9803	3.5299
C	-4.9624	-0.7114	0.8965
C	-5.4582	0.3128	0.0783
C	-6.8063	0.6358	0.0582
C	-7.6576	-0.1011	0.8921
C	-7.1632	-1.1257	1.7084
C	-5.7999	-1.4483	1.7200
C	-3.4917	-0.8202	0.7110
C	-4.3297	0.9136	-0.6769
H	-7.1788	1.4318	-0.5770
H	-8.7185	0.1261	0.9081
H	-7.8488	-1.6768	2.3437
H	-5.4079	-2.2401	2.3489
O	-2.6805	-1.5210	1.2764
O	-4.2726	1.8726	-1.4053
O	-1.9519	0.5135	-0.6588
C	-1.3974	1.4462	0.2899
O	-1.9227	1.6562	1.3457
C	-0.1198	1.8431	-0.2996
H	-0.2210	2.2623	-1.2978
N	-3.2197	0.0771	-0.3479
H	2.6653	1.7322	-0.6113
H	2.5054	0.9572	1.0330
H	0.6692	2.3533	1.6206
C	1.2883	3.9134	0.2925
C	1.2346	4.8657	1.3151
C	1.6620	4.3265	-0.9940
C	1.5449	6.2022	1.0611
H	0.9421	4.5583	2.3155
C	1.9722	5.6601	-1.2517
H	1.7102	3.6000	-1.8022
C	1.9146	6.6027	-0.2228
H	1.4952	6.9299	1.8652
H	2.2579	5.9668	-2.2535
H	2.1551	7.6421	-0.4222

Product-IS-Eq$_{trans}$

Ru	-0.2725	0.6482	-0.0499
C	2.0735	2.2350	-1.2478
C	0.7027	2.1308	-1.0075
C	-0.1210	3.2050	-1.4359
C	0.3958	4.3423	-2.0674

C	1.7669	4.4217	-2.2891
C	2.5946	3.3689	-1.8819
H	2.7452	1.4398	-0.9452
H	-0.2717	5.1395	-2.3796
H	2.1920	5.2919	-2.7775
H	3.6646	3.4324	-2.0608
C	-3.3076	1.6874	-0.6592
C	-3.7322	3.1444	-0.9706
H	-3.9826	3.6947	-0.0587
C	-1.5183	2.9561	-1.1797
N	-1.8486	1.8427	-0.5943
H	-3.5364	1.0347	-1.5141
O	-2.5321	3.7509	-1.5462
C	-3.9367	1.0824	0.5715
C	-5.2076	0.5039	0.4744
C	-3.2750	1.0690	1.8037
C	-5.7971	-0.1057	1.5809
H	-5.7334	0.5201	-0.4769
C	-3.8586	0.4456	2.9095
H	-2.3044	1.5396	1.8922
C	-5.1162	-0.1490	2.8000
H	-6.7809	-0.5549	1.4903
H	-3.3316	0.4320	3.8589
H	-5.5685	-0.6341	3.6590
C	-1.2282	-2.9808	2.3175
C	-1.0562	-3.3025	0.8675
H	-4.5368	3.2330	-1.6990
C	-0.2434	-1.2604	-2.5662
N	-0.3792	-0.4980	-1.7050
C	-0.0253	-2.2394	-3.6167
H	0.0255	-1.7464	-4.5913
H	0.9215	-2.7513	-3.4199
H	-0.8428	-2.9648	-3.6161
C	0.5775	2.1353	2.6165
N	0.0999	1.6550	1.6760
C	1.2335	2.7038	3.7823
H	1.3661	3.7813	3.6518
H	0.6403	2.5238	4.6831
H	2.2116	2.2253	3.8864
C	4.9055	-0.4299	0.7972
C	4.7232	-1.2287	-0.3440
C	5.8014	-1.6901	-1.0826
C	7.0843	-1.3321	-0.6456
C	7.2659	-0.5364	0.4912
C	6.1712	-0.0697	1.2320
C	3.5790	-0.0913	1.3767
C	3.2694	-1.4473	-0.5586
H	5.6517	-2.3082	-1.9610
H	7.9523	-1.6796	-1.1958
H	8.2720	-0.2792	0.8052
H	6.3029	0.5451	2.1156
O	3.2695	0.5182	2.3745
O	2.6610	-2.1117	-1.3659
O	1.3299	-0.7159	0.6915
C	0.9358	-1.8715	1.4499
O	1.7580	-2.6176	1.8908
C	-0.5253	-1.9687	1.4487
H	-1.1050	-1.0816	1.1951
N	2.6723	-0.6446	0.4433
H	-2.2136	-2.6889	2.6658
H	-0.6183	-3.5270	3.0289
H	-0.2603	-4.0111	0.6583
C	-2.1343	-3.2801	-0.1542
C	-2.0283	-4.1479	-1.2495
C	-3.2330	-2.4133	-0.0795
C	-2.9972	-4.1526	-2.2533
H	-1.1849	-4.8306	-1.3083
C	-4.1965	-2.4156	-1.0863
H	-3.3495	-1.7328	0.7583
C	-4.0829	-3.2796	-2.1772
H	-2.9088	-4.8444	-3.0858
H	-5.0445	-1.7454	-1.0084
H	-4.8411	-3.2813	-2.9537

TS-OS-Eq$_{trans}$

Ru	-1.3376	0.5232	-0.2310
C	-1.2308	2.4018	2.2938
C	-1.9536	1.5365	1.4650
C	-3.3027	1.2932	1.8396
C	-3.9032	1.8892	2.9570
C	-3.1556	2.7554	3.7464
C	-1.8199	3.0007	3.4125
H	-0.1853	2.6096	2.0903
H	-4.9381	1.6647	3.1959
H	-3.6002	3.2271	4.6164
H	-1.2256	3.6638	4.0356
C	-4.3164	-1.1291	-0.6861
C	-5.6089	-0.9970	0.1814
H	-5.8791	-1.9287	0.6819
C	-4.0034	0.3521	0.9894
N	-3.4044	-0.1926	-0.0206
H	-4.4998	-0.7828	-1.7093
O	-5.2758	-0.0183	1.2070
C	-3.7551	-2.5338	-0.7482
C	-3.7242	-3.2331	-1.9563
C	-3.2762	-3.1550	0.4113
C	-3.2274	-4.5382	-2.0093
H	-4.0959	-2.7574	-2.8599
C	-2.7702	-4.4519	0.3604
H	-3.2945	-2.6126	1.3519
C	-2.7442	-5.1484	-0.8510
H	-3.2279	-5.0789	-2.9512
H	-2.4069	-4.9292	1.2657
H	-2.3608	-6.1633	-0.8880
H	-6.4660	-0.6183	-0.3782
C	-2.5934	2.9852	-1.8092
N	-2.0785	2.0836	-1.2959
C	-3.2402	4.1230	-2.4476
H	-4.1729	3.8063	-2.9230
H	-3.4669	4.8902	-1.7017
H	-2.5848	4.5516	-3.2113
C	-0.4044	-1.8660	1.6642
N	-0.7332	-1.0160	0.9545
C	0.0084	-2.9350	2.5576
H	0.5482	-2.5137	3.4103
H	-0.8674	-3.4778	2.9230
H	0.6674	-3.6140	2.0117
C	5.1887	-1.6887	0.2865
C	4.3434	-2.7853	0.5080
C	4.8206	-3.9682	1.0496
C	6.1827	-4.0250	1.3768
C	7.0255	-2.9299	1.1563
C	6.5371	-1.7390	0.6000
C	4.3929	-0.5836	-0.3085
C	2.9679	-2.4292	0.0706
H	4.1614	-4.8138	1.2139
H	6.5917	-4.9326	1.8082
H	8.0751	-3.0050	1.4207
H	7.1829	-0.8860	0.4233
O	4.7060	0.5423	-0.6218
O	1.9355	-3.0728	0.1182
O	2.0230	-0.3765	-0.8255
C	1.5991	0.4640	0.2349
O	2.0946	0.3906	1.3283
C	0.4508	1.3116	-0.1558
H	0.6040	2.3418	0.1627
N	3.1034	-1.1460	-0.4738
C	0.0823	-3.1470	-2.7298
H	-0.7241	-3.8813	-2.8014
H	0.8019	-3.4730	-1.9722
H	0.5812	-3.0502	-3.6974
C	-0.4657	-1.8715	-2.3016
N	-0.8835	-0.8728	-1.8929
C	1.0053	1.9716	-2.2582
C	2.2251	2.4365	-1.8587
H	0.9197	1.0103	-2.7466
H	0.1381	2.6152	-2.3147
H	3.0719	1.7588	-1.9140
C	2.4987	3.7093	-1.2174
C	3.7850	3.9240	-0.6834
C	1.5235	4.7169	-1.0634
C	4.0889	5.1114	-0.0246
H	4.5295	3.1400	-0.7779
C	1.8302	5.8994	-0.4028
H	0.5268	4.5716	-1.4680
C	3.1143	6.1010	0.1166
H	5.0828	5.2648	0.3832
H	1.0741	6.6702	-0.2916
H	3.3505	7.0273	0.6307

Product-OS-Eq$_{trans}$

Ru	-1.5231	-0.1247	-0.1439
C	-2.2805	2.0498	1.9987
C	-2.5381	0.8203	1.3798
C	-3.6601	0.0949	1.8702
C	-4.4833	0.5559	2.9054
C	-4.1965	1.7851	3.4883
C	-3.0972	2.5227	3.0320
H	-1.4438	2.6658	1.6818
H	-5.3264	-0.0414	3.2391
H	-4.8167	2.1687	4.2915
H	-2.8719	3.4824	3.4893
C	-3.4068	-2.7368	-0.4010
C	-4.5483	-3.1923	0.5541
H	-4.2324	-4.0086	1.2083
C	-3.8394	-1.1581	1.1786
N	-2.9913	-1.4846	0.2439
H	-3.8013	-2.5011	-1.3973

O	-4.8286	-2.0311	1.3957
C	-2.2826	-3.7349	-0.5480
C	-2.0801	-4.3944	-1.7617
C	-1.4319	-4.0053	0.5291
C	-1.0424	-5.3187	-1.9004
H	-2.7348	-4.1836	-2.6031
C	-0.3855	-4.9136	0.3892
H	-1.5800	-3.4827	1.4683
C	-0.1889	-5.5750	-0.8260
H	-0.9044	-5.8390	-2.8435
H	0.2748	-5.1131	1.2278
H	0.6201	-6.2912	-0.9312
H	-5.4698	-3.4640	0.0399
C	-3.5216	1.3092	-2.1707
N	-2.7627	0.7735	-1.4762
C	-4.4631	2.0051	-3.0360
H	-5.2316	1.3133	-3.3923
H	-4.9460	2.8170	-2.4847
H	-3.9391	2.4261	-3.8990
C	0.5558	-1.4001	1.8957
N	-0.2732	-0.9764	1.2097
C	1.6442	-1.9026	2.7160
H	2.1540	-1.0553	3.1827
H	1.2663	-2.5734	3.4918
H	2.3537	-2.4300	2.0721
C	5.8322	0.4210	0.2661
C	5.4131	-0.9023	0.4683
C	6.2057	-1.8161	1.1460
C	7.4419	-1.3655	1.6277
C	7.8589	-0.0441	1.4267
C	7.0551	0.8732	0.7364
C	4.7682	1.1481	-0.4708
C	4.0679	-1.0810	-0.1357
H	5.8760	-2.8385	1.2960
H	8.0880	-2.0504	2.1670
H	8.8208	0.2744	1.8147
H	7.3688	1.8990	0.5774
O	4.6258	2.3173	-0.7342
O	3.3033	-2.0265	-0.0894
O	2.5750	0.4909	-1.2270
C	1.8053	0.9637	-0.1494
O	2.1619	0.8249	0.9922
C	0.6091	1.6622	-0.6363
H	-0.1855	1.7340	0.1137
N	3.8408	0.1231	-0.8284
C	1.5027	-2.7729	-2.7338
H	1.1083	-3.7749	-2.9173
H	2.3268	-2.8264	-2.0168
H	1.8593	-2.3392	-3.6718
C	0.4592	-1.9424	-2.1568
N	-0.3614	-1.2888	-1.6689
C	0.3305	1.8926	-2.1065
C	0.8834	3.0368	-1.3354
H	0.9819	1.3803	-2.8026
H	-0.7111	1.9300	-2.3968
H	1.9437	3.2318	-1.4707
C	0.0705	4.2129	-0.9283
C	0.7060	5.4451	-0.7338
C	-1.3130	4.1235	-0.7126
C	-0.0235	6.5663	-0.3387
H	1.7778	5.5238	-0.8923
C	-2.0388	5.2406	-0.3055
H	-1.8283	3.1776	-0.8391
C	-1.3978	6.4675	-0.1209
H	0.4843	7.5148	-0.1956
H	-3.1058	5.1508	-0.1250
H	-1.9647	7.3377	0.1940
